# Research progress and application of high efficiency organic solar cells based on benzodithiophene donor materials

**DOI:** 10.1002/EXP.20230122

**Published:** 2024-02-29

**Authors:** Congqi Lin, Ruixiang Peng, Jingyu Shi, Ziyi Ge

**Affiliations:** ^1^ Zhejiang Provincial Engineering Research Center of Energy Optoelectronic Materials and Devices Ningbo Institute of Materials Technology and Engineering Chinese Academy of Sciences Ningbo People's Republic of China; ^2^ Faculty of Materials and Chemical Engineering Ningbo University Ningbo People's Republic of China

**Keywords:** benzodithiophene, donor material, molecular design strategy, organic solar cell, photoelectric performance

## Abstract

In recent decades, the demand for clean and renewable energy has grown increasingly urgent due to the irreversible alteration of the global climate change. As a result, organic solar cells (OSCs) have emerged as a promising alternative to address this issue. In this review, we summarize the recent progress in the molecular design strategies of benzodithiophene (BDT)‐based polymer and small molecule donor materials since their birth, focusing on the development of main‐chain engineering, side‐chain engineering and other unique molecular design paths. Up to now, the state‐of‐the‐art power conversion efficiency (*PCE*) of binary OSCs prepared by BDT‐based donor materials has approached 20%. This work discusses the potential relationship between the molecular changes of donor materials and photoelectric performance in corresponding OSC devices in detail, thereby presenting a rational molecular design guidance for stable and efficient donor materials in future.

## Graphical abstract

This summary diagram comprehensively and reasonably reviews the evolution of BDT‐based donor materials over the last 20 years by subdividing the molecular design strategies of main‐chain engineering, side‐chain engineering as well as other engineering based on the detailed molecular structure of BDT‐based polymer donors and small molecule donors.

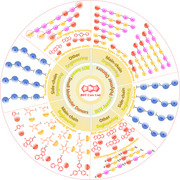

## 1 INTRODUCTION

Solar cells that utilize the photovoltaic effect have gained significant attention due to their ability to convert solar energy into electric energy safely and efficiently. These cells can be categorized into inorganic solar cells, organic solar cells and inorganic‐organic hybrid solar cells. Among them, organic solar cells (OSCs) have emerged as an advanced photovoltaic technology due to their lightweight nature, flexibility, solution process ability and semitransparency. Remarkably, the power conversion efficiency (*PCE*) of single‐junction OSCs has surpassed 19%.^[^ ^1^, ^2^ ^]^ This achievement can be attributed to the advancement of efficient polymer donors like PM6,^[^ ^3^, ^4^, ^5^, ^6^ ^]^ PM7,^[^ ^7^ ^]^ PBDTT1Cl^[^ ^8^ ^]^ and D18,^[^ ^9^ ^]^ among others and small molecular acceptors such as ITIC^[^ ^10^, ^11^ ^]^ and its derivatives,^[^ ^4^ ^]^ Y6,^[^ ^5^ ^]^ L8BO^[^ ^12^ ^]^ and BTP‐eC9,^[^ ^13^ ^]^ among others. These materials exhibit outstanding optical and electrical properties.

The active layer of organic solar cells is critical to the energy conversion process, which involves the mixing of electron donor and acceptor material. As a result, appropriately selecting and matching the materials between polymer or small molecular donors (SMDs) and acceptors can decide the device's photovoltaic performance. In general, active layer materials need to satisfy the following requirements: (i) excellent light harvesting ability and complementary absorption spectrum to achieve high current; (ii) matching energy level structure to achieve efficient exciton dissociation and suppressed non‐radiative energy loss to achieve high voltage simultaneously; and (iii) suitable crystallization and solubility to achieve appropriate phase region size along with elevated phase region purity. Furthermore, as crucial as molecular design engineering, device physics engineering and OSC device application research should all be studied simultaneously.

Since the development of the first polymer donor material, MEH‐PPV,^[^ ^14^ ^]^ in 1995, a large number of conjugated polymer donors have been synthetized through the combination of donor and acceptor units due to their outstanding advantages in molecular chemical structural variety and energy level regulation. Some crucial components were present among these donors during the historical process of OSCs development, such as thiophene (T), thieno^[^ ^3^, ^4^ ^]^ thiophene (TT),^[^ ^15^ ^]^ benzo [1, 2‐*b*: 4, 5‐*b*’] dithiophene (BDT),^[^ ^16^ ^]^ thieno [3, 4‐*c*] pyrrole‐4, 6 (5*H*)‐dione (TPD),^[^ ^17^ ^]^ quinoxaline (Qx)^[^ ^18^ ^]^ etc.

Here, in order to get a clear cognition of the molecular design engineering of OSCs, this review systematically summarizes the progress of the BDT‐based polymer donor materials. Before that, we will introduce OSCs firstly, including the device construction, the working mechanism and some relevant photovoltaic parameters. Next, the detailed summary and classification of BDT‐based polymer donors in terms of molecular design strategies (e.g. main‐chain engineering, side‐chain engineering, multicomponent copolymerization and introducing BDT derivatives as donor units) as well as the molecular design strategies (e.g. main‐chain engineering and side‐chain engineering) for SMDs are presented in turn. Finally, we review the current status of different types of BDT‐based donor materials, highlighting the application of various molecular design strategies in polymer donors and SMDs, thereby proposing potential routines for further optimizing the photoelectric performance in OSC devices.

## 2 OSC DEVICES

**FIGURE 1:**
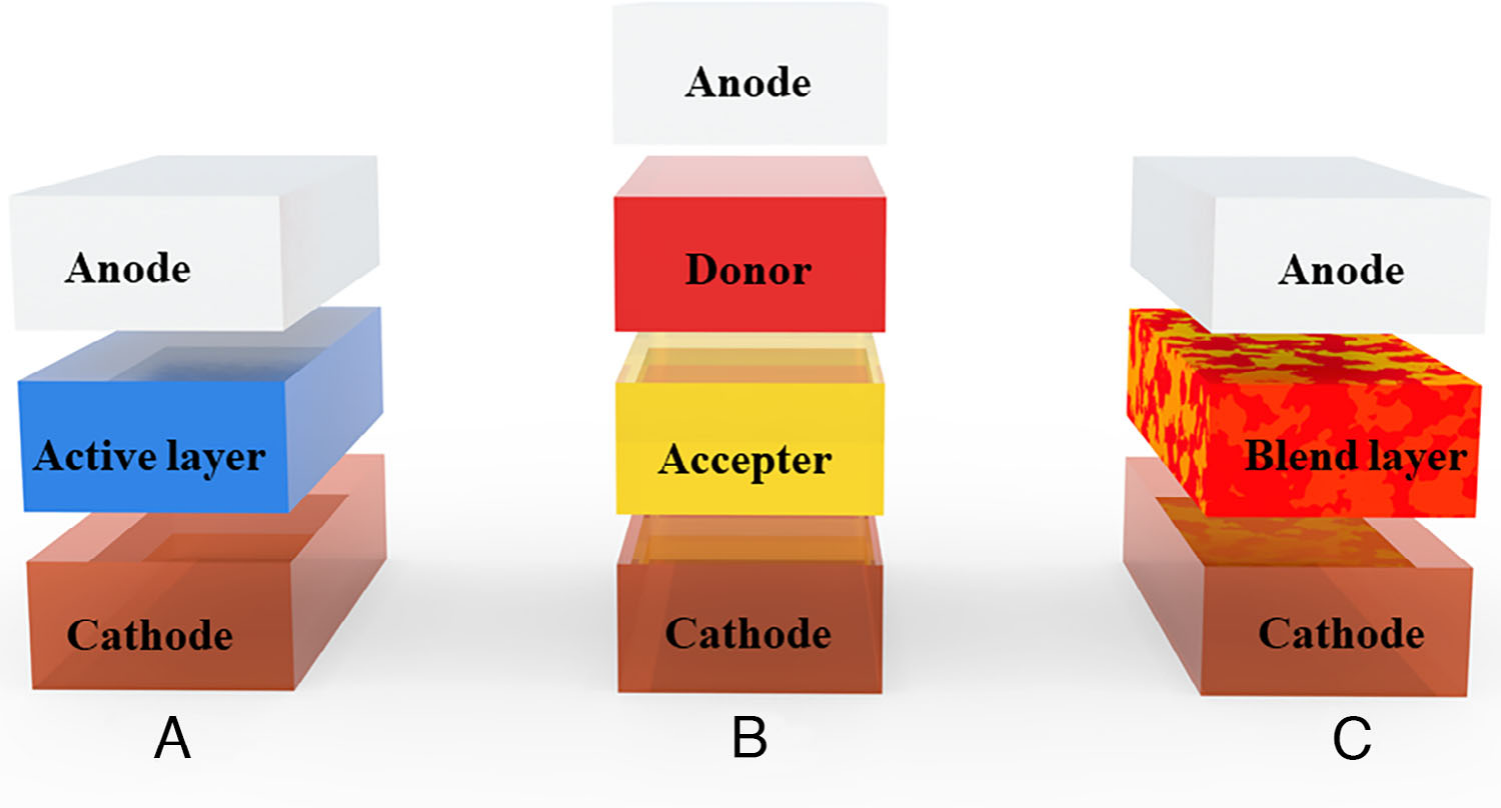
Structural diagram of OSCs: (A) Single‐layer Schottky type; (B) planar heterojunction type; (C) bulk heterojunction type.

In 1958, a Schottky type single active layer between two separate electrodes was used to create the first organic solar cell (Figure 1A).^[^ ^19^ ^]^ However, the output power of this device was very low owing to the Frenkel exciton, a pair of hole and electron, was difficult to dissociate at ambient temperature because of the low dielectric constant of organic semiconductor materials. The planar heterojunction organic solar cell (Figure 1B) was created in 1986 by sequentially evaporating electron donor material and electron acceptor material in order to effectively dissociate active layer‐producing excitons.^[^ ^20^ ^]^ The exciton dissociation, which resulted in the *PCE* of over 1%, was driven by the energy difference between the two active layers. However, the planar heterojunction OSCs’ limited donor‐acceptor interfaces and substantial phase domination resulted in a short exciton lifespan and low *PCE*, prompting research on bulk heterojunction type OSCs (Figure 1C) in 1995.^[^ ^21^ ^]^ As an acceptor material, the fullerene derivative PC_61_BM was equally mixed with a polymer donor material, MEH‐PPV. The relative rising donor‐acceptor contact interface and exciton diffusion length considerably improved the *PCE* to 2.9%. Later, bulk heterojunction organic solar cells gradually became the research standard.

**FIGURE 2:**
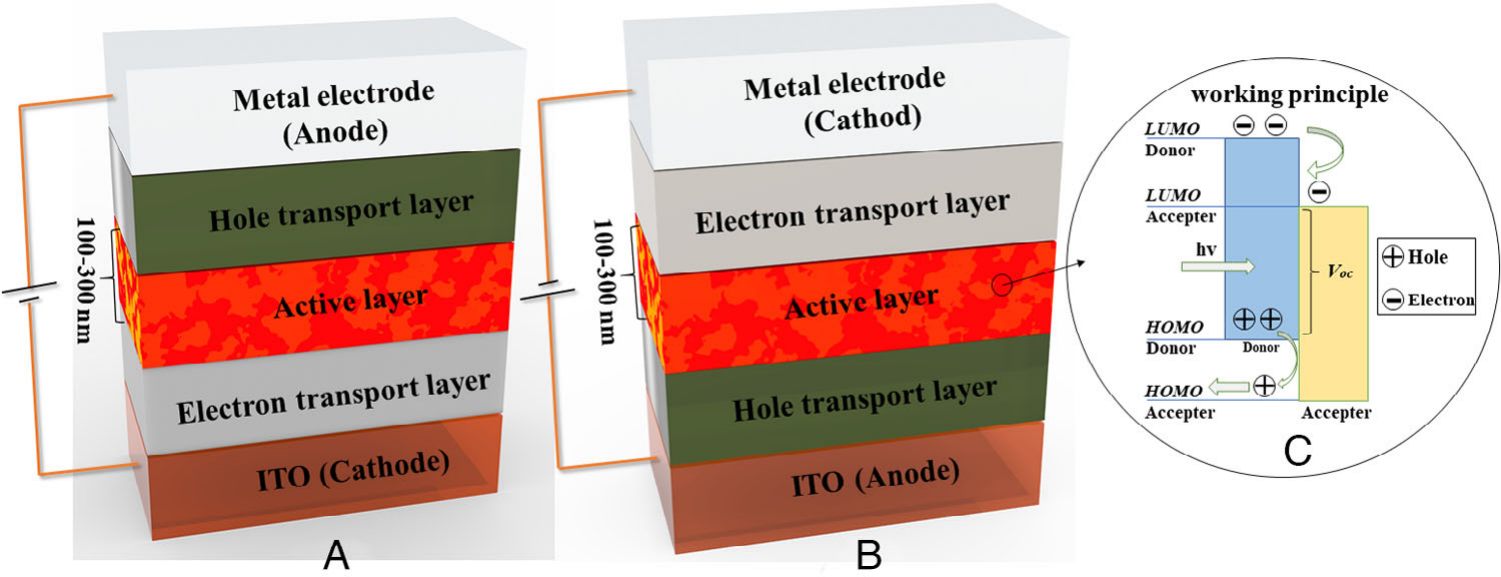
Structural diagram of OSCs: (A) Invert bulk heterojunction solar cell structure; (B) the structure of convention bulk heterojunction OSCs; (C) the working principle of convention bulk heterojunction OSCs.

To examine the working mechanism of the OSCs, the conventional bulk heterojunction type OSC (Figure 2B), with a structure of cathode/electron transport layer/active layer/hole transport layer/anode, was illustrated here. As for invert bulk heterojunction type OSC (Figure 2A), which was normally fabricated based on the structure of anode/hole transport layer/active layer/electron transport layer/cathode. When sunlight illuminates, photons with energy greater than the bandgap of the donor and acceptor materials are collected and absorbed and then electrons stimulated from the highest occupied molecular orbital (HOMO) to the lowest unoccupied molecular orbital (LUMO), leaving holes on the HOMO. Unlike other inorganic OSCs, organic semiconductor materials contain a low dielectric constant, resulting in electron–hole pairs referred to as excitons. Following that, the excitons diffuse to the donor‐acceptor interface and dissociate to free charges in the presence of an electric field (Figure 2A,B). Later, the liberated electrons and holes are transmitted to the cathode and anode via the acceptor and donor materials’ continuing channels, respectively. Finally, charges are collected by an electrode and output to an external circuit, resulting in photoelectric conversion (Figure 2C).

**FIGURE 3:**
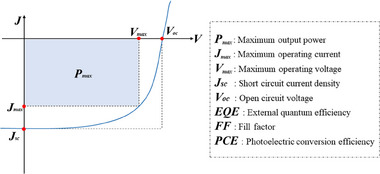
*J–V* curve and photovoltaic parameters.

In general, there are two photocurrent generating pathways in OSC: photo‐induced electron transfers from donors to acceptors and photo‐induced hole transfer from acceptors to donors. As a consequence, three crucial characteristics, *V_oc_*, *J_sc_* and *FF*, which directly determine the *PCE* of OSC devices, are characterized by the output characteristic *J–V* curve (Figure 3). *V_oc_* is mostly related to the energy level difference between the donor's HOMO energy level and the acceptor's LUMO energy level, whereas voltage loss, including radiative and non‐radiative recombination, is primarily caused by poor active layer morphology.^[^ ^22^ ^]^ *J_sc_* is affected by incident light intensity, active layer light absorption capacity and blending phase morphology. The *FF* is directly related to exciton dissociation, carrier mobility and collection, which is inextricably linked to active layer morphology. An optimal nanostructure phase morphology has a domain size of 20–30 nm, which roughly corresponds to the length of exciton diffusion and ensures efficient exciton dissociation into free charges at the donor–acceptor interface.^[^ ^23^ ^]^ Because morphology is the critical connection between organic active materials and device photoelectronic performance, this review will cover not only the donor molecular design strategies linked with the BDT unit, but also the associated device morphological features.

## 3 BDT‐BASED POLYMER DONORS

### 3.1 Main‐chain engineering

#### 3.1.1 D‐A type polymer donors

**FIGURE 4:**
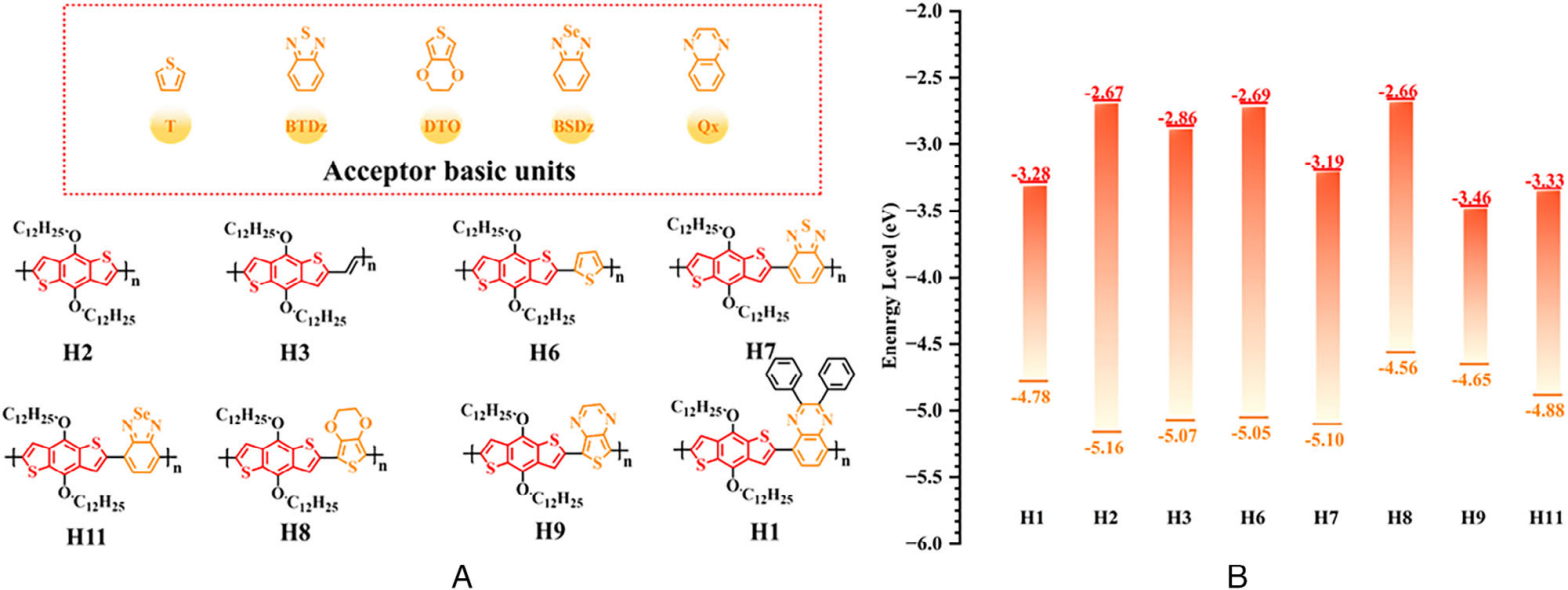
The detailed molecular structure of (A) original D‐A type polymer donors and (B) their detailed HOMO energy level and LUMO energy level.

For the first time, Hou et al.^[^ ^24^ ^]^ used BDT as the core donor unit in 2008. By copolymerizing BDT with thiophene (T), benzo [*c*] [1,2,5] thiadiazole (BT), thieno [3, 4‐*b*] pyrazine (TPZ), 3, 4‐ethylene dioxythiophene (EDOT), benzo [*c*] [1,2,5] selenadiazole (BSe) or 2, 3‐diphenylquinoxaline (DPQ) acceptor units, the detailed molecular structure of relative polymer donors and their HOMO and LUMO energy levels were shown in (Figure 4). Furthermore, after blending them with the acceptor PCBM, the changes of photovoltaic parameters (Table 1) in different types of OSC are sufficient to demonstrate that continuous breakthroughs in photovoltaic performance of OSCs can be achieved through rational molecular design strategies to regulate the molecular structure of polymer donors.

**TABLE 1:** Photovoltaic parameters of OSCs related to Figure 4.

Donor	Acceptor	D/A^(1)^	HOMO [eV] ^(2)^	$E_{g}^{opt}$ [eV] ^(3)^	*V_OC_* [V]	*J_SC_* [mA cm^−2^]	*FF* [%]	*PCE* [%] ^(4)^	Ref.
H1	PCBM	1:1	−4.78	1.63	0.60	1.54	26	0.23	[24]
H2	PCBM	1:1	−5.16	2.13					[24]
H3	PCBM	1:1	−5.07	2.03	0.56	1.16	38	0.25	[24]
H6	PCBM	1:1	−5.05	2.06	0.75	3.78	56	1.60	[24]
H7	PCBM	1:1	−5.10	1.70	0.68	2.97	44	0.90	[24]
H8	PCBM	1:1	−4.56	1.97	0.37	2.46	40	0.36	[24]
H9	PCBM	1:1	−4.65	1.05	0.22	1.41	35	0.11	[24]
H11	PCBM	1:1	−4.88	1.52	0.55	1.05	32	0.18	[24]

(1) Weight ratio; (2) The HOMO energy level of polymer donors; (3) Estimated from the absorption edge in film ($E_{g}^{opt}=1240/\lambda_{onset}$); (4) Average *PCE* of OSCs.

**FIGURE 5:**
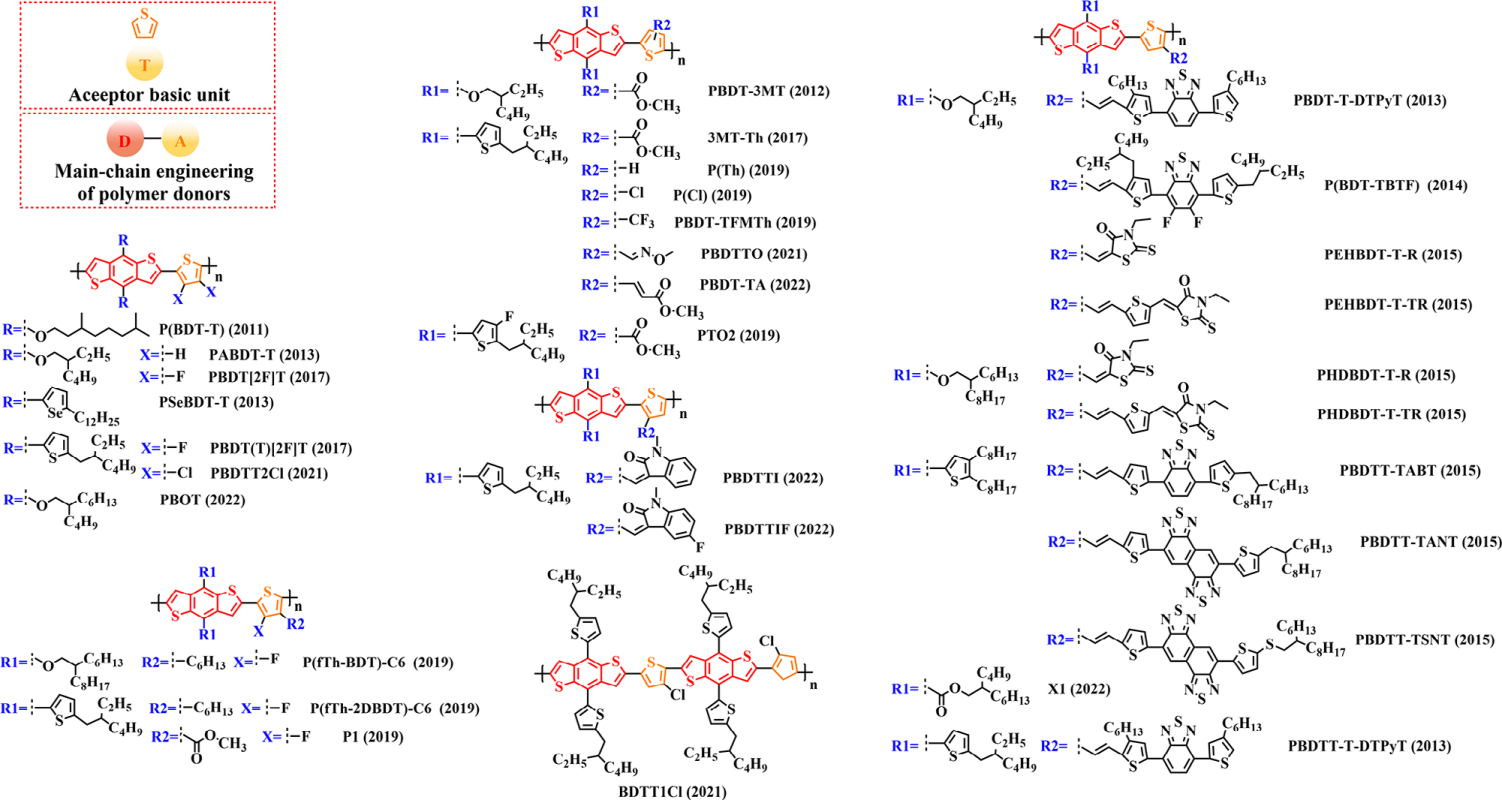
The detailed molecular structure of D‐A type polymer donors synthesized by introducing thiophene as acceptor basic unit.

Thiophene (T), as a unit with weak electron‐donating property, is well recognized to perform a variety of roles in polymer donors (e.g. acceptor basic unit, π‐bridge unit and side‐chain substitution unit, among others). However, in main‐chain engineering, thiophene units generally serve as acceptor units versus BDT units with strong electron‐donating properties. Interestingly, numerous scholars have embraced the molecular design strategy of polymer donors based on the BDT‐T polymer backbone as a straightforward and efficient synthesis route in the past few years. To better understand the advantageous effects of this main‐chain engineering, the precise molecular structure (Figure 5) and photovoltaic characteristics (Table 2) of D‐A polymer donors manufactured with the BDT‐T main‐chain were reviewed.

**TABLE 2:** Photovoltaic parameters of OSCs related to Figure 5.

Donor	Acceptor	D/A^A^	HOMO [eV]^B^	$E_{g}^{opt}$ [eV]^C^	*V_OC_* [V]	*J_SC_* [mA cm^−2^]	*FF* [%]	*PCE* [%]^D^	Ref.
PBDTT1Cl	Y18‐1F	1:1	−5.47	2.03	0.87	26.43	71.35	16.95	[8]
PBDTT2Cl	Y18‐1F	1:1	−5.60	2.01	0.90	20.92	58.60	11.37	[8]
P(BDT‐T)	PC_70_BM	1:1.5	−5.07	2.11	0.75	4.50	60.8	2.05	[25]
PABDT‐T	PC_71_BM	1:2	−5.04	2.10	0.68	7.24	39	2.03	[26]
PSeBDT‐T	PC_71_BM	1:2	−5.14	1.96	0.72	7.73	46	2.73	[26]
P(Th)^a^, ^b^	ITIC‐Th	1:1.25	−5.32	2.03	0.779	8.8	37.3	2.0	[27]
P(Cl)^a^, ^c^	ITIC‐Th	1:1.25	−5.47	1.97	0.859	18.2	68.9	10.7	[27]
PBOT	BTP‐eC9	1:1	−5.22	2.15	0.663	13.97	63.54	5.89	[28]
X1^a^, ^d^	BTP‐eC9	1:1	−5.44	1.85	0.845	27.62	70.69	16.5	[28]
X1^a^, ^e^	Y6	1:1	−5.44	1.85	0.833	26.19	70.50	15.4	[28]
PBDT‐3MT	PC_71_BM	1:1	−5.42		0.86	10.50	50	4.52	[29]
PBDT‐T‐DTPyT	PCBM	1:3	−5.38	1.74	0.78	4.47	38	1.32	[30]
PBDTT‐T‐DTPyT^f^	PCBM	1:3	−5.22	1.59	0.82	6.76	38	2.08	[30]
P(BDT‐TBTF)	PC_70_BM	1:4	−5.36	1.90	0.88	11.23	57.3	5.29	[31]
PEHBDT‐T‐R	PC_71_BM	1:2	−5.48	1.86	0.99	8.61	47.50	3.89	[32]
PHDBDT‐T‐R	PC_71_BM	1:3	−5.56	1.92	0.82	3.92	32.20	0.97	[32]
PEHBDT‐T‐TR	PC_71_BM	1:1.5	−5.45	1.84	0.87	9.36	52.30	4.01	[32]
PHDBDT‐T‐TR	PC_71_BM	1:2	−5.51	1.90	0.95	7.56	48.31	3.34	[32]
PBDT[2F]T^g^	ITIC	3:2	−5.3	2.10	0.96	10.3	55	5.3	[33]
PBDT(T)[2F]T^g^	ITIC	1:1	−5.2	2.00	0.94	16.9	62	9.1	[33]
P(fTh‐BDT)‐C6^g^, ^h^	ITIC‐Th	1:1.5	−5.47	2.08	0.92	3.2	33.5	1.1	[34]
P(fTh‐2DBDT)‐C6^c^, ^h^	ITIC‐Th	1:1.5	−5.51	2.03	0.94	17.1	62.8	11.1	[34]
3MT‐Th(PBDT‐TC)^a^	ITIC	1:1	−5.42	2.00	0.95	17.01	60.08	9.35	[35]
PBDT‐TFMTh^i^	IDT(TCV)_2_	2:1	−5.29	2.07	0.31	2.53	46.20	0.37	[36]
P1(2019)	ITIC‐Th		−5.60	1.95	1.01	17.89	63.05	11.17	[37]
PBDTTO^b^	Y6	1:1.2	−5.60	2.03	0.83	27.03	59	12.99	[38]
PBDTTI^j^	ITIC	1.5:1	−5.59	1.91	0.96	15.60	55	7.50	[39]
PBDTTIF^j^	ITIC	1.5:1	−5.60	1.89	0.98	13.38	55	7.60	[39]
PBDT‐TA^b^	ITIC	1:1	−5.46	2.02	0.96	19.30	55	10.08	[40]
PBDTTTABT^k^	PC_71_BM		−5.54	1.98	0.98	11.12	62.1	6.61	[41]
PBDTTTANT^k^	PC_71_BM		−5.47	1.87	0.95	13.06	65.2	7.84	[41]
PBDTTTSNT^k^	PC_71_BM		−5.50	1.85	0.96	10.25	60.9	5.96	[41]
PTO2	IT‐4F	1:1	−5.59		0.91	21.5	75	14.4	[42]

^A^ Weight ratio.

^B^ The HOMO energy level of polymer donors.

^C^ Estimated from the absorption edge in film ($E_{g}^{opt}=1240/\lambda_{onset}$).

^D^ Average *PCE* of OSCs.

^a^ 0.5% DIO.

^b^ 130°C annealing for 10 min.

^c^ 110°C annealing for 10 min.

^d^ 70°C annealing for 15 min.

^e^ 100°C annealing for 15 min.

^f^ inverted device.

^g^ 160°C annealing for 10 min.

^h^ DIO.

^i^ 120°C annealing.

^j^ 1% CB.

^k^ solvent vapor annealing (SVA) treatment.

Min et al. synthesized a novel polymer donor P(BDT‐T) (2011) based on the polymer backbone of BDT‐T by inserting the side chain (3, 7‐dimethyloctyl)‐*λ* ^1^‐oxidane into the BDT donor unit.^[^ ^25^ ^]^ P(BDT‐T): PC_70_BM‐based OSC reached the *V_oc_* of 0.75 V, *J_sc_* of 4.50 mA cm^−2^, *FF* of 60.8% and *PCE* of 2.05%, demonstrating the application potential of polymer donors with BDT‐T as molecular skeleton. Similarly, in 2013, Byun et al. created PABDT‐T (2013) and PSeBDT‐T (2013) through inserting side chains (2‐ethylhexyl)‐*λ* ^1^‐oxidane and 2‐dodecyl‐selenophene into the BDT donor unit in turn,^[^ ^26^ ^]^ thereby PABDT‐T: PC_71_BM‐based and PseBDT‐T: PC_71_BM‐based OSCs obtained the *V_oc_* of 0.68 and 0.72 V, *J_sc_* of 7.24 and 7.73 mA cm^−2^, *FF* of 39% and 46% and *PCE* of 2.03% and 2.73%, respectively. In addition, Jeon et al. synthesized P(Th) (2019) by introducing the side chain 2‐(2‐ethylhexyl) thiophene into the BDT donor unit,^[^ ^27^ ^]^ while Wang et al. developed X1 (2022) and PBOT (2022) by introducing 2‐butyloctyl acetate and 5‐(methoxymethyl) undecane into the BDT donor unit,^[^ ^28^ ^]^ accordingly. After blending with acceptors, P(Th): ITIC‐Th‐based, PBOT: BTO‐eC9‐based and X1: BTP‐eC9‐based OSCs achieved the *V_oc_* of 0.779, 0.663 and 0.845 V; *J_sc_* of 8.8, 13.97 and 27.62 mA cm^−2^; *FF* of 37.3%, 63.54% and 70.69%; and *PCE* of 2.0%, 5.89% and 16.5%, respectively. Notably, X1 with the substitution of carboxylate side chain considerably increases *J_sc_* and *FF* in X1: BTP‐eC9 based OSC.

Following the reported results of PABDT‐T (2013), Cho et al. synthesized PBDT‐3MT (2012) by inserting the side chain methyl acetate into the thiophene unit.^[^ ^29^ ^]^ Afterward, Kang et al.^[^ ^30^ ^]^ introduced the side chain (*E*)−4‐(4‐hexyl‐5‐(prop‐1‐en‐1‐yl) thiophen‐2‐yl)−7‐(4‐hexylthio‐phen‐2‐yl) benzo [*c*] [1,2,5] thiadiazole into the thiophene unit, synthesizing PBDT‐T‐DTPyl (2013); Shen et al.^[^ ^31^ ^]^ prepared P(BDT‐TBTF) (2014) by introducing the functional side chain (*E*)−4‐(4‐(2‐ethylhexyl)−5‐(prop‐1‐en‐1‐yl) thio‐phen‐2‐yl)−7‐(5‐(2‐ethylhexyl) thio‐phen‐2‐yl)−5, 6‐difluoro‐benzo [*c*] [1,2,5] thiadiazole into the thiophene unit; Chen et al. created PEHBDT‐T‐R (2015) and PEHBDT‐T‐TR (2015) by introducing side chains (*E*)−3‐ethyl‐5‐ethylidene‐2‐thioxothiazolidin‐4‐one and (*Z*)−3‐ethyl‐5‐((5‐((*E*)‐prop‐1‐en‐1‐yl) thiophen‐2‐yl) methylene)−2‐thioxothiazolidin‐4‐one into the thiophene unit in turn^[^ ^32^ ^]^; Firdaus et al. produced PBDT[2F]T (2017) by inserting two fluorine (F) atoms into the thiophene unit.^[^ ^33^ ^]^ After applying for OSC devices, PBDT‐3MT: PC_71_BM‐based, PBDT‐T‐DTpyT: PCBM‐based, P(BDT‐TBTF): PC_70_BM‐based, PEHBDT‐T‐R: PC_71_BM‐based, PEHBDT‐T‐TR: PC_71_BM‐based and PBDT[2F]T: ITIC‐based OSCs obtained the *V_oc_* of 0.86, 0.78, 0.88, 0.99, 0.87 and 0.96 V; *J_sc_* of 10.50, 4.47, 11.23, 8.61, 9.36 and 10.3 mA cm^−2^; *FF* of 50%, 38%, 57.3%, 47.50%, 52.30% and 55%; and *PCE* of 4.52%, 1.32%, 5.29%, 3.89%, 4.01% and 5.3%, in that order. Notably, the utilization of side chain (*E*)−3‐ethyl‐5‐ethylidene‐2‐thioxothiazolidin‐4‐one and “F” atom dramatically increased the *V_oc_* of OSC, but the low *J_sc_* and *FF* continued to be an enormous obstacle. Additionally, P(BDT‐TBTF): PC_71_BM‐based OSC outperformed PBDT‐T‐DTPyT: PCBM‐based OSC in terms of photoelectric property, which can be associated to the substitutions of “F” atom and the solubilized alkyl chain 2‐ethylhexyl. likewise, based on the BDT donor unit with the side chain 7‐(methoxymethyl) pentadecane, Chen et al. synthesized two polymer donors PHDBDT‐T‐R (2015) and PHDBDT‐T‐TR (2015) by introducing side chains (*E*)−3‐ethyl‐5‐ethylidene‐2‐thioxothiazolidin‐4‐one and (*Z*)−3‐ethyl‐5‐((5‐((*E*)‐prop‐1‐en‐1‐yl) thiophen‐2‐yl) methylene)−2‐thioxothiazolidin‐4‐one into the thiophene unit.^[^ ^32^ ^]^ Yu et al. manufactured P(fTh‐BDT)‐C6 (2019) by incorporating the side chain hexyl and a single “F” atom into the thiophene unit.^[^ ^34^ ^]^ After blending with different acceptors, PHDBDT‐T‐R: PC_71_BM‐based, PHDBDT‐T‐TR: PC_71_BM‐based and P(fTh‐BDT)‐C6: ITIC‐based OSCs acquired the *V_oc_* of 0.82, 0.95 and 0.92 V; *J_sc_* of 3.92, 7.56 and 3.2 mA cm^−2^; *FF* of 32.20%, 48.31%, 33.5%; and *PCE* of 0.97%, 3.34% and 1.1%, accordingly. Among them, the substitution of side chains (*Z*)−3‐ethyl‐5‐((5‐((*E*)‐prop‐1‐en‐1‐yl) thiophen‐2‐yl) methyl‐ene)−2‐thioxothiaz‐olidin‐4‐one and “F” atom greatly improved the *V_oc_* of OSC, which can be associated to the lower HOMO energy level as well as the more ideal molecular conjugate structure. Furthermore, a promising decomposition temperature of 5% weight loss (*Td_5%_*) was obtained by PEHBDT‐T‐R (329°C), PHDBDT‐T‐R (327°C), PEHBET‐T‐TR (299°C) and PHDBDT‐T‐TR (316°C), demonstrating the applicability of these polymer donors in high temperature environments. Additionally, P(BDT‐TBTF): PC_70_BM‐based (0.535 nm), PEHBDT‐T‐R: PC_71_BM‐based (5.31 nm), PHDBDT‐T‐R: PC_71_BM‐based (3.17 nm), PEHBDT‐T‐TR: PC_71_BM‐based (3.89 nm) and PHDBET‐T‐TR: PC_71_BM‐based (4.60 nm) are among the blend film morphological characterization of root‐mean‐square (RMS) values. The *FF* for OSCs was 57.3%, 47.5%, 32.2%, 52.30%, and 48.31%, in that order. Therefore, the considerably lower *RMS* indicates the blend film creates a more refined phase separation morphology before the active layer's surface morphology achieves an optimal state, enabling the associated photovoltaic devices to acquire a higher *FF*.

Numerous studies have been conducted on the molecular design strategy of P(Th), presenting an excellent utilization potential. As a result, Kang and co‐workers produced PBDTT‐T‐DTPyT (2013) through incorporating the side chain (*E*)−4‐(4‐hexyl‐5‐(prop‐1‐en‐1‐yl)‐thiophen‐2‐yl)−7‐(4‐hexyl‐thiophen‐2‐yl) benzo‐[*c*] [1,2,5] thiadiazole into the thiophene unit.^[^ ^30^ ^]^ Firdaus et al.^[^ ^33^ ^]^ and Park et al.^[^ ^35^ ^]^ announced two polymer donors PBDT(T)[2F]T (2017) and 3MT‐Th (2017), which were synthesized by introducing “F” atom and side chain methyl acetate into the thiophene unit. In 2019, four polymer donors P(Cl) (2019), PBDT‐TFMTh (2019), P(fTh‐2DBDT)‐C6 (2019) and P1 (2019) have been identified by Jeon et al.,^[^ ^27^ ^]^ Pitchamuthu et al.,^[^ ^36^ ^]^ Yu et al.^[^ ^34^ ^]^ and Tang et al.^[^ ^37^ ^]^ in turn, which were created by further introducing single chlorine (Cl) atom, side chain trifluoro‐*λ* ^3^‐methane, side chain hexyl as well as single “F” atom, and the side chain methyl acetate along with single “F” atom into the thiophene unit, accordingly. He et al. successfully produced PBDTTO (2021) in 2021 via the addition of an additional side chain (*E*)‐acetaldehyde O‐methyl oxime into the thiophene unit.^[^ ^38^ ^]^ Wang et al. reported PBDTT1Cl (2021) and PBDTT2Cl (2021) by introducing single or double “Cl” atoms into the thiophene unit in the same year.^[^ ^8^ ^]^ In 2022, Li et al. investigated PBDTTI (2022) and PBDTTIF (2022) by introducing side chains (*Z*)−3‐ethylidene‐1‐methylindolin‐2‐one and (*Z*)−3‐ethylidene‐5‐fluoro‐1‐methylindolin‐2‐one into the thiophene unit.^[^ ^39^ ^]^ Through adding the side chain methyl (*E*)‐but‐2‐enoate to the thiophene unit, Yuan et al.^[^ ^40^ ^]^ additionally reported PBDT‐TA (2022). After blending with acceptors, PBDTT‐T‐DTPyT: PCBM‐based (2.08%), PBDT(T)[2T]T: ITIC‐based (9.1%), 3MT‐Th: ITIC‐based (9.35%), P(Cl): ITIC‐Th‐based (10.7%), PBDT‐TFMTh: IDT(TCV)_2_‐based (0.37%), P(fTh‐2DBDT)‐C6: ITIC‐Th‐based (11.1%), P1: ITIC‐Th‐based (11.17%), PBDTTO: Y6‐based (12.99%), PBDTT1Cl: Y18‐1F‐based (16.95%), PBDTT2Cl: Y18‐1F‐based (11.37%), PBDTTI: ITIC‐based (7.50%), PBDTTIF: ITIC‐based (7.60%) and PBDT‐TA: ITIC‐based (10.08%) OSCs exhibited a promising *PCE* in turn. Notably, the molecular design strategies of fluorination, chlorination, or introducing carboxylate side chain and other functional groups with appropriate extended conjugate plane were effective in endowing OSCs with a higher *V_oc_*, while the *J_sc_* and *FF* remained low. Furthermore, compared to PBDTT2Cl, PBDTT1Cl with the substitution of single “Cl” atom significantly enhanced the *J_sc_* and *FF* as well as ultimately achieved better *PCE*, demonstrating that polymer donors with an appropriate chlorination degree conducive to optimize the HOMO energy level, exciton dissociation and both hole and electron mobility. Moreover, P(fTh‐2DBDT)‐C6: ITIC‐Th‐based OSC (62.8%) achieved a higher *FF* than 3MT‐Th: ITIC‐based (60.08%), PBDT‐TFMTh: IDT(TCV) based (46.20%) and PBDTTO: Y6‐based (59%) OSCs, which can be attributed to the formation of P(fTh‐2DBDT)‐C6: ITIC‐Th‐based blend film (1.18 nm) with a relatively lower *RMS* than 3MT‐Th: ITIC‐based (1.14 nm), PBDT‐TFMTh: IDT(TCV)‐based (2.67 nm) and PBDTTO:Y6‐based (3.85 nm) blend films, which further proves the above‐conclusion.

Furthermore, based on the main chain BDT‐T, Xu et al. announced three novel polymer donors PBDTT‐TABT (2015), PBDTT‐TANT (2015) and PBDTT‐TSNT (2015),^[^ ^41^ ^]^ which were created by introducing the side chain 5‐methyl‐2,3‐dioctylthiophene into the BDT donor unit as well as (*E*)−4‐(5‐(2‐hexyldecyl) thiophen‐2‐yl)−7‐(5‐(prop‐1‐en‐1‐yl)thiophen‐2‐yl)benzo [*c*] [1, 2, 5] thiadiazole, (*E*)−5‐(5‐(2‐hexyldecyl) thiophen‐2‐yl)−10‐(5‐(prop‐1‐en‐1‐yl) thiophen‐2‐yl) naphtha [1, 2‐*c*: 5, 6‐*c*’] bis ([1, 2, 5] thiadiazole) and (*E*)−5‐(5‐((2‐hexyldecyl) thio) thiophen‐2‐yl)−10‐(5‐(prop‐1‐en‐1‐yl)thiophen‐2‐yl) naphtho [1, 2‐*c*: 5, 6‐*c*’] bis ([1, 2, 5] thiadiazole) into the thiophene unit, in that order. Subsequently, Yao et al. also reported PTO2 (2019) through introducing side chains 2‐(2‐ethylhexyl)−3‐fluoro‐5‐methylthiophene and methyl acetate into the BDT and thiophene units in turn.^[^ ^42^ ^]^ Following the applications for OSC devices, PTO2: IT‐4F‐based, PBDTT‐TABT: PC_71_BM‐based, PBDTT‐TANT: PC_71_BM‐based and PBDTT‐TSNT: PC_71_BM‐based OSCs acquired the *V_oc_* of 0.98, 0.95, 0.96, 0.91 V; *J_sc_* of 11.12, 13.06, 10.25 and 21.5 mA cm^−2^; *FF* of 62.1%, 65.2%, 60.9% and 75%; and *PCE* of 6.61%, 7.84%, 5.96% and 14.4%, respectively. In accordance with the conclusions drawn above, the incorporation of side chains with a greater conjugated plane in the polymer donor optimizes the stacking between molecules, which contributes OSC to obtain an ideal *V_oc_*, while decreased *J_sc_* and *FF* continues to be the primary obstacles to further boosting *PCE*. Notably, in PTO2, the substitution of “F” atom and carboxylate side chain with electron‐withdrawing not only offering effective electron dissociation and hole/electron mobility, guaranteeing OSC with an ideal *V_oc_*.

**FIGURE 6:**
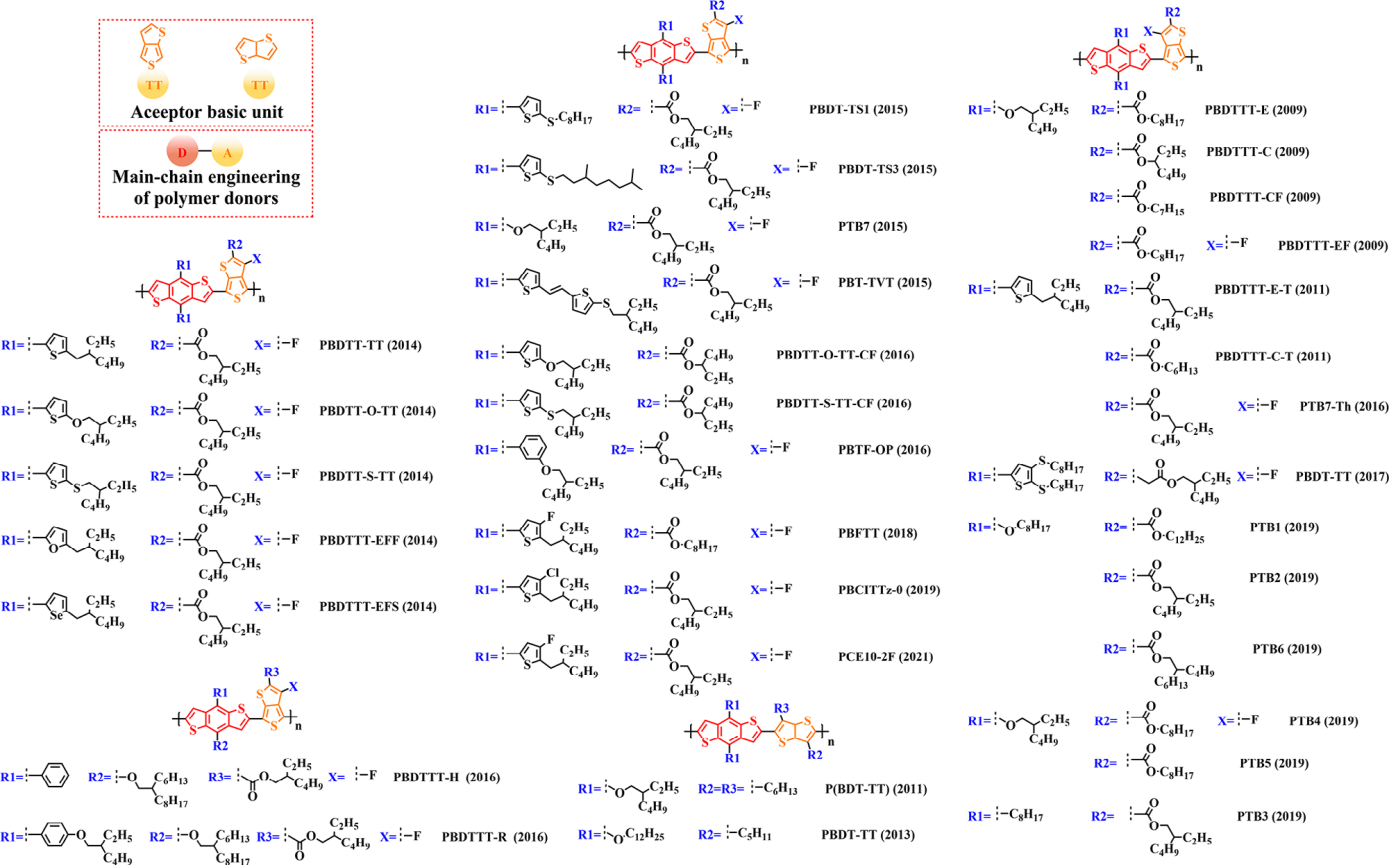
The detailed molecular structure of D‐A type polymer donors synthesized by introducing TT as acceptor basic unit.

Thieno[3,4‐b] thiophene (TT) unit exhibits a wider conjugated plane than thiophene unit, is advantageous for generating better crystals and tighter molecular stacking. Additionally, a variety of polymer donors (e.g. PTB7‐Th,^[^ ^43^ ^]^ PBDT‐TS1^[^ ^44^ ^]^ and *PCE*10‐2F^[^ ^45^ ^]^) have also made substantial strides in recent years based on the main chain BDT‐TT. In this section, the detailed molecular structure of representative polymer donors (Figure 6) and photovoltaic parameters in the corresponding OSC (Table 3) were covered as follows.

**TABLE 3:** Photovoltaic parameters of OSCs related to Figure 6.

Donor	Acceptor	D/A^A^	HOMO [eV]^B^	$E_{g}^{opt}$ [eV]^C^	*V_OC_* [V]	*J_SC_* [mA cm^−2^]	*FF* [%]	*PCE* [%]^D^	Ref.
PBDTTT‐E‐T^B^, ^a^	DCNBT‐TPIC	1:0.7	−5.31		0.69	22.33	63.5	9.78	[13]
P(BDT‐TT)	PC_70_BM	1:1.5	−5.05	2.05	0.73	2.22	33	0.54	[25]
PTB7‐Th^c^	PC_71_BM	1:1.5	−5.22	1.58	0.82	19.1	69.1	10.4	[43]
PBDT‐TS1^d^, ^e^	PC_71_BM	1:1.5	−5.29	1.60	0.80	17.93	69.75	9.79	[44]
*PCE*10‐2F	Y6		−5.47	1.60	0.773	25.18	65.34	12.68	[45]
PBDTTT‐E	PC_70_BM	1:1	−5.01	1.63	0.62	13.2	63	4.8	[46]
PBDTTT‐CF	PC_70_BM	1:1.5	−5.22		0.76	15.2	66.9	7.4	[46]
PBDTTT‐EF									[46]
PBDTTT‐C	PC_70_BM	1:1.5	−5.07	1.60	0.70	15.51	59.2	6.43	[47]
PBDTTT‐C‐T	PC_70_BM	1:1.5	−5.11	1.58	0.74	17.48	58.7	7.59	[47]
PTB1	PC_61_BM	1:1	−4.90	1.58	0.58	12.5	65.4	4.76	[48]
PTB2	PC_61_BM	1:1	−4.94	1.59	0.60	12.8	66.3	5.10	[48]
PTB3	PC_61_BM	1:1	−5.04	1.60	0.74	13.1	56.8	5.53	[48]
PTB4	PC_61_BM	1:1	−5.12	1.63	0.76	9.20	44.5	3.10	[48]
PTB5	PC_61_BM	1:1	−5.01	1.62	0.68	10.3	43.1	3.02	[48]
PTB6	PC_61_BM	1:1	−5.01	1.61	0.62	7.74	47.0	2.26	[48]
PTB7	PC_71_BM	1:1	−5.15	1.63	0.76	13.58	54.51	5.63	[49]
PBDT‐TT	PC_61_BM	1:2	−5.14	2.07	0.61	4.02	46	1.12	[50]
PBDTT‐TT^d^	PC_70_BM	1:1.5	−5.30	1.58	0.77	14.99	63.92	7.35	[51]
PBDTT‐O‐TT^d^	PC_70_BM	1:1.5	−5.18	1.53	0.73	15.17	64.44	7.01	[51]
PBDTT‐S‐TT	PC_70_BM	1:1.5	−5.41	1.57	0.84	15.32	65.49	8.35	[51]
PBT‐TVT	PC_71_BM	1:1.5	−5.23	1.63	0.81	15.16	62.71	8.04	[52]
PBClTTz‐0^d^	PC_71_BM	1:1.5	−5.55	1.68	0.94	12.77	56.98	6.34	[53]
PBFTT	ITIC	1:1.5	−5.47		0.94	16.0	60.5	8.9	[54]
PBDTT‐S‐TT‐CF	PC_70_BM	1:1.5	−5.31		0.890	15.1	71.0	9.58	[55]
PBDTT‐O‐TT‐CF	PC_70_BM	1:1.5	−5.44		0.780	16.5	68.0	8.68	[55]
PBDT‐TS3^d^	PC_71_BM	1:1.5	−5.29	1.61	0.809	16.22	56.0	7.36	[56]
PBDT‐TT^f^	PC_71_BM	1:1.5	−5.56	1.59	0.95	10.51	50.6	4.87	[57]
PBDTTT‐EFF^d^	PC_71_BM	1:1.5	−5.19	1.55	0.693	11.77	64.81	5.28	[58]
PBDTTT‐EFT^d^	PC_71_BM	1:1.5	−5.24	1.58	0.784	16.86	68.16	9.00	[58]
PBDTTT‐EFS^d^	PC_71_BM	1:1.5	−5.29	1.58	0.807	16.57	65.64	8.78	[58]
PBDTTT‐H	PC_71_BM	1:1.5	−5.10	1.68	0.80	17.37	67.5	9.21	[59]
PBDTTT‐R	PC_71_BM	1:1.5	−5.06	1.67	0.82	12.10	57.6	5.58	[59]
PBTF‐OP^g^	PC_71_BM	1:1.5	−5.45	1.62	0.86	16.4	62.2	8.9	[60]

^A^ Weight ratio.

^B^ The HOMO energy level of polymer donors.

^C^ Estimated from the absorption edge in film ($E_{g}^{opt}=1240/\lambda_{onset}$).

^D^ Average *PCE* of OSCs.

^a^ 2% DIO.

^b^ 120°C annealing for 20 min.

^c^ 1.5% NMP and 1.5% DIO.

^d^ 3% DIO.

^e^ CB solvent.

^f^ 0.1% DIO.

^g^ 1.5% DIO.

Chen et al., based on the polymer skeleton BDT‐TT, created two polymer donors, PBDTTT‐E (2009) and PBDTTT‐CF (2009) by introducing the side chain heptyl acetate into the BDT unit as well as functional groups octyl acetate and heptan‐3‐yl acetate into the TT unit, accordingly.^[^ ^46^ ^]^ After co‐blending with the acceptor PC_70_BM, PBDTTT‐E: PC_70_BM‐based and PBDTTT‐CF: PC_70_BM‐based OSCs generated the *V_oc_* of 0.62 and 0.76 V, *J_sc_* of 13.2 and 15.2 mA cm^−2^, *FF* of 63% and 66.9% and *PCE* of 4.8% and 6.43%, respectively. In the same year, the polymer donor PBDTTT‐C (2009) was also reported by Huo et al.^[^ ^47^ ^]^ Additionally, Liang et al. created PTB4 (2019), PTB5 (2019) and PTB7 (2015) by introducing the functional groups 2‐ethylhexyl acetate and “F” atom, octyl acetate and “F” atom, as well as octyl acetate into the TT unit, accordingly.^[^ ^48^, ^49^ ^]^ In comparison to the photoelectric performance of PBDTTT‐E: PC_70_BM‐based OSC, PTB7: PC_71_BM‐based OSC obtained a higher *V_oc_* and *J_sc_*, which can be attributed to the optimized co‐blending morphology formed by introducing solubilized alkyl chain 2‐ethylhexyl. Afterward, Liang et al. announced three polymer donors PTB1 (2019), PTB2 (2019) and PTB6 (2019),^[^ ^48^ ^]^ which were designed through introducing the side chain 1‐methoxyoctane into the BDT unit as well as dodecyl acetate, 2‐ethylhexyl acetate and 2‐butyloctyl acetate into the TT unit, in that order. Among them, the highest *J_sc_* (12.8 mA cm^−2^) and *FF* (66.3%) generated in PTO2: PC_61_BM‐based OSC confirms introducing solubilized alkyl chain 2‐ethylhexyl is conducive to boost the *J_sc_* and *FF* in OSC devices. In addition, Min et al.^[^ ^25^ ^]^ and Li et al.^[^ ^50^ ^]^ altered the orientation of TT unit, synthesizing two polymer donors P(BDT‐TT) (2014) and PBDT‐TT (2011) in turn. Regrettably, P(BDT‐TT): PC_70_BM‐based and PBDT‐TT: PC_70_BM‐based OSCs did not achieve optimum *PCE*. Regarding PTB3 (2019), which was synthesized by introducing the solubilized alkyl chain octyl and side chain 2‐ethylhexyl acetate into the BDT and TT units in turn.^[^ ^48^ ^]^ After blending with the acceptor PC_61_BM, PTB3: PC_61_BM‐based OSC exhibit a similar *PCE* compared with PTB2‐based and PTB7‐based OSCs. Moreover, Feng et al. also characterized the polymer donor PBDTTT‐E‐T (2011)‐based on the main‐chain engineering BDT‐TT.^[^ ^13^ ^]^ This novel polymer donor was synthesized by introducing side chains 2‐(2‐ethylhexyl)−5‐methylthiophene and 2‐ethylhexyl acetate into the BDT and TT units, accordingly. Particularly, the introduction of the side chain alkyl‐thiophene into the BDT donor unit substantially raised the *J_sc_* and *FF* in OSCs than introducing the functional group alkyloxy. In consequence, PBDTTT‐E‐T: DCNBT‐TPIC‐based OSC achieved the *V_oc_* of 0.69 V, *J_sc_* of 22.33 mA cm^−2^, *FF* of 63.5% and *PCE* of 9.78%. Similar to this, Huo et al. synthesized the polymer donor PBDTTT‐C‐T (2011) by incorporating the side chain hexyl acetate into the TT unit,^[^ ^47^ ^]^ which was designed based on the alkyl‐thiophene substituted BDT unit. After blending with the acceptor PC_70_BM, PBDTTT‐C‐T: PC_70_BM‐based OSC exhibited the *V_oc_* of 0.74 V, *J_sc_* of 17.48 mA cm^−2^, *FF* of 58.7% and *PCE* of 7.59%. Subsequently, based on the reported results of PBDTTT‐E‐T, Cui et al.^[^ ^51^ ^]^ and Wan et al.^[^ ^43^ ^]^ further introduced single “F” atom into the TT unit and then reporting two polymer donors PTB7‐Th (2016) and PBDTT‐TT (2017). Notably, PTB7‐Th: PC_71_BM‐based OSC achieved an improvement in photovoltaic performance, while PBDTT‐TT: PC_70_BM‐based OSC did not. This phenomenon reminds us the effect of steric hindrance on molecular stacking of polymer donors will greatly affect the final *PCE*. Subsequently, based on the PTB7‐Th, Yao et al. adjusted the substitution groups on BDT unit, synthesizing PBT‐TVT (2015) by introducing the side chain (*E*)−2‐((2‐ethylhexyl) thio)−5‐(2‐(5‐methylthiophen‐2‐yl) vinyl) thiophene;^[^ ^52^ ^]^ Yuan et al. reported PBClTTz‐0 (2019) by further introducing single “Cl” atom into the alkyl‐thiophene side chain;^[^ ^53^ ^]^ PBFTT (2018)^[^ ^54^ ^]^ and *PCE*10‐2F (2021)^[^ ^45^ ^]^ were designed by further introducing single “F” atom into the alkyl‐thiophene side chain. Interestingly, compared with the PTB7‐Th: PC_71_BM‐based OSC, the *J_sc_* (25.18 mA cm^−2^) presented in *PCE*10‐2F: Y6‐based OSC was significantly enhanced, which can be attributed to the accelerated exciton dissociation and hole/electron mobility induced by suitable fluorination degree in *PCE*10‐2F.

Indeed, functional groups alkyloxy‐thiophene and alkylthio‐thiophene with strong electron‐donating are commonly used as substituted side chains. In 2014, Cui et al. based on the BDT unit with the substitution of 2‐((2‐ethylhexyl) oxy) thiophene, synthesized PBDTT‐O‐TT (2014) by further introducing the side chain 2‐ethylhexyl acetate and “F” atom into the TT unit.^[^ ^51^ ^]^ Similarly, PBDTT‐O‐TT‐CF (2016) was also designed by introducing the side chain heptan‐3‐yl acetate into the TT unit.^[^ ^55^ ^]^ Compared with PBDTTT‐E: PC_70_BM‐based OSC, the *V_oc_* in PBDTT‐O‐TT: PC_70_BM‐based OSC (0.73 V) and PBDTT‐O‐TT‐CF: PC_70_BM‐based OSC (0.78 V) both significantly enhanced. As for the substitution of alkylthio‐thiophene side chain, Cui et al. synthesized PBDTT‐S‐TT (2014) by introducing the side chain 2‐((2‐ethylhexyl) thio)−5‐methylthiophene into the BDT unit as well as 2‐ethylhexyl acetate and single “F” atom into the TT unit in turn.^[^ ^51^ ^]^ In 2015, based on the reported results of PBDTT‐S‐TT, Zhang et al. reported two novel polymer donors PBDT‐TS1 (2015) and PBDT‐TS3 (2015),^[^ ^44^, ^56^ ^]^ which were designed by introducing side chains 2‐methyl‐5‐(octylthio) thiophene and 2‐((3, 7‐dimethyloctyl) thio)−5‐methylthiophene to replace the original substitution of BDT unit. Afterward, Cui et al. prepared PBDTT‐S‐TT‐CF (2016) by introducing side chains 2‐((2‐ethylhexyl) thio)−5‐methylthiophene and heptan‐3‐yl acetate into the BDT and TT units in turn;^[^ ^55^ ^]^ Yu et al. synthesized PBDT‐TT (2017) by introducing the side chain 5‐methyl‐2, 3‐bis (octylthio) thiophene into the BDT unit as well as 2‐ethylhexyl propionate and single “F” atom into the TT unit, respectively.^[^ ^57^ ^]^ Interestingly, after co‐blending with acceptors, the *V_oc_* in such OSC devices obviously enhanced, especially for PBDTT‐S‐TT‐CF: PC_70_BM‐based (0.89 V) and PBDT‐TT: PC_71_BM‐based (0.95 V) OSCs. However, similar to the effect of introducing the side chain alkoxy‐thiophene, the low *J_sc_* and *FF* still hindered the improvement of *PCE*.

To explore more efficient molecular design strategies, a plenty of studies have focused on introducing some unique side chain, such as alkyl‐furan, alkyl‐selenophene, benzene and alkoxy‐benzene. In 2014, based on the reported results of PBDTT‐TT, Zhang et al. designed PBDTTT‐EFF (2014) and PBDTTT‐EFS (2014) by introducing the side chains 2‐(2‐ethylhexyl)−5‐methylfuran and 2‐(2‐ethylhexyl)−5‐methylselenophene to replace the original substitution into the BDT unit.^[^ ^58^ ^]^ After blending with the acceptor PC_71_BM, PBDTTT‐EFS: PC_71_BM‐based OSC obtained a significantly enhanced *V_oc_* (0.807 V) than PBDTT‐TT: PC_70_BM‐based OSC (0.77 V), which proved the reasonable application of introducing side chain alkyl‐selenophene. As for benzene‐based side chains, Liu et al.^[^ ^59^ ^]^ created two novel polymer donors PBDTTT‐H (2016) and PBDTTT‐R (2016) as well as Li et al.^[^ ^60^ ^]^ reported PBTF‐OP (2016). Compared with PBDTT‐TT, the detailed molecular structure of PBDTTT‐H, PBDTTT‐R and PBTF‐OP were designed by further introducing functional side chains benzene and 7‐(methoxymethyl) pentadecane, 1‐((2‐ethylhexyl) oxy)−4‐methylbenzene and 7‐(methoxymethyl) pentadecane, as well as 1‐((2‐ethylhexyl) oxy)−3‐methylbenzene into the BDT unit, in that order. Compared to PBDTT‐TT: PC_71_BM‐based OSC, with the incensement of the conjugated plane in the side chains, the *V_oc_* in PBDTTT‐H: PC_71_BM‐based, PBDTTT‐R: PC_71_BM‐based and PBTF‐OP: PC_71_BM‐based OSCs increased. Furthermore, the best photoelectric performance exhibited by PBDTTT‐H: PC_71_BM‐based OSC also reminds us only appropriately extending the conjugate plane of polymer donors can optimize *V_oc_* and ensure efficient exciton dissociation in corresponding OSC devices.

Among these types of polymer donors, PBDT‐TS1 (370°C) and PBDT‐TS3 (367°C) both obtained a high *Td_5%_*, exhibiting an excellent stability for application. Meanwhile, compared with PBDTTT‐E: PC_70_BM‐based (0.96 nm) and PBDTTT‐C: PC_70_BM‐based (0.92 nm) blend films, the relatively lower *RMS* endows PBDTTT‐CF: PC_70_BM‐based (0.84 nm) OSC with a higher *FF* (66.9%), which can be attributed to the accelerated hole/electron mobility and exciton dissociation induced by more suitable bi‐continuous interpenetrating network morphology. In addition, between the morphology characterizations of PBDT‐TS3: PC_71_BM‐based (0.70 nm) and PBDT‐TT: PC_71_BM‐based (6.69 nm) blend films, the smoother active layer morphology endows PBDT‐TS3: PC_71_BM‐based OSC with a better *FF* (56.0%), which also further verified the above‐conclusion.

**FIGURE 7:**
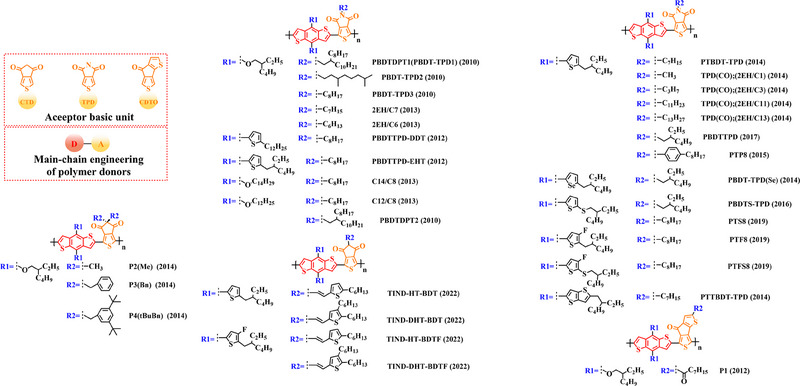
The detailed molecular structure of D‐A type polymer donors synthesized by introducing CTD, TPD, or CDTO as acceptor basic units.

Based on the successful application of TT unit, three novel acceptor units 4H‐cyclopenta [*c*] thiophene‐4, 6 (5*H*)‐dione (CTD), 4*H*, 6*H*‐5*λ* ^2^‐thieno [3, 4‐*c*] pyrrole‐4, 6‐dione (TPD) and 4*H*‐cyclopenta [2, 1‐*b*: 3, 4‐*c*’] dithiophen‐4‐one (CDTO) were developed by different research teams. The emergence of these novel acceptor units with a large conjugated plane further promotes the hole/electron mobility in main‐chain engineering. Therefore, based on the polymer backbones, BDT‐CTD, BDT‐TPD and BDT‐CDTO, a series of polymer donors with excellent photoelectric performance were synthesized, the detailed molecular structure (Figure 7) of representative donor materials and photovoltaic parameters (Table 4) in corresponding OSCs are shown as follows.

**TABLE 4:** Photovoltaic parameters of OSCs related to Figure 7.

Donor	Acceptor	D/A^A^	HOMO [eV]^B^	$E_{g}^{opt}$ [eV]^C^	*V_OC_* [V]	*J_SC_* [mA cm^−2^]	*FF* [%]	*PCE* [%]^D^	Ref.
P1^a^	PC_71_BM		−5.44	1.91	0.86	4.97	55	2.72	[61]
P2(Me)	PC_61_BM				0.95	8.0	52	4.0	[62]
P3(Bn)	PC_61_BM				0.49	2.0	33	0.32	[62]
P4(tBuBn)	PC_61_BM				0.24	0.094	28	0.0063	[62]
TIND‐HT‐BDT^b^, ^c^	Y6BO	3:3	−5.37	1.52	0.75	13.3	50.1	4.84	[63]
TIND‐HT‐BDTF^b^, ^c^	Y6BO	3:3	−5.42	1.58	0.84	16.7	44.4	6.21	[63]
TIND‐DHT‐BDT^b^, ^c^	Y6BO	3:3	−5.26	1.54	0.76	24.8	56.2	10.5	[63]
TIND‐DHT‐BDTF^b^, ^c^	Y6BO	3:4	−5.34	1.60	0.82	22.5	59.3	10.9	[63]
PBDTDPT1^d^	PC_71_BM	1:2	−5.42		0.93	6.58	56	3.42	[64]
PBDTDPT2^d^	PC_71_BM	1:2	−5.44		0.91	10.34	51	4.79	[64]
PBDT‐TPD1^d^	PC_61_BM	1:2	−5.48	1.75	0.87	8.1	56	3.9	[65]
PBDT‐TPD2^d^	PC_61_BM	1:1.5	−5.57	1.70	0.81	9.7	67	5.4	[65]
PBDT‐TPD3^d^	PC_61_BM	1:1.5	−5.4	1.73	0.85	11.5	68	6.6	[65]
C14/C8^e^	PC_71_BM	1:1.5			0.93	8.3	53	3.8	[66]
C12/C8^e^	PC_71_BM	1:1.5			0.89	6.5	45	2.5	[66]
2EH/C7^e^	PC_71_BM	1:1.5			0.97	12.6	70	8.3	[66]
2EH/C6^e^	PC_71_BM	1:1.5			0.96	11.1	62	6.3	[66]
PBDTTPD‐DDT^d^	PC_71_BM	1:1.5	−5.49	1.87	0.86	4.21	47	1.71	[67]
PBDTTPD‐EHT^f^	PC_71_BM	1:2	−5.54	1.85	0.99	8.69	45	3.87	[67]
PTBDT‐TPD^g^	PC_71_BM	1:1	−5.63	1.86	0.94	9.27	63	5.44	[68]
PTTBDT‐TPD^g^	PC_71_BM	1:1	−5.52	1.85	0.91	10.69	62	6.03	[68]
TPD(CO);(2EH/C1)^h^	PC_71_BM	1:1			1.05	10.3	57	5.9	[69]
TPD(CO);(2EH/C3)^h^	PC_71_BM	1:1			1.08	10.7	57	6.3	[69]
TPD(CO);(2EH/C11)^h^	PC_71_BM	1:1			1.06	7.7	57	4.4	[69]
TPD(CO);(2EH/C13)^h^	PC_71_BM	1:1	−5.20		1.05	7.0	56	3.8	[69]
PBDTTPD^g^, ^i^	PC_71_BM	1:1.5	−5.54	1.85	1.02	5.53	58.3	3.29	[70]
PTP8^j^	N_22_00		−5.56		0.978	8.43	52.8	4.20	[71]
PBDT‐TPD(Se)^d^	PC_71_BM	1:2	−5.73	1.85	0.68	9.94	52.76	3.58	[72]
PBDTS‐TPD	PC_70_BM	1:2	−5.51	1.88	0.93	5.23	41.61	2.14	[73]
PTF8^k^	P(NDI2HD‐T)	1.3:1	−5.60	1.84	1.22	5.70	57.5	3.86	[74]
PTS8^k^	P(NDI2HD‐T)	1.3:1	−5.36	1.90	1.03	6.30	56.0	3.45	[74]
PTFS8^k^	P(NDI2HD‐T)	1.3:1	−5.73	1.86	1.09	1.80	32.0	0.51	[74]

^A^ Weight ratio.

^B^ The HOMO energy level of polymer donors.

^C^ Estimated from the absorption edge in film ($E_{g}^{opt}=1240/\lambda_{onset}$).

^D^ Average *PCE* of OSCs.

^a^ 1.5% DIO.

^b^ 0.5% CN.

^c^ 110°C annealing for 10 min.

^d^ 1% DIO.

^e^ 90°C annealing for 10 min.

^f^ 2% DIO.

^g^ 3% DIO.

^h^ 3% 1‐CN.

^i^ 100°C annealing.

^j^ 0.5% DIO.

^k^ DIO.

In 2012, based on the polymer backbone BDT‐CDTO, Zhang et al. designing P1 (2012) by introducing side chains 3‐(methoxymethyl) heptane and nonan‐2‐one into the BDT and CDTO units in turn.^[^ ^61^ ^]^ After blending with the acceptor PC_71_BM, P1: PC_71_BM based OSC obtained the *V_oc_* of 0.86 V, *J_sc_* of 4.97 mA cm^−2^, *FF* of 55% and *PCE* of 2.72%. Indeed, based on the main chain BDT‐CDTO, the introduction of alkyloxy side chain endows P1: PC_71_BM‐based OSC with a high *V_oc_*.

In 2014, based on the polymer backbone BDT‐CTD, Owczarczyk et al. synthesized three novel polymer donors P2(Me) (2014), P3(Bn) (2014) and P4(tBuBn) (2014) by introducing the side chain 3‐(methoxymethyl) heptane into the BDT unit as well as ethane, ethylbenzene, and 1, 3‐di‐tert‐butyl‐5‐ethylbenzene into the CTD unit, in that order.^[^ ^62^ ^]^ After blending with the acceptor PC_61_BM, P2(Me): PC_61_BM‐based, P3(Bn): PC_61_BM‐based and P4(tBuBn): PC_61_BM‐based OSCs obtained the *V_oc_* of 0.95, 0.49 and 0.24 V; *J_sc_* of 8.0, 2.0 and 0.094 mA cm^−2^; *FF* of 53%, 33% and 28%; and *PCE* of 4.0%, 0.32% and 0.0063%, respectively. Notably, P2(Me) with the simple substitution of methyl into the CTD unit endows P2(Me): PC_61_BM‐based OSC with a better photoelectric property than P3(Bn): PC_61_BM‐based and P4(tBuBn): PC_61_BM‐based OSCs, which proved that introducing such functional side chains with an enlarge conjugated plane is not a rational choice. In 2022, based on the polymer backbone BDT‐CTD, D'Orazio‐Colman et al. also synthesized four polymer donors TIND‐HT‐BDT (2022), TIND‐DHT‐BDTF (2022), TIND‐DHT‐BDT (2022) and TIND‐DHT‐BDTF (2022).^[^ ^63^ ^]^ After blending them with the acceptor Y6BO, TIND‐HT‐BDTF and TIND‐DHT‐BDTF with the substitution of 2‐(2‐ethylhexyl)−3‐fluoro‐5‐methylthiophene endow TIND‐HT‐BDTF: Y6BO‐based and TIND‐DHT‐BDTF: T6BO‐based OSCs with a significantly enhanced *V_oc_*, while TIND‐DHT‐BDT and TIND‐DHT‐BDTF with the substitution of (*E*)−2, 3‐dihexyl‐5‐(prop‐1‐en‐1‐yl) thiophene obviously increase the *J_sc_* and *FF* in TIND‐DHT‐BDT: Y6BO‐based and TIND‐DHT‐BDTF: Y6BO‐based OSCs. Consistent with the above conclusion, the introduction of “F” atom efficient decreases the HOMO energy level of donor materials. Furthermore, the introduction of longer alkyl chain also effectively increases the miscibility of donor in the active layer, thus the more balanced and accelerated hole/electron mobility in TIND‐DHT‐BDT: Y6BO‐based OSC (2.18×10^−3^/2.06×10^−3^ cm^2^ V^−1^ s^−1^) and TIND‐DHT‐BDTF: Y6BO‐based OSC (2.06×10^−3^/1.99×10^−3^ cm^2^ V^−1^ s^−1^) can be achieved.

With the presence of the main chain BDT‐TPD, a series of novel D‐A polymer donors began to emerge. In 2010, Zhang et al. reported two polymer donors PBDTDPT1 (2010) and PBDTDPT2 (2010).^[^ ^64^ ^]^ In the same year, Claudia and his coworkers also synthesized PBDT‐TPD1 (2010), PBDT‐TPD2 (2010) and PBDT‐TPD3 (2010).^[^ ^65^ ^]^ Among these donors, due to the substitution of 3‐(methoxymethyl) heptane or 1‐methoxydodecane decreases the HOMO energy level in polymer donors, the *V_oc_* in corresponding OSC devices stay at a high level. Furthermore, after introducing the long alkyl chain into the acceptor unit, the negative effect caused by alkyloxy side chain has been effectively alleviated, which can be verified by PBDT‐TD2 and PBDT‐TPD3. Compared with PBDT‐TPD1, the substitution of 2, 6‐dimethylnonane in PBDT‐TPD2 as well as octyl in PBDT‐TPD3 endow PBDT‐TPD2: PC_61_BM‐based and PBDT‐TPD3: PC_61_BM‐based OSCs with a higher *J_sc_* and *FF* than PBDT‐TPD1: PC_61_BM‐based OSC. Subsequently, based on the reported results of PBDT‐TPD3, Cabanetos et al. designed two novel polymer donors C14/C8 (2013) and C12/C8 (2013) by introducing side chains 1‐methoxytetradecane and 1‐methoxydodecane to replace the original substitution into the BDT unit, respectively.^[^ ^66^ ^]^ Meanwhile, the other two polymer donors 2EH/C7 (2013) and 2EH/C6 (2013) were synthesized by introducing the long alkyl chains heptane and hexane into the TPD unit to replace the original substitution. Consistent with the above conclusion, the introduction of long alkyl chain into the TPD unit effectively enhanced the *J_sc_* and *FF* in OSCs. Therefore, 2EH/C7: PC_71_BM‐based and 2EH/C6: PC_71_BM‐based OSCs also achieved a higher *J_sc_* and *FF* than the PBDT‐TPD1 based OSC.

In addition, based on the main‐chain engineering BDT‐TPD, two polymer donors PBDTTPD‐DDT (2012) and PBDTTPD‐EHT (2012) were reported by Lu et al.^[^ ^67^ ^]^, synthesizing by introducing side chains 2‐dodecyl‐5‐methylthiophene and 2‐(2‐ethylhexyl)−5‐methylthiophene into the BDT unit as well as solubilized alkyl chain octyl into the CTD unit, in that order. Similarly, PTBDT‐TPD (2014) was synthesized by Kim et al.^[^ ^68^ ^]^, which was designed by introducing the side chain 2‐(2‐ethylhexyl)−5‐methylthiophene into the BDT unit as well as heptyl into the TPD unit in turn. In the same year, based on the characterized results of PTBDT‐TPD, Warnan et al. prepared four polymer donors 2EH/C1 (2014), 2EH/C3 (2014), 2EH/C11 (2014) and 2EH/C13 (2014), with the substitution of methyl, propyl, undecyl and tridecyl into the TPD unit, respectively.^[^ ^69^ ^]^ In 2017, by introducing the side chain 3‐ethylheptane into the TPD unit, PBDTTPD (2017) was reported by Gao et al.^[^ ^70^ ^]^ After co‐blending with the acceptor PC_71_BM, the *V_oc_* in these polymer donor: PC_71_BM based OSCs stay at a high level, which can be attributed to the optimized molecular stacking of donor materials within the active layer achieved by introducing the substitution of alkyl‐thiophene side chain. Meanwhile, benefiting such polymer donors prepared based on the main chain BDT‐TPD with faster and more balanced hole/electron mobility than others constructed based on the polymer backbone BDT‐CTD after applying for OSC devices, thus higher *J_sc_* and *FF* have achieved. Moreover, the *J_sc_* and *FF* in photovoltaic devices can be enhanced by introducing the long alkyl chain into the acceptor unit of polymer skeleton, while the overlong alkyl chain with a large steric hindrance cannot. Furthermore, based on the molecular design strategy of PTBDT‐TPD, Kim et al. synthesized PTTBDT‐TPD (2014) by introducing the side chain 2‐(2‐ethylhexyl)−5‐methylthieno [3, 2‐*b*] thiophene into the BDT unit.^[^ ^68^ ^]^ Similarly, Yuan et al. designed the polymer donor PTP8 (2015) by introducing the side chain 1‐methyl‐4‐octylbenzene into the TPD unit.^[^ ^71^ ^]^ Consistent with the above‐conclusion, the introduction with a suitable broaden conjugated plane effectively enhanced the *V_oc_* in OSC devices. In addition, similar to PBDTTPD, Chakravarthi et al. reported PBDT‐TPD(Se) (2014) by introducing the side chain 2‐(2‐ethylhexyl)−5‐methylselenophene into the BDT unit;^[^ ^72^ ^]^ Zhang et al. also prepared PBDTS‐TPD (2016) by introducing the side chain 2‐((2‐ethylhexyl) thio)−5‐methylthiophene into the BDT unit.^[^ ^73^ ^]^ Three novel polymer donors PTF8 (2019), PTS8 (2019) and PTFS8 (2019) were also designed by Li et al.^[^ ^74^ ^]^, based on the reported results of PBDTTPD‐EHT. Among them, these polymer donors were synthesized by introducing side chains 2‐(2‐ethylhexyl)−3‐fluoro‐5‐methylthiophene, 2‐((2‐ethylhexyl) thio)−5‐methylthiophene and 2‐((2‐ethylhexyl) thio)−3‐fluoro‐5‐methylthiophene into the BDT unit, in that order. After co‐blending with acceptors, PBDTS‐TPD: PC_70_BM‐based, PTF8: P(NDI2HD‐T)‐based, PTS8: P(NDI2HD‐T)‐based and PTFS8: P(NDI2HD‐T)‐based OSCs obtained a high *V_oc_* (1.09 V), which could be attributed to the collaboratively optimized molecular energy level of polymer donors induced by the introduction of “F” atoms with electron‐withdrawing and side chain alkylthio with electron‐donating.

Moreover, due to the similar *RMS* exhibited by PBDTTPD‐DDT: PC_71_BM‐based (0.902 nm) and PBDTTPD‐EHT: PC_71_BM‐based (0.657 nm) blend films, which endows them with the *FF* of 47 and 45% in turn. As for PTF8: P(NDI2HD‐T)‐based (2.8 nm), PTS8: P(NDI2HD‐T)‐based (1.5 nm) and PTFS8: P(NDI2HD‐T)‐based (1.7 nm) blend films, with the *FF* of 57.5%, 56.0% and 32.0% after applying for OSC devices, respectively. Indeed, by reducing the surface roughness of the active layer, it is usually beneficial to further improve the *FF* in OSC devices, but before forming an ideal morphology, the slightly increased *RMS* induced by introducing appropriate polar groups into the polymer donor also can make a positive optimization for *FF*, which can be attributed to the accelerated hole/electron mobility.

**FIGURE 8:**
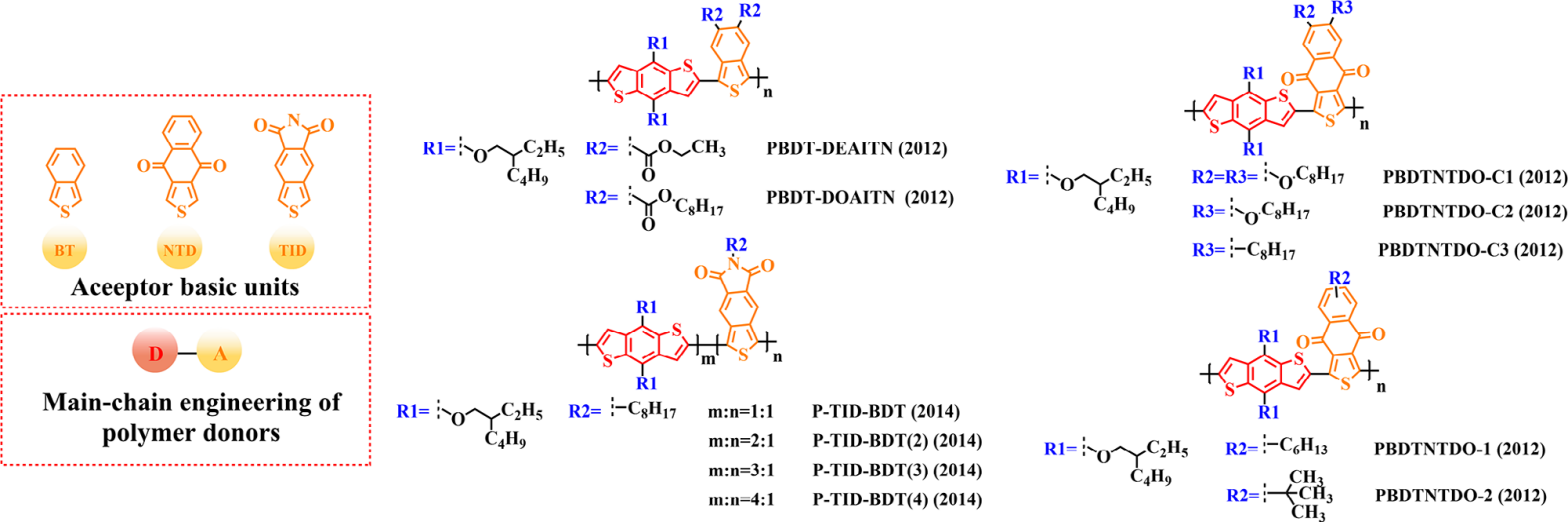
The detailed molecular structure of D‐A type polymer donors synthesized by introducing BT, NTD, or TID as acceptor basic units.

To synthesize ideal donor materials, developing novel acceptor units with a wider π‐π conjugated plane as a rational path receives extensive attention. Therefore, three acceptor units benzo [*c*] thiophene (BT), naphtho [2, 3‐*c*] thiophene‐4, 9‐dione (NTD) and 5*H*, 7*H*‐6λ^2^‐thieno [3, 4‐*f*] isoindole‐5, 7‐dione (TID) have been constructed. Based on three novel polymer backbones BDT‐BT, BDT‐NTD and BDT‐TID, a series of polymer donors with detailed molecular structure (Figure 8) and photovoltaic parameters (Table 5) in corresponding OSC devices were discussed as follows.

**TABLE 5:** Photovoltaic parameters of OSCs related to Figure 8.

Donor	Acceptor	D/A^A^	HOMO [eV]^B^	$E_{g}^{opt}$ [eV]^C^	*V_OC_* [V]	*J_SC_* [mA cm^−2^]	*FF* [%]	*PCE* [%]^D^	Ref.
PBDT‐DEAITN	PC_61_BM	1:1.5	−5.30	1.52	0.64	5.90	33	1.25	[75]
PBDT‐DOAITN	PC_61_BM	1:1.5	−5.42	1.58	0.78	4.65	33	1.20	[75]
PBDTNTDO‐1	PC_70_BM	1:1.5	−5.14	1.82	0.81	4.71	28.7	1.09	[76]
PBDTNTDO‐2	PC_70_BM	1:1.5	−5.19	1.83	0.88	5.67	30.5	1.52	[76]
PBDTNTDO‐C1	PC_71_BM	1:2	−5.19	1.81	0.87	4.42	50.8	1.96	[77]
PBDTNTDO‐C2	PC_71_BM	1:2	−5.23	1.80	0.71	4.21	34.0	1.01	[77]
PBDTNTDO‐C3	PC_71_BM	1:2	−5.27	1.79	0.81	4.93	55.2	2.21	[77]
P‐TID‐BDT^a^	PC_61_BM	1;1		1.40	0.76	4.60	46	1.60	[78]
P‐TID‐BDT(2)^a^	PC_61_BM	1:1		1.54	0.78	6.80	52	2.70	[78]
P‐TID‐BDT(3)^a^	PC_61_BM	1:1		1.64	0.84	6.20	38	1.90	[78]
P‐TID‐BDT(4)^a^	PC_61_BM	1:2		1.70	0.86	6.00	37	1.90	[78]

^A^ Weight ratio.

^B^ The HOMO energy level of polymer donors.

^C^ Estimated from the absorption edge in film ($E_{g}^{opt}=1240/\lambda_{onset}$).

^D^ Average *PCE* of OSCs.

^a^ 4% DIO.

In 2012, based on the main chain BDT‐BT, two polymer donors, PBDT‐DEAITN (2012) and PBDT‐DOAITN (2012) were reported by Long et al.^[^ ^75^ ^]^ At the same year, Cui et al. synthesized two polymer donors, PBDTNTDO‐1 (2012) and PBDTNTDO‐2 (2012) based on the main‐chain engineering BDT‐NTD.^[^ ^76^ ^]^ Similarly, Chen et al. also synthesized three polymer donors, PBDTNTDO‐C1 (2012), PBDTNTDO‐C2 (2012) and PBDTNTDO‐C3 (2012).^[^ ^77^ ^]^ In 2014, based on the polymer backbone BDT‐TID, Braunecker et al. reported four polymer donors, P‐TID‐BDT (2014), P‐TID‐BDT(2) (2014), P‐TID‐BDT(3) (2014) and P‐TID‐BDT(4) (2014),^[^ ^78^ ^]^ which were designed by adjusting the ratio of donor and acceptor unit from 1:1, 1:2, 1:3 to 1:4 in subsequence. After applying for OSC devices, due to the wide conjugated plane optimized the molecular stacking of such polymer donors, which endows corresponding OSC devices with a high *V_oc_*. In addition, it is also worth noting that the photovoltaic performance can be optimized by changing the ratio of donor and acceptor units in D‐A type polymer donors, which provides another reasonable molecular design strategy to explore further optimization in OSC devices. As for PBDTNTDO‐C1: PC_71_BM‐based (1.76 nm), PBDTNTDO‐C2: PC_71_BM‐based (3.22 nm) and PBDTNTDO‐C3: PC_71_BM‐based (2.90 nm) blend films, which obtained the *FF* of 50.8%, 34.0% and 55.2% after applying for OSC devices in turn. Among them, the highest *FF* achieved by PBDTNTDO‐C3: PC_71_BM‐based OSC can be attributed to the formation of the most suitable active layer morphology.

**FIGURE 9:**
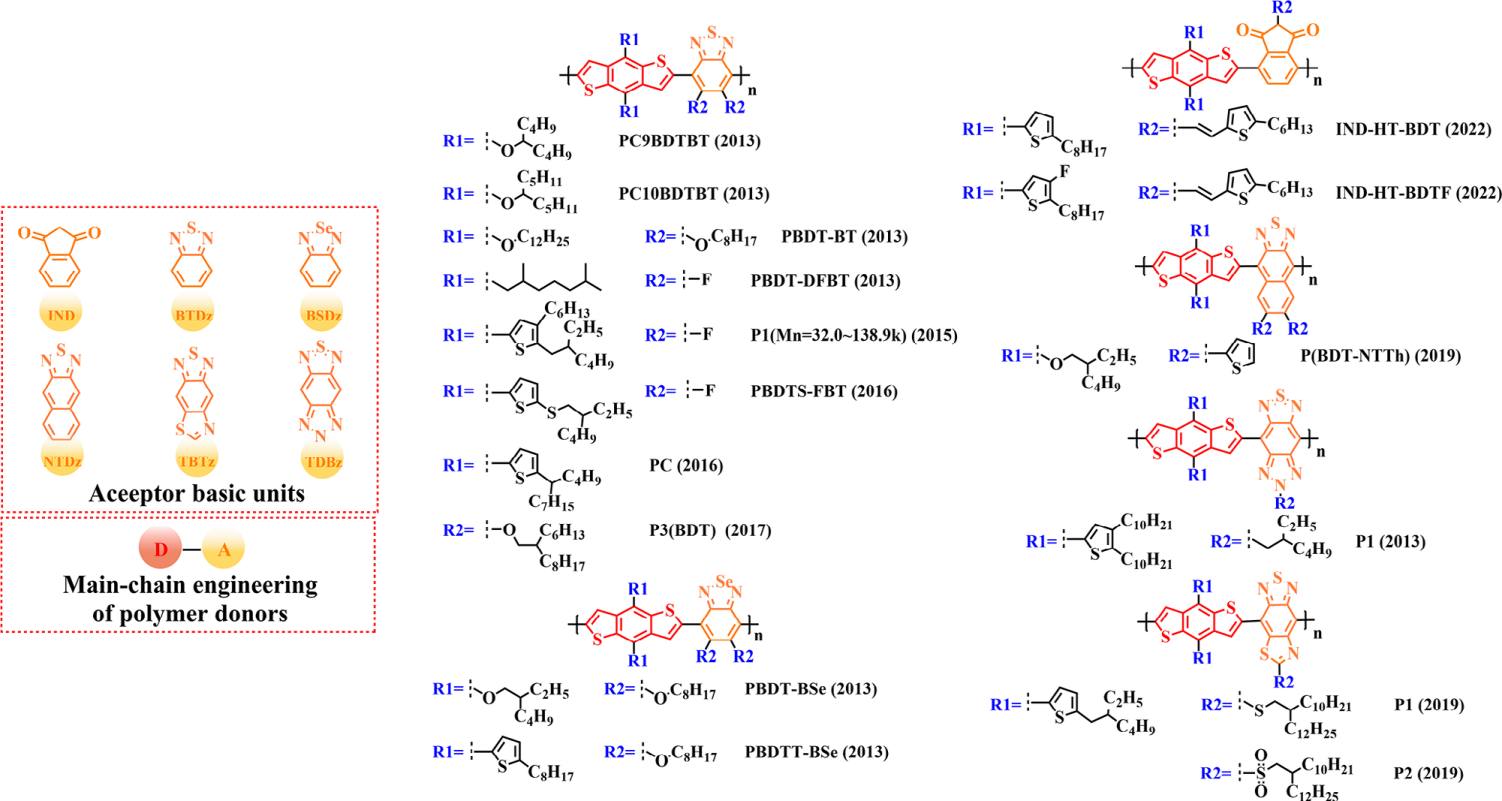
The detailed molecular structure of D‐A type polymer donors synthesized by introducing IND and its derivatives as acceptor basic units.

Based on the acceptor unit BT, considering that the introduction of sulphur (S), nitrogen (N) or oxygen (O) atoms can effectively enhance the electron‐withdrawing of acceptor unit as well as extending the conjugated plane by introducing another thiophene or benzene unit, a series of efficient acceptor units (e.g. 1*H*‐indene‐1, 3 (2*H*)‐dione (IDD), benzo [*c*] [1, 2, 5] thiadiazole (BTDz) and thiazolo [4′, 5′: 4, 5] benzo [1, 2‐*c*] [1, 2, 5] thiadiazole (TBTz)) have been successfully constructed. By introducing such effective acceptor units, a series of polymer donors synthesized based on such novel main‐chain engineering were emerging. Among them, the detailed molecular structure (Figure 9) of representative polymer donors and photovoltaic parameter (Table 6) in corresponding OSC devices were shown as follows.

**TABLE 6:** Photovoltaic parameters of OSCs related to Figure 9.

Donor	Acceptor	D/A^A^	HOMO [eV]^B^	$E_{g}^{opt}$ [eV]^C^	*V_OC_* [V]	*J_SC_* [mA cm^−2^]	*FF* [%]	*PCE* [%]^D^	Ref.
PBDT‐BT	PC_61_BM	1:3	−5.08	1.72	0.64	1.41	42	0.38	[50]
PBDTS‐FBT	PC_70_BM	1:1	−5.49	1.73	0.80	6.65	30.30	1.42	[73]
PC9BDTBT^a^	PC_61_BM	1:1.5			0.887	6.18	40.8	2.24	[79]
PC10BDTBT^a^	PC_61_BM	1:1.5			0.913	4.44	38.4	1.56	[79]
PC^b^	PC_71_BM	1:2	−5.71	1.77	0.861	6.914	37.974	2.262	[80]
P3(BDT)^c^	PC_71_BM	1:1.5	−5.77	1.83	1.01	8.97	70	6.19	[81]
PBDT‐DFBT									[82]
P1‐32.0k	PC_71_BM	1:2	−5.52	1.69	0.92	7.4	47	3.35	[83]
P1‐72.9k	PC_71_BM	1:2	−5.52	1.67	0.89	9.9	45	4.10	[83]
P1‐138.9k	PC_71_BM	1:2	−5.49	1.67	0.92	8.6	48	3.80	[83]
PBDT‐BSe^c^	PC_71_BM	1:1	−5.27		0.76	7.01	46	2.45	[85]
PBDTT‐BSe^c^	PC_71_BM	1:1	−5.31		0.77	11.03	42	3.57	[85]
IND‐HT‐BDT	Y6BO	3:5	−5.44	1.86	0.88	5.80	40.0	2.05	[86]
IND‐HT‐BDTF	Y6BO	3:4	−5.47	1.87	0.82	13.21	59.22	6.38	[86]
P1(2013)	PC_61_BM		−5.12	0.96	0.51	0.47	32	0.1	[87]
P1(2019)	PC_71_BM	1:1.2	−5.26		0.78	12.1	65	5.97	[88]
P2(2019)	PC_71_BM	1:1	−5.35		0.79	3.2	45	1.12	[88]
P(BDT‐NTTh)	PC_71_BM	1:1	−5.34	1.55	0.67	2.04	25.2	0.34	[89]

^A^ Weight ratio.

^B^ The HOMO energy level of polymer donors.

^C^ Estimated from the absorption edge in film ($E_{g}^{opt}=1240/\lambda_{onset}$).

^D^ Average *PCE* of OSCs.

^a^ 2% CN.

^b^ 5% DIO.

^c^ 3% DIO.

In 2013, based on the main chain of BDT‐BTDz, Khantha et al. synthesized PC9BDTBT (2013) and PC10BDTBT (2013) by introducing side chains 5‐methoxynonane and 6‐methoxyundecane into the BDT unit in turn.^[^ ^79^ ^]^ Similarly, Hong et al.^[^ ^80^ ^]^ and Kini et al.^[^ ^81^ ^]^ also prepared PC (2016) and P3(BDT) (2017) by introducing side chains 2‐(dodecan‐5‐yl)−5‐methylthiophene and 7‐(methoxymethyl) pentadecane into the BDT unit, respectively. After co‐blending with acceptors, polymer donor: acceptor‐based OSCs obtained a promising *V_oc_*, especially for P3(BDT): PC_71_BM‐based OSC (1.01 V). Interestingly, PC(BDT): PC_71_BM‐based OSC also obtained an enhanced *FF* (70%), which can be attributed to the optimized blending morphology induced by the substitution of solubilized alkyl chain 2‐hexyldecyl. Furthermore, based on the reported results of PC9BDTBT, Li et al. designed PBDT‐BT (2013) by introducing side chains 1‐methoxydodecane and 1‐methoxyoctane into the BDT and BTDz units, respectively.^[^ ^50^ ^]^ compared with the PC9BDTBT: PC_61_BM‐based OSC, the further introduction of alkyloxy side chain into the BTDz unit did not endow PBDT‐BT: PC_61_BM‐based OSC with higher *J_sc_* and *FF*. Afterward, based on the fluorinated BT unit, Dou et al.^[^ ^82^ ^]^, Xiao et al.^[^ ^83^ ^]^ and Zhang et al.^[^ ^73^ ^]^ explored three polymer donors PBDT‐DFBT (2013), P1 (2015) and PBDTS‐FBT (2016) by further introducing side chains 2,6‐dimethyloctane, 2‐(2‐ethylhexyl)−3‐hexyl‐5‐methylthiophene and 2‐((2‐ethylhexyl) thio)−5‐methylthiophene into the BDT unit, in that order. After applying for OSC devices, P1 with different molecular weight presented various of photoelectric performance in OSC. Compared with P1 (*Mn *= 32.0 k): PC_71_BM‐based and P1 (*Mn *= 138.9 k): PC_71_BM‐based OSCs, P1(*Mn *= 72.9 k): PC_71_BM‐based OSC presented a better photoelectric performance, with the *V_oc_* of 0.89 V, *J_sc_* of 9.9 mA cm^−2^, *FF* of 45% and *PCE* of 4.10%. Notably, optimizing the photovoltaic performance of OSC devices also can be achieved by adjusting the polymer degree of polymer donors, which deserves further attention.

Considering the large π‐overlap of selenium (Se)‐based materials with strong interaction between “Se” and “Se” atoms, which not only extends the π‐orbitals of polymer donors but also accelerates hole/electron mobility in corresponding OSCs.^[^ ^84^ ^]^ Therefore, based on the acceptor unit BTDz, benzo [*c*] [1, 2, 5] selenadiazole (BSDz) was constructed by introducing the “Se” atom to replace the “S” atom. Subsequently, two polymer donors PBDT‐BSe (2013) and PBDTT‐BSe (2013) were reported by Shin and co‐workers,^[^ ^85^ ^]^ which were designed by introducing the side chain 1‐methoxyoctane into the BSDz unit as well as 3‐(methoxymethyl) heptane and 2‐methyl‐5‐octylthiophene into the BDT unit, respectively. Notably, compared with the PBDT‐BT: PC_61_BM‐based OSC, the main‐chain engineering BDT‐BSDz with a better hole/electron mobility endows PBDT‐BSe: PC_71_BM‐based OSC with a higher *J_sc_* (7.01 mA cm^−2^) and *FF* (46%). Furthermore, the substitution of alkyl‐thiophene side chain not only extended the conjugated plane of PBDTT‐BSe, but also enhanced the electron‐donating of BDT unit. Therefore, the *V_oc_* (0.77 V) and *J_sc_* (11.03 mA cm^−2^) in PBDTT‐BSe: PC_71_BM‐based OSC achieved a further breakthrough. Similarly, by introducing the cyclopent‐4‐ene‐1,3‐dione unit with electron‐withdrawing to replace the thiophene unit in BTDz, the novel acceptor unit IDD was also successfully constructed. In 2022, IND‐HT‐BDT (2022) and IND‐HT‐BDTF (2022) were reported by Nisa et al.,^[^ ^86^ ^]^ which were synthesized by introducing the side chain (*E*)−2‐hexyl‐5‐(prop‐1‐en‐1‐yl) thiophene into the IDD unit as well as 2‐methyl‐5‐octylthiophene and 3‐fluoro‐5‐methyl‐2‐octylthiophene into the BDT unit, respectively. After blending with the acceptor Y6BO, IND‐HT‐BDT: Y6BO‐based and IND‐HT‐BDTF: Y6BO‐based OSCs both obtained a high *V_oc_*. Meanwhile, the substitution of fluorinated alkyl‐thiophene further endowed IND‐HT‐BDTF: Y6BO‐based OSC with a higher *J_sc_* (13.21 mA cm^−2^) and *FF* (59.22%), which can be attributed to the accelerated hole/electron mobility within the active layer induced by introducing “F” atom with electron‐withdrawing and small steric hindrance.

In addition, to further extend the conjugated plane and electron‐withdrawing of BTDz unit, three novel acceptor units naphtho [2, 3‐*c*] [1, 2, 5] thiadiazole (NTDz), TBTz, or 5*H*‐[1, 2, 5] thiadiazolo [3, 4‐*f*] benzotriazole (TDBz) were also successfully prepared. In 2013, based on the polymer backbone BDT‐TDBz, Dong et al. synthesized P1 (2013) by introducing side chains 2, 3‐didecyl‐5‐methylthiophene and 2‐ethylhexyl into the BDT and TDBz units in turn.^[^ ^87^ ^]^ Nakamura et al. prepared P1 (2019) and P2 (2019) by introducing the side chain 2‐(2‐ethylhexyl)−5‐methylthiophene into the BDT unit as well as (2‐decyltetradecyl) (methyl) sulphane and 11‐((methylsulphonyl) methyl) tricosane into the TBTz unit, in that order.^[^ ^88^ ^]^ At the same year, based on the main chain BDT‐NTDz, Ratha et al. also synthesized P(BDT‐NTTh) (2019) by introducing side chains 3‐(methoxymethyl) heptane and 2‐methylthiophene into the BDT and NTDz units in turn.^[^ ^89^ ^]^ Notably, after blending with acceptors, P1(2019): PC_71_BM‐based OSC exhibited the best photoelectric performance, with the *V_oc_* of 0.78 V, *J_sc_* of 12.1 mA cm^−2^, *FF* of 65% and *PCE* of 5.97%. However, compared with P1(2019): PC_71_BM‐based OSC, the *J_sc_* and *FF* in P2(2019): PC_71_BM‐based OSC significantly decreased. Therefore, although the introduction of aldehyde groups endows the functional side chain alkylthio with a strong electron‐withdrawing, the larger steric hindrance and the offset of electron donating‐withdrawing effect by groups aldehyde and alkylthio are not beneficial to the *J_sc_* and *FF* in corresponding OSC devices. As for P(BDT‐NTTh) and P1(2013), the substitution of thiophene‐based side chains dispersed the electron density of polymer donor, thus the low *J_sc_* and *FF* obstacle the further improvement of *PCE*.

**FIGURE 10:**
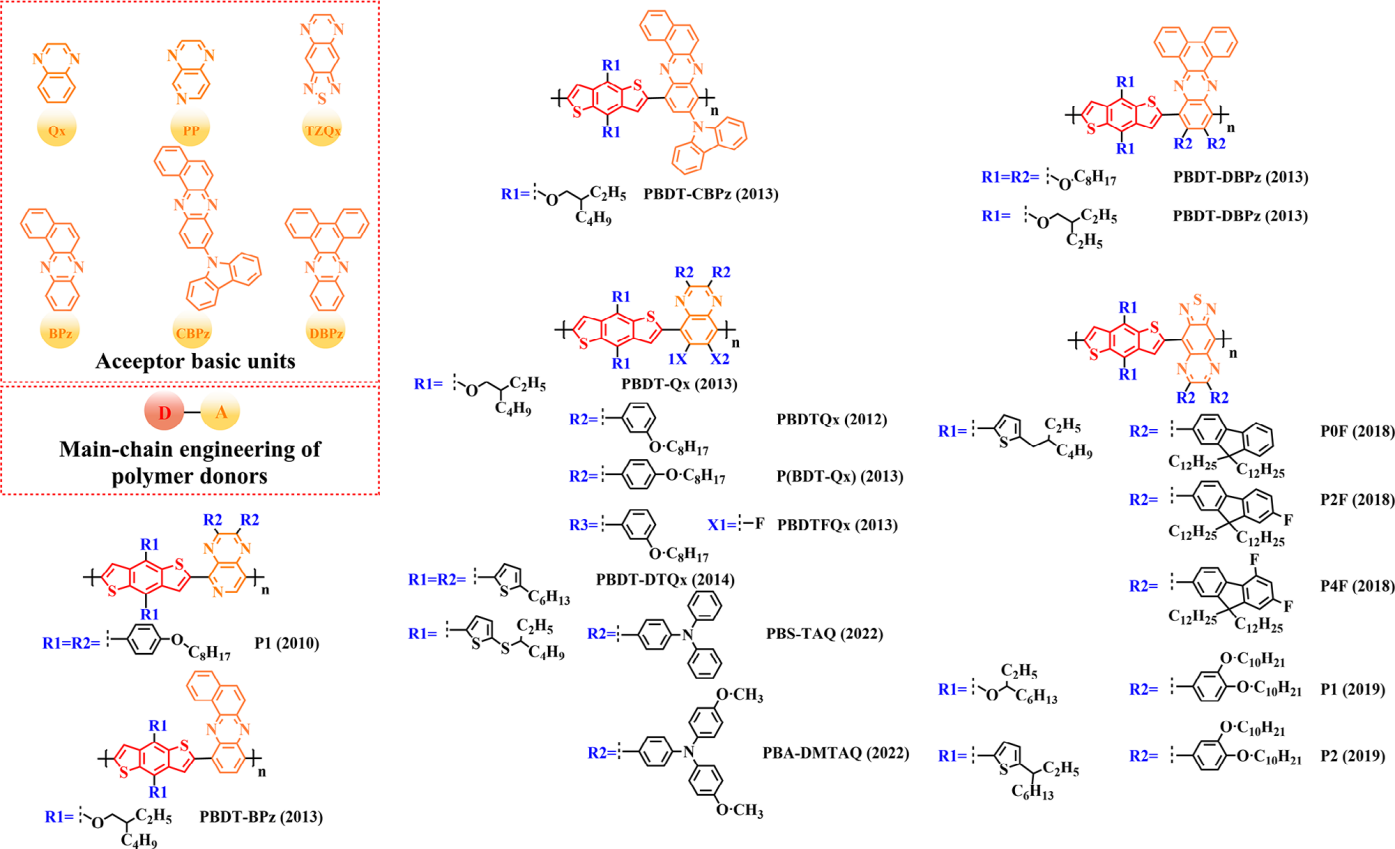
The detailed molecular structure of D‐A type polymer donors synthesized by introducing Qx and its derivatives as acceptor basic units.

In recent years, with the emergence of quinoxaline (Qx) based acceptor units, a serious of novel acceptor units (e.g., pyrido [3, 4‐*b*] pyrazine (PP), benzo [*a*] phenazine (BPz), 9‐(9*H*‐carbazol‐9‐yl) benzo [*a*] phenazine (CBPz) and dibenzo [*a*, *c*] phenazine (DBPz)) with wide conjugated plane and enhanced electron‐withdrawing are constantly emerging. Therefore, to explore the potential advantage of such acceptor units after constructing main‐chain engineering, the detailed molecular structure (Figure 10) of polymer donors and photovoltaic parameters (Table 7) in corresponding OSCs were discussed as follows.

**TABLE 7:** Photovoltaic parameters of OSCs related to Figure 10.

Donor	Acceptor	D/A^A^	HOMO [eV]^B^	$E_{g}^{opt}$ [eV]^C^	*V_OC_* [V]	*J_SC_* [mA cm^−2^]	*FF* [%]	*PCE* [%]^D^	Ref.
PBDT‐Qx	PC_71_BM	1:1.5	−5.19	1.80	0.65	6.30	37	1.54	[90]
PBDT‐BPz	PC_71_BM	1:1.5	−5.25	1.55	0.72	7.74	37	2.04	[90]
PBDT‐CBPz	PC_71_BM	1:1.5	−5.34	1.54	0.78	9.68	51	3.87	[90]
PBDT‐DBPz	PC_71_BM	1:1.5	−5.26	1.67	0.70	7.63	37	2.01	[90]
PBDTQx^a^	PC_61_BM	1:5	−5.26	1.78	0.80	2.67	47	1.01	[91]
P(BDT‐Qx)	PC_71_BM		−5.41	1.78	0.77	5.0	50.3	1.9	[92]
PBDTFQx	PC_61_BM	1:2	−5.28		0.77	2.46	54	1.02	[93]
PBDT‐DTQx^b^	PC_70_BM	1:2.5	−5.35	1.72	0.83	11.71	40	3.90	[94]
PBS‐TAQ	PC_71_BM	1:1.5	−5.35	1.77	0.85	12.64	47.93	5.24	[95]
PBS‐DMTAQ	PC_71_BM	1:1.5	−5.15	1.74	0.78	7.53	46.53	2.74	[95]
PBDT‐DBPz	PC_71_BM	1:2	−5.20	1.77	0.82	2.10	28.1	0.46	[96]
P1	PC_70_BM	1:4	−5.22	1.58	0.74	4.69	34.5	1.20	[97]
P0F^c^	PC_71_BM	1:2	−5.12	1.04	0.58	13.16	66	4.95	[98]
P2F^c^	PC_71_BM	1:2	−5.20	1.09	0.65	14.23	69	6.31	[98]
P4F^c^	PC_71_BM	1:2	−5.29	1.14	0.69	15.96	74	8.03	[98]
P1(2019)	PCBM	1:3	−5.15	1.04	0.38	2.16	40	0.33	[99]
P2(2019)^d^	PCBM	1:1	−5.35	1.10	0.52	2.82	50	0.73	[99]

^A^ Weight ratio.

^B^ The HOMO energy level of polymer donors.

^C^ Estimated from the absorption edge in film ($E_{g}^{opt}=1240/\lambda_{onset}$).

^D^ Average *PCE* of OSCs.

^a^ 2.5% DIO.

^b^ 120°C annealing for 10 min.

^c^ SVA treatment.

^d^ 1% DIO.

Kim and co‐workers reported several studies on copolymers based on the main chain BDT‐Qx.^[^ ^90^ ^]^ In 2013, PBDT‐Qx (2013) was prepared by introducing the side chain 3‐(methoxymethyl) heptane into the BDT unit. After applying for OSC devices, the PBDT‐Qx: PC_61_BM‐based OSC obtained the *V_oc_* of 0.65 V, *J_sc_* of 6.30 mA cm^−2^, *FF* of 37% and *PCE* of 1.54%. Furthermore, similar to the PBDT‐Qx, PBDTQx (2012) was synthesized by Wu et al.^[^ ^91^ ^]^ P(BDT‐Qx) (2013) and PBDTFQx (2013) were synthesized by Fu et al.^[^ ^92^ ^]^ and Gao et al.^[^ ^93^ ^]^, respectively. Among these polymer donors, which were synthesized by further introducing side chains 1‐methyl‐3‐(octyloxy) benzene, 1‐methyl‐4‐(octyloxy) benzene as well as 1‐methyl‐3‐(octyloxy) benzene and “F” atom into the Qx unit, respectively. After blending with acceptors, the *V_oc_* in OSCs significantly enhanced, while *J_sc_* and *FF* were not. Interestingly, compared to the PBDTQx: PC_61_BM‐based OSC, P(BDT‐Qx): PC71BM‐based OSC obtained higher *J_sc_* (5.0 mA cm^−2^) and *FF* (50.3%), which can be attributed to the reduced steric hindrance formed by para‐substitution alkoxy side chain than meta‐substitution into the donor unit. Subsequently, based on the thiophene substituted BDT donor unit, Wang et al. prepared the PBDT‐DTQx (2014) by introducing the side chain 2‐hexyl‐5‐methylthiophene into the BDT and Qx units.^[^ ^94^ ^]^ Lee et al. designed PBS‐TAQ (2022) and PBS‐MDTAQ (2022) by introducing the side chain 2‐(heptan‐3‐ylthio)−5‐methylthiophene into the BDT donor as well as 4‐methyl‐*N*, *N*‐diphenylaniline and 4‐methoxy‐*N*‐(4‐methoxyphenyl)‐*N*‐(*p*‐tolyl) aniline into the Qx unit, respectively.^[^ ^95^ ^]^ Notably, the substitution with wide conjugated plane endows such polymer donors: PCBM based OSCs with a high *V_oc_*, meanwhile, the best photoelectric performance presented in PBS‐TAQ: PC_71_BM‐based OSC also reminds us the introduction of alkoxy with electron‐donating into the acceptor unit of polymer donors cannot effectively optimize the *J_sc_* and *FF* in OSCs.

Furthermore, by constructing the polymer backbone (e.g., BDT‐BPz and BDT‐CBPz) with a wider conjugated plane, Kim et al. prepared two polymer donors PBDT‐BPz (2013) and PBDT‐CBPz (2013) through introducing the side chain 3‐(methoxymethyl) heptane into the BDT unit.^[^ ^90^ ^]^ In the same year, Li et al.^[^ ^96^ ^]^ and Kim et al.^[^ ^90^ ^]^ also synthesized PBDT‐DBPz (2013) and PBDT‐DBPz (2013) in turn. Among these donors, due to the formation of broaden conjugated plane, the *V_oc_* in such polymer donor: PC_71_BM‐based OSCs stay at a high level, meanwhile, the better photoelectric performance presented in PBDT‐CBPz: PC_71_BM‐based OSC can be attributed to the more matched effect of electron‐donating and electron‐withdrawing between donor and acceptor units in polymer skeleton of PBDT‐CBPz. Moreover, in order to explore an effective way to accelerate the dissociation of exciton and hole/electron mobility, Yuan and co‐workers constructed the acceptor unit PP with a strong electron‐withdrawing,^[^ ^97^ ^]^ synthesizing P1 (2010) based on the polymer backbone BDT‐PP. In 2019, based on the main‐chain BDT‐TZQx, Keshtov et al. prepared P0F (2018), P2F (2018) and P4F (2018)^[^ ^98^ ^]^; Hacioglu et al. also synthesized P1(2019) and P2(2019).^[^ ^99^ ^]^ After co‐blending with acceptors, P4F: PC_71_BM‐based OSC obtained the *V_oc_* of 0.69 V, *J_sc_* of 15.96 mA cm^−2^, *FF* of 74% and *PCE* of 8.03%, exhibiting the best photoelectric performance. Although the molecular packing and *V_oc_* can be optimized by appropriately extending the conjugated plane of polymer backbone, the electron distribution will be dispersed, resulting in low *J_sc_* and *FF*. Therefore, on the basis of a wide π‐π conjugate plane, the introduction of “F” atoms with electronegative and small atomic radius can effectively optimizes the hole/electron mobility.

Notably, P0F (365°C), P2F (390°C) and P4F (410°C) obtained a high *Td_5%_*, exhibiting an excellent stability. Meanwhile, it is worth noting that compared with PBDT‐Qx: PC_71_BM‐based (8.47 nm), PBDT‐BPz: PC71BM‐based (2.27 nm) and PBDT‐DBPz: PC_71_BM‐based (6.83 nm) blend films, the decreased *RMS* of PBDT‐CBPz: PC_71_BM‐based (1.50 nm) and P(BDT‐Qx): PC_71_BM‐based (1.13 nm) blend films endow corresponding OSCs with the *FF* of 51 and 50.3% in turn, which can be attributed to the formation of a smoother morphology.

**FIGURE 11:**
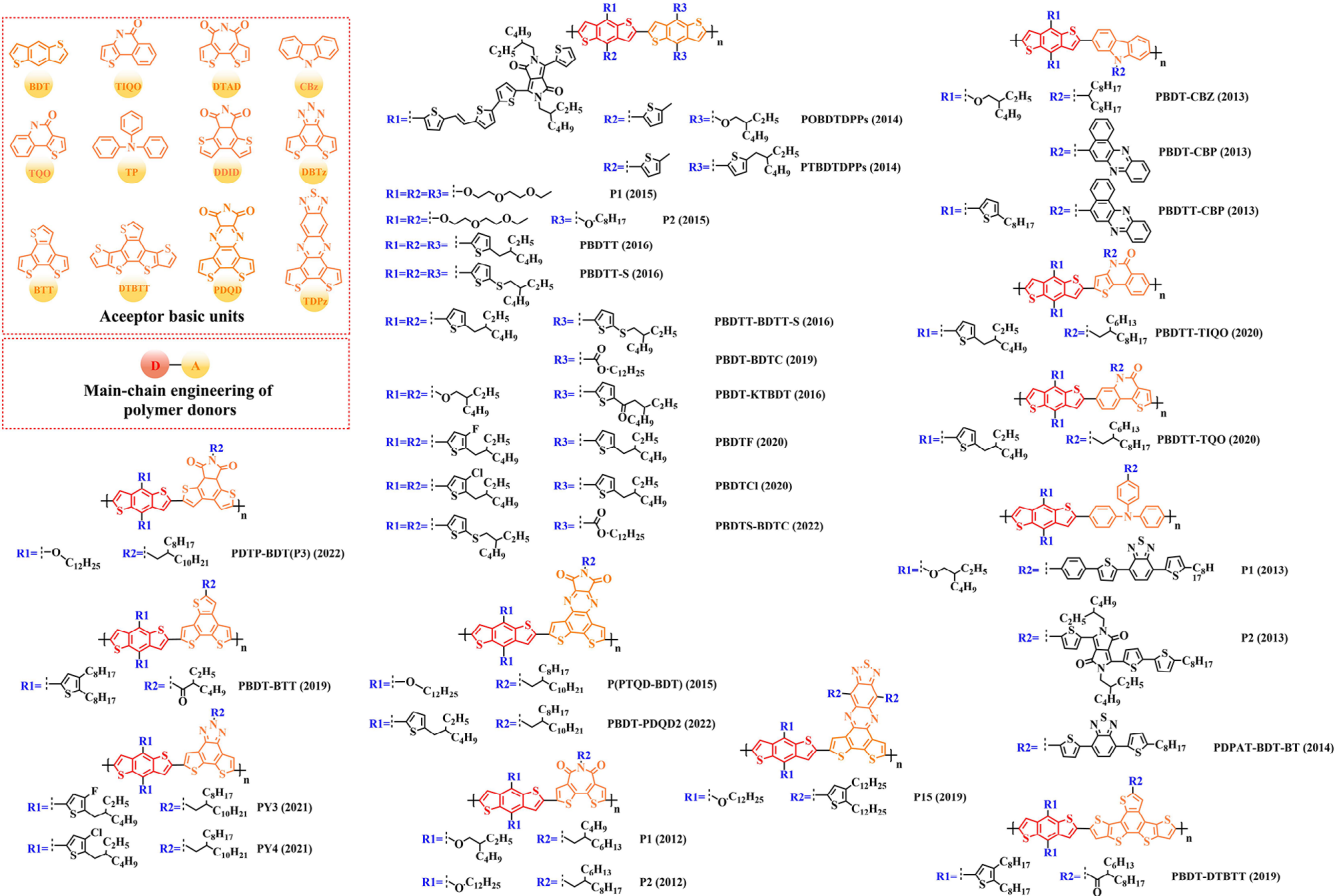
The detailed molecular structure of D‐A type polymer donors synthesized by introducing BDT and its derivatives as acceptor basic units.

Although the linear acceptor units constructed by introducing thiophene, benzene or other planar groups effectively extended the π‐π conjugated plane of main‐chain engineering to a certain extent, the dispersed electron density led to low hole/electron mobility is still a serious problem cannot be ignored. In recent years, compared with linear acceptor units, the acceptor group constructed by meta‐substitution not only ensures the polymer backbone with a larger conjugated plane, but also reduces the torsion angle between the acceptor and donor units. Therefore, many researchers synthesized a series of meta‐substituted acceptor units (e.g., 6b, 9a‐dihydro‐8λ^2^‐dithieno [2, 3‐*e*: 3′, 2′‐*g*] isoindole‐7,9‐dione (DDID), 2λ^2^‐dithieno [3′, 2′: 3, 4; 2″, 3″: 5, 6] benzo [1, 2‐*d*] [1, 2, 3] triazole (DBTz), 9λ^2^‐pyrrolo [3, 4‐*b*] dithieno [3, 2‐*f*: 2′, 3′‐*h*] quinoxaline‐8, 10‐dione (PDQD) and [1, 2, 5] thiadiazolo [3, 4‐*i*] dithieno [3, 2‐*a*: 2′, 3′‐*c*] phenazine (TDPz)). Interestingly, based on such polymer backbone, the photovoltaic performance in PBDTCl: Y6‐based and PBDT‐PDQD2: Y6‐based OSCs have made a great breakthrough, proving the feasibility of constructing these main‐chain engineering. In this chapter, the detailed donor molecular structure (Figure 11) of representative polymer donors and the corresponding photovoltaic parameters (Table 8) in OSC devices were discussed as follow.

**TABLE 8:** Photovoltaic parameters of OSCs related to Figure 11.

Donor	Acceptor	D/A^A^	HOMO [eV]^B^	$E_{g}^{opt}$ [eV]^C^	*V_OC_* [V]	*J_SC_* [mA cm^−2^]	*FF* [%]	*PCE* [%]^D^	Ref.
POBDTTDPPs	PC_71_BM	1:3.5	−5.09	1.71	0.80	8.24	38.87	2.56	[100]
PTBDTTDPPs	PC_71_BM	1:3.5	−5.18	1.73	0.94	7.80	39.82	2.92	[100]
PBDTT	PC_71_BM	1:1	−5.40	2.12	0.92	11.59	42	4.45	[101]
PBDTT‐S	PC_71_BM	1:1	−5.58	2.06	0.96	13.15	49	6.14	[101]
PBDTT‐BDTT‐S	PC_71_BM	1:1	−5.54	2.09	0.99	13.92	51	7.05	[101]
PBDT‐BDTC^a^	ITIC‐Th	1:1	−5.50	1.90	0.92	13.91	65.21	8.03	[102]
PBDTS‐BDTC^a^, ^b^	ITIC‐2F	1:l	−5.52	>1.90	0.895	17.55	69.25	11.01	[103]
PBDTF	Y6		−5.56	2.06	0.82	22.73	67.9	12.23	[104]
PBDTCl	Y6		−5.57	2.07	0.84	23.16	69.2	13.02	[104]
PBDT‐BDT	Y6	1:1	−5.45	2.06	0.80	21.58	71	12.26	[105]
P1(2015)	PCBM	1:1	−5.11	2.19	0.68	2.06	50.55	0.71	[106]
P2(2015)	PCBM	1:1	−5.18	2.21	0.68	4.86	60.46	2.00	[106]
PBDT‐KTBDT	PC_71_BM	1:3	−5.38	2.18	0.73	4.5	45.6	1.50	[107]
P1(2012)^c^	PC_71_BM	1:1.5	−5.58		0.876	9.69	51.8	4.39	[108]
P2(2012)^c^	PC_71_BM	1:1	−5.63		0.922	9.62	62.0	5.50	[108]
PBDT‐CBZ	PC_71_BM	1:3	−5.21	2.41	0.71	1.30	29	0.25	[109]
PBDT‐CBP	PC_71_BM	1:2	−5.50	1.56	0.81	6.97	41	2.33	[109]
PBDTT‐CBP	PC_71_BM	1:3	−5.50	1.55	0.71	4.05	30	0.90	[109]
P1(2013)	PC_71_BM	1:3	−5.57	1.93	0.86	9.40	39	3.09	[110]
P2(2013)	PC_71_BM	1:3	−5.53	1.74	0.80	10.77	35	2.95	[110]
PDPAT‐BDT‐BT^d^	PC_71_BM	1:4	−5.42	1.82	0.87	7.61	49.8	3.27	[111]
PBDTT‐TQO	IT‐4F	1:2	−5.49	2.20	0.90	14.44	58.92	7.38	[112]
PBDTT‐TIQO	ITIC	1:2	−5.40	2.05	0.95	13.33	51.67	6.45	[112]
PBDT‐BTT	PC_71_BM	1:2	−5.43	2.07	0.87	5.03	45.20	1.98	[113]
PBDT‐DTBTT	PC_71_BM	1:2	−5.35	2.04	0.82	6.29	52.45	2.73	[113]
PY3	ITIC‐4Cl		−5.62	2.03	0.798	19.09	73.5	11.20	[114]
PY4	ITIC‐4F		−5.63	2.00	0.874	20.26	70.7	12.51	[114]
PDTP‐BDT(P3)^e^	PThIND‐Cl	1:1.2	−5.46	1.96	0.97	21.48	63	12.93	[115]
P(PTQD‐BDT)	Y6	1:1.2	−5.30	1.84	0.83	22.86	64	11.97	[116]
PBDT‐PDQD2	Y6	1:1.2	−5.38	1.88	0.88	24.68	71	15.24	[116]
P(PTQD‐BDT)^c^	PC_71_BM	1:2			0.88	10.34	61	5.55	[117]
P15(2019)^f^	PC_71_BM	1:1.5	−5.30	1.14	0.90	14.08	64	8.01	[118]

^A^ Weight ratio.

^B^ The HOMO energy level of polymer donors.

^C^ Estimated from the absorption edge in film ($E_{g}^{opt}=1240/\lambda_{onset}$).

^D^ Average *PCE* of OSCs.

^a^ 0.5% DIO.

^b^ o‐xy solvent.

^c^ 3% DIO.

^d^ 1% DIO.

^e^ THF SVA treatment for 40 s.

^f^ SVA treatment.

Based on the main‐chain BDT‐BDT, two novel polymer donors POBDTTDPPs (2014) and PTBDTTDPPs (2014) were reported by Chen and co‐workers.^[^ ^100^ ^]^ Consistent with previous conclusions, the substitution of thiophene‐based side chain with a large conjugated plane endowed polymer donor: PC_71_BM‐based OSCs with a high *V_oc_*, especially for PTBDTTDPPs: PC_71_BM‐based OSC (0.94 V), which can be attributed to the substitution of 2‐(2‐ethylhexyl)−5‐methylthiophene further broaden the conjugated plane. Afterward, Kim et al. synthesized three polymer donors PBDTT (2016), PBDTT‐S (2016) and PBDTT‐BDTT‐S (2016).^[^ ^101^ ^]^ Compared with the side chain 2‐(2‐ethylhexyl)−5‐methylthiophene, 2‐((2‐ethylhexyl) thio)−5‐methylthiophene with a strong electron‐donating further enhances the electron density in polymer backbone, thereby PBDTT‐S: PC_71_BM‐based OSC achieved higher *J_sc_* (13.15 mA cm^−2^) and *FF* (49%) than PBDTT:PC_71_BM‐based OSC. Furthermore, PBDTT‐BDTT‐S with different substitutions also accelerates the hole/electron mobility, thus enhancing the *J_sc_* (13.92 mA cm^−2^) and *FF* (51%) in PBDTT‐BDTT‐S: PC_71_BM‐based OSC. Afterward, based on the reported results of PBDTT and PBDTTT‐S, PBDT‐BDTC (2019) and PBDTS‐BDTC (2022) were synthesized by Hao et al.^[^ ^102^ ^]^ and Li et al.^[^ ^103^ ^]^ in turn, designing by further introducing the side chain dodecyl acetate into the BDT unit. Among them, PBDTS‐BDTC with the substitution of alkylthio endows PBDTS‐BDTC: ITIC‐Th‐based OSC with a higher *J_sc_* (17.55 mA cm^−2^) and *FF* (69.25%) than PBDT‐BDTC:ITIC‐Th‐based OSC. Likewise, Xu et al. synthesized PBDTF (2020) and PBDTCl (2020) by further introducing side chains 2‐(2‐ethylhexyl)−3‐fluoro‐5‐methylthiophene and 3‐chloro‐2‐(2‐ethylhexyl)−5‐methylthiophene into the BDT unit.^[^ ^104^ ^]^ Benefiting the substitution of “F” and “Cl” atoms with strong electron‐withdrawing, which not only decreases the HOMO energy level of polymer donors, but also enhances the hole/electron mobility in OSCs. Compared with PBDTF: Y6‐based OSC (2.71 × 10^−4^/8.79 × 10^−5^ cm^2^ V^−1^ s^−1^), the faster and more balanced hole/electron mobility (1.30 × 10^−4^/2.06 × 10^−5^ cm^2^ V^−1^ s^−1^) endows PBDTCl: Y6‐based OSC with a higher *J_sc_* (23.16 mA cm^−2^) and *FF* (69.2%). As for PBDT‐BDT (2023), which was designed by innovatively introducing side chains 2‐(2‐ethylhexyl)−3‐fluorothiophene and 4‐methoxy‐*N*‐(4‐methoxyphenyl)‐*N*‐phenylaniline into the BDT unit, respectively.^[^ ^105^ ^]^ After applying for OSC devices, the promising *FF* (71%) and efficient *PCE* (12.26%) presented by PBDT‐BDT: Y6‐based OSC verifying the potential development. In addition, based on the alkyloxy substituted BDT unit, Liu and co‐workers prepared P1 (2015) and P2 (2015).^[^ ^106^ ^]^ In the next year, Ha et al. designed PBDT‐KTBDT (2016).^[^ ^107^ ^]^ Among these donors, compared with P1 (2015) and P2 (2015), although the substitution of 3‐ethyl‐1‐(5‐methylthiophen‐2‐yl) heptan‐1‐one extends the π‐π conjugated plane of PBDT‐KTBDT as well as matches with the side chain 3‐(methoxymethyl) heptane to promote the hole/electron mobility, while the *J_sc_* (4.5 mA cm^−2^) and *FF* (45.6%) in PBDT‐KTBDT: PC_71_BM‐based OSC still remain at a low level.

Furthermore, by constructing acceptor units (e.g., 5*H*‐4λ^2^‐thieno [3, 2‐*c*] isoquinolin‐5‐one (DIQO), 5λ^2^‐dithieno [3, 2‐*c*: 2′, 3′‐*e*] azepine‐4, 6‐dione (DTAD) and 9λ^2^‐carbazole (CBz)) containing meta‐substituted π–π conjugated units, a series of novel main‐chain engineering are also emerging. In 2012, Zhou et al. based on the main chain BDT‐DTAD, prepared P1(2012) and P2(2012) by introducing the side chain 7‐ethylpentadecane into the DTAD unit and 3‐(methoxymethyl) heptane and 1‐methoxy‐dodecane into the BDT unit, respectively.^[^ ^108^ ^]^ Consistent with above conclusions, the DTAD unit with a wide conjugated plane endows P1(2012): PC_71_BM‐based and P2(2012): PC_71_BM‐based OSCs with an excellent *V_oc_*. Meanwhile, due to P2(2012) with the introduction of dodecyl substituted alkyloxy side chain, which further enhances the *J_sc_* (9.62 mA cm^−2^) and *FF* (62.0%) in P2(2012): PC_71_BM‐based OSC. In 2013, similar to the substitutions of P1(2012), Kim et al. based on the polymer backbone BDT‐CBz, synthesized PBDT‐CBZ (2013) by introducing side chains 3‐(methoxymethyl) heptane and 9‐methyl‐heptadecane into the BDT and CBz units in turn.^[^ ^109^ ^]^ In addition, by introducing the side chain 5‐methylbenzo [*a*] phenazine into the CBP unit to replace the original substitution in PBDT‐CBZ, PBDT‐CBP (2013) was successfully prepared. As for the PBDTT‐CBP, which was designed by introducing the side chain 2‐methyl‐5‐octylthiophene into the BDT unit to replace the original substitution in PBDTT‐CBP (2013). Compared with PBDT‐CBZ: PC_71_BM‐based and PBDTT‐CBP: PC_71_BM‐based OSCs, PBDT‐CBP: PC_71_BM‐based OSC exhibited a better photoelectric performance, which can be attributed to the more suitable conjugated plane and optimized hole/electron mobility formed by introducing such substitutions. At the same year, based on the 3‐(methoxymethyl) heptane substituted BDT unit, Nie et al. constructed the main chain BDT‐TP, preparing P1(2013) and P2(2013).^[^ ^110^ ^]^ In 2014, Su et al. synthesized PABDT‐BDT‐BT (2014).^[^ ^111^ ^]^ After blending with the acceptor PC_71_BM, P1(2013): PC_71_BM‐based, P2(2013): PC_71_BM‐based and PABDT‐BDT‐BT: PC_71_BM‐based OSCs obtained the *V_oc_* of 0.86, 0.80 and 0.87 V; *J_sc_* of 9.40, 10.77 and 7.61 mA cm^−2^; *FF* of 39%, 35% and 49.8%; and *PCE* of 3.09%, 2.95% and 3.27%, respectively. Compared with P1(2013) and P2(2013), the smaller conjugated plane of PDPAT‐BDT‐BT endows PDPAT‐BDT‐BT: PC_71_BM‐based OSC with a better photoelectric performance, which further verifying that appropriated conjugated plane as well as hole/electron mobility of donor materials are critical factors for OSC devices to obtain an efficient *PCE*. Afterward, Ha et al. based on the polymer skeleton BDT‐TIQO and BDT‐TQO, prepared PBDT‐TIQO (2020) and PBDT‐TQO (2020) by introducing side chains 2‐(2‐ethylhexyl)−5‐methylthiophene and 7‐ethylpentadecane into the BDT and TIQO units in subsequence.^[^ ^112^ ^]^ After blending with acceptors, PBDTT‐TIQO: ITIC‐based and PBDTT‐TQO: IT‐4F‐based OSCs achieved the *V_oc_* of 0.95 and 0.90 V, *J_sc_* of 13.33 and 14.44 mA cm^−2^, *FF* of 51.67% and 58.92%, and *PCE* of 6.45% and 7.38%, respectively. Indeed, the photovoltaic performance of OSCs fabricated based on corresponding polymer donors can be optimized by adjusting the orientation direction of asymmetric acceptor units in polymer backbone.

Afterward, by further extending the conjugated plane of acceptor unit as well as adjusting the hole/electron mobility in OSC devices, acceptor units compose with four, five or even more units (e.g., benzo [1, 2‐*b*: “3, 4‐b’: 6, 5‐b”] trithiophene (BTT), [1, 2, 5] thiadiazolo [3, 4‐*i*] dithieno [3, 2‐a: 2′, 3′‐*c*] phenazine (TDPz) and 2λ^2^‐dithieno [3′, 2′: 3, 4; 2″, 3″: 5, 6] benzo [1, 2‐d] [1, 2, 3] triazole (DBTz)) have also emerged in recent years. Based on the main chain BDT‐BTT, PBDT‐BTT (2019) was reported by Zhang et al.^[^ ^113^ ^]^ In 2021, Zhao et al. designed PY3 (2021) and PY4 (2021) based on the main chain BDT‐DTBz.^[^ ^114^ ^]^ In 2022, Keshtov et al. based on the polymer skeleton BDT‐DDID, constructed PDTP‐BDT(P3) (2022).^[^ ^115^ ^]^ After applying these donors into the OSC devices, PBDT‐BTT: PC_71_BM‐based, PY3: ITIC‐4Cl‐based, PY4: ITIC‐4Cl‐based and PDTP‐BDT(P3): PThIND‐Cl‐based OSCs obtained the *V_oc_* of 0.87, 0.798, 0.874 and 0.97 V; *J_sc_* of 5.03, 19.09, 20.26 and 21.48 mA cm^−2^; *FF* of 45.2%, 73.5%, 70.7% and 63%; and *PCE* of 1.98%, 11.20%, 12.51% and 12.93%, respectively. Although these polymer skeleton with analogously molecular structure, “N” atoms and aldehyde group with electron‐withdrawing increase the electronegative of DDID and DTBz units, thus the fast hole/electron mobility endows PY3: ITIC‐4Cl‐based, PY4: ITIC‐4Cl‐based and PDTP‐BDT(P3): PThIND‐Cl‐based OSCs with a high *J_sc_* and *FF*. Furthermore, based the reported results of PBDT‐BTT, Zhang et al. synthesized PBDT‐DTBTT (2019) by further introducing two thiophene groups into the BTT unit.^[^ ^113^ ^]^ Notably, compared with the PBDT‐BTT: PC_71_BM‐based OSC, the *V_oc_* (0.82 V) in PBDT‐DTBTT: PC_71_BM‐based OSC still retain at a high level, meanwhile, the *J_sc_* (6.29 mA cm^−2^) and *FF* (52.45%) also slightly improved. Similarly, based on the PDTP‐BDT(P3), Keshtov et al. constructed the novel acceptor unit 9λ^2^‐pyrrolo [3, 4‐*b*] dithieno [3, 2‐*f*: 2′, 3′‐*h*] quinoxaline‐8, 10‐dione (PDQD) with a stronger electronegative by further introducing the pyrazine group into the DDID unit.^[^ ^117^, ^118^ ^]^ Afterward, two polymer donors P(PTQD‐BDT) (2015) and PBDT‐PDQD2 (2019) were successfully synthesized based on the main chain BDT‐PDQD. In addition, Keshtov and co‐workers also prepared P15 (2019) based on the polymer backbone BDT‐TDPz by introducing side chain 1‐methoxydodecane into the BDT as well as 2,3‐didodecyl‐5‐methylthiophene into the TDPz units in turn.^[^ ^118^ ^]^ After applying for OSC devices, P(PTQD‐BDT): Y6‐based, PBDT‐PDQD2: Y6‐based and P15: PC_71_BM‐based OSCs obtained the *V_oc_* of 0.83, 0.88 and 0.90 V; *J_sc_* of 22.86, 24.68 and 14.08 mA cm^−2^; *FF* of 64%, 71% and 64%; and *PCE* of 11.97%, 15.24% and 8.01%, respectively. Compared with PDTP‐BDT(P3): PThIND‐Cl‐based OSC, the wider conjugated plane and stronger molecular polarity of PBDT‐PDQD2 endows PBDT‐PDQD2: Y6‐based OSC with a high *V_oc_*, *J_sc_* and *FF*, which once again verified that suitable π–π conjugated structure and fast hole/electron mobility are critical factors for donor materials to optimize the photovoltaic performance in polymer donor‐based OSCs.

In addition, based on the characterization of blend film morphology and device stability, PBDTT (436°C), PBDTT‐S (401°C), PBDTT‐BDTT‐S (410°C), PBDT‐BDTC (370°C), PBDTS‐BDTC (350°C), P1(2015) (318°C), P2(2015) (316°C), PBDT‐KTBDT (351°C), PY3 (409.7°C) and PY4 (431.2°C) obtained a high *Td_5%_*, exhibiting excellent stability. Meanwhile, the perfect device stability (*PCE* drops ≈5.5% after 16 days) presented by P2 (2015): PCBM‐based OSC also exhibiting a promising application. Furthermore, compared with PBDTT: PC_71_BM‐based blend film (4.04 nm), the significantly decreased *RMS* of PBDTT‐S: PC_71_BM‐based blend film (1.66 nm) endows corresponding OSC with a higher *FF*, which can be attributed to the accelerated hole/electron mobility induced by the formation of more suitable phase separation morphology. Interestingly, between the morphology characterization of P1(2015): PCBM‐based (5.01 nm) and P2(2015): PCBM‐based (2.54 nm) blend films, P1(2012): PC_71_BM‐based (5.47 nm) and P2(2012): PC_71_BM‐based (2.15 nm) blend films as well as PBDT‐CBP: PC_71_BM‐based (3.08 nm) and PBDTT‐CBP: PC_71_BM‐based (6.21 nm) blend films, the relatively decreased *RMS* is conducive to endow corresponding OSCs with a higher *FF*, which has further proved the above‐conclusion.

#### 3.1.2 D‐A‐A, A‐D‐A, D‐D‐A, D‐D‐D‐A, or D‐A‐A‐A type polymer donors

**FIGURE 12:**
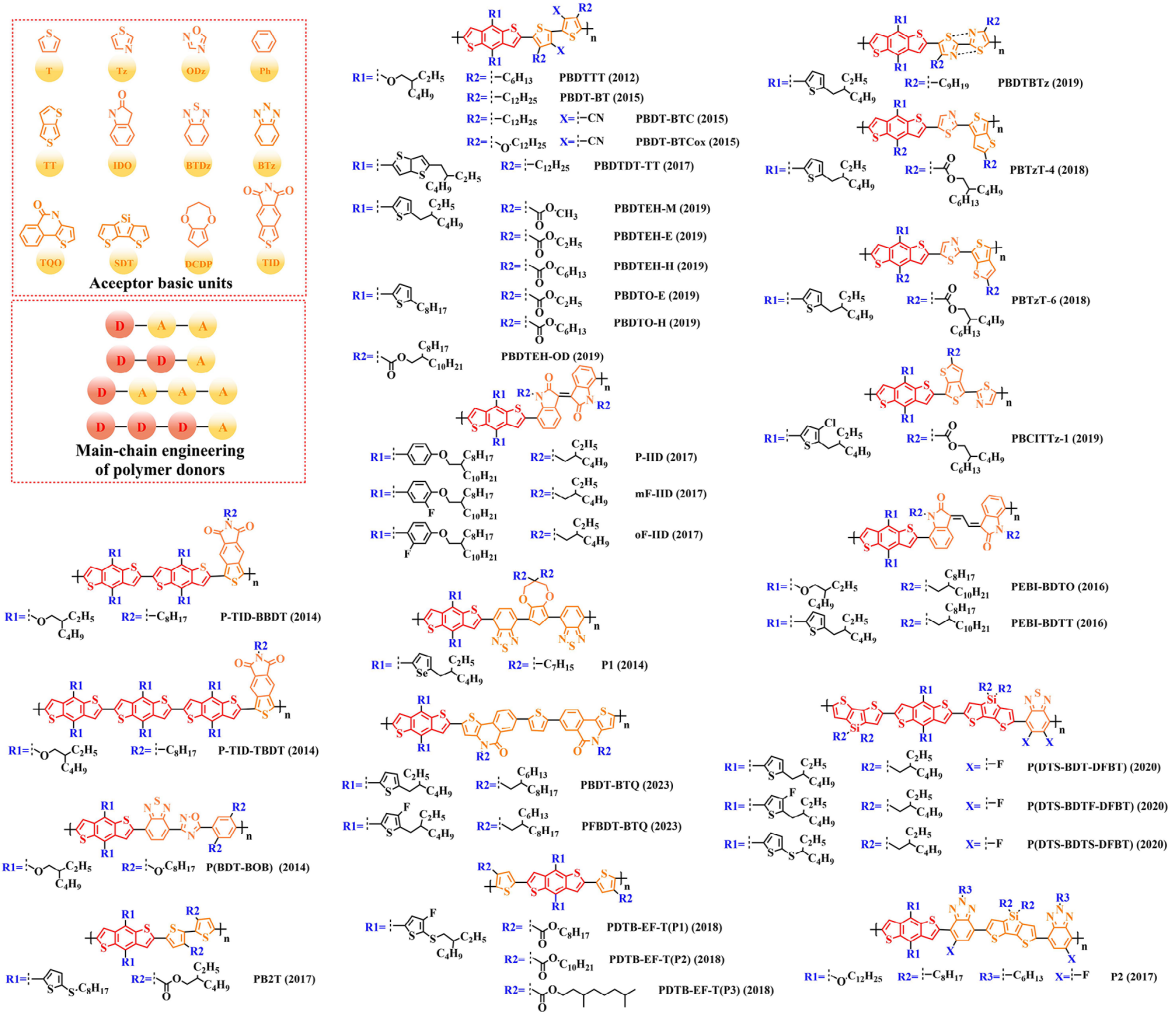
The detailed molecular structure of D‐A‐A, A‐D‐A, D‐D‐A, D‐D‐D‐A, or D‐A‐A‐A type polymer donors.

With the deepening of the research field of OSC, D‐A type polymer donor, as the oldest type of polymer donor, usually maintain a high *V_oc_* in OSC devices, while the problem of low *J_sc_* and *FF* seriously hinders the further breakthrough of *PCE*. Therefore, in order to solve this problem, the plenty of researches have begun to focus on the molecular design strategy of further introducing donor or acceptor units into the main‐chain engineering, thereby further enhancing the dissociation of excitons and hole/electron mobility. In this chapter, the detailed molecular structure (Figure 12) of representative polymer donors and photoelectric parameters (Table 9) in corresponding OSC devices are discussed as follows.

**TABLE 9:** Photovoltaic parameters of OSCs related to Figure 12.

Donor	Acceptor	D/A^A^	HOMO [eV]^B^	$E_{g}^{opt}$ [eV]^C^	*V_OC_* [V]	*J_SC_* [mA cm^−2^]	*FF* [%]	*PCE* [%]^D^	Ref.
PBClTTz‐1^a^	PC_71_BM	1:1.5	−5.57	1.70	0.94	14.09	63.57	8.02	[53]
P1^b^	PC_71_BM	1:2	−5.34	1.64	0.65	11.72	58.69	4.51	[72]
P‐TID‐BBDT^c^	PC_61_BM	1:1		1.50	0.70	4.7	28	0.9	[78]
P‐TID‐TBDT	PC_61_BM	1:1		1.60	0.78	3.5	42	1.2	[78]
PBDTTT	PCBM	1:3	−5.32	1.96	0.72	1.61	31	0.36	[119]
PBDT‐BT	PC_71_BM		−5.37	2.10	0.82	5.86	45.8	2.01	[120]
PBDT‐BTC^d^	PC_71_BM		−5.58	1.96	0.98	1.27	41.4	0.52	[120]
PBDT‐BTcox^d^	PC_71_BM		−5.50	1.74	0.86	10.1	58.4	4.90	[120]
PBDTDT‐TT	ITM	1:1	−5.27	1.8	0.82	16.17	63.40	8.43	[121]
PBDTEH‐M^e^	ITIC‐Th	1:1	−5.37	1.96	0.88	14.03	50.4	6.03	[122]
PBDTEH‐E^e,f^	ITIC‐Th	1:1	−5.43	1.98	0.92	14.29	58.2	7.48	[122]
PBDTEH‐H^f,g^	ITIC‐Th	1:1	−5.47	2.00	0.99	14.37	43.6	6.09	[122]
PBDTO‐E	ITIC‐Th	1:1	−5.32	2.02				(‐)	[122]
PBDTD‐H^f,g^	ITIC‐Th	1:1	−5.45	1.98	0.94	14.82	59.4	8.10	[122]
PBDT‐OD^g^	ITIC‐Th	1:1	−5.52	2.10	1.04	8.94	43.2	3.90	[122]
PB2T^h^	IT‐M	1:1	−5.63	2.24	0.56	0.06	26	0.01	[123]
PDTB‐EF‐T(P1)	IT‐4F	1:1	−5.51	1.93	0.892	20.13	61	11.0	[124]
PDTB‐EF‐T(P2)	IT‐4F	1:1	−5.50	1.93	0.897	20.50	70	12.9	[124]
PDTB‐EF‐T(P3)	IT‐4F	1:1	−5.54	1.94	0.904	19.97	61	10.8	[124]
PEBI‐BDTO	PC_71_BM	1:2	−5.46	1.65	0.86	6.53	53.4	3.00	[125]
PEBI‐BDTT	PC_71_BM	1:1.5	−5.51	1.67	0.94	7.88	62.1	4.59	[125]
P‐IID	PC_71_BM	1:2	−5.35	1.63	0.84	9.68	64.3	5.23	[126]
mF‐IID	PC_71_BM	1:1	−5.48	1.67	0.88	5.61	50.5	2.50	[126]
oF‐IID	PC_71_BM	1:2	−5.53	1.71	0.96	1.61	60.2	0.93	[126]
PBTzT‐4^i^	PC_71_BM	1:1.5	−5.35	1.65	0.78	16.48	69.7	9.19	[127]
PBTzT‐6^a^	PC_71_BM	1:1.5	−5.31	1.66	0.77	15.45	69.1	8.28	[127]
PBDTBTz^j^	IT‐4F	1:1	−5.60	1.99	0.89	13.35	64.80	7.74	[128]
P(BDT‐BOB)^k^	PC_61_BM	1:2	−5.27	1.99	0.70	11.23	53.82	4.21	[129]
P2(2017)^l^	PC_71_BM	1:2	−5.21	1.76	0.85	14.08	68	8.06	[130]
P(DTS‐BDT‐DFBT)^m^	IEICO‐4F		−5.06		0.71	22.7	62.5	9.7	[131]
P(DTS‐BDTS‐DFBT)^m^	IEICO‐4F		−5.21		0.80	16.4	54.7	6.3	[131]
P(DTS‐BDTF‐DFBT)^m^	IEICO‐4F		−5.11		0.81	12.9	55.2	5.3	[131]
PBDT‐BTQ^n^	IT‐4F	1:1.5	−5.45	2.12	0.86	18.27	54.25	8.50	[132]
PFBDT‐BTQ^o^	IT‐4F	1:1.5	−5.58	2.16	0.90	17.01	55.66	8.55	[132]

^A^ Weight ratio.

^B^ The HOMO energy level of polymer donors.

^C^ Estimated from the absorption edge in film ($E_{g}^{opt}=1240/\lambda_{onset}$).

^D^ Average *PCE* of OSCs.

^a^ 2% DIO.

^b^ 1% DIO.

^c^ 4% DIO.

^d^ 1.5% DIO.

^e^ 150°C annealing.

^f^ 0.5% DIO.

^g^ 100°C annealing.

^h^ 150°C annealing for 10 min.

^i^ 1% DIO.

^j^ 0.25% DIO.

^k^ isopropyl alcohol (IPA).

^l^ DIO.

^m^ 1% CN.

^n^ 1.5% DPE and 130°C annealing for 10 min.

^o^ 0.5% DIO and 170°C annealing for 10 min.

Based on the main‐chain engineering BDT‐T‐T, Yang et al. synthesized PBDTTT (2012) by introducing side chains 3‐(methoxymethyl) heptane into the BDT unit as well as hexyl into the thiophene unit.^[^ ^119^ ^]^ After blending with the acceptor PCBM. PBDTTT: PCBM based OSC obtained the *V_oc_* of 0.72 V, *J_sc_* of 1.61 mA cm^−2^, *FF* of 31% and *PCE* of 0.36%. Subsequently, based on the reported results of PBDTTT, PBDT‐BT (2015), PBDT‐BTC (2015) and PBDT‐BTCox (2015) were reported by Kim and co‐workers.^[^ ^120^ ^]^ Notably, after applying for OSC devices, these polymer donors: PC_71_BM‐based OSCs presented a significantly enhanced *V_oc_* than PBDTTT: PCBM‐based OSC, which can be attributed to the optimized miscibility of donor in active layer induced by introducing long alkyl side chain. Meanwhile, cyano (CN) group with a strong electron‐withdrawing also effectively decreased the HOMO energy level of donor. Furthermore, benefiting the side chain 1‐methoxydodecane with electron‐donating matched with “CN” group in PBDT‐BTCox, thus synergistically promoting the electron/hole mobility in the active layer, endowing PBDT‐BTCox: PC_71_BM‐based OSC with a higher *J_sc_* (10.1 mA cm^−2^) and *FF* (58.4%) than other types of OSC devices. In 2017, based on the reported results of PBDT‐BT, Zhang and co‐workers synthesized PBDTDT‐TT (2017) via introducing the side chain 2‐(2‐ethylhexyl)−5‐methyl‐3*a*, 6*a*‐dihydrothieno [3, 2‐*b*] thiophene to replace the original substitution in BDT unit.^[^ ^121^ ^]^ Compared with PBDT‐BT: PC_71_BM‐based OSC, PBDTDT‐TT: ITM‐based OSC presented a significantly improved *J_sc_* (16.17 mA cm^−2^) and *FF* (63.40%), which can be attributed to the more complemental absorption achieved in active layer. Afterward, a series of polymer donors PBDTEH‐M (2019), PBDTEH‐E (2019), PBDTEH‐H (2019), PBDTO‐E (2019) and PBDTO‐H (2019) with the substitution of alkyl‐thiophene and carboxylate were also reported by Wang et al.^[^ ^122^ ^]^ After blending with the acceptor PC_61_BM, due to the side chain alkyl‐thiophene broaden the π–π conjugated plane of such polymer donors, the optimized molecular stacking in active layer endows these polymer donors: PC_61_BM‐based OSC with a high *V_oc_*. At the same time, the substitution of carboxylate with a strong electronegative further accelerates hole/electron mobility, thereby an optimized *J_sc_* was achieved in OSC achieves. Furthermore, the introduction of suitable alkyl chain also further enhances the miscibility of PBDTD‐H, which endows PBDTD‐H: ITIC‐Th‐based OSC with a more ideal photoelectric performance. In addition, to explore the effect of introducing carboxylate side chain into the polymer donors on photovoltaic performance of corresponding OSCs, Liu et al. reported a novel polymer donor PB2T (2017), which was synthesized by introducing side chains 2‐methyl‐5‐(octylthio) thiophene and 2‐ethylhexyl acetate into the BDT and thiophene units in turn;^[^ ^123^ ^]^ Wang and co‐workers also prepared PBDTEH‐OD (2019) by introducing the side chain 2‐octyldodecyl acetate into the BDT and thiophene units.^[^ ^122^ ^]^ After applying for OSC devices, PB2T: IT‐M‐based and PBDTEH‐OD: ITIC‐Th‐based OSCs obtained the *V_oc_* of 0.56 and 1.04 V, *J_sc_* of 0.06 and 8.94 mA cm^−2^, *FF* of 26% and 43.2%, and *PCE* of 0.01% and 3.90%, respectively. Although the introduction of alkylthio significantly decreased the HOMO energy level of polymer donors, selecting alkylthio substituted thiophene as acceptor unit do not optimize the hole/electron mobility and finally the low *J_sc_* and *FF* hinder the improvement of *PCE*. In addition, by transforming the main chain BDT‐T‐T into a T‐BDT‐T, Li et al. prepared PDTB‐EF‐T(P1) (2018), PDTB‐EF‐T(P2) (2018) and PDTB‐EF‐T(P3) (2018) by introducing the side chain 2‐((2‐ethylhexyl) thio)−3‐fluoro‐5‐methylthiophene into the BDT unit as well as octyl acetate, decyl acetate, and 3,7‐dimethyloctyl acetate into the thiophene unit, in that order.^[^ ^124^ ^]^ After blending with acceptors, P1: IT‐4F‐based, P2: IT‐4F‐based and P3: IT‐4F‐based OSCs achieved the *V_oc_* of 0.892, 0.897 and 0.904 V; *J_sc_* of 20.13, 20.50 and 19.97 mA cm^−2^; *FF* of 61%, 70% and 61%; and *PCE* of 11.0%, 12.9%, 10.8%, respectively. Among them, by introducing side chains alkylthio and linear alkyl chain both conducive to enhance the π–π interaction between polymer chains, thereby forming a more ideal blend film morphology. Therefore, P2 with the substitution of n‐decyl, presents a significantly red‐shift trend compared with the P1 and P3 under the UV–vis characterization after blending with the acceptor IT‐4F, which also endows P2: IT‐4F‐based OSC with higher *J_sc_* (20.50 mA cm^−2^) and *FF* (70%).

Subsequently, based on the molecular design strategy of D‐A‐A type main‐chain engineering, a series of novel polymer donors were prepared. In 2016, based on the main chain BDT‐IDO‐IDO, PEBI‐BDTO (2016) and PEBI‐BDTT (2016) were reported by Wu and co‐workers,^[^ ^125^ ^]^ synthesizing by introducing the side chain 9‐ethylnonadecane into the 1λ^2^‐indolin‐2‐one (IDO) unit as well as 3‐(methoxymethyl) heptane and 2‐(2‐ethylhexyl)−5‐methylthiophene into the BDT unit in turn; Cong and co‐workers^[^ ^126^ ^]^ reduced the length between the IDO units in original polymer backbone BDT‐IDO‐IDO, synthesizing P‐IID (2017), mF‐IID (2017) and oF‐IID (2017) by introducing the side chain 3‐ethylheptane into the IDO unit as well as 1‐methyl‐4‐((2‐octyldodecyl) oxy) benzene, 2‐fluoro‐4‐methyl‐1‐((2‐octyldodecyl) oxy) benzene, and 2‐fluoro‐1‐methyl‐4‐((2‐octyldodecyl) oxy) benzene into the BDT unit, respectively; Zhu et al. based on the polymer backbone BDT‐Tz‐TT, synthesized two polymer donors PBTzT‐4 (2018) and PBTzT‐6 (2018) with different orientation directions of TT unit along the main chain,^[^ ^127^ ^]^ designing by introducing side chains 2‐(2‐ethylhexyl)−5‐methylthiophene and 2‐butyloctyl acetate into the BDT and TT units in subsequence; Huang et al. based on the main‐chain BDT‐Tz‐Tz, reported PBDTBTz (2019) by introducing side chains 2‐(2‐ethylhexyl)−5‐methylthiophene and heptyl into the BDT and Tz units in turn^[^ ^128^ ^]^; Yuan and co‐workers based on the polymer backbone BDT‐TT‐Tz, reported PBCITTZ‐1 (2019) by introducing side chains 3‐chloro‐2‐(2‐ethylhexyl)−5‐methylthiophene and 2‐butyloctyl acetate into the BDT and TT units, in that order.^[^ ^53^ ^]^ Among these donors, benefiting thiazole unit effectively decreased the dihedral angle between adjacent units in polymer skeleton,^[^ ^127^ ^]^ meanwhile, the introduction of thiophene‐based or benzene‐based side chains with a wide conjugated plane also significantly broaden the π–π conjugated plane of polymer donors, thereby the *V_oc_* in corresponding OSCs retain at a high level. Furthermore, selecting alkylthio substituted TT group as acceptor unit also effectively accelerate hole/electron mobility in the active layer. Therefore, PBTzT‐4: PC_71_BM‐based, PBTzT‐6: PC_71_BM‐based and PBCITTz: IT‐4F‐based OSCs achieve a higher *J_sc_* and *FF* than others types of OSCs. Moreover, compared with PEBI‐BDTO and PEBI‐BDTT, the substitution of alkyloxy‐benzene and shortened polymer backbone in P‐IID not only enhance the electron‐donating of BDT unit, but also promote the hole/electron mobility in the active layer. Finally, P‐IID: PC_71_BM‐based OSC obtained an optimized *J_sc_* and *FF*. However, the introduction of “F” atom weakens the electron‐donating of alkyloxy‐benzene substituted BDT unit, which explains the reason why the *J_sc_* and *FF* in mF‐IID: PC_71_BM‐based and oF‐IID: PC_71_BM‐based OSCs are low.

Moreover, to further explore the molecular design strategy of D‐A‐A, D‐A‐A‐A, or D‐D‐D‐A type main‐chain engineering. P(BDT‐BOB) (2014), based on the polymer backbone BDT‐BTDz‐ODz‐Ph, was reported by Agneeswari et al.^[^ ^129^ ^]^ In the same year, Braunecker et al. based on the D‐D‐A type main chain BDT‐BDT‐TID and D‐D‐D‐A type main chain BDT‐BDT‐BDT‐TID, prepared P‐TID‐BBDT (2014) and P‐TID‐TBDT (2014) in turn.^[^ ^78^ ^]^ In addition, Chakravarthi et al. also synthesized P1 (2014) based on the polymer skeleton BDT‐BDTz‐DDCP‐BDTz;^[^ ^72^ ^]^ P2 (2017) was synthesized based on the main chain BDT‐BTz‐SDT‐BTz^[^ ^130^ ^]^; P(DTS‐BDT‐DFBT) (2020), P(DTS‐BDTF‐DFBT) (2020) and P(DTS‐BDTS‐DFBT) (2020) were reported by Paleti et al.^[^ ^131^ ^]^ designing based on the polymer backbone SDT‐BDT‐SDT‐BTDz. In recent year, PBDT‐BTQ (2023) and PFBDT‐BTQ (2023) were also synthesized based on the polymer skeleton BDT‐TQO‐T‐TQO.^[^ ^132^ ^]^ After applying for OSC devices, the promising *V_oc_* presented in P2: PC_71_BM‐based, PBDT‐BTQ: IT‐4F‐based and PFBDT‐BTQ: IT4F‐based OSCs were also verifying the potential application of D‐A‐A‐A type main‐chain engineering. Indeed, although the planar conjugated structure can be maintained after adjusting the ratio of donor and acceptor units, the large twist dihedral angle between adjacent units is not conducive to close molecular stacking within the active layer. As a result, the *V_oc_* in corresponding OSC devices is often reduced. Meanwhile, introducing functional side chains with electron‐donating or electron‐withdrawing is also affected by the large conjugated plane, the dispersed electron density also leads to limited incensement of *J_sc_* and *FF*.

Among these donors, PBDTEH‐M (460°C), PBDTEH‐E (420°C), PBDTO‐E (430°C), and PBDTD‐H (420°C) obtained a high *Td_5%_*, exhibiting an excellent stability. Furthermore, among PBDTEH‐M: ITIC‐Th‐based (0.972 nm), PBDTEH‐E: ITIC‐Th‐based (0.840 nm) and PBDTEH‐H: ITIC‐Th‐based (0.766 nm) blend films, the suitable *RMS* endows PBDTEH‐E: ITIC‐Th‐based OSC with a high *FF* (58.2%). Interestingly, based on the morphology characterization of PDTB‐EF‐T(P1): IT‐4F‐based (2.06 nm), PDTB‐EF‐T(P2): IT‐4F‐based (1.70 nm) and PDTB‐EF‐T(P3): IT‐4F‐based (1.82 nm) blend films as well as P(DTS‐BDT‐DFBT): IEICO‐4F‐based (2.78 nm), P(DTS‐BDTS‐DFBT): IEICO‐4F‐based (2.64 nm) and P(DTS‐BDTF‐DFBT): IEICO‐4F‐based (2.62 nm) blend films, the most suitable *RMS* also endows PDTB‐EF‐T(P2): IT‐4F‐based (70%) and P(DTS‐BDT‐DFBT): IEICO‐4F‐based (62.5%) OSCs with a high *FF*, which further proves the above‐conclusion. As for PBDTD‐H: ITIC‐Th‐based (1.06 nm) and PBDT‐OD: ITIC‐Th‐based (2.54 nm) blend films, the decreased *RMS* effectively accelerating exciton dissociation and hole/electron mobility, thereby PBDTD‐H: ITIC‐Th‐based OSC achieves a higher *FF* (59.4%).

#### 3.1.3 D‐π‐A‐π type polymer donors

In order to further optimizing the photoelectric performance of D‐A type polymer donors, synthesizing D‐π‐A‐π type polymer donors by introducing π‐bridge units into the main‐chain engineering has made a great breakthrough in recent years. Among them, D‐π‐A‐π type polymer backbone as the most popular main‐chain engineering, has spawned a series of efficient polymer donors, such as PM6,^[^ ^3^, ^4^ ^]^ D18,^[^ ^9^ ^]^ D18‐Cl^[^ ^133^ ^]^ etc.

**FIGURE 13:**
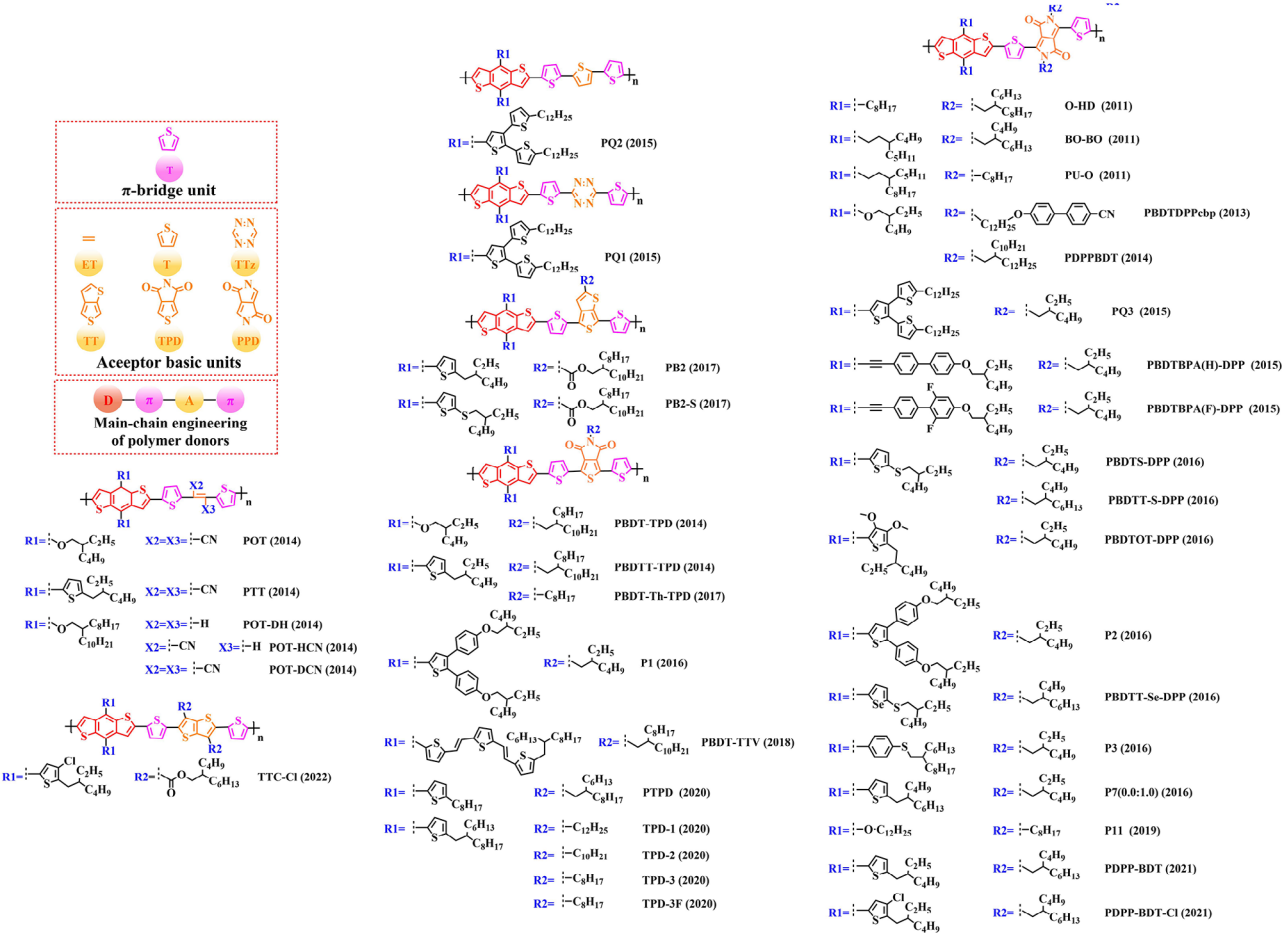
The detailed molecular structure of D‐π‐A‐π type polymer donors synthesized by introducing thiophene as π‐bridge unit, ET, T, TTz, TT, TPD, or PPD as acceptor basic units.

Thiophene is a planar unit with electron‐donating, as π‐bridge units into the main‐chain engineering can not only extends the π–π conjugated plane, but also promotes the hole/electron mobility. Therefore, to explore the potential effect of constructing main‐chain engineering with thiophene π‐bridge unit for polymer donors, the detailed molecular structure (Figure 13) of representative polymer donors and photovoltaic parameters (Table 10) in corresponding OSC devices.

**TABLE 10:** Photovoltaic parameters of OSCs related to Figure 13.

Donor	Acceptor	D/A^A^	HOMO [eV] ^B^	$E_{g}^{opt}$ [eV]^C^	*V_OC_* [V]	*J_SC_* [mA cm^−2^]	*FF* [%]	*PCE* [%]^D^	Ref.
PBDTS‐DPP	PC_70_BM	1:2	−5.30	1.45	0.72	6.72	37.69	1.82	[73]
P11(2019)^a^	PC_71_BM	1:1.5	−5.23	1.34	0.83	13.88	64	7.29	[118]
POT	PC_60_BM	1:1.5	−5.45	1.60	0.795	0.138	28.83	0.03	[134]
PTT	PC_60_BM	1:1.5	−5.37	1.48	0.750	0.150	25.65	0.03	[134]
POT‐DH	PC_71_BM	1:1.5	−5.32	1.99	0.66	9.36	60.3	3.73	[135]
POT‐HCN	PC_71_BM	1:1.5	−5.54	1.74	0.79	9.20	57.9	4.21	[135]
POT‐DCN	PC_71_BM	1:1.5	−5.59	1.60	0.77	0.14	28.8	0.03	[135]
PQ1	PC_61_BM	1:3	−5.61	1.96	1.01	2.12	50.68	1.08	[136]
PQ2^b^	PC_71_BM	1:1.5	−5.28	1.97	0.80	7.68	62.05	3.80	[136]
PQ3^b^	PC_71_BM	1:2	−5.31	1.29	0.78	2.67	52.36	1.09	[136]
PB2	ITIC	1:1	−4.94	1.63	0.628	12.41	47.8	3.72	[137]
PB2‐S	ITIC	1:1	−5.08	1.64	0.730	14.61	50.1	5.35	[137]
TTC‐Cl	HCl‐BTA3		−5.43		1.20	12.84	69.6	11.04	[138]
PBDT‐TPD	PC_71_BM	1:1	−5.32	1.91	0.85	6.97	68	3.89	[139]
PBDTT‐TPD	PC_71_BM	1:1	−5.34	1.88	0.85	7.09	73	4.32	[139]
P1(2016)	PC_71_BM	1:3	−5.50	1.86	0.99	4.45	46.59	2.05	[140]
P2(2016)	PC_71_BM	1:2	−5.35	1.35	0.78	2.91	41.75	0.96	[140]
PBDT‐Th‐TPD	PC_71_BM	1:1.5	−5.41	2.17	0.74	4.17	36.08	1.11	[141]
PBDT‐TTV	PC_70_BM	1:2	−5.61		0.85	10.0	56	4.72	[142]
PTPD^c^, ^d^	Y6	1:1.2	−5.05	1.86	0.66	19.5	46	5.5	[143]
TPD‐1^c^	IT‐4F	1:1	−5.48	1.87	0.81	19.4	74.7	11.2	[144]
TPD‐2^c^	IT‐4F	1:1	−5.45	1.88	0.81	19.6	74.2	11.5	[144]
TPD‐3^c^	IT‐4F	1:1	−5.49	1.90	0.80	20.1	75.3	11.9	[144]
TPD‐3F^c^	IT‐4F	1:1	−5.62	1.90	0.91	20.5	73.8	13.6	[144]
O‐HD^e^	PC_71_BM		−5.15	1.45	0.71	9.4	61	4.1	[145]
BO‐BO^e^	PC_71_BM		−5.14	1.51	0.59	3.4	46	0.93	[145]
PU‐O^e^	PC_71_BM		−5.10	1.36	0.62	5.2	43	1.4	[145]
PBDTDPPcbp^f^	PCBM	1:2	−5.39		0.68	4.11	44.8	1.2	[146]
PDPPBDT	PC_71_BM	1:2	−5.37	1.81	0.742	7.12	62	3.28	[147]
PBDTBPA(H)‐DPP^g^	PC_71_BM	1:1.5	−5.25	1.39	0.73	12.36	51.21	4.62	[148]
PBDTBPA(F)‐DPP^g^	PC_71_BM	1:1.5	−5.20	1.29	0.68	9.49	46.99	3.04	[148]
PBDTOT‐DPP	PC_70_BM	1:2	−5.20	1.33	0.78	8.29	47.2	3.05	[149]
PBDTT‐S‐DPP^h^	PC_71_BM		−5.41	1.48	0.76	10.97	53.54	4.49	[150]
PBDTSe‐S‐DPP^h^	PC_71_BM		−5.29	1.48	0.76	11.34	57.87	5.01	[150]
P3	PC_71_BM	1:2	−5.50	1.44	0.74	4.55	56.21	1.90	[151]
P7(0.0:1.0)	PC_71_BM		−5.20	1.43	0.71	9.93	59.1	4.02	[152]
PDPP‐BDT	PC_71_BM	1:2	−5.16		0.74	11.75	53	4.53	[153]
PDPP‐BDT‐Cl	PC_71_BM	1:2	−5.34		0.86	10.01	60	5.02	[153]

^A^ Weight ratio.

^B^ The HOMO energy level of polymer donors.

^C^ Estimated from the absorption edge in film ($E_{g}^{opt}=1240/\lambda_{onset}$).

^D^ Average *PCE* of OSCs.

^a^ isopropyl alcohol (IPA).

^b^ 1.5% DIO.

^c^ 150°C annealing.

^d^ 0.5% DIO.

^e^ 1% DIO.

^f^ 100°C annealing.

^g^ 2% DIO.

^h^ 4% DIO.

By selecting ethane (ET), thiophene (T) and 1, 2, 4, 5‐tetrazine (TTz) as acceptor basic units, several novel main‐chain engineering was successfully constructed. In 2014, based on the main‐chain BDT‐T‐ET‐T, POT (2014) and PTT (2014) were reported by Liu et al.^[^ ^134^ ^]^, POT‐DH (2014), POT‐HCN (2014) and POT‐DCN (2014) were reported by Lu et al.^[^ ^135^ ^]^ After blending with acceptors, POT: PC_60_BM‐based, PTT: PC_60_BM‐based, POT‐DH: PC_71_BM‐based, POT‐HCN: PC_71_BM‐based and POT‐DCN: PC_71_BM‐based OSCs achieved the *V_oc_* of 0.795, 0.750, 0.66, 0.79 and 0.77 V; *J_sc_* of 0.138, 0.150, 9.36, 9.20 and 0.14 mA cm^−2^; *FF* of 28.83%, 25.65%, 60.3%, 57.9% and 28.8%; and *PCE* of 0.03%, 0.03%, 3.73%, 4.21% and 0.3%, respectively. Among them, the substitution of alkyloxy and “CN” group enhanced the electron‐donating of BDT unit as well as electron‐withdrawing of ET unit, while the *J_sc_* and *FF* in OSC devices were still low. Furthermore, compared with POT‐DCN: PC_71_BM‐based OSC, POT‐HCN with a suitable caynation degree makes POT‐HCN: PC_71_BM‐based OSC to obtain a better photoelectric performance. In addition, based on the main‐chain engineering BDT‐T‐T‐T and BDT‐T‐TTz‐T, Liu et al. also synthesized PQ1 (2015) and PQ2 (2015).^[^ ^136^ ^]^ Notably, the introduction of side chain 5, 5″‐didodecyl‐5′‐methyl‐2, 2′: 3′, 2″‐terthiophene with a wide conjugated plane also endows PQ1: PC_61_BM‐based OSC with a promising *V_oc_* (1.01 V). Interestingly, the *V_oc_* in PQ2: PC_71_BM‐based OSC decreases, whereas *J_sc_* and *FF* increase, which can be attributed to the TTz unit with a stronger electronegative than thiophene unit further promote the hole/electron mobility.

PB2 (2017) and PB2‐S (2017) were reported based on the main chain BDT‐T‐TT‐T,^[^ ^137^ ^]^ synthesizing by introducing the side chain 2‐octyldodecyl acetate into the TT unit as well as 2‐(2‐ethylhexyl)−5‐methylthiophene and 2‐((2‐ethylhexyl) thio)−5‐methylthiophene into the BDT unit in turn. Compared with PB2: ITIC‐based OSC, the substitution of alkylthio endows BDT unit of PB2‐S with a stronger electron‐donating, thus the faster and more balanced hole/electron mobility endows PB2‐S: ITIC‐based OSC with a higher *J_sc_* (14.61 mA cm^−2^) and *FF* (50.1%). Afterward, by changing the orientation of TT unit in polymer skeleton, TTC‐Cl (2022)^[^ ^138^ ^]^ achieved an excellent *V_oc_* (1.20 V) after co‐blending with the acceptor HCl‐BTA3, which can be attributed the significantly decreased HOMO energy level induced by more ideal blend film morphology. Furthermore, to further enhancing the electronegative of TT unit, by creating two active sites and introducing the functional group carbonyl, 4*H*, 6*H*‐5*λ* ^2^‐thieno [3, 4‐*c*] pyrrole‐4,6‐dione (TPD) unit with a strong electron‐withdrawing was successfully constructed. Subsequently, based on the polymer backbone BDT‐T‐TPD‐T, PBDT‐TPD (2014) and PBDTT‐TPD (2014) were designed by Kim et al.^[^ ^139^ ^]^; P1 (2016), PBDT‐Th‐TPD (2017), PBDT‐TTV (2018) and PTPD (2020) were prepared by Kranthiraja et al.,^[^ ^140^ ^]^ Kotturappa et al.,^[^ ^142^ ^]^ Kim et al.,^[^ ^142^ ^]^ and Zhao et al.^[^ ^143^ ^]^ in turn; a series of polymer donors TPD‐1 (2020), TPD‐2 (2020), TPD‐3 (2020) and TPD‐3F (2020) were also synthesized by Liao et al.^[^ ^144^ ^]^ After applying for OSC devices, the introduction of thiophene‐based and benzene‐based side chains further extend the π–π conjugated plane of polymer donors, thereby these polymer donor: acceptor‐based OSC achieved a promising *V_oc_*, especially for P1 (2016): PC_71_BM‐based OSC (0.99 V). Furthermore, based on the main chain of BDT‐T‐TPD‐T, due to the substitution of alkyl‐thiophene side chain extend the original π–π conjugated plane of polymer backbone as well as enhance the electron‐donating of BDT unit, meanwhile, the introduction of long alkyl chain also optimized the miscibility and blend film morphology of active layer. Therefore, TPD‐1: IT‐4F‐based, TPD‐2: IT‐4F‐based, TPD‐3: IT‐4F‐based and TPD‐3F: IT‐4F‐based OSCs achieved a significantly breakthrough. Among them, TPD‐3F with the substitution of fluorinated alkyl‐thiophene further increased the *V_oc_* (0.91 V) in TPD‐3F: IT‐4F‐based OSC, which is consistent with the previous conclusions.

In addition, after adjusting the substitution site of carbonyl groups in TPD unit, 1*H*, 4*H*‐2*λ* ^2^, 5*λ* ^2^‐pyrrolo [3, 4‐*c*] pyrrole‐1, 4‐dione (PPD) unit with an asymmetric structure also exhibits an excellent electron‐withdrawing. In 2011, Li et al. based on the main chain BDT‐T‐PPD‐T, prepared three polymer donors O‐HD (2011), BO‐BO (2011) and PU‐O (2011) by adjusting the alkyl side chains into the BDT and PPD units.^[^ ^145^ ^]^ After applying for OSC devices, O‐HD with the substitution of octyl and 7‐ethylpentadecane into the BDT and PPD units endows O‐HD: PC_71_BM‐based OSC with the best *J_sc_* (9.4 mA cm^−2^) and *FF* (61%), verifying that the suitable substitution of alkyl chains can effectively optimize the miscibility of donor materials and blend film morphology. Afterward, PBDTDPPcbp (2013) and PDPPBDT (2014) were reported by Han et al.^[^ ^146^ ^]^ and Tae et al.^[^ ^147^ ^]^ in turn. Compared with the PBDTDPPcbp: PCBM‐based OSC, the substitution of 11‐ethyltricosane increases the miscibility of PDPPBDT, thereby the *J_sc_* (7.12 mA cm^−2^) and *FF* (62%) in PDPPBDT: PC_71_BM‐based OSC both enhanced, which further proves the above‐conclusions. Subsequently, based on the alkyl chain substituted PPD unit, PQ3 (2015) was reported by Liu et al.,^[^ ^136^ ^]^ PBDTBA(H)‐DPP (2015) and PBDTBPA(F)‐DPP (2015) were prepared by Chakravarthi et al.,^[^ ^148^ ^]^ PBDTS‐DPP (2016), PBDTOT‐DPP (2016) and P2 (2016) were synthesized by Zhang et al.,^[^ ^73^ ^]^ Wang et al.,^[^ ^149^ ^]^ and Kranthiraja et al.,^[^ ^140^ ^]^ respectively. Similarly, PBDTT‐S‐DPP (2016),^[^ ^150^ ^]^ PBDTT‐Se‐DPP (2016),^[^ ^150^ ^]^ P3 (2016),^[^ ^151^ ^]^ P7(0.0: 1.0) (2016),^[^ ^152^ ^]^ PDPP‐BDT (2021)^[^ ^153^ ^]^ and PDPP‐BDT‐Cl (2021)^[^ ^153^ ^]^ were also prepared in subsequence. Notably, although the alkyl‐based, selenophene‐based, or benzene‐based side chains with a wide conjugated plane endow such polymer donors: PCBM‐based OSCs with a moderate *V_oc_* (∼0.80 V), while the dispersed electron density reduces the *J_sc_* and *FF*. As for P11 (2019),^[^ ^118^ ^]^ which is synthesized by introducing side chains 1‐methoxydodecane and octyl into the BDT and PPD units, respectively. Benefiting alkyloxy side chain enhanced the electron‐donating of BDT unit as well as long alkyl side chain further optimized the miscibility of P11, thus P11: PC_71_BM‐based OSC achieved a high *J_sc_* (13.88 mA cm^−2^) and *FF* (64%).

Furthermore, among these donors, PQ2, PQ3, PB2 and PTPD obtained a high *Td_5%_* of 445°C, 420°C, 410°C and 424°C, respectively. Moreover, after 170 h test for light soaking, PDPPDTT: PC_71_BM‐based OSC only experienced a 20% *PCE* drop, meanwhile, PBDTBPA(F)‐DPP: PC_71_BM‐based OSC still maintains 80% of the highest *PCE* after 1440 h storage in ambient conditions, which further demonstrating the promising stability of donor materials synthesized based on such main‐chain engineering in photovoltaic devices. In addition, based on the morphology characterization of POT‐DH: PC_71_BM‐based (1.75 nm), POT‐HCN: PC71BM‐based (2.35 nm) and POT‐DCN: PC71BM‐based (2.39 nm) blend films, the smoother active layer effectively accelerates the exciton dissociation of POT‐DH: PC_71_BM‐based OSC, thereby endowing it with a better FF (60.3%). Notably, compared with PQ1: PC_71_BM‐based (10.1 nm) and PQ3: PC_71_BM‐based (13.5 nm) blend films, PQ2: PC_71_BM‐based (3.95 nm) blend film, the smoother active layer endows PQ2: PC_71_BM‐based OSC with a higher *FF* (62.05%). Meanwhile, among PB2: ITIC‐based (0.73 nm) and PB2‐S: ITIC‐based (0.69 nm) blend films as well as PBDT‐Th‐TPD: PC_71_BM‐based (7.4 nm) and PBDT‐TTV: PC_70_BM based (1.3 nm) blend films, the decreased *RMS* also endows PB2‐S: ITIC‐based (50.1%) and PBDT‐TTV: PC_70_BM‐based (56%) OSCs with a higher *FF*. As for TPD‐1: IT‐4F‐based (1.03 nm), TPD‐2: IT‐4F‐based (0.93 nm), TPD‐3: IT‐4F‐based (0.89 nm) and TPD‐3F: IT‐4F‐based (1.19 nm) blend films, the formation of ideal blend film morphology endows corresponding OSCs with a high *FF*.

**FIGURE 14:**
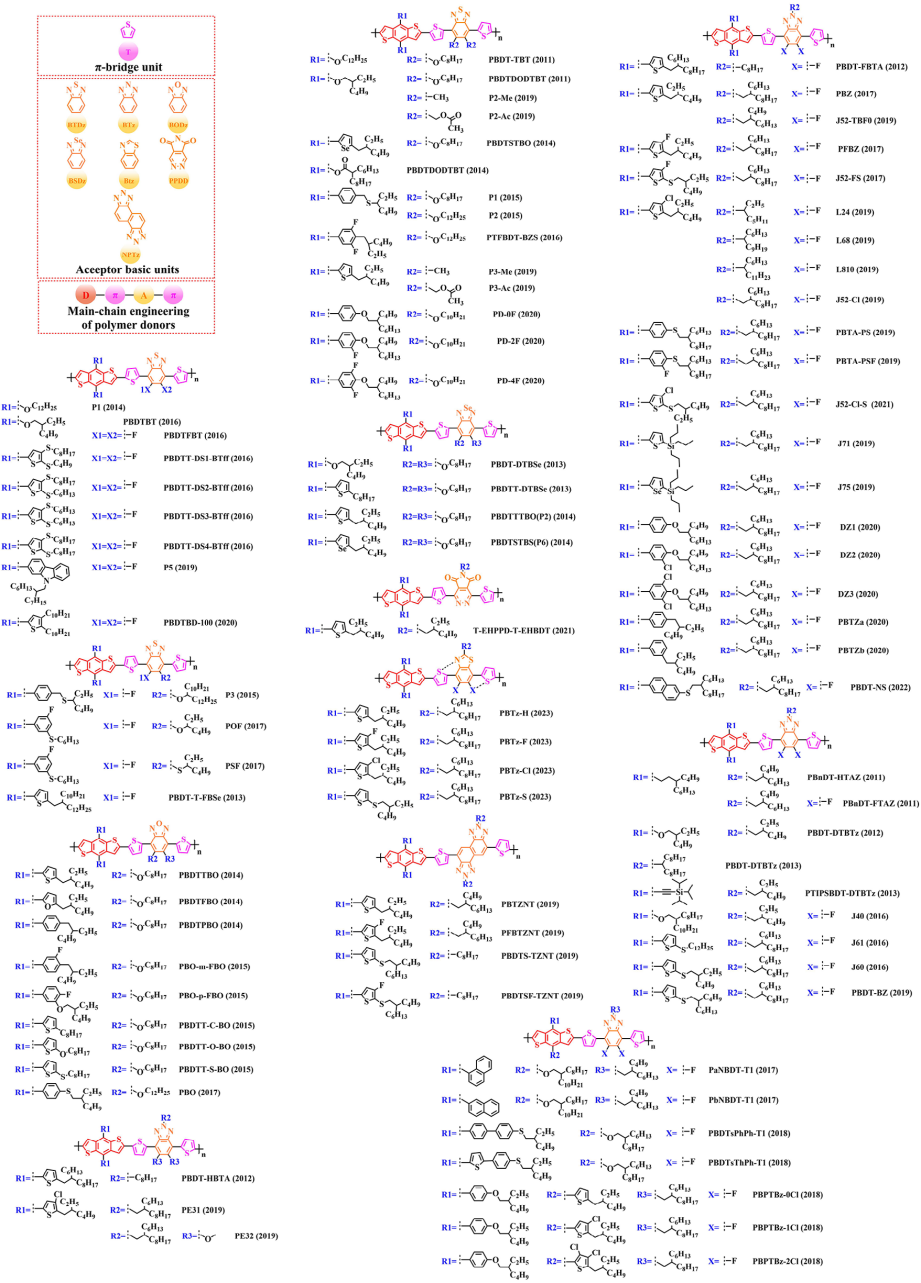
The detailed molecular structure of D‐π‐A‐π type polymer donors synthesized by introducing thiophene as π‐bridge unit, BTDz and its derivatives as acceptor basic units.

In recent years, the polymer backbone BDT‐T‐BTDz‐T constructed based on the acceptor unit benzo [*c*] [1, 2, 5] thiadiazole (BTDz), which promotes the emergence of abundant polymer donors. Afterward, by introducing “O”, “N” units as well as carbonyl group to adjusting the electronegativity of BTDz unit, a serious of novel acceptor units (e.g. 2*λ* ^2^‐benzo [*d*] [1, 2, 3] triazole (BTz), benzo [*c*] [1, 2, 5] ox diazole (BODz), benzo [*c*] [1, 2, 5] selenadiazole (BSDz), benzo [*d*] thiazole (Btz) and 6*λ* ^2^‐pyrrolo [3, 4‐*d*] pyridazine‐5, 7‐dione (PPDD) units) were successfully constructed. Afterward, based on such efficient acceptor units, the detailed molecular structure of representative polymer donors (Figure 14) and photovoltaic parameters in corresponding OSCs (Table 11) were discussed as follows.

**TABLE 11:** Photovoltaic parameters of OSCs related to Figure 14.

Donor	Acceptor	D/A^A^	HOMO [eV]^B^	$E_{g}^{opt}$ [eV]^C^	*V_OC_* [V]	*J_SC_* [mA cm^−2^]	*FF* [%]	*PCE* [%]^D^	Ref.
PBDT‐DTBSe^a^	PC_71_BM	1:1	−5.20		0.70	9.37	48	3.13	[85]
PBDTT‐DTBSe^a^	PC_71_BM	1:1	−5.10		0.67	10.23	46	3.18	[85]
PBDT‐TBT	PC_61_BM	1:3	−5.23	1.75	0.68	3.48	47	1.11	[154]
PBDT‐DODTBT	PC_71_BM	1:2	−5.17		0.74				[155]
PBDTSTBO(P4)	PC_71_BM	1:2	−5.46	1.78	0.86	13.9	64	7.52	[156]
PBDTTTBO(P1)	PC_71_BM	1:2	−5.46	1.78	0.85	11.8	59	5.76	[156]
PBDTSTBS(P6)	PC_71_BM	1:2	−5.29	1.69	0.67	11.4	52	3.88	[156]
P4^b^	PC_71_BM	1:1	−5.22	1.76	0.71	10.38	57.82	4.30	[157]
P1	PC_71_BM	1:2.5	−5.42	1.79	0.74	11.60	63	5.4	[158]
P2	PC_71_BM	1:2.5	−5.41	1.78	0.76	11.26	72	6.1	[158]
P3(PPBDTBT)	PC_71_BM	1:2	−5.60	1.76	0.82	12.28	73	7.4	[158]
PTFBDT‐BZS^c^	IDIC	1:1	−5.43		0.905	17.16	70.8	10.97	[159]
PD‐0F	rr‐PBN		−5.09	1.80	0.86	9.42	46.5	3.72	[160]
PD‐2F	rr‐PBN		−5.20	1.80	0.92	8.35	41.9	3.15	[160]
PD‐4F	rr‐PBN		−5.27	1.82	1.11	11.53	50.4	6.38	[160]
CO‐BDT‐DTBT	PC_71_BM	1:3	−5.62	1.90	0.89	3.6	31.6	1.00	[161]
P2‐Me	PC_71_BM	1:1	−5.39	2.06	0.68	5.09	26.2	0.86	[162]
P2‐Ac	PC_71_BM	2:1	−5.40	1.93	0.69	7.40	26.5	1.29	[162]
P3‐Me	PC_71_BM	1:1	−5.49	2.12	0.57	3.50	27.1	0.54	[162]
P3‐Ac^a^	PC_71_BM	1:1	−5.46	1.91	0.63	7.65	29.3	1.13	[162]
P1^d^	PC_70_BM	1:1	−5.24	1.58	0.84	10.52	60	5.30	[163]
PBDTBT^e^	PC_70_BM	1:2	−5.34	1.74	0.88	10.73	53.6	5.06	[164]
PBDTFBT	PC_61_BM	1:1	−5.51		0.87	7.98	52.2	3.62	[165]
PBDT‐T‐FBSe	PC_71_BM		−5.19	1.60	0.78	11.80	54	5.00	[166]
PBDTT‐DS1‐BTff^e^	PC_71_BM	1:1	−5.53	1.65	0.93	7.54	33.9	2.38	[167]
PBDTT‐DS2‐BTff^e^	PC_71_BM	1:1	−5.53	1.69	0.92	11.06	57.2	5.82	[167]
PBDTT‐DS3‐BTff^e^	PC_71_BM	1:1	−5.51	1.61	0.91	9.91	46.0	4.15	[167]
PBDTT‐DS4‐BTff^e^	PC_71_BM	1:1	−5.56	1.73	0.93	10.40	51.7	5.00	[167]
P5(2019)	PC_71_BM	1:2	−5.40	1.65	0.91	13.53	63.2	7.57	[168]
PBDTBD‐100	IT‐4F	1:1.5	−5.44	1.71	0.73	17.03	67.96	8.19	[169]
POF^a^	ITIC	1:2	−5.49	1.79	0.86	14.9	57	7.10	[170]
PSF^a^	ITIC	1:2	−5.66	1.78	0.96	5.6	29	1.46	[170]
PBnDT‐HTAZ	PC_61_BM	1:2	−5.29		0.70	11.14	55.2	4.30	[171]
PBnDT‐FTAZ	PC_61_BM	1:2	−5.36		0.79	11.83	78.9	6.81	[171]
PE31	Y6		−5.24	1.89	0.80	20.63	47.18	7.47	[301]
PE32	Y6		−5.20	1.92	0.75	18.69	51.95	7.26	[301]
PBDT‐DTBTz	PC_61_BM	1:2	−5.22	1.99	0.51	7.42	41.0	1.55	[172]
PBDT‐DTBTz^f^	PC_71_BM	1:1	−5.25	1.97	0.77	7.87	48	2.88	[173]
PTIPSBDT‐DTBTz^f^	PC_71_BM	1:1	−5.39	1.95	0.80	12.69	55	5.53	[173]
J40^g^	ITIC	1:1	−5.40		0.89	11.59	59.35	6.13	[174]
PBDT‐HBTA^h^	PC_70_BM	1:1	−5.13		0.58	7.41	56.5	1.70	[175]
PBDT‐FBTA^h^	PC_70_BM	1:1	−5.26		0.76	10.94	53.8	4.47	[175]
J60^i^	ITIC	1:1	−5.32	1.93	0.91	16.33	60.38	8.67	[176]
J61^i^	ITIC	1:1	−5.32	1.93	0.89	17.43	61.48	9.22	[176]
J61	BTA3	1:1	−5.32	1.94	1.15	10.53	66.17	8.25	[176]
PBZ	ITIC	1:1.25	−5.18	1.94	0.79	16.5	56	7.5	[177]
PFBZ (J52‐F)	ITIC	1:1.25	−5.36	1.94	0.89	18.3	60	9.8	[177]
J52‐F	BTA5		−5.36		1.17	13.80	69.82	10.95	[177]
PBDT‐BZ	BDTB‐Ph	1:1	−5.38	1.92	0.918	17.52	64.5	10.38	[178]
J52‐TBF0	ITIC	1:1	−5.34	1.94	0.832	11.50	58.6	5.49	[179]
J52‐FS^j^	BTA3	1:1	−5.32	1.93	1.24	6.82	45.34	3.83	[180]
L24	IT‐4F		−5.43		0.495	7.41	36.3	1.3	[181]
L68	IT‐4F		−5.51		0.758	19.50	63.2	9.1	[181]
L810	IT‐4F		−5.57		0.790	20.76	73.5	11.7	[181]
J52‐Cl	BTA3	1:1	−5.39		1.24	13.16	66.62	10.35	[181]
J71	m‐ITIC	1:1			0.94	17.53	71.20	11.45	[182]
J75^k^, ^l^	m‐ITIC	1:1	−5.49	1.93	0.96	17.11	69.47	11.27	[182]
PBTZa^m^	ITIC‐4Cl	1:1	−5.12	1.93	0.84	13.74	73.6	8.31	[182]
PBTZb^m^	ITIC‐4Cl	1:1	−5.20	1.93	0.87	21.75	76.8	14.34	[182]
J52‐Cl‐S	Y6		−5.60	1.96	0.86	22.23	54.86	10.23	[183]
PBTA‐PS	ITIC	1:1.2	−5.34	1.94	0.94	18.23	69.19	11.52	[184]
PBTA‐PSF	ITIC	1:1.2	−5.52	1.98	1.01	18.51	74.40	13.60	[184]
DZ1	MelC	1:1	−5.26	1.84	0.814	15.237	66.7	7.939	[185]
DZ2	MelC	1:1	−5.31	1.92	0.877	17.061	68.4	9.983	[185]
DZ3	MelC	1:1	−5.40	1.97	1.00	10.311	60.9	5.77	[185]
PBDT‐NS^n^	LC301	1:1.2			0.848	21.30	66.4	11.99	[186]
PaNBDT‐T1	ITIC	1:1.5	−5.40	1.95	0.87	18.53	59.63	9.41	[187]
PbNBDT‐T1	ITIC	1:1.5	−5.32	1.90	0.74	18.19	50.02	6.52	[187]
PBDTsPhPh‐T1	ITIC	1:2	−5.35	1.98	0.863	15.73	65.1	8.85	[188]
PBDTsThPh‐T1	ITIC	1:1.5	−5.30	1.97	0.846	17.52	63.1	9.34	[188]
PBPTBz‐0Cl	IT‐M	1:1	−5.27	1.96	0.81	14.26	52.95	6.15	[189]
PBPTBz‐1Cl	IT‐M	1:1	−5.32	1.97	0.87	14.78	50.20	6.46	[189]
PBPTBz‐2Cl	IT‐M	1:1	−5.41	1.98	0.99	14.92	48.61	7.18	[189]
PBDTTBO^o^	PC_71_BM	1:2	−5.46	1.78	0.86	12.8	67	7.4	[190]
PBDTPBO^o^	PC_71_BM	1:2	−5.40	1.78	0.85	11.8	64	6.4	[190]
PBDTFBO^b^	PC_71_BM	1:2	−5.38	1.78	0.81	11.2	60	5.4	[190]
PBO‐m‐FBO^e^	PC_71_BM	1:2	−5.53		0.88	14.2	63	7.9	[191]
PBO‐p‐FBO^e^	PC_71_BM	1:1	−5.54		0.95	10	53	5.0	[191]
PBDTT‐C‐BO	PC_71_BM	1:2	−5.42	1.77	0.74	15.7	64	7.2	[192]
PBDTT‐O‐BO	PC_71_BM	1:2	−5.37	1.74	0.71	13.0	58	5.3	[192]
PBDTT‐S‐BO	PC_71_BM	1:2	−5.46	1.77	0.86	13.2	58	6.4	[192]
PBO	PC_71_BM	1:2.5	−5.47	1.80	0.83	12.37	71	7.0	[193]
PBO^p^	ITIC	1:2	−5.47	1.80	0.92	14.3	54	6.9	[193]
T‐EHPPD‐T‐EHBDT	ITIC‐F	1:1	−5.47	1.79	0.778	16.1	58	7.21	[194]
PBTz‐H	L8‐BO		−5.31	2.04	0.756	25.62	60.00	11.23	[195]
PBTz‐S	L8‐BO		−5.38	2.05	0.841	26.09	68.04	14.35	[195]
PBTz‐F	L8‐BO		−5.43	2.06	0.896	26.71	77.59	18.37	[195]
PBTz‐Cl	L8‐BO		−5.47	2.07	0.922	26.39	74.35	17.84	[195]
PBTZNT^q^	m‐ITIC	1:1	−5.35	1.97	0.89	15.55	66.4	9.02	[196]
PFBTZNT^q^	m‐ITIC	1:1	−5.47	1.97	0.91	17.63	68.7	10.85	[196]
PBDTS‐TZNT^r^	IT‐4F	1:1	−5.39	1.99	0.87	18.54	68.2	11.00	[197]
PBDTSF‐TZNT^r^	IT‐4F	1:1	−5.45	1.97	0.92	19.13	73.4	12.92	[197]

^A^ Weight ratio.

^B^ The HOMO energy level of polymer donors.

^C^ Estimated from the absorption edge in film ($E_{g}^{opt}=1240/\lambda_{onset}$).

^D^ Average *PCE* of OSCs.

^a^ 180°C annealing for 5 min.

^b^ 1% DIO.

^c^ 0.25% DIO.

^d^ DIO and THF.

^e^ 3% DIO.

^f^ DIO.

^g^ 120°C annealing for 10 min.

^h^ DIO and 110°C annealing for 10 min.

^i^ 100°C annealing for 10 min.

^j^ 150°C annealing for 10 min.

^k^ 0.5% CN.

^l^ 130°C annealing for 2 min.

^m^ 100°C annealing.

^n^ 100°C annealing.

^o^ 1% CN.

^p^ 0.5% DIO.

^q^ 100°C annealing for 10 min.

^r^ 0.2% DIO.

Based on the main chain BDT‐T‐BTDz‐T, PBDT‐TBT (2011), PBDTDODTBT (2011) and PBDTSTBO (2014) were reported by Li et al.,^[^ ^154^ ^]^ Wang et al.,^[^ ^155^ ^]^ Jiang et al.^[^ ^156^ ^]^ and Kranthiraja et al.^[^ ^157^ ^]^ in turn. Similarly, Gong et al. synthesized P1 (2015) and P2 (2015)^[^ ^158^ ^]^; Lin et al. designed PTFBDT‐BZS (2016)^[^ ^159^ ^]^; Wang et al. also prepared PD‐0F (2020), PD‐2F (2020) and PD‐4F (2020), respectively.^[^ ^160^ ^]^ Among them, the substitution of selenophene‐based or benzene‐based effectively broaden the π–π conjugated plane of polymer donors, endowing PBDTSTBO: PC_71_BM‐based (0.86 V), P1: PC_71_BM‐based (0.74 V), P2: PC_71_BM‐based (0.76 V), PTFBDT‐BZS: IDIC‐based (0.905 V), PD‐0F: rr‐PBN‐based (0.86 V) and PD‐2F: rr‐PBN‐based (0.92 V) OSCs with a high *V_oc_*. Meanwhile, the highest *V_oc_* exhibited in PD‐4F: rr‐PBN‐based OSC (1.11 V) can be attributed to the decreased HOMO energy level (−5.27 eV) of PD‐4F as well as optimized molecular stacking in the active layer induced by introducing the substitution of fluorinated side chains. Furthermore, benefiting the more complemental absorption achieved by PTFBDT‐BZS: IDIC blend film, the higher *J_sc_* (17.16 mA cm^−2^) and *FF* (70.8%) endows PTFBDT‐BZS: IDIC‐based OSC with a more ideal *PCE* (10.97%). Moreover, based on the polymer backbone BDT‐T‐BTDz‐T with alkyl or carboxylate substituted BTDz unit, Kim and co‐workers reported CO‐BDT‐DTBT (2014) by introducing the side chain methyl 2‐hexyldecanoate into the BDT and BTDz units;^[^ ^161^ ^]^ Ratha et al. prepared a series of novel polymer donors P2‐Me (2016), P2‐Ac (2016), P3‐Me (2016) and P3‐Ac (2016).^[^ ^162^ ^]^ After blending with the acceptor PC_71_BM, the introduction of ethyl acetate endows BTDz unit with a stronger electronegative, thereby P2‐Ac: PC_71_BM‐based and P3‐Ac: PC_71_BM‐based OSCs both obtained a faster hole/electron mobility than P2‐Me: PC_71_BM‐based and PC‐Me: PC_71_BM‐based OSCs. In addition, by introducing the alkyloxy side chain into the BDT unit, Keshtov et al.^[^ ^163^ ^]^ and Shen et al.^[^ ^164^ ^]^ also synthesized P1 (2014) and PBDTBT (2014) based on the polymer backbone BDT‐T‐BTDz‐T. Compared with CO‐BDT‐DTBT: PC_71_BM‐based OSC, the substitution of alkyloxy enhanced the electron‐donating of BDT unit, which significantly increases *J_sc_* and *FF* in P1: PC_70_BM‐based and PBDTBT: PC_70_BM‐based OSCs. In addition, the substitution of long alkyl chain effectively optimized the blend film morphology of P1: PC_71_BM, thereby effectively increasing the *FF* in P1: PC_71_BM‐based OSC. Subsequently, based on the reported results of PBDTBT, Wang and co‐workers synthesized PBDTFBT (2016) by further introducing two “F” atoms into the BTDz unit, while the *J_sc_* and *FF* in PBDTBT: PC_71_BM‐based OSC are still low.^[^ ^165^ ^]^ Afterward, to further explore the potential application of fluorinated BTDz unit in main‐chain engineering, PDT‐T‐FBSe (2013) synthesized by Li et al.,^[^ ^166^ ^]^ a series of novel polymer donors PBDTT‐DS1‐BTff (2016), PBDTT‐DS2‐BTff (2016), PBDTT‐DS3‐BTff (2016) and PBDTT‐DS4‐BTff (2016) were reported by Deng et al.;^[^ ^167^ ^]^ P5 (2019) and PBDTBD‐100 (2020) were also designed by Li et al.^[^ ^168^ ^]^ and Jung et al.,^[^ ^169^ ^]^ respectively. Consistent with the above‐conclusions, due to the introduction of alkylthio‐thiophene or 9*λ* ^2^‐carbazole based side chains further broaden the π–π conjugated plane in polymer donors, PBDT‐T‐FBSe: PC_71_BM‐based (0.78 V), PBDTT‐DS1‐BTff: PC_71_BM‐based (0.93 V), PBDTT‐DS2‐BTff: PC_71_BM‐based (0.92 V), PBDTT‐DS3‐BTff: PC_71_BM‐based (0.91 V), PBDTT‐DS4‐BTff: PC_71_BM‐based (0.93 V) and P5 (2019): PC_71_BM‐based (0.91 V) OSCs obtained a high *V_oc_*. As for the PBDTBT‐100: IT‐4F‐based OSC, the significantly enhanced *J_sc_* (17.03 mA cm^−2^) and *FF* (67.96%) can be attributed to the formation of more complemental absorption in active layer. Moreover, P3 (2015), POF (2017) and PSF (2017) were also reported by Gong et al.,^[^ ^170^ ^]^ compared with PD‐0F: rr‐PBN‐based, PD‐2F: rr‐PBN‐based and PD‐4F: rr‐PBN‐based OSCs, the formation of more complemental absorption and optimized blend film morphology also endows P3 (2015): PC_71_BM‐based and PSF: ITIC‐based OSCs with an enhanced *J_sc_* and *FF*.

Similar to the main‐chain engineering BDT‐T‐BTDz‐T, a series of novel polymer skeleton BDT‐T‐BTz‐T, BDT‐T‐BODz and BDT‐T‐PPDD‐T were also successfully fabricated by different research teams. Afterward. based on the main chain BDT‐T‐BTz‐T, PBnDT‐HTAZ (2011) and PBDT‐FTAz (2011) were reported by price et al.;^[^ ^171^ ^]^ PBDT‐DTBTz (2012) was designed by Yang et al;^[^ ^172^ ^]^ PBDT‐DTBTz (2013) and PTIPSBDT‐DTBTz (2013) were prepared by Kim et al.;^[^ ^173^ ^]^ Bin and co‐workers also synthesized J40 (2016).^[^ ^174^ ^]^ After blending with acceptors, the fluorinated BTz unit with a stronger electron‐withdrawing further decreases the HOMO energy level of PBDT‐FTAZ (−5.36 eV) as well as accelerates the hole/electron mobility in the active layer. Therefore, PBDT‐FTAZ: PC_61_BM based OSC achieves a higher *V_oc_* (0.79 V), *J_sc_* (11.83 mA cm^−2^) and *FF* (78.9%) than PBDT‐HTAZ: PC_61_BM‐based OSC. Interestingly, this phenomenon also occurs between PBDT‐DTBTz: PC_61_BM‐based and J40: ITIC‐based OSCs. In addition, considering to introduce thiophene‐based side chain into the BDT unit, PBDT‐HBTA (2012) and PBDT‐FBTA (2012) were reported by Min et al.^[^ ^175^ ^]^ Benefiting the introduction of “F” atom with electron‐withdrawing and small atomic radius, PBDT‐FBTA: PC_70_BM‐based OSC obtain a lower HOMO energy level (−5.26 eV) and higher *V_oc_* (0.76 V). Meanwhile, the enhanced electron‐withdrawing of fluorinated BTz unit also further promote the hole/electron mobility, which endows PBDT‐FBTA: PC_70_BM‐based OSC with a higher *J_sc_* (10.94 mA cm^−2^). Afterward, based on the reported results of PBDT‐HBTA, Tang et al. ^[^ ^301^ ^]^ further designed PE31 (2019) and PE32 (2019) by introducing chlorinated thiophene side chain as well as methoxy group into the BDT and BTz units, respectively. Notably, higher *V_oc_* and *J_sc_* endows PE31: Y6‐based and PE32: Y6‐based OSCs with a more ideal *PCE*. Moreover, based on the reported results of PBDT‐FBTA, J61 (2016) and J60 (2016) were reported by Bin et al.;^[^ ^176^ ^]^ PBZ (2017), PBDT‐BZ (2019) and J52‐TBF0 (2019) were also synthesized by Fan et al.,^[^ ^177^ ^]^ Wang et al.^[^ ^178^ ^]^ and Wang et al.,^[^ ^179^ ^]^ respectively. Interestingly, compared with PBDT‐FBTA: PC_70_BM‐based OSC, the introduction of alkylthio with electron‐donating effectively promotes the hole/electron mobility in the active layer, thereby J61: ITIC‐based, J60: ITIC‐based and PBDT‐BZ: BDTB‐Ph‐based OSCs achieved a significantly increased *J_sc_* and *FF*. Meanwhile, the substitution of solubilized alkyl chain (e.g. 7‐ethylpentadecane or 5‐ethylundecane) also increased the miscibility in the active layer, the more complemental absorption presented by PBZ: ITIC based (16.5 mA cm^−2^) and J52‐TBF0: ITIC‐based (11.50 mA cm^−2^) OSCs effectively enhances the *J_sc_*. Afterward, based on the reported results of PBZ (2017), PFBZ (2017) and J52‐FS (2017) were reported by Fan et al.^[^ ^177^ ^]^ and Tang et al.^[^ ^180^ ^]^ Likewise, L24 (2019), L68 (2019) and L810 (2019) were synthesized by Liao et al.^[^ ^181^ ^]^ At the same year, Tang and co‐workers also prepared J52‐Cl (2019).^[^ ^182^ ^]^ As for J52‐Cl‐S (2022),^[^ ^183^ ^]^ which was designed based on J52‐Cl, synthesized by introducing alkylthio substituted thiophene side chain into the BDT unit. Compared with PBZ: ITIC‐based OSC, PBFZ: ITIC‐based and J52‐FS: BTA3‐based OSCs obtained the *V_oc_* of 0.89 and 1.24 V, *J_sc_* of 18.3 and 6.82 mA cm^−2^, *FF* of 60% and 45.34%, and *PCE* of 9.8% and 3.83%, respectively. Indeed, the introduction of fluorinated thiophene‐based side chain significantly enhanced the *V_oc_* in OSC devices, especially for J52‐FS: BTA3‐based OSC. Meanwhile, fluorinated thiophene‐based side chain also conducive to optimize the hole/electron mobility and blend film morphology in the active layer, which endows PFBZ: ITIC‐based OSC with a higher *J_sc_* (18.3 mA cm^−2^) and *FF* (60%). Furthermore, the decreased *J_sc_* and *FF* in J52‐FS: BTA3‐based OSC can be attributed to the poor miscibility of J52‐FS as for L24: IT‐4F‐based, L68: IT‐4F‐based, L810: IT‐4F‐based and J52‐Cl: BTA3‐based OSCs, the introduction of “Cl” atoms with a strong electronegative significantly enhances the *V_oc_* in such OSCs, especially for J52‐Cl: BTA3‐based OSC (1.24 V). Notably, compared with L24: IT‐4F‐based OSC, L68: IT‐4F‐based and L810: IT‐4F‐based OSCs obtained the *J_sc_* of 19.50 and 20.76 mA cm^−2^, *FF* of 63.2% and 73.5% and *PCE* of 9.1% and 11.7%, respectively. Consistent with the above‐conclusion, the substitution of long alkyl chain effectively optimizes the miscibility and blend film morphology in the active layer. Afterward, based on the reported results of PBZ, Zhong et al. synthesized J71 (2019) and J75 (2019) by introducing side chains (5‐methylthiophen‐2‐yl) tripropylsilane and (5‐methylselenophen‐2‐yl) tripropylsilane into the BDT unit to replace the original substitutions.^[^ ^182^ ^]^ Notably, the miscibility of J71 and J75 within the active layer was effectively optimized by introducing side chain methyltripropylsilane, thus J71: m‐ITIC‐based and J75: m‐ITIC‐based OSCs both achieved higher *J_sc_* and *FF*. Similarly, to further exploring the potential effect of introducing benzene‐based or naphthalene‐based side chains into the polymer backbone BDT‐T‐BTz‐T, PBTA‐PS (2019) and PBTA‐PSF (2019) were reported by Li et al.;^[^ ^184^ ^]^ DZ1 (2020), DZ2 (2020) and DZ3 (2020) were synthesized by Zhang et al.;^[^ ^185^ ^]^ PBTZa (2020) and PBTZb (2020) were prepared by Xue et al.;^[^ ^182^ ^]^ and PBDT‐NS (2022) was designed by Li and co‐workers,^[^ ^186^ ^]^ respectively. After applying for OSC devices, PBTA‐PS: ITIC‐based, PBTA‐PSF: ITIC‐based, DZ1: MelC‐based, DZ2: MelC‐based, DZ3: MelC‐based, PBTZa: ITIC‐4Cl‐based, PBTZb: ITIC‐4Cl‐based and PBDT‐NS: LC301‐based OSCs obtained the *PCE* of 11.52%, 13.60%, 7.939%, 9.983%, 5.77%, 8.31%, 14.34% and 11.99%, respectively. Consistent with the previous conclusions, benzene‐based or naphthalene‐based side chains effectively broaden the π–π conjugated plane of polymer donors, endowing corresponding OSCs with a promising *V_oc_*, especially for PBTA‐PSF‐based and DZ3‐based OSCs. Furthermore, compared with PBTA‐PS: ITIC OSC, the introduction of “F” atom further optimized molecular stacking and hole/electron mobility of PBTA‐PSF: ITIC‐based blend film, which endows PBTA‐PSF: ITIC‐based OSC with a higher *J_sc_* and *FF*. Meanwhile, compared with DZ1: MelC‐based and DZ3: MelC‐based OSCs, the better photoelectric performance achieved in DZ2: MelC‐based OSC also verified that only appropriate chlorination degree is conducive to improve the photovoltaic performance of OSCs. Moreover, by adjusting the substitution sites of solubilized alkyl chain in benzene‐based side chain, PBTZb (meta‐substituted) based OSC also achieved a better photoelectric performance than PBTZa (Para‐substituted) based OSC. Therefore, the effect of steric hindrance in donor materials is also an essential factor cannot be ignored. Furthermore, based on the reported results of PBDT‐NS, Liu and co‐workers synthesized PaNBDT‐T1 (2017) and PbNBDT‐T1 (2017) by simultaneously introducing side chains naphthalene and alkoxy into the BDT unit.^[^ ^187^ ^]^ Similarly, Zhong et al. prepared PBDTsPhPh‐T1 (2018) and PBDTsThPh‐T1 (2018);^[^ ^188^ ^]^ PBDTBz‐0Cl (2019), PBDTBz‐1Cl (2019) and PBDTBz‐2Cl (2019) were reported by Chao et al.^[^ ^189^ ^]^ Compared to PbNBDT‐T1, PaNBDT‐T1 with the substitution of 1‐methylnaphthalene significantly enhanced the *V_oc_* in PaNBDT‐T1: ITIC‐based OSC (0.87 V), proving polymer donors with substitutions into the different substituted sites will produce a significantly effect on photovoltaic performance in corresponding OSC devices. Moreover, with the deepening of chlorination degree, PBPTBz‐0Cl: IT‐M‐based, PBPTBz‐1Cl: IT‐M‐based and PBPTBz‐2Cl: IT‐M‐based OSCs obtained the *V_oc_* of 0.81, 0.87 and 0.99 V; *J_sc_* of 14.26, 14.78 and 14.92 mA cm^−2^; and *FF* of 52.95%, 50.20% and 48.61%, respectively. Although the *V_oc_* in such OSC devices was improved, the enhanced molecular polarity leads to decreased miscibility in the active layer, causing a low *FF*.

By constructing the main chain BDT‐T‐BODz‐T, Jiang et al. synthesized PBDTTBO (2014), PBDTTFBO (2014) and PBDTPBO (2014) by introducing the side chain 1‐methoxyoctane into the BODz unit as well as 2‐(2‐ethylhexyl)−5‐methylthiophene, 2‐(2‐ethylhexyl)−5‐methylfuran, and 1‐(2‐ethylhexyl)−4‐methylbenzene into the BDT unit, respectively.^[^ ^190^ ^]^ Likewise, based on the polymer backbone BDT‐T‐BODz‐T, PBO‐m‐FBO (2015) and PBO‐p‐FBO (2015) were reported by Yuan et al.^[^ ^191^ ^]^; PBDTT‐C‐BO (2015), PBDTT‐O‐BO (2015) and PBDTT‐S‐BO (2015) were synthesized by Jiang et al.;^[^ ^192^ ^]^ PBO (2017) was also prepared by Gong et al.,^[^ ^193^ ^]^ respectively. After applying for OSC devices, the *V_oc_* in polymer donor: acceptor‐based OSCs stay at a high level, especially for PBDTT‐O‐BO: PC_71_BM‐based OSC (0.95 V) and PBO: ITIC‐based OSC (0.92 V), which can be attributed to the extended π–π conjugated plane induced by introducing benzene‐based side chain. Moreover, compared with the PBDTT‐C‐BO: PC_71_BM‐based OSC (0.74 V), the substitution of alkylthio with strong electron‐donating also endows PBDTT‐S‐BO: PC_71_BM‐based OSC with a significantly enhanced *V_oc_* (0.86 V), whereas PBDTT‐O‐BO with the substitution of alkyloxy decreases the *V_oc_* in PBDTT‐P‐BO: PC_71_BM‐based OSC (0.71 V).

As for the main‐chain engineering BDT‐T‐BSDz‐T, BDT‐T‐Btz‐T and BDT‐T‐PPDD‐T, a series of breakthroughs have also been made. In 2013, shin and co‐workers synthesized PBDT‐DTBSe (2013) and PBDTT‐DTBSe (2013) based on the polymer skeleton BDT‐T‐BSDz‐T.^[^ ^85^ ^]^ Similarly, Jiang et al. also based on the main chain BDT‐T‐BSDz‐T, prepared PBDTTTBO(P2) (2014) and PBDTSTBS(P6) (2014).^[^ ^156^ ^]^ Afterward, Knall et al. prepared T‐EHPPD‐T‐EHBDT (2021) based on the polymer backbone BDT‐T‐PPDD‐T.^[^ ^194^ ^]^ In 2023, Pang and co‐workers synthesized a series efficient polymer donors PBTz‐H (2023), PBTz‐S (2023), PBTz‐F (2023) and PBTz‐Cl (2023) based on the polymer skeleton BDT‐T‐Btz‐T.^[^ ^195^ ^]^ After blending with acceptors, PBDT‐DTBSe: PC_71_BM‐based, PBDTT‐DTBSe: PC_71_BM‐based, PBDTTTBO: PC_71_BM‐based, PBDTSTBS: PC_71_BM‐based, T‐EHPPD‐T‐EHBDT: ITIC‐F‐based, PBTz‐H: L8‐BO‐based, PBTz‐S: L8‐BO‐based, PBTz‐F: L8‐BO‐based and PBTz‐Cl: L8‐BO‐based OSCs obtained the *PCE* of 3.13%, 3.18%, 5.76%, 3.88%, 7.21%, 11.23%, 13.35%, 18.37% and 17.84%, in that order. Consistent with the above‐conclusion, the substitution of thiophene‐based side chain broadens the original conjugated plane of donor materials, especially for PBTz‐F and PBTz‐Cl, meanwhile, the introduction of “F” and “Cl” atoms also further decreases the HOMO energy level of donor materials. It is also worth mentioning that main‐chain engineering constructed by introducing acceptor unit with a stronger electronegativity can usually match with BDT‐based donor unit, thus promoting hole/electron mobility in the active layer, which explains the reason why such polymer donors synthesized based on the polymer backbone BDT‐T‐Btz‐T and BDT‐T‐PPDD‐T always exhibited a more complemental absorption with acceptors than others synthesized based on the main chain BDT‐T‐BSDz‐T, thereby achieves a more ideal *J_sc_* and *FF* in corresponding OSC devices. In addition, Li et al.^[^ ^196^ ^]^ and Feng et al.^[^ ^197^ ^]^ designed a novel acceptor unit 2*λ* ^2^, 7*λ* ^2^‐naphtho [1, 2‐*d*: 5, 6‐*d*’] bis ([1, 2, 3] triazole) (NPTz) in 2019. After constructed the main chain BDT‐T‐NPTz‐T, a series of polymer donors PBTZNT (2019), PFBTZNT (2019), PBDTS‐TZNT (2019) and PBDTSF‐TZNT (2019) were successfully synthesized. Among them, PBDTS‐TZNT: IT‐4F‐based and PBDTSF‐TZNT: IT‐4F‐based OSCs obtained a better photovoltaic performance than PBTZNT: m‐ITIC‐based and PFBTZNT: m‐ITIC‐based OSCs, which can be attributed to the further enhanced electron‐donating of BDT unit in the main chain BDT‐T‐NPTz‐T induced by introducing the substitution of alkylthio. Therefore, the dissociation and translation of exciton have accelerated in the active layer.

Moreover, based on the characterization of blend film morphology and device stability, the high *Td_5%_* obtained by PBDTBT (419°C), PBDTBD‐100 (393°C), J75 (448°C), DZ3 (400°C), PBPTBz‐0Cl (428°C), PBPTBz‐1Cl (412°C), PBPTBz‐2Cl (426°C) and T‐EHPPD‐T‐EHBDT (415°C) exhibiting a promising application. Meanwhile, due to the formation of an ideal blend film morphology, the suitable *RMS* of PTFBDT‐BZS: IDIC‐based (0.904 nm), PBTZa: ITIC‐4Cl‐based (1.44 nm), PBTZb: ITIC‐4Cl‐based (1.26 nm), PBTz‐S: L8‐BO‐based (0.843 nm), PBTz‐F: L8‐BO‐based (1.02 nm) and PBTz‐Cl: L8‐BO‐based (0.919 nm) blend films endows corresponding OSCs with a high *FF* of 70.8%, 73.6%, 76.8%, 68.04%, 77.59%, and 74.35%, respectively.

**FIGURE 15:**
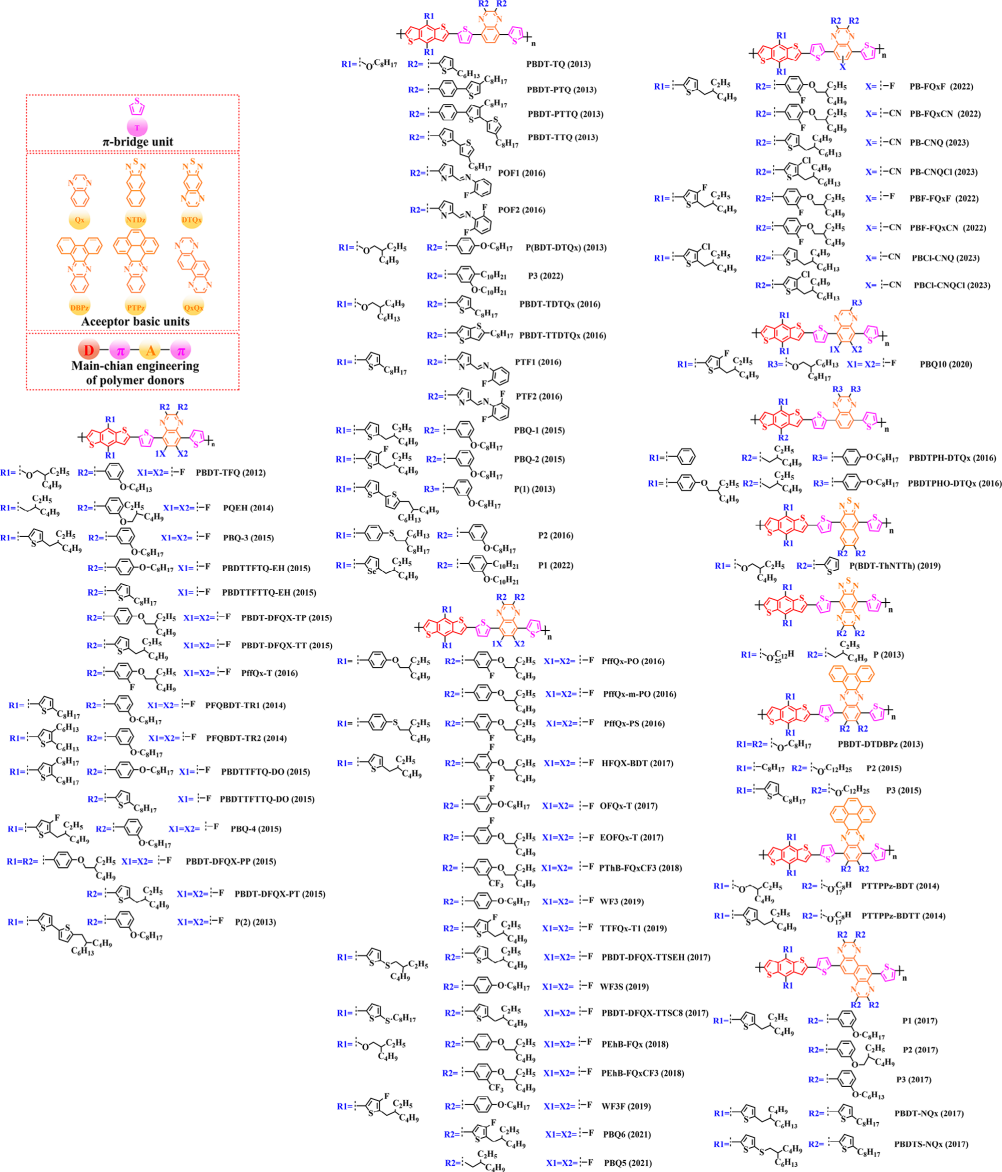
The detailed molecular structure of D‐π‐A‐π type polymer donors synthesized by introducing thiophene as π‐bridge unit, Qx and its derivatives as acceptor basic units.

Quinoxaline (Qx), a planar group with strong electronegativity, as an efficient acceptor unit in the main‐chain engineering has promoted the emergence of plenty efficient polymer donors. Therefore, considering the success achieved by Qx unit, a series efficient derivative unit of Qx (e.g. naphtho [2, 3‐*c*] [1, 2, 5] thiadiazol (NTDz), [1, 2, 5] thiadiazolo [3, 4‐*g*] quinoxaline (DTQx), dibenzo [*a*, *c*] phenazine (DBPz) and phenanthro [4, 5‐*abc*] phenazine (PTPz)) have also been fabricated. Among them, the detailed molecular structure (Figure 15) of representative polymer donors and photovoltaic performance (Table 12) in corresponding OSCs were discussed as follows.

**TABLE 12:** Photovoltaic parameters of OSCs related to Figure 15.

Donor	Acceptor	D/A^A^	HOMO [eV]^B^	$E_{g}^{opt}$ [eV]^C^	*V_OC_* [V]	*J_SC_* [mA cm^−2^]	*FF* [%]	*PCE* [%]^D^	Ref.
P(BDT‐ThNTTh)	PC_71_BM	1:2	−5.33	1.47	0.48	10.49	26.2	1.19	[89]
P(BDT‐DTQx)	PC_71_BM		−5.43	1.68	0.65	6.3	55.2	2.3	[92]
PBDT‐DTDBPz	PC_71_BM	1:2	−5.23	1.86	0.76	9.67	64.6	4.75	[96]
P2^a^	PC_61_BM	1:2	−5.34	1.73	0.81	7.72	68.79	4.28	[151]
PBDT‐TQ	PC_70_BM		−4.99	1.61	0.65	12.5	54	4.39	[198]
PBDT‐TTQ	PC_70_BM		−5.08	1.62	0.62	4.80	51	1.52	[198]
PBDT‐PTQ	PC_70_BM		−5.11	1.62	0.66	10.53	52	3.58	[198]
PBDT‐PTTQ	PC_70_BM		−5.18	1.62	0.72	8.13	45	2.62	[198]
PBDT‐TDTQx	PC_71_BM	1:2	−5.34	1.79	0.80	5.67	50	2.26	[199]
PBDT‐TTDTQx	PC_71_BM	1:2	−5.40	1.74	0.78	8.50	51	3.42	[199]
PTF1^b^	PC_70_BM	1:1	−4.97	1.56	0.72	4.97	29	1.04	[200]
POF1	PC_70_BM	1:1.5	−4.84	1.63	0.79	5.17	30	1.22	[200]
PTF2	PC_70_BM	1:1.5	−4.88	1.56	0.72	8.70	34	2.13	[200]
POF2	PC_70_BM	1:1	−4.78	1.63	0.77	5.25	40	1.60	[200]
BDT‐T‐Qx‐T(P1)^c^	PC_71_BM	1:2	−5.56	1.79	0.80	8.39	60.8	4.10	[201]
BDT‐T‐Qx‐T(P3)	PC_71_BM	1:1	−5.63	1.82	0.80	10.08	55.5	4.47	[201]
PBDT‐TFQ^d^, ^e^	PC_71_BM	1:1	−5.52	1.73	0.76	17.9	57.6	7.8	[202]
PEhB‐FQx	PC_71_BM	1:1	−5.35	1.75	0.68	11.1	63.5	4.68	[203]
PEhB‐FQxCF3	PC_71_BM	3:4	−5.42	1.74	0.82	11.0	61.9	5.43	[203]
PThB‐FQxCF3	PC_71_BM	3:4	−5.58	1.75	0.88	12.2	67.8	7.26	[203]
PQEH^d^, ^f^	PC_71_BM	1:1	−5.35	1.81	0.78	12.72	64.3	6.22	[204]
P(2)	PC_61_BM	1:1	−5.96		0.90	10.2	58	5.3	[205]
P(1)^g^	PC_71_BM	1:1	−5.68		0.74	9.5	53	3.7	[205]
PBQ‐1^h^	PC_71_BM	1:1.5	−5.05	1.64	0.63	12.39	69.81	5.40	[206]
PBQ‐2^h^	PC_71_BM	1:1.5	−5.19	1.66	0.75	12.96	63.14	6.13	[206]
PBQ‐3^h^	PC_71_BM	1:1.5	−5.19	1.68	0.79	13.46	67.58	7.19	[206]
PBQ‐4^h^	PC_71_BM	1:1	−5.35	1.73	0.90	13.52	70.02	8.52	[206]
PFQBDT‐TR1	PC_61_BM	1;1	−5.89	1.70	0.84	11.1	61	5.7	[207]
PFQBDT‐T2R2	PC_61_BM	1:2	−5.87	1.77	0.96	6.5	54	3.4	[207]
PBDTTFTQ‐EH^i^	PC_71_BM	1:1.5	−5.29	1.68	0.78	13.2	70.8	7.15	[208]
PBDTTFTQ‐DO^j^	PC_71_BM	1:3	−5.37	1.72	0.88	11.4	75.9	7.42	[208]
PBDTTFTTQ‐EH^j^	PC_71_BM	1:2	−5.12	1.66	0.72	13.4	70.8	6.65	[208]
PBDTTFTTQ‐DO^j^	PC_71_BM	1:3	−5.31	1.72	0.85	11.3	75.7	7.09	[208]
PBDT‐DFQX‐PP^k^	PC_71_BM	1:2	−5.35	1.83	0.89	8.66	51.38	3.96	[209]
PBDT‐DFQX‐TP	PC_71_BM	1:1	−5.25	1.78	0.82	12.34	60.12	6.08	[209]
PBDT‐DFQX‐PT	PC_71_BM	1:1	−5.29	1.75	0.85	12.13	63.46	6.54	[209]
PBDT‐DFQX‐TT	PC_71_BM	1:1.2	−5.20	1.72	0.86	12.77	69.93	7.68	[209]
PffQx‐T^k^	ITIC	1:1.5	−5.36	1.73	0.87	16.33	59.6	8.31	[210]
HFQx‐BDT^l^	ITIC	1:1.125	−5.45	1.76	0.92	15.59	65	9.25	[211]
OFQx‐T^m^	PC_71_BM	1:1.5	−5.36	1.70	0.86	12.43	70.70	7.34	[212]
EHFQx‐T^n^	PC_71_BM	1:1.5	−5.45	1.71	0.91	13.31	69.70	8.27	[212]
PBDT‐DFQX‐TTSEH^o^	PC_71_BM	1:1.2	−5.48	1.73	0.84	13.59	61.34	6.44	[213]
PBDT‐DFQX‐TTSC8^o^	PC_71_BM	1:1.2	−5.37	1.72	0.84	14.12	69.32	8.10	[213]
WF3^p^	PC_71_BM	1:2	−5.44	1.73	0.77	16.02	63.1	7.71	[214]
WF3S^p^	PC_71_BM	1:1.5	−5.45	1.75	0.82	16.11	63.6	7.91	[214]
WF3F^p^	PC_71_BM	1:2	−5.62	1.78	0.88	16.26	64.5	9.11	[214]
TTFQx‐T1^q^, ^r^	Y5	1:1.2	−5.31	1.70	0.89	21.2	69.6	12.9	[215]
PBQ10	Y6	1:1.2	−5.47	1.92	0.85	25.77	74.6	16.15	[216]
PBQ5^s^, ^t^	Y6	1:1.3	−5.55	1.88	0.839	25.81	70.23	15.22	[217]
PBQ6^s^, ^t^	Y6	1:1.3	−5.64	1.71	0.845	26.33	77.47	17.34	[217]
PB‐FQxF^u^	Y6	1:1.5	−5.04	1.79	0.54	22.97	52.39	6.46	[218]
PBF‐FQxF^u^	Y6	1:1.5	−5.08	1.81	0.63	24.00	58.06	8.73	[218]
PB‐FQxCN^u^	Y6	1:1.5	−5.17	1.68	0.68	25.15	63.94	10.90	[218]
PBF‐FQxCN^u^	Y6	1:1.5	−5.26	1.69	0.76	24.19	62.59	11.51	[218]
PB‐CNQ	Y6	1:1	−5.42	1.61	0.71	29.83	52.74	11.19	[219]
PBCl‐CNQ	Y6	1:1	−5.56	1.65	0.80	27.68	57.76	12.80	[219]
PB‐CNQCl	Y6	1:1	−5.49	1.59	0.77	27.49	65.04	13.98	[219]
PBCl‐CNQCl	Y6	1:1	−5.52	1.63	0.83	21.50	54.70	9.84	[219]
PffQx‐m‐fPO^d^	PC_71_BM	1:2			0.95	12.2	61	6.7	[220]
PffQx‐PO^u^	PC_71_BM	1:1			0.92	12.4	65	7.2	[220]
PffQx‐PS^d^	PC_71_BM	1:2			0.93	12.3	70	7.8	[220]
PBDTPH‐DTQx	PC_71_BM	1:2	−5.37	1.75	0.70	11.89	67.3	5.47	[221]
PBDTPHO‐DTQx	PC_71_BM	1:2	−5.34	1.74	0.75	9.60	60.4	4.25	[221]
P^r^	PC_70_BM				0.72	11.47	62	5.12	[222]
P2	PC_71_BM	1:2	−5.25	1.73	0.79	8.59	42	2.85	[223]
P3	PC_71_BM	1:2	−5.21	1.65	0.80	9.21	50	3.68	[223]
PTTPPz‐BDT	PC_71_BM		−5.29	1.70	0.74	9.79	58.2	4.25	[224]
PTTPPz‐BDTT	PC_71_BM		−5.34	1.59	0.70	11.10	62.5	4.86	[224]
P1	PC_71_BM	1:2	−5.16	1.65	0.80	4.9	43	1.7	[225]
P2	PC_71_BM	1:2	−5.16	1.77	0.88	9.1	48	3.8	[225]
P3	PC_71_BM	1:2	−5.16	1.70	0.79	8.0	42	2.7	[225]
PBDT‐NQx^v^	ITIC	1:0.8	−5.24	1.80	0.87	16.21	64.6	8.86	[226]
PBDTS‐NQx	PC_71_BM	1:1.5	−5.31		0.85	12.71	65.3	6.72	[226]

^A^ Weight ratio.

^B^ The HOMO energy level of polymer donors.

^C^ Estimated from the absorption edge in film ($E_{g}^{opt}=1240/\lambda_{onset}$).

^D^ Average *PCE* of OSCs.

^a^ DIO.

^b^ 180°C annealing for 5 min.

^c^ 120°C annealing for 10 min.

^d^ 3% DIO.

^e^ 2% DPE.

^f^ 1% CN.

^g^ 0.25% DIO.

^h^ 1% DIO.

^i^ DIO and 110°C annealing for 10 min.

^j^ 150°C annealing for 10 min.

^k^ 0.5% CN.

^l^ 150°C annealing and CF.

^m^ 130°C annealing for 2 min.

^n^ 100°C annealing.

^o^ 80°C annealing.

^p^ 100°C annealing.

^q^ DIO and THF.

^r^ 100°C annealing for 10 min.

^s^ 0.2% DIO.

^t^ 90°C annealing for 5 min.

^u^ 180°C annealing for 5 min.

^v^ 0.5% DIO.

Based on the main chain BDT‐T‐Qx‐T, Qin and co‐workers synthesized a series of novel polymer donors PBDT‐TQ (2013), PBDT‐TTQ (2013), PBDT‐PTQ (2013) and PBDT‐PTTQ (2013), which were designed by introducing the functional side chain alkyloxy into the BDT as well as thiophene‐based and benzene‐based side chains into the Qx unit, in that turn.^[^ ^198^ ^]^ Similarly, P(BDT‐DTQx) (2013) was prepared by Fu et al^[^ ^92^ ^]^; PBDT‐TDTQx (2016) and PBDT‐TTDTQx (2016) were reported by Kim et al^[^ ^199^ ^]^; POF1 (2016) and POF2 (2016) were designed by Singh et al^[^ ^200^ ^]^; P3 (2022) was also prepared by Aslan and co‐workers, respectively.^[^ ^201^ ^]^ Notably, after blending with the acceptor PC_71_BM, PBDT‐TDQx and PBDT‐TTDTQx with the substitution of solubilized alkyl chain 2‐butyloctyl effectively improve the miscibility of donor in the active layer. Furthermore, benefiting benzene‐based side chain broads the conjugated plane of P3 as well as “F” atoms further decrease the HOMO energy level of POF1 and POF2, thus PBDT‐TDTQx: PC_71_BM‐based (0.80 V), PBDT‐TTDTQx: PC_71_BM‐based (0.78 V), POF1: PC_70_BM‐based (0.79 V), POF2: PC_70_BM‐based (0.77 V) and P3: PC_71_BM‐based (0.80 V) OSCs obtained a higher *V_oc_* than PBDT‐TQ: PC_70_BM‐based OSC (0.65 V). However, the higher *J_sc_* (12.5 mA cm^−2^) and *FF* (54%) presented in PBDT‐TQ: PC_70_BM‐based OSC can be attributed to the fast and balanced hole/electron mobility in the active layer induced by forming a suitable π–π conjugated plane in PBDT‐TQ. Interestingly, after further introducing the “F” atom into the Qx unit, Chen et al. reported a novel polymer donor PBDT‐TFQ (2012)^[^ ^202^ ^]^; Putri and co‐workers prepared PEhB‐FQx (2018) and PEhB‐FQxCF3 (2018).^[^ ^203^ ^]^ After applying for OSC devices, PBDT‐TFQ: PC_71_BM‐based, PEhB‐FQx: PC_71_BM‐based and PEhB‐FQxCF3: PC_71_BM‐based OSCs obtained the *V_oc_* of 0.76, 0.68 and 0.88 V; *J_sc_* of 17.9, 11.1 and 12.2 mA cm^−2^, *FF* of 57.6%, 63.5% and 57.8%; and *PCE* of 7.8%, 4.68% and 7.26%, respectively. Compared with the P(BDT‐DTQx): PC_71_BM‐based OSC, Qx unit with a strong electronegative significantly decreases the HOMO energy level of P(BDT‐DTQx) and accelerates the hole/electron mobility in the active layer, thereby P(BDT‐DTQx): PC_71_BM‐based OSC obtains a higher *V_oc_* and *J_sc_*. Moreover, compared with PEhB‐FQx, the appropriate fluorination degree also endows PEhB‐FQxCF3‐based OSC with a better photovoltaic performance. In addition, based on the reported results of PBDT‐TFQ, Tseng et al. designed PQEH (2014) by introducing the alkyl chain 2‐ethylhexyl to replace the original substitution of BDT unit.^[^ ^204^ ^]^ Although the optimized miscibility of donor increases the *V_oc_* from 0.76 to 0.78 V as well as *FF* from 57.6% to 64.3% in OSC devices, the decreased *J_sc_* can be attributed to the weakened electron‐donating of BDT unit.

In addition, based on the polymer backbone BDT‐T‐Qx‐T, the plenty of research focus on introducing thiophene‐based side chains into the BDT unit, Bolognesi et al. first reported P(1) (2013) by introducing side chains 5‐(2‐butyloctyl)−5′‐methyl‐2,2′‐bithiophene and 1‐methyl‐3‐(octyloxy)benzene into the BDT and Qx units in turn.^[^ ^205^ ^]^ Subsequently, based on the reported results of P(1), PBQ‐1 (2013) and PBQ‐2 (2013) were synthesized by Liu et al^[^ ^206^ ^]^; PTF1 (2016) and PTF2 (2016) were explored by Singh and co‐workers.^[^ ^200^ ^]^ After blending with the acceptor PC_71_BM, P(1): PC_71_BM‐based, PBQ‐1: PC_71_BM‐based, PBQ‐2: PC_71_BM‐based, PTF1: PC_71_BM‐based and PTF2: PC_71_BM‐based OSCs obtained the *V_oc_* of 0.74, 0.63, 0.75, 0.72 and 0.72 V; *J_sc_* of 9.5, 12.39, 12.96, 4.97 and 8.70 mA cm^−2^; *FF* of 53%, 69.81%, 63.14%, 29% and 34%; and *PCE* of 3.7%, 5.40%, 6.13%, 1.04% and 2.13%, respectively. Consistent with the above‐conclusions, P(1), PTF1 and PTF2 with a wider π–π conjugated plane enhance the *V_oc_* in corresponding OSC devices. Meanwhile, PBQ‐2 with the substitution of 2‐(2‐ethylhexyl)−3‐fluoro‐5‐methylthiophene also improves the *V_oc_*, *J_sc_* and *FF* in PBQ‐2: PC_71_BM‐based OSC. Furthermore, PTF2‐based OSC with a deeper degree of fluorination also achieved a better photoelectric performance than PTF1‐based OSC, which deserves to further attention. In addition, to further exploring the effect of fluorinated Qx unit in polymer skeleton BDT‐T‐Qx‐T, Bolognesi et al. first reported P(2) (2013) in 2013;^[^ ^205^ ^]^ PFQBDT‐TR1 (2014) and PFQBDT‐TR2 (2014) were synthesized by Tessarolo et al^[^ ^207^ ^]^; PBQ‐3 (2015) and PBQ‐4 (2015) were designed by Liu et al^[^ ^206^ ^]^; PBDTTFTQ‐EH (2015), PBDTTFTQ‐DO (2015), PBDTTFTTQ‐EH (2015) and PBDTTFTTQ‐DO (2015) with different types of thiophene‐based and benzene‐based side chains were reported by Wu et al^[^ ^208^ ^]^; Wang and co‐workers fabricated PBDT‐DFQX‐TP (2015) and PBDT‐DFQX‐TT (2015).^[^ ^209^ ^]^ Moreover, a series of novel polymer donors PffQx‐T (2016);^[^ ^210^ ^]^ HFQx‐BDT (2017);^[^ ^211^ ^]^ OFQx‐T (2017) and EOFQx‐T (2017);^[^ ^212^ ^]^ PBDT‐DFQX‐TTSEH (2017) and PBDT‐DFQX‐TTSCS (2017);^[^ ^213^ ^]^ PThB‐FQxCF3 (2018);^[^ ^203^ ^]^ WF3 (2019), WF3S (2019) and WF3F (2019);^[^ ^214^ ^]^ TTFQx‐T1 (2019);^[^ ^215^ ^]^ PBQ10 (2020);^[^ ^216^ ^]^ PBQ5 (2021) and PBQ6 (2021)^[^ ^217^ ^]^ were also successfully synthesized. Among these donors, under the synergistic optimization by introducing “F” atoms and side chains with a suitable π‐π conjugated, the *V_oc_* in corresponding donor: acceptor‐based OSCs stay at a high level, especially for P(2): PC_61_BM‐based (0.90 V), PFQBDT‐TR2: PC_61_BM‐based (0.96 V), PBQ‐4: PC_71_BM‐based (0.90 V), HFQx‐BDT: ITIC‐based (0.92 V) and EHFQx‐T: PC_71_BM‐based (0.91 V) OSCs. It is also worth noting that PBQ5: Y6‐based and PBQ6: Y6‐based OSCs exhibited a promising photoelectric performance, with the *V_oc_* of 0.839 and 0.845 V, *J_sc_* of 25.81 and 26.33 mA cm^−2^, *FF* of 70.23% and 77.47% and *PCE* of 15.22% and 17.34%, respectively. Although the reduced conjugated plane slightly decreases the *V_oc_* in PBQ5: Y6‐based and PBQ6: Y6‐based OSCs, the simpler molecular structure as well as the substitution of solubilized alkyl chain 2‐ethylhexyl both significantly increased the miscibility of donor, thereby forming a more complemental absorption in the blend film. Furthermore, compared with PBQ5, the introduction of alkyloxy side chain with a higher electron‐donating in PBQ10 also endows PBQ10: Y6‐based OSC with a higher *V_oc_* (0.85 V) and *FF* (74.6%). However, the better photoelectric performance presented by PBQ6: Y6‐based OSC reminds us that synthesizing an ideal polymer donor should not only be focusing on the regulation of substitutions. In 2022, based on the Qx unit with a single “F” and “CN” groups, Sagita et al. reported a series of polymer donors PB‐FQxF (2022), PBF‐FQxF (2022), PB‐FQxCN (2022) and PBF‐FQxCN (2022) by further introducing thiophene‐based and benzene‐based side chains into the BDT and Qx units.^[^ ^218^ ^]^ In 2023, PB‐CNQ (2023), PBCl‐CNQ (2023), PB‐CNQCl (2023) and PBCl‐CNQCl (2023) were also prepared by Lee and co‐workers.^[^ ^219^ ^]^ Notably, compared with EHFQx‐T: PC_71_BM‐based OSC, the decreased fluorination degree makes such polymer donor: Y6 based OSCs with a lower *V_oc_* (<0.85 V), while *J_sc_* significantly enhanced. Among them, the highest *J_sc_* (29.83 mA cm^−2^) exhibited in PB‐CNQ: Y6‐based OSC can be attributed to the more complemental absorption in the active layer. Moreover, benefiting “CN” group with a stronger electron‐withdrawing than “F” atom, PB‐FQxCN: Y6‐based and PBF‐FQxCN: Y6‐based OSCs obtained a higher *V_oc_* of 0.68 and 0.76 V, *J_sc_* of 25.15 and 24.19 mA cm^−2^, and *FF* of 63.84% and 62.59% than PB‐FQxF: Y6‐based and PBF‐FQxF: Y6‐based OSCs. Indeed, Qx unit with a stronger electronegative in the main‐chain BDT‐T‐Qx‐T not only effectively optimizes the HOMO energy level of donor, but also promotes the hole/electron mobility in the active layer. Among them, the fast and balanced hole/electron of PB‐FQxCN: Y6‐based (3.45 × 10^−5^/2.28 × 10^−5^ cm^2^ V^−1^ s^−1^) and PBF‐FQxCN: Y6‐based (2.88 × 10^−5^/2.97 × 10^−5^ cm^2^ V^−1^ s^−1^) blend films further prove above‐conclusions. As for PB‐CNQ: Y6‐based, PBCl‐CNQ: Y6‐based, PB‐CNQCl: Y6‐based and PBCl‐CNQCl: Y6‐based OSCs, the introduction of “CN” atoms also endows them with a more ideal photovoltaic performance, with the *PCE* of 11.19%, 12.80%, 13.98% and 9.84%, in that order. Interestingly, after adjusting the substitution sites of “Cl” atoms, Qx unit with synergistic effect of “F” and “Cl” atoms in PB‐CNQCl endows PBCNQCl: Y6‐based OSC with a better photoelectric performance than PBCl‐CNQ: Y6‐based OSC. Therefore, by further enhancing the electronegativity of acceptor unit is also an effective molecular design strategy to improve the *J_sc_* and *FF* in OSC devices. In addition, Fan et al. prepared P2 (2016) by introducing the side chain (2‐hexyldecyl) (*p*‐tolyl) sulphane into the BDT unit as well as 1‐methyl‐3‐(octyloxy) benzene into the Qx unit, respectively.^[^ ^151^ ^]^ Similarly, Aslan et al. synthesized P1 (2022) by introducing selenophene‐based and benzene‐based side chains into the BDT and Qx unit in turn,^[^ ^201^ ^]^ designing based on the theory that “Se”‐based materials with strong interaction between “Se” and “Se” molecules not only extends the π‐orbitals but also promotes hole/electron mobility.^[^ ^84^ ^]^ After blending with acceptors, P2 (2016): PC_61_BM‐based and P1 (2022): PC_71_BM‐based OSCs obtained the *V_oc_* of 0.81 and 0.80 V, *J_sc_* of 7.72 and 8.39 mA cm^−2^, *FF* of 68.79% and 60.8% and *PCE* of 4.28% and 4.10%, respectively. Consistent with the above‐conclusion, although the broaden π–π conjugated plane endows such OSCs with a high *V_oc_*, how to simultaneously improve *J_sc_* and *FF* is still a tough challenge. In order to break up this dilemma, Wang and co‐workers synthesized PBDT‐DFQX‐PP (2015) and PBDT‐DFQX‐PT (2015), which were designed based on the fluorinated Qx unit.^[^ ^209^ ^]^ Similarly, Yuan et al. also prepared PffQx‐m‐PO (2016), PffQx‐PO (2016) and PffQx‐PS (2016).^[^ ^220^ ^]^ Benefiting the substitution of “F” atom enhances the electron‐withdrawing of Qx unit, the *V_oc_* in such polymer donor: PC_71_BM based OSCs were significantly increased, meanwhile, the faster and more balanced hole/electron mobility also promotes the incensement of *J_sc_*. As for PBDTPH‐DTQx (2016) and PBDTPHO‐DTQx (2016), Liu et al. introduced 2‐ethylhexyl and benzene‐based side chains into the BDT unit.^[^ ^221^ ^]^ Compared with P2 (2016): PC_61_BM‐based OSC, the reduced π–π conjugated plane of PBDTPH‐DTQx and PBDTPHO‐DTQx decreases the *V_oc_* in PBDTPH‐DTQx: PC_71_BM‐based OSC (0.70 V) and PBDTPHO‐DTQx: PC_71_BM‐based OSC (0.75 V), while solubilized alkyl chain 2‐ethylhexyl endows such OSCs with a more complemental absorption after blending with acceptors, thus effectively enhancing the *J_sc_* in OSC devices. However, *FF* failed to be improved.

Furthermore, based on the breakthrough achieved by the main‐chain engineering BDT‐T‐Qx‐T, a series of novel polymer backbone (e.g. BDT‐T‐NTDz‐T, BDT‐T‐DBPz‐T and BDT‐T‐PTPz‐T) were also successfully fabricated. In 2013, Keshtov and co‐workers reported P (2013) based on the main chain BDT‐T‐DTQx‐T.^[^ ^222^ ^]^ Subsequently, Ratha et al. synthesized P(BDT‐ThNTTh) (2019) based on the polymer backbone BDT‐T‐NTDz‐T^[^ ^89^ ^]^; PBDT‐DTDBPz (2013),^[^ ^96^ ^]^ P2 (2015)^[^ ^223^ ^]^ and P3 (2015)^[^ ^223^ ^]^ were designed based on the polymer skeleton BDT‐T‐DBPz‐T; PTTPPz‐BDT (2014) and PTTPPz‐BDTT (2014) were prepared based on the main chain BDT‐T‐PTPz‐T^[^ ^224^ ^]^; Liu et al.^[^ ^225^ ^]^ and Yu et al.^[^ ^226^ ^]^ also innovatively constructed the polymer backbone BDT‐T‐QxQx‐T and then synthesized a series of polymer donors P1 (2017), P2 (2017), P3 (2017), PBDT‐NQx (2017) and PBDTS‐NQx (2017). Compared with the acceptor unit naphtho [2, 3‐*c*] [1, 2, 5] thiadiazole (NTDz), the introducing of “N” atoms further increase the electron‐withdrawing of [1, 2, 5] thiadiazolo [3, 4‐*g*] quinoxaline (DTQx) unit, which endows P: PC_71_BM‐based OSC with a higher *V_oc_* (0.72 V) and *J_sc_* (11.47 mA cm^−2^) than P(BDT‐ThNTTh): PC_71_BM‐based OSC. Furthermore, it is also worth noting that the excessively extended conjugated plane of PBDT‐DTDBPz, P2 (2015), P3 (2015), PTPPz‐BDT (2014) and PTTPPz‐BDTT disperses the electron density in polymer backbone, which leads to the decreased *J_sc_* in corresponding OSCs. As for PBDT‐NQx: ITIC‐based and PBDTS‐NQx: PC_71_BM‐based OSCs, compared with P1 (2017): PC_71_BM‐based, P2 (2017): PC_71_BM‐based and P3: PC_71_BM‐based OSCs, the significantly enhanced *V_oc_*, *J_sc_* and *FF* verifying again that an efficient polymer donor not only need to form a wide conjugated plane, but also retain a fast and balanced hole/electron mobility.

Notably, based on the characterization of blend film morphology and device stability, PBDT‐TTDTQx (399°C), PBDT‐DFQX‐TT (402°C), OFQx‐T (431°C), EHFQx‐T (405°C) and PDBT‐NQx (413°C) presented a high *Td_5%_*. Meanwhile, after 1400 h test for storage, only experienced a 40% *PCE* drop experienced by PDPPDTT: PC_71_BM‐based OSC in ambient conditions, demonstrating the promising device stability. In addition, compared with PBDTTFTTQ‐EH: PC_71_BM‐based (1.76 nm) and PBDTTFTTQ‐DO: PC_71_BM‐based (0.73 nm) blend films, the decreased *RMS* effectively enhances the *FF* of PBDTTFTQ‐DO: PC_71_BM‐based (0.64 nm) OSC, which can be attributed to the optimized hole/electron mobility induced by the suitable substitution. Interestingly, among OFQx‐T: PC_71_BM‐based (3.61 nm) and EHFQx‐T: PC_71_BM‐based (2.53 nm) blend films; WF3: PC_71_BM‐based (0.55 nm), WF3S: PC_71_BM‐based (0.94 nm) and WF3F: PC_71_BM‐based (0.81 nm) blend films; PB‐FQxCN: Y6‐based (1.01 nm) and PBF‐FQxCN: Y6‐based (1.20 nm) blend films as well as PffQx‐m‐Fpo: PC_71_BM‐based (2.99 nm) and PffQx‐PO: PC_71_BM‐based (2.13 nm) blend films, the formation of a suitable *RMS* endows OFQx‐T: PC_71_BM‐based (70.70%), WF3F: PC_71_BM‐based (64.5%), PB‐FQxCN: Y6‐based (63.94%) and PffQx‐PO: PC_71_BM‐based (65%) OSCs achieved a high *FF*, which further prove the above‐conclusion.

**FIGURE 16:**
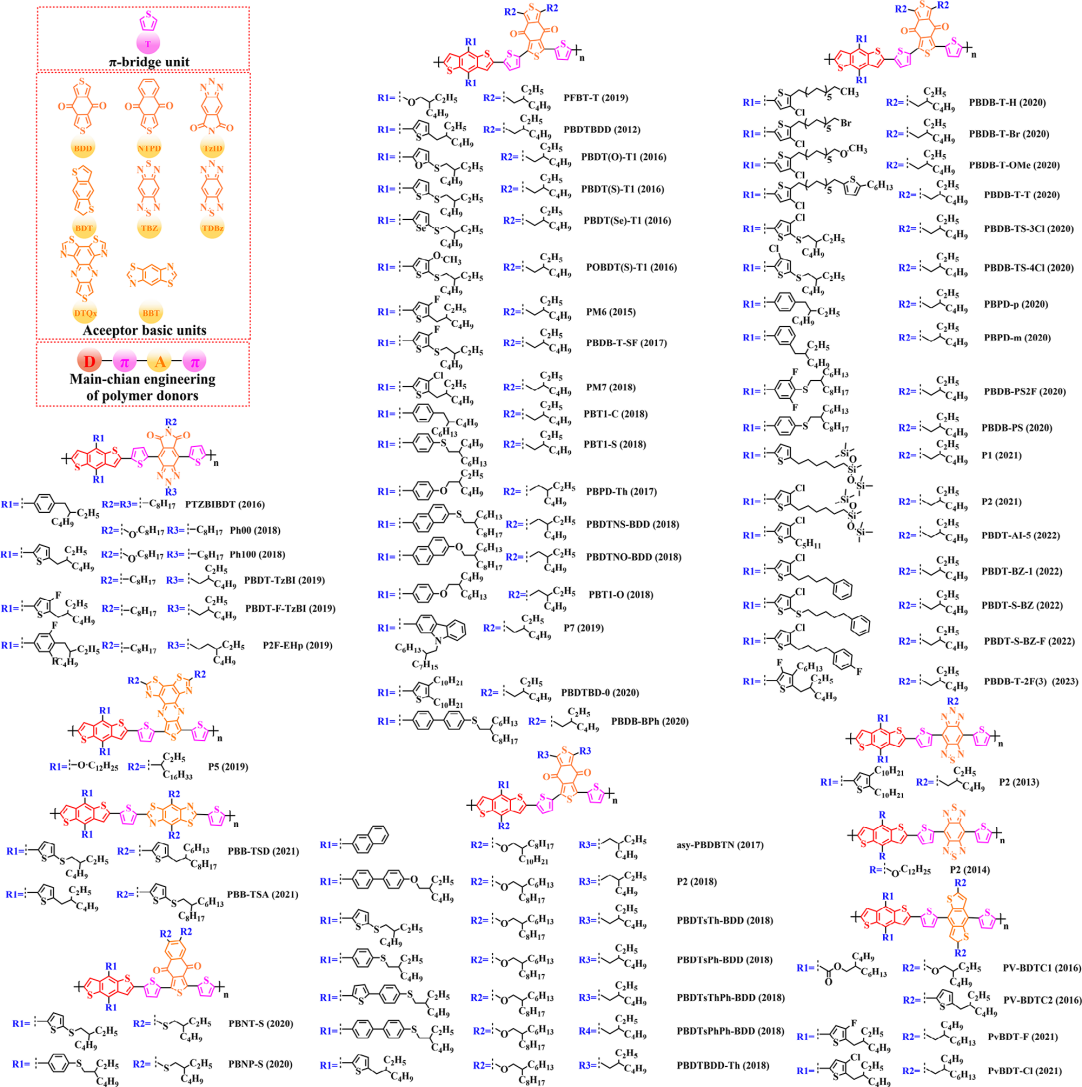
The detailed molecular structure of D‐π‐A‐π type polymer donors synthesized by introducing thiophene as π‐bridge unit, BDD and its derivatives as acceptor basic units.

As an efficient acceptor unit, 4*H*, 8*H*‐benzo [1, 2‐*c*: 4, 5‐*c*’] dithiophene‐4, 8‐dione (BDD) has attracted the plenty attention of researchers in recent years. Based on the main‐chain engineering BDT‐T‐BDD‐T, the famous polymer donor PM6 has already achieved a promising *PCE* of 17.97%, which greatly promotes the development of polymer donor. Furthermore, based on the breakthrough of BDD unit, a series of novel acceptor units were also constructed, such as naphtho [2, 3‐*c*] thiophene‐4, 9‐dione (NTPD), 5*H*, 7*H*‐2*λ* ^2^, 6*λ* ^2^‐[1, 2, 3] triazolo [4, 5‐*f*] isoindole‐5, 7‐dione (TzID), TDBz and dithiazolo [4, 5‐*f*: 5′, 4′‐*h*] thieno [3, 4‐*b*] quinoxaline (DTQx) et al. By introducing such efficient acceptor units, the detailed molecular structure (Figure 16) of representative polymer donors and photovoltaic parameters (Table 13) in corresponding OSCs were discussed as follows.

**TABLE 13:** Photovoltaic parameters of OSCs related to Figure 16.

Donor	Acceptor	D/A^A^	HOMO [eV]^B^	$E_{g}^{opt}$ [eV]^C^	*V_OC_* [V]	*J_SC_* [mA cm^−2^]	*FF* [%]	*PCE* [%]^D^	Ref.
PM6	Y6	1:1.2	−5.56	1.81	0.83	25.3	74.8	15.6	[5]
PM7(PBDB‐T‐2Cl)	IT‐4F	1:1	−5.40		0.86	21.80	76	14.0	[7]
P2	PC_61_BM		−5.04	1.00	0.38	1.71	43	0.4	[87]
P2^a^	PC_70_BM	1:1	−5.10	1.18	0.58	6.05	45	1.58	[163]
P7(2019)	PC_71_BM	1:1.5	−5.47	1.85	1.01	8.62	52.4	4.49	[168]
PBDTBD‐0^b^	IT‐4F	1:1.5	−5.54	1.82	0.88	7.30	30.93	1.71	[169]
PBDTBDD(PBDB‐T)	PC_61_BM	1:1	−5.23		0.86	10.68	72.27	6.67	[227]
PBDT(O)‐T1	PC_70_BM	1:1	−5.31	1.74	0.83	9.1	49.5	4.0	[228]
PBDT(S)‐T1	PC_70_BM	1:1	−5.52	1.81	0.89	12.2	67.7	7.3	[228]
PBDT(Se)‐T1	PC_70_BM	1:1	−5.53	1.80	0.91	12.6	71.7	8.4	[228]
PM6	L8‐BO		−5.49		0.87	25.66	80.5	17.97	[229]
POBDT(S)‐T1^c^	PC_70_BM	1:1			0.98	12.8	71.4	9.0	[230]
PBDB‐T‐SF	IT‐4F	1:1	−5.40	1.80	0.88	20.88	71.3	13.10	[231]
PBDB‐T‐H^d^, ^e^	Y6	1:1.2	−5.69	1.82	0.79	20.65	60	9.76	[232]
PBDB‐T‐Br^e^	Y6	1:1	−5.51	1.82	0.79	25.26	65	12.88	[232]
PBDB‐T‐OMe^d^, ^e^	Y6	1:1.2	−5.45	1.82	0.74	24.20	70	12.46	[232]
PBDB‐T‐T^e^	Y6	1:1.2	−5.58	1.82	0.79	24.42	64	12.27	[232]
PBDB‐TS‐3Cl	Y6		−5.61	1.82	0.86	23.45	64	12.91	[233]
PBDB‐TS‐4Cl	Y6		−5.61	1.82	0.87	22.06	65	12.46	[233]
P1^f^, ^g^	IT‐4F		−5.34	1.81	0.67	17.78	48.98	5.40	[234]
P2^f^, ^g^	IT‐4F		−5.50	1.84	0.84	17.43	69.78	10.25	[234]
PBDT‐AI‐5^f^	IT4F	1:1	−5.52	1.85	0.80	17.43	52.01	7.32	[235]
PBDT‐BZ‐1^h^, ^i^	IT‐4F	1:1	−5.66	1.86	0.84	19.10	70.06	11.11	[236]
PBDT‐S‐BZ^h^, ^i^	IT‐4F	1:1	−5.62	1.87	0.83	23.43	71.20	13.73	[236]
PBDT‐BZ‐F^h^, ^i^	IT‐4F	1:1	−5.53	1.88	0.82	20.03	59.46	9.64	[236]
PBDB‐T‐2F(3)	Y6‐HU		−5.64	1.90	0.92	17.05	60	9.26	[237]
PBPD‐Th^j^	ITIC	1:1.25	−5.42	1.91	1.01	18.1	56	10.5	[238]
PBT1‐O	ITCPTC	1:1	−5.30	1.82	0.91	12.6	64	7.3	[239]
PBT1‐C	ITCPTC	1:1	−5.43	1.84	0.94	17.0	78	12.5	[239]
PBT1‐S	ITPCTC	1:1	−5.45	1.83	0.94	15.2	66.7	9.5	[239]
PBDB‐BPh^k^	ITCPTC	1:1	−5.30	1.83	0.91	17.21	68.66	11.03	[240]
PBPD‐p	IT‐4F	1:1	−5.39	1.82	0.81	15.41	53.9	6.93	[241]
PBPD‐m	IT‐4F	1:1.5	−5.50	1.87	0.88	18.87	68.7	11.88	[241]
PBDB‐PS2F^l^	IT‐4F	1:1	−5.72	1.89	0.89	21.31	68.37	13.02	[242]
PBDB‐PS^l^	IT‐4F	1:1	−5.56	1.86	0.78	19.52	66.38	10.05	[242]
PBDTNS‐BDD	ITIC	1:1.5	−5.35	1.81	0.87	13.49	60.05	6.89	[243]
PBDTNO‐BDD	ITIC	1:1.5	−5.27	1.83	0.94	14.86	66.47	9.18	[243]
PFBT‐T	IT‐M	1:1	−5.26	1.79	0.878	10.88	59.04	5.45	[244]
asy‐PBDBTN^k^, ^m^	ITIC	1:1	−5.41		0.930	16.38	65.4	10.22	[245]
P2(2018)	ITIC	1:1	−5.46	1.82	0.873	17.60	65.37	9.88	[246]
PBDTsTh‐BDD^f^	ITIC	1:1	−5.41		0.954	14.02	53	6.96	[247]
PBDTsPh‐BDD^f^	ITIC	1:1	−5.37		0.917	14.65	53.8	6.99	[247]
PBDTsThPh‐BDD^f^	ITIC	1:1	−5.36		0.909	16.95	68.2	10.38	[247]
PBDTsPhPh‐BDD^f^	ITIC	1:1	−5.39		0.895	17.07	65.9	9.91	[247]
PBDTBDD‐Th^n^	ITIC	1:1	−5.38		0.949	15.51	68.1	10.02	[248]
PV‐BDTC1	PC_71_BM	1:2	−5.56	2.07	0.93	5.90	59.39	3.15	[249]
PV‐BDTC2^j^	PC_71_BM	1:2	−5.67	2.09	1.03	10.37	70.0	7.33	[249]
PvBDT‐F^o^	Y6‐T	1:1	−5.44	2.01	0.87	14.28	52.5	5.89	[250]
PvBDT‐Cl^o^	Y6‐T	1:1	−5.49	2.04	0.91	15.31	59.1	7.76	[250]
PTZBIBDT^p^	PC_71_BM	1:1	−5.34	1.81	0.87	13.50	71.09	8.35	[251]
Ph00^q^	ITIC	1:1.5	−5.26	1.84	0.85	15.92	54.82	7.30	[252]
Ph100^q^	ITIC	1:1.5	−5.33	1.90	0.90	15.23	55.58	7.48	[252]
PBDT‐TzBI	ITIC	1:1	−5.90	1.80	0.97	16.72	68	11.02	[253]
PBDT‐F‐TzBI^g^	ITIC	1:1	−5.97	1.81	0.93	18.10	72	12.12	[253]
P2F‐EHp^r^, ^s^	Y6	1:1.2	−5.46	1.82	0.81	26.66	71.98	15.58	[254]
P5(2019)	PC_71_BM		−5.11	1.13	0.74	15.74	66	7.81	[255]
PBNT‐S	Y6	1;1.5	−5.44		0.798	23.00	60.5	11.04	[256]
PBNP‐S	Y6	1:1.5	−5.44		0.810	25.50	69.4	14.25	[256]
PBB‐TSA^t^	IT‐4F	1:1.2	−5.29	1.98	0.82	19.1	68.4	10.4	[257]
PBB‐TSD^t^	IT‐4F	1:1.2	−5.54	1.96	0.89	21.9	74.5	14.3	[257]

^A^ Weight ratio.

^B^ The HOMO energy level of polymer donors.

^C^ Estimated from the absorption edge in film ($E_{g}^{opt}=1240/\lambda_{onset}$).

^D^ Average *PCE* of OSCs.

^a^ DIO and THF.

^b^ 2% DPE.

^c^ 0.7% DIO.

^d^ 0.5% CN.

^e^ 110°C annealing.

^f^ 100°Cannealing for 10 min.

^g^ 0.5% DPE.

^h^ 0.3% DPE.

^i^ 100°C annealing.

^j^ 3% DIO.

^k^ 0.5% DIO.

^l^ SVA treatment.

^m^ 150°C annealing for 10 min.

^n^ 160°C annealing for 10 min.

^o^ 0.3% DIO and 10% 2F‐IC.

^p^ 120°C annealing.

^q^ 0.5% DBE.

^r^ 1.0% DBE.

^s^ 110°C annealing for 10 min.

^t^ DIO.

In 2012, based on the main chain BDT‐T‐BDD‐T, PBDTBDD (2012) was first reported by Qian and co‐workers,^[^ ^227^ ^]^ synthesizing by introducing side chains 2‐(2‐ethylhexyl)−5‐methylthiophene and 2‐ethylhexyl into the BDT and BDD units in turn. After blending with the acceptor PC_61_BM, PBDTBDD: PC_61_BM‐based OSC obtained the *V_oc_* of 0.86 V, *J_sc_* of 10.68 mA cm^−2^, *FF* of 72.27% and *PCE* of 6.67%. Due to the formation of suitable π–π conjugated plane, PBDTBDD: PC_61_BM‐based OSC achieved a high *V_oc_*, meanwhile, the solubilized alkyl chain 2‐ethylhexyl also enhanced the miscibility of PBDTBDD, which also makes PBDTBDD: PC_61_BM‐based OSC to form a uniform and smooth surface morphology. Subsequently, based on the reported results of PBDTBDD, Xue and co‐workers reported three novel polymer donors PBDT(O)‐T1 (2016), PBDT(S)‐T1 (2016) and PBDT(Se)‐T1 (2016), which were designed by introducing side chains 2‐((2‐ethylhexyl) thio)−5‐methylfuran, 2‐((2‐ethylhexyl) thio)−5‐methylthiophene, and (2‐ethylhexyl) (5‐methylselenophen‐2‐yl) sulphane to replace the original substitutions into the BDT unit.^[^ ^228^ ^]^ Notably, compared with the alkyl side chain, alkylthio with an enhanced electron‐donating further increases the *J_sc_* in PBDT(S)‐T1: PC_70_BM‐based OSC (12.2 mA cm^−2^) and PBDT(Se)‐T1: PC_70_BM‐based OSC (12.6 mA cm^−2^). Furthermore, benefiting from the stronger interaction between selenophene units, PBDT(Se)‐T1: PC_70_BM‐based OSC also obtained a significantly enhanced *V_oc_* (0.91 V) and finally achieved the highest *PCE* (8.4%) among these OSCs. Afterward, to further explore the potential application of polymer skeleton BDT‐T‐BDD‐T with the substitution of thiophene‐based side chains into the BDT unit, PM6 (2015),^[^ ^229^ ^]^ POBDT(S)‐T1 (2016),^[^ ^230^ ^]^ PBDB‐T‐SF (2017),^[^ ^231^ ^]^ PM7 (2018)^[^ ^7^ ^]^ and PBDTBD‐0 (2020)^[^ ^169^ ^]^ were synthesized by different research teams, respectively. In 2020, Liu et al. considered to introduce end functional units (e.g. bromine (Br), alkoxy and alkyl‐thiophene groups) into the substitutions, preparing a series of novel polymer donors PBDB‐T‐H (2020), PBDB‐T‐Br (2020) and PBDB‐T‐OMe (2020).^[^ ^232^ ^]^ In the same year, Lee and Je et al. also synthesized PBDB‐TS‐3Cl (2020) and PBDB‐TS‐4Cl (2020) by adjusting the substituted active site of “Cl” atom into the side chains.^[^ ^233^ ^]^ In recent years, P1 (2021),^[^ ^234^ ^]^ P2 (2021),^[^ ^234^ ^]^ PBDT‐AI‐5 (2022),^[^ ^235^ ^]^ PBDT‐BZ‐1 (2022),^[^ ^236^ ^]^ PBDB‐S‐BZ (2022),^[^ ^236^ ^]^ PBDB‐S‐BZ‐F (2022)^[^ ^236^ ^]^ and PBDB‐T‐2F (3) (2023)^[^ ^237^ ^]^ were also successfully prepared. After applying for OSC devices, POBDT(S)‐T1 with the substitution of alkylthio and alkyloxy substituted thiophene side chains achieved the highest *V_oc_* (0.98 V) after applying for OSC devices, which can be attributed to the optimized HOMO energy level induced by the synergistic effect of alkoxy and alkylthio side chains. Interestingly, this phenomenon has also occurred in D‐A type polymer donors. Furthermore, PBDB‐T‐2F: Y6‐HU‐based OSC obtained a promising *V_oc_* (0.92 V); PM6: Y6‐based (15.6%) and PM6: L8‐BO‐based (17.97%) OSCs also achieved a promising *PCE*; the *V_oc_* in such OSCs fabricated based on the chlorinated polymer donors stay at a high level. These characterization results are enough to verifying that fluorination and chlorination are effective molecular design strategies. As for benzene‐based side chain substituted BDT unit into the main chain BDT‐T‐BDD‐T, Fan and co‐workers reported a novel polymer donor PBDB‐Th (2017).^[^ ^238^ ^]^ Similarly, PBT1‐O (2018), PBT1‐C (2018) and PBT1‐S (2018) were synthesized by Liu et al.^[^ ^239^ ^]^; PBDB‐BPh (2020) was designed by Li et al.^[^ ^240^ ^]^; PBPD‐p (2020) and PBPD‐m (2020) were prepared by Guo et al.^[^ ^241^ ^]^; PBDB‐PS2F (2020) and PBDB‐PS (2020) were also successfully constructed by Kang and co‐workers,^[^ ^242^ ^]^ respectively. Due to benzene‐based side chains effectively extend the π–π conjugated plane of such polymer donors, the optimized molecular stacking endows such polymer donors: acceptor‐based OSCs with a high *V_oc_*, especially for PBPD‐Th: ITIC‐based (1.01 V), PBT1‐O: ITCPTC‐based (0.91 V), PBT1‐C: ITCPTC‐based (0.91 V), PBT1‐S: ITCPTC‐based (0.94 V) and PBDB‐BPh: ITCPTC‐based (0.94 V) OSCs. Furthermore, compared with PBT1‐O: ITCPTC‐based and PBT1‐S: ITCOTC‐based OSCs, the solubilized alkyl chains 2‐butyloctyl and 2‐ethylhexyl synergistically enhances the miscibility of PBT1‐C, thus endowing PBT1‐C: ITCPTC‐based OSC with a better *FF* (78%) and *PCE* (12.5%). As for the PBDB‐PS2F, the substitution of fluorinated benzene‐based side chain further promotes the mobility of hole/electron in polymer backbone. Therefore, PBDB‐PS2F: IT‐4F‐based OSC obtained a significantly enhanced *J_sc_* (21.31 mA cm^−2^) and finally achieves the best *PCE* (13.02%). In addition, Huang et al. reported PBDTNS‐BDD (2018) and PBDTNO‐BDD (2018) by introducing naphthalene‐based side chains into the BDT unit^[^ ^243^ ^]^; PFBT‐T (2018)^[^ ^244^ ^]^ and P7 (2019)^[^ ^168^ ^]^ were synthesized by introducing alkyloxy and carbazole‐based side chains in turn. Consistent with the above reported results, the *V_oc_* in such polymer donors: acceptor‐based OSCs stay at a high level, whereas *J_sc_* and *FF* are low. Moreover, the plenty of studies have also focused on the main chain BDT‐T‐BDD‐T with asymmetric substitutions. In 2017, asy‐PBDBTN was first reported by Li and co‐workers,^[^ ^245^ ^]^ designing by introducing side chains naphthalene and alkyloxy into the BDT unit. Afterward, P2 (2018),^[^ ^246^ ^]^ PBDTsTh‐BDD (2018),^[^ ^247^ ^]^ PBDTsPh‐BDD (2018),^[^ ^247^ ^]^ PBDTsThPh‐BDT (2018),^[^ ^247^ ^]^ PBDTsPhPh‐BDD (2018)^[^ ^247^ ^]^ and PBDTBDD‐Th (2018)^[^ ^248^ ^]^ were also successfully prepared. Under the synergistic optimization of the donor's HOMO energy level and molecular stacking in the active layer by introducing thiophene‐based, benzene‐based, or naphthalene‐based side chains as well as alkylthio or alkyloxy groups, the *V_oc_* in these OSCs were stay at a high level. However, how to effectively improve the *J_sc_* and *FF* in OSC devices is still worth further attention.

In addition, with the emergence of derivative units based on the BDD unit, these novel main‐chain engineering have also greatly promoted the development of OSC. In 2013, Keshtov et al. first reported P2 (2013) based on the main chain BDT‐T‐TBz‐T.^[^ ^163^ ^]^ Subsequently, Dong et al. also reported P2 (2013) based on the polymer backbone BDT‐T‐TDBz‐T.^[^ ^87^ ^]^ In 2016, Tao and co‐workers innovatively constructed the main chain BDT‐T‐BDT‐T, preparing PV‐BDTC1 (2016) and PV‐BDTC2 (2016).^[^ ^249^ ^]^ Zhang and co‐workers also designed PvBDT‐F (2021) and PvBDT‐Cl (2021) based on the main chain BDT‐T‐BDT‐T.^[^ ^250^ ^]^ Furthermore, PTZBIBDT (2016),^[^ ^251^ ^]^ Ph00 (2018),^[^ ^252^ ^]^ Ph100 (2018),^[^ ^252^ ^]^ PBDT‐TzBI (2019),^[^ ^253^ ^]^ PBDT‐F‐TzBI (2019)^[^ ^253^ ^]^ and P2F‐EHp (2019)^[^ ^254^ ^]^ were synthesized based on the polymer backbone BDT‐T‐TzID‐T; P5 (2019) was prepared based on the polymer skeleton BDT‐T‐DTQx‐T^[^ ^255^ ^]^; PBNT‐S (2020) and PBNP‐S (2020) were constructed based on the main chain BDT‐T‐NTPD‐T^[^ ^256^ ^]^; PBB‐TSD (2021) and PBB‐TSA (2021) were also reported by Raji et al,^[^ ^257^ ^]^ designing based on the polymer backbone BDT‐T‐BBT‐T. Among these donors, PBNT‐S: Y6‐based (23.00 mA cm^−2^), PBNP‐S: Y6‐based (25.50 mA cm^−2^), P2F‐EHp: Y6‐based (26.66 mA cm^−2^) and PBB‐TSD: IT‐4F‐based (21.9 mA cm^−2^) OSCs achieved a significantly enhanced *J_sc_*. Meanwhile, with the improvement of *J_sc_*, such OSCs also obtained an increased *FF*, especially for PBB‐TSD: IT‐4F‐based OSC (74.5%). Indeed, compared with alkyl side chain, the functional group alkylthio with a stronger electron‐donating, not only promotes the hole/electron mobility in the active layer, but also but also makes corresponding blend films to form a more ideal fibrous bi‐continuous interpenetrating morphology, thereby endowing a high *FF* in corresponding OSC devices. Furthermore, compared with PTzBIBDT: PC_71_BM‐based OSC, benzene‐based side chains with two “F” atoms also effectively accelerate the hole/electron mobility in P2F‐EHp: Y6‐based OSC. Therefore, the more complemental absorption formed in P2F‐EHp: Y6‐blend film endows P2F‐EHp: Y6‐based OSC with a high *J_sc_* (26.66 mA cm^−2^) and *PCE* (15.58%). Furthermore, although the *V_oc_* in OSC devices can be realized by utilizing the molecular design strategies of fluorination, chlorination as well as forming a suitable π‐π conjugated planes in polymer donors, OSC devices with a high *V_oc_* are normally face the dilemma of low *J_sc_* and *FF*.

Among these donors, the high *Td_5%_* presented by PBDTBD‐0 (406°C), PBDT‐AI‐5 (383°C) and Ph100 (395°C) exhibiting an excellent stability for application. As for PM6: Y6‐based (0.97 nm) and PM7: IT‐4F‐based (2.00 nm) blend films, the suitable *RMS* exhibited by PM6: Y6‐based blend film endows corresponding OSC with a high *FF* (76%). Furthermore, compared with PBDT(O)‐T1: PC_70_BM‐based (1.84 nm) and PBDT(S)‐T1: PC_70_BM‐based (2.20 nm) blend films, the slightly increased *RMS* of PBDT(Se)‐T1: PC_70_BM‐based (2.55 nm) blend film endows corresponding OSC with a higher *FF*. Similarly, the more suitable *RMS* endows PBDB‐T‐OMe: Y6‐based (1.07 nm), PBDT‐S‐BZ: IT‐4F‐based (0.43 nm), PBPD‐m: IT‐4F‐based (1.53 nm), PBDT‐F‐TzBI: ITIC‐based (1.07 nm), PBNP‐S: Y6‐based (1.44 nm) and PBB‐TSD: IT‐4F‐based (0.94 nm) blend films with a higher *FF*, with the *FF* of 70%, 71.20%, 68.7%, 72%, 69.4%, and 74.5% in turn, which further prove the above‐conclusion.

**FIGURE 17:**
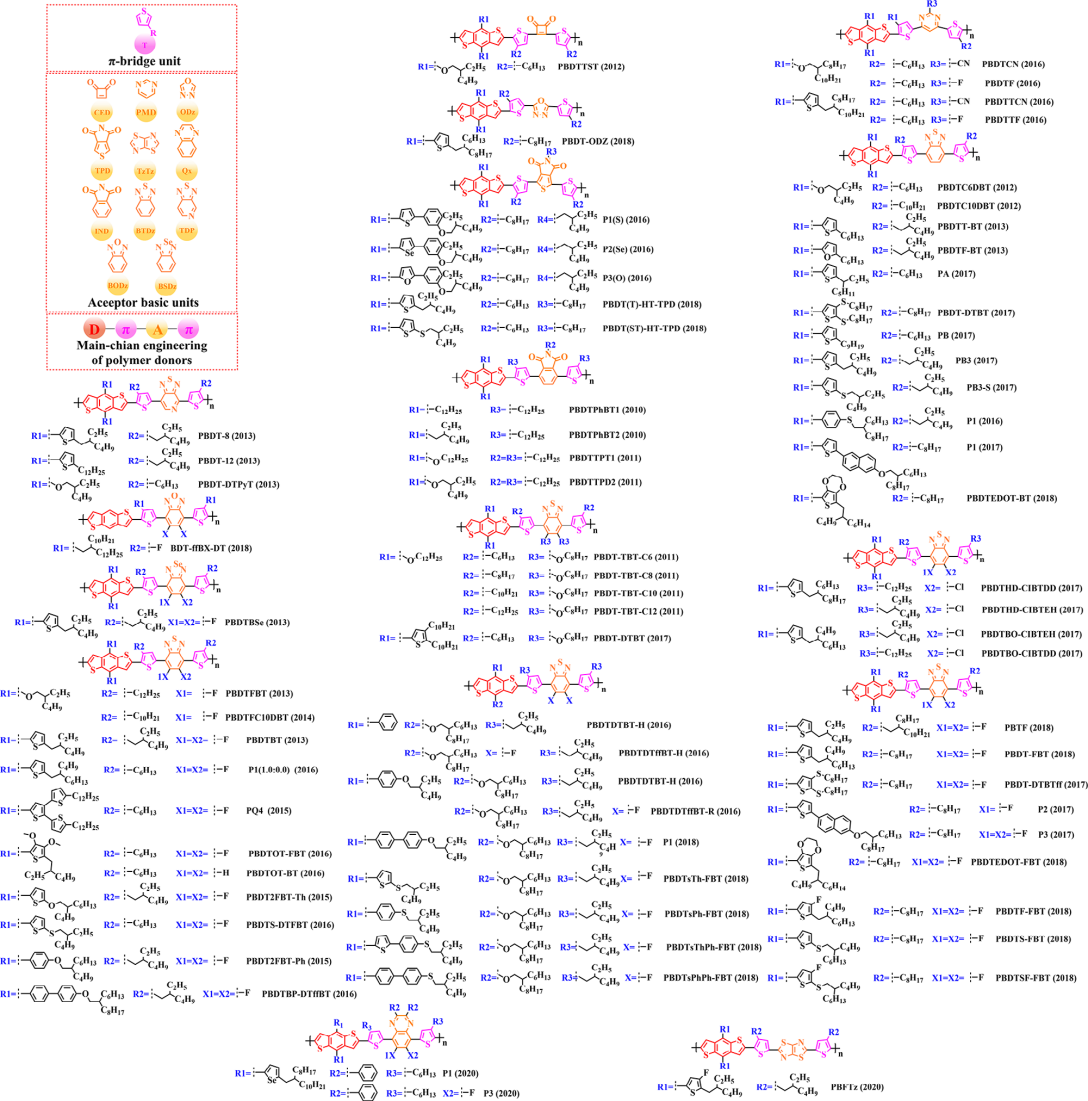
The detailed molecular structure of D‐π‐A‐π type polymer donors synthesized by introducing alkyl‐thiophene as π‐bridge unit, CED, PMD, ODz, TPD, Qx and other functional groups as acceptor basic units.

Indeed, thiophene, as a widely used π‐bridge unit, not only effectively extends the π–π conjugated plane of polymer donors, but also optimizes the hole/electron mobility in corresponding OSC devices. Therefore, compared with D‐A, D‐A‐A, or D‐D‐A type polymer donors, D‐π‐A‐π type polymer donors have achieved a great success, meanwhile, the emergence of a series of efficient polymer donors has also promoted the prosperity of OSC field. However, the extension of the conjugated plane also leads to the reduced miscibility of polymer donors in the active layer as well as dispersed electron density. In order to solve this problem, with the emergence of molecular strategy by introducing alkyl substituted thiophene as π‐bridge unit, the plenty of novel main‐chain engineering was also successfully constructed. Based on such molecular design strategy, the detailed molecular structure (Figure 17) of representative polymer donors and photoelectric parameters (Table 14) in corresponding OSCs were shown as follows.

**TABLE 14:** Photovoltaic parameters of OSCs related to Figure 17.

Donor	Acceptor	D/A^A^	HOMO [eV]^B^	$E_{g}^{opt}$ [eV]^C^	*V_OC_* [V]	*J_SC_* [mA cm^−2^]	*FF* [%]	*PCE* [%]^D^	Ref.
PBDT‐DTPyT	PCBM	1:3	−5.24	1.50	0.50	8.16	33	1.34	[30]
PBDT‐DTBTff^a^	PC_71_BM	1:1	−5.52	1.71	0.86	13.32	66.8	7.46	[57]
PBDT‐DTBT^b^	PC_71_BM	1:1	−5.60	1.65	0.91	11.69	61.2	6.33	[57]
PBDTDTBT‐H	PC_71_BM	1:1.5	−5.04	1.73	0.83	13.92	66.5	7.56	[59]
PBDTDTffBT‐H	PC_71_BM	1:1.5	−5.06	1.76	0.88	14.92	71.9	9.34	[59]
PBDTDTBT‐R	PC_71_BM	1:1	−5.01	1.72	0.84	13.64	71.7	8.12	[59]
PBDTDTffBT‐R	PC_71_BM	1:1.5	−5.03	1.75	0.88	13.98	73.9	9.01	[59]
PBDTS‐DTFBT	PC_70_BM	1:1	−5.47	1.69	0.83	8.27	46.42	3.19	[73]
PBDTS‐FBT	PC_70_BM	1:1	−5.49	1.73	0.80	6.65	30.30	1.42	[73]
PA^a^	PC_71_BM	1:2	−5.76	1.82	0.855	8.669	50.606	3.752	[80]
PB^a^	PC_71_BM	1:2	−5.66	1.81	0.769	6.900	45.551	2.416	[80]
PBDTTST^c^	PCBM	1:3	−5.41	1.57	0.85	3.38	39	1.12	[119]
PQ4^d^	PC_71_BM	1:1	−5.59	1.73	0.94	6.17	48.80	2.83	[136]
PB3	ITIC		−5.14	1.70	0.822	13.47	59.8	6.61	[137]
PB3‐S	ITIC		−5.24	1.71	0.873	16.93	56.0	8.28	[137]
PBDTOT‐FBT	PC_70_BM	1:2.5	−5.46	1.79	0.92	8.71	60.3	4.84	[149]
PBDTOT‐BT^c^	PC_70_BM	1:3	−5.25	1.56	0.88	7.60	36.9	2.47	[149]
P1^e^	PC_61_BM	1:3	−5.49	1.73	0.85	4.22	41.05	1.48	[151]
P1(1.0:0.0)	PC_71_BM		−5.32	1.73	0.76	12.57	63.4	5.87	[152]
PBDT‐TBT‐C6	PC_61_BM	1:3	−5.26	1.76	0.74	4.50	44	1.92	[154]
PBDT‐TBT‐C8	PC_61_BM	1:3	−5.29	1.77	0.70	7.19	52	2.88	[154]
PBDT‐TBT‐C10	PC_61_BM	1:3	−5.25	1.77	0.72	3.95	37	1.04	[154]
PBDT‐TBT‐C12	PC_71_BM	1:3	−5.23	1.75	0.71	8.60	51	3.15	[154]
PBDTBT	PC_70_BM	1:2	−5.34	1.74	0.90	9.30	46.9	3.93	[164]
PBDTBSe^c^	PC_70_BM	1:2	−5.30	1.66	0.81	5.36	50.6	2.20	[164]
P1(2018)	PC_71_BM	1:1.5	−5.41	1.70	0.838	14.35	70.27	8.29	[246]
PBDTsTh‐FBT^f^	PC_71_BM	1:1.5	−5.35		0.892	15.17	75.5	10.12	[247]
PBDTsPh‐FBT	PC_71_BM	1:1.5	−5.33		0.884	14.84	75	9.70	[247]
PBDTsThPh‐FBT	PC_71_BM	1:1.5	−5.32		0.876	14.98	70.9	9.14	[247]
PBDTsPhPh‐FBT	PC_71_BM	1:1.5	−5.32		0.868	14.05	70.3	8.29	[247]
PBDTCN^c^	PC_71_BM	1:1.5	−5.39	2.04	0.74	2.37	36	0.63	[258]
PBDTF^c^	PC_71_BM	1:1.5	−5.14	2.07	0.64	2.25	35	0.50	[258]
PBDTTCN^c^	PC_71_BM	1:1.5	−5.67	2.01	0.86	3.14	40	1.09	[258]
PBDTTF^c^	PC_71_BM	1:1.5	−5.58	2.06	0.82	3.28	39	1.05	[258]
PBDT‐ODZ^g^	ITIC‐Th	1:1	−5.68	2.12	1.06	17.10	68.1	12.07	[259]
P1(S)	PC_71_BM	1:2	−5.44	1.85	0.92	8.90	41.11	3.37	[260]
P2(Se)	PC_71_BM	1:2	−5.48	1.81	0.96	7.84	46.69	3.53	[260]
P3(O)	PC_71_BM	1:2	−5.35	1.77	0.80	5.29	36.33	1.54	[260]
PBDT(T)‐HT‐TPD	P(NDI2OD‐Se)	1.5:1	−5.30	1.85	0.95	11.97	52.51	5.63	[261]
PBDT(ST)‐HT‐TPD	P(NDI2HD‐Se)	1.5:1	−5.32	1.80	0.95	8.17	59.54	4.45	[261]
PBFTz^h^	Y6	1:1.25	−5.63	1.94	0.91	13	59	6.7	[262]
P1^i^	PC_71_BM	1:3	−5.35	1.67	0.79	7.29	41	2.36	[263]
P3^j^	PC_71_BM	1:4	−5.38	1.64	0.77	7.59	41	2.45	[263]
PBDTC6DBT	PC_61_BM	1:2	−5.20		0.63	6.18	48	1.86	[264]
PBDTC10DBT	PC_61_BM	1:2	−5.21		0.53	3.50	54	0.99	[264]
PBDTT‐BT	PC_71_BM	1:1	−5.26	1.67	0.88	5.83	36.0	1.85	[265]
PBDTF‐BT	PC_71_BM	1:1	−5.24	1.70	0.85	8.41	40.3	2.88	[265]
PBDT‐DTBT	PC_71_BM	1:1	−5.26	1.73	1.00	5.80	34.6	2.11	[266]
P1	P(NDI2HD‐T2)	2.5:1	−5.14	1.60	0.63	10.51	40	2.60	[267]
P2	P(NDI2HD‐T2)	2.5:1	−5.20	1.60	0.72	14.03	50	4.97	[267]
P3	P(NDI2HD‐T2)	2.5:1	−5.26	1.65	0.79	15.23	53	6.29	[267]
PBDTEDOT‐FBT^k^	PC_71_BM	1:1.5	−5.32		0.88	16.01	72.6	9.78	[268]
PBDTEDOT‐BT^l^	PC_71_BM	1:1.5	−5.22		0.80	15.20	63.1	7.21	[268]
PBDTFBT	PC_71_BM	1:2	−5.30	1.64	0.72	9.31	60.0	4.0	[269]
PBDTFC10DBT	PC_61_BM	1:4	−5.42	1.68	0.62	3.31	58	1.19	[270]
PBDTHD‐ClBTDD	PC_71_BM	1:1.5	−5.53	1.68	0.76	16.65	70.82	9.01	[271]
PBDTBO‐ClBTDD	PC_71_BM	1:1.5	−5.47	1.70	0.68	11.20	60.54	4.72	[271]
PBDTHD‐ClBTEH	PC_71_BM	1:1.5	−5.53	1.71	0.79	13.02	62.11	6.51	[271]
PBDTBO‐ClBTEH	PC_71_BM	1:1.5	−5.50	1.69	0.78	10.71	62.45	5.29	[271]
PBDT2FBT‐Th^m^	PC_71_BM	1:1.2	−5.12	1.70	0.82	8.61	42.6	2.98	[272]
PBDT2FBT‐Ph^m^	PC_71_BM	1:1.2	−5.23	1.72	0.83	11.33	66.3	6.02	[272]
PBDTBP‐DTffBT^n^	PC_71_BM	1:1	−5.46	1.71	0.85	12.72	62.14	6.58	[273]
PBDT‐FBT^f^	PC_71_BM	1:1	−5.14	1.69	0.79	15.41	68.2	7.83	[274]
PBDTF‐FBT^f^	PC_71_BM	1:1	−5.34	1.71	0.86	16.47	68.7	9.31	[274]
PBDTS‐FBT^f^	PC_71_BM	1:1	−5.20	1.70	0.84	16.28	68.3	8.82	[274]
PBDTSF‐FBT^f^	ITIC	1:1	−5.41	1.71	1.03	17.09	66.3	11.12	[274]
PBTF^c^	PC_71_BM	1:1.5	−5.53	1.68	0.96	8.00	76.1	4.81	[275]
PBDTTPT1	PC_71_BM	1:1	−5.30	1.78	0.89	3.04	62	1.68	[276]
PBDTTPD2	PC_71_BM	1:1	−5.35	1.82	0.92	3.93	57	2.05	[276]
PBPT‐8^c^, ^o^	PC_61_BM		−5.41	1.56	0.75	14.07	45.9	4.84	[277]
PBPT‐12^c^, ^o^	PC_61_BM		−5.44	1.53	0.75	12.56	54.2	5.11	[277]
BDT‐ffBX‐DT	PDI4		−5.58		1.14	10.6	63	7.3	[278]
PBDTPhBT1	PC_71_BM	1:1	−5.35	1.98	0.90	2.40	50	1.08	[333]
PBDTPhBT2	PC_71_BM	1:1	−5.32	1.98	0.93	2.96	56	1.54	[333]

^A^ Weight ratio.

^B^ The HOMO energy level of polymer donors.

^C^ Estimated from the absorption edge in film ($E_{g}^{opt}=1240/\lambda_{onset}$).

^D^ Average *PCE* of OSCs.

^a^ 3% DIO.

^b^ 3% CN.

^c^ 5% DIO.

^d^ 90°C annealing for 10 min.

^e^ 100°C annealing.

^f^ 0.5% DIO.

^g^ 4% Cul.

^h^ 0.75% 1‐CN.

^i^ 2% o‐DCB.

^j^ 3% o‐DCB.

^k^ 2.5% DIO.

^l^ 2% DIO.

^m^ 1.5% DIO.

^n^ 110°C annealing.

^o^ inverted device.

In 2012, based on the main chain BDT‐TR‐CED‐TR, Yang and co‐workers first reported PBDTTST (2012) by further introducing side chains hexyl and 3‐(methoxymethyl) heptane into the cyclobut‐3‐ene‐1,2‐dione (CED) and BDT units in turn.^[^ ^119^ ^]^ Afterward, PBDTCN (2016), PBDTF (2016), PBDTTCN (2016) and PBDTTF (2016) were synthesized by Kim et al^[^ ^258^ ^]^; PBDT‐ODZ (2018) was also prepared by Xu et al.^[^ ^259^ ^]^ Notably, PBDT‐ODz: ITIC‐Th‐based OSC achieves a promising *V_oc_* of 1.06 V, *J_sc_* of 17.10 mA cm^−2^, *FF* of 68.1% and *PCE* of 12.07%. Compared with the acceptor units CED and pyrimidine (PMD), 1,3,4‐oxadiazole (ODz) presented a more ideal electron‐withdrawing, which effectively promotes the hole/electron mobility in polymer backbone. Therefore, the simple and efficient molecular structure also endows PBDT‐ODZ: ITIC‐Th‐based OSC with a better photoelectric performance.

Furthermore, based on the main chain BDT‐TR‐TPD‐TR, P1(s) (2016), P2(Se) (2016), P3(O) (2016) were reported by Chakravarthi et al.^[^ ^260^ ^]^ Similarly, Zhang et al. synthesized PBDT(f)‐HT‐TPD (2018) and PBDT(ST)‐HT‐TPD (2018)^[^ ^261^ ^]^; Wu et al. prepared PBFTz (2020), designing based on the polymer backbone BDT‐TR‐TzTz‐TR.^[^ ^262^ ^]^ As for P1 (2020) and P3 (2020),^[^ ^263^ ^]^ which were constructed based on the polymer skeleton BDT‐TR‐Qx‐TR. Interestingly, the extended π–π conjugated plane and the substitution of alkylthio or alkyloxy effectively optimize the *V_oc_* in such polymer donor: acceptor‐based OSCs, especially for P2(Se): PC_71_BM‐based (0.96 V), PBDT(T)‐HT‐TPD: P(NDI2OD‐Se)‐based (0.95 V) and PBDT(ST)‐HT‐TPD: P(NDI2HD‐Se)‐based (0.95 V) OSCs.

In addition, based on the famous BTDz acceptor unit and its derivatives, Qu et al. constructed the main chain BDT‐TR‐BTDz‐TR, synthesizing two novel polymer donors PBDTC6DBT (2012) and PBDTC10DBT (2012) by introducing side chains 3‐(methoxymethyl)heptane and hexyl into the BDT and π‐bridge units in turn.^[^ ^264^ ^]^ Subsequently, based on the reported results of PBDTC6DBT and PBDTC10DBT, a series of polymer donors PBDTT‐BT (2013),^[^ ^265^ ^]^ PBDTF‐BT (2013),^[^ ^265^ ^]^ P1 (2016),^[^ ^151^ ^]^ PBDT‐DTBT (2017),^[^ ^266^ ^]^ PA (2017),^[^ ^80^ ^]^ PB (2017),^[^ ^80^ ^]^ PB3 (2017),^[^ ^137^ ^]^ PB3‐S (2017)^[^ ^137^ ^]^ and P1 (2017)^[^ ^267^ ^]^ were also prepared by introducing thiophene‐based, furan‐based and benzene‐based side chains into the BDT as well as solubilized alkyl chain into the π‐bridge units, respectively. Moreover, Feng and co‐workers innovatively designed PBDTEDOT‐BT (2018) by introducing the 2,3‐dihydrothieno [3, 4‐*b*] [1, 4] dioxine based side chain into the BDT unit.^[^ ^268^ ^]^ Regrettably, after applying for the OSC devices, low *J_sc_* and *FF* seriously hindered the breakthrough of *PCE*. In addition, Li et al. try to further introducing two alkyloxy side chains into the BTDz unit, reporting a series of novel polymer donors PBDT‐TVT‐C6 (2011), PBDT‐TBT‐C8 (2011), PBDT‐TBT‐C10 (2011) and PBDT‐TBT‐C12 (2011).^[^ ^154^ ^]^ Similarly, Yu and co‐workers also prepared PBDT‐DTBT (2017).^[^ ^57^ ^]^ Among these donors, the substitution of alkyloxy with strong electron‐donating decreases the electronegative of BTDz unit to a certain extent, thus the hole/electron mobility has not been optimized. Subsequently, the plenty of research have begun to further enhance the electron‐withdrawing of BTDz unit in the polymer backbone BDT‐TR‐BTDz‐TR. In 2013, Xiao and co‐workers. based on this polymer skeleton, by introducing single “F” atom into the BTDz unit, successfully synthesized PBDTFBT (2013).^[^ ^269^ ^]^ Afterward, based on the reported results of PBDTFBT, Liu et al.^[^ ^270^ ^]^ and Kranthiraja et al.^[^ ^267^ ^]^ also prepared PBDTFC10DBT (2014) and P2 (2017) in turn. Furthermore, PBDTHD‐CIBTDD (2017), PBDTBO‐CIBTDD (2017), PBDTHD‐CIBTEH (2017) and PBDTBO‐CIBTEH (2017) were also designed by Mo et al.^[^ ^271^ ^]^, which were constructed by further introducing single “Cl” atom into the BTDz unit. Notably, compared to “F” atom, “Cl” atom with a stronger electronegativity further enhances the electron‐withdrawing of BTDz unit, thereby hole/electron mobility in the polymer backbone was accelerated. Furthermore, the higher *J_sc_* and *FF* normally exhibited in OSCs fabricated based on chlorinated polymer donors than fluorinated donor materials are also worth to further attention. Subsequently, considering further enhancing the electronegativity of acceptor unit in the main‐chain engineering is an effective molecular design strategy, Shen et al.^[^ ^164^ ^]^ and Liu et al.^[^ ^136^ ^]^ based on the polymer backbone BDT‐TR‐BTDz‐TR, reported PBTFBT (2013) and PB4 (2015) by introducing two “F” atoms into the BTDz unit. Afterward, PBDT2FBT‐Th (2015),^[^ ^272^ ^]^ PBDT2FBT‐Ph (2015),^[^ ^272^ ^]^ PBDTS‐DTFBT (2016),^[^ ^73^ ^]^ PBDTOT‐FBT (2016),^[^ ^73^ ^]^ PBDTOT‐BT (2016),^[^ ^149^ ^]^ PBDTBP‐DTffBT (2016),^[^ ^273^ ^]^ P1(1.0: 0.0) (2016),^[^ ^152^ ^]^ PBDT‐DTBTff (2017),^[^ ^57^ ^]^ P3 (2017),^[^ ^267^ ^]^ PBDTEDOT‐FBT (2018),^[^ ^268^ ^]^ PBDT‐FBT (2018),^[^ ^274^ ^]^ PBDTF‐FBT (2018),^[^ ^274^ ^]^ PBDTS‐FBT (2018),^[^ ^274^ ^]^ PBDTSF‐FBT (2018)^[^ ^274^ ^]^ and PBTF (2018)^[^ ^275^ ^]^ were also successfully synthesized. Among these donors, due to “F” atom with a strong electron‐withdrawing optimizes the HOMO energy level of polymer donors as well as promotes the hole/electron mobility in corresponding OSCs. Therefore, with the fluorination degree deepens, the *J_sc_* and *FF* increases. As for PBDTBT: PC_71_BM‐based, P1(1.0: 0.0): PC_71_BM‐based and PBTF: PC_71_BM based OSCs, which were obtained the *V_oc_* of 0.90, 0.76 and 0.96 V; *J_sc_* of 9.30, 12.57 and 8.00 mA cm^−2^; *FF* of 46.9%, 63.4% and 76.1%; and *PCE* of 3.93%, 5.87% and 4.81%, respectively. Among them, the significantly enhanced *FF* in PBTF: PC_71_BM‐based OSC can be attributed to the formation of more ideal blend film morphology induced by introducing long alkyl side chain into the π‐bridge unit of PBTF. In addition, Liu and co‐workers further explore the polymer skeleton BDT‐TR‐BTDz‐TR with different substitution in BDT unit.^[^ ^59^ ^]^ Afterward, PBDTDTBT‐H (2016), PBDTDTffBT‐H (2016), PBDTDTBTBT‐H (2016) and PBDTDTffBT‐R (2016) were also synthesized by introducing the substitution of benzene‐based side chain and alkyloxy group into the BDT unit. Likewise, P1(2018), PBDTsTh‐FBT (2018), PBDTsTh‐FBT (2018), PBDTsPh‐FBT (2018), PBDTsThPh‐FBT (2018) and PBDTsPhPh‐FBT (2018) were also successfully prepared by Zhu et al.^[^ ^246^ ^]^ and Liu et al.^[^ ^247^ ^]^ Interestingly, due to the synergistic optimization of benzene side chain as well as alkoxy and alkylthio groups on the energy level of polymer donors, which endows corresponding OSCs with a high *V_oc_*. Furthermore, compared with PBDTsThPh‐FBT: PC_71_BM‐based and PBDTsPhPh‐FBT: PC_71_BM‐based OSCs, the better photoelectric performance exhibited by PBDTsTh‐FBT: PC_71_BM‐based and PBDTsPh‐FBT: PC_71_BM‐based OSCs also verifies excessively extending the π‐π conjugated plane of polymer backbone will produce a negative role.

As for other D‐π‐A‐π type main‐chain engineering with BDTz derivative acceptor unit, Zhang et al.^[^ ^248^ ^]^ and Duan et al.^[^ ^276^ ^]^ reported PBDTPhBDT1 (2010), PBDTPhBDT2 (2010), PBDTTPT1 (2011) and PBDTTPD2 (2011) based on the polymer backbone BDT‐TR‐TPD‐TR. Furthermore, PBDT‐8 (2013),^[^ ^277^ ^]^ PBDT‐12 (2013)^[^ ^277^ ^]^ and PBDT‐DTPyT (2013)^[^ ^30^ ^]^ were prepared based on the main chain BDT‐TR‐TDP‐TR; PBTBSe (2013)^[^ ^164^ ^]^ and BDT‐ffBX‐DT (2018)^[^ ^278^ ^]^ were also synthesized based on the polymer backbone BDT‐TR‐BSDz‐TR and BDT‐TR‐BODz‐TR, respectively. Although such donors do not achieve an ideal photoelectric performance after applying for OSC devices, the emergence of these novel main‐chain engineering provides a series of reasonable molecular design strategy.

Based on the characterization of blend film morphology and device stability, the high *Td_5%_* obtained by PA (431.7°C), PB3 (466°C), PQ4 (447°C), PBDTTCN (410°C), PBDTTF (429°C), P1(S) (451°C), P2(Se) (441°C), P3(O) (419°C), PBDT‐FBT (407°C), PBDTF‐FBT (428°C) and PBTF (444°C) presenting a high stability. As for PBDT‐DTBTff: PC_71_BM‐based (1.10 nm) and PBDT‐DTBT: PC_71_BM‐based (2.49 nm) blend films; PBDTBSe: PC_70_BM‐based (5.13 nm) and P1(2018): PC_71_BM‐based (0.97 nm) blend films; PBDTHD‐ClBTDD: PC_71_BM‐based (2.39 nm) and PBDTBO‐ClBTDD: PC_71_BM‐based (3.10 nm) blend films as well as PBDT2FBT‐Th: PC_71_BM‐based (4.97 nm), PBDT2FBT‐Ph: PC_71_BM‐based (2.38 nm) and PBDTBP‐DTffBT: PC_71_BM‐based (2.92 nm) blend films, the decreased *RMS* is conducive to form a more ideal bi‐continuous network morphology, which endows PBDT‐DTBTff: PC_71_BM‐based (66.8%), P1(2018): PC_71_BM‐based (70.27%), PBDTHD‐ClBTDD: PC_71_BM‐based (70.82%) and PBDT2FBT‐Ph: PC_71_BM‐based (66.3%) OSCs with a higher *FF*.

**FIGURE 18:**
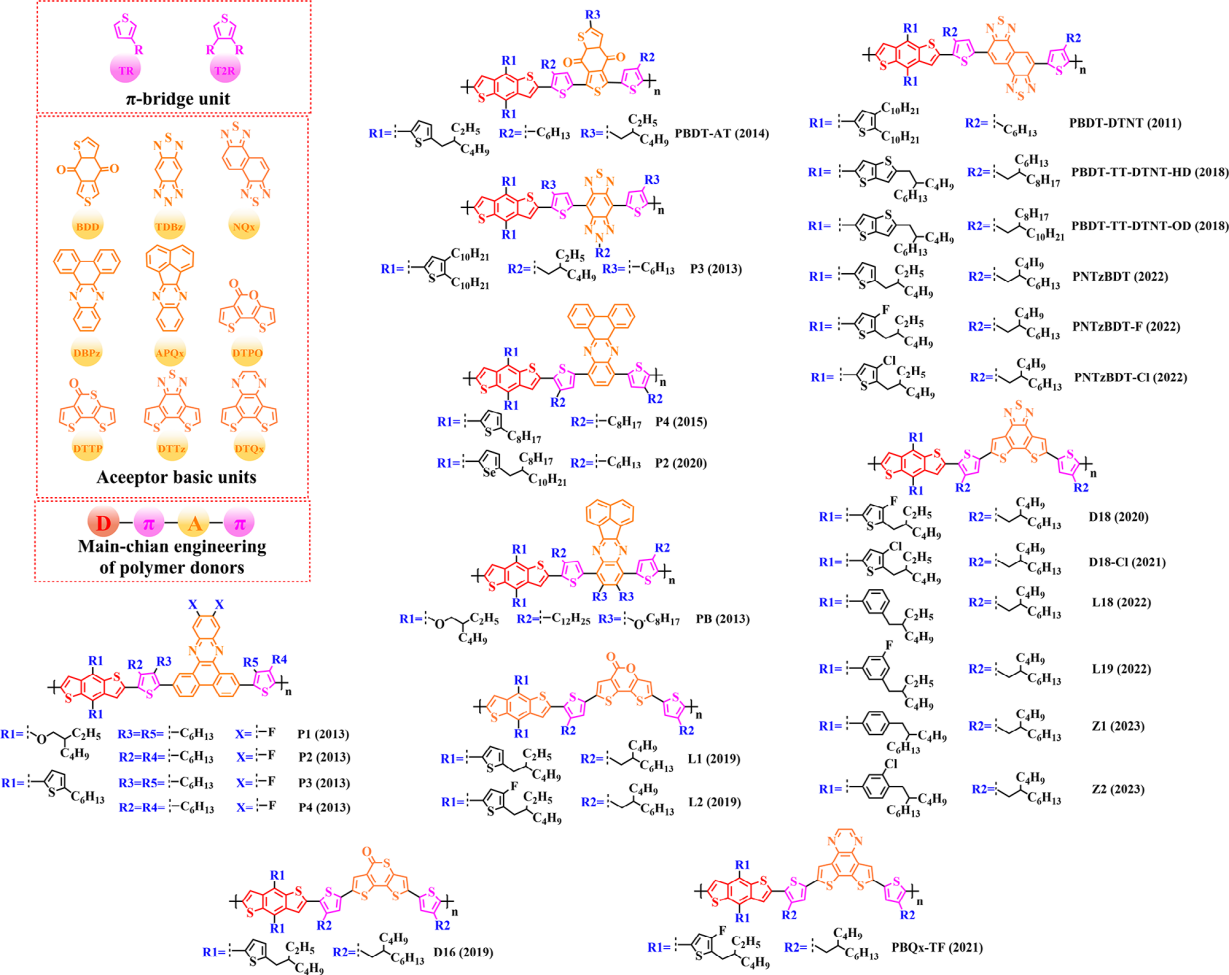
The detailed molecular structure of D‐π‐A‐π type polymer donors synthesized by introducing alkyl‐thiophene as π‐bridge unit as well as BDD, TDBz, DTTz and other functional groups as acceptor basic units.

In recent years, the emergence of the famous polymer donor D18 has greatly promoted the development of OSC, which was synthesized based on the polymer backbone BDT‐TR‐DTTz‐TR. Afterward, based on the reported results of D18, the success obtained by such polymer donors (e.g. D18‐Cl, PBQx‐TF and Z2) also demonstrates the superiority of this main‐chain engineering. Among these donor materials, the detailed molecular structure (Figure 18) and photoelectric performance (Table 15) in corresponding OSCs were discussed as follows.

**TABLE 15:** Photovoltaic parameters of OSCs related to Figure 18.

Donor	Acceptor	D/A^A^	HOMO [eV]^B^	$E_{g}^{opt}$ [eV]^C^	*V_OC_* [V]	*J_SC_* [mA cm^−2^]	*FF* [%]	*PCE* [%]^D^	Ref.
D18^a^	Y6	1:1.6	−5.51	1.98	0.859	27.70	76.6	18.01	[9]
D18	Cl‐BTA5		−5.51	1.98	1.16	14.73	67.96	11.67	[9]
P3	PC_61_BM		−5.13	1.10	0.62	0.89	32	0.25	[87]
D18‐Cl^b^	N3	1:1.4	−5.56	1.99	0.859	27.85	75.7	17.83	[133]
P4	PC_71_BM	1:2	−5.12	1.54	0.76	11.6	49	4.32	[223]
P2(2020)^c^	PC_71_BM	1:2	−5.32	1.45	0.71	5.27	55	2.07	[263]
PBDT‐DTNT	PC_71_BM	1:1	−5.19	1.58	0.80	11.71	61.0	6.00	[266]
P1	PC_60_BM	1:2	−5.37	2.26	0.86	2.36	43.4	0.88	[279]
P2	PC_70_BM	1:1	−5.32	2.33	0.63	1.72	27.4	0.30	[279]
P3	PC_60_BM	1:1	−5.24	2.17	0.83	3.37	34.5	1.21	[279]
P4	PC_70_BM	1:2	−5.49	2.20	0.68	3.32	26.6	0.60	[279]
PB	PC_61_BM	1:2	−5.54	1.81	0.78	3.58	43	1.20	[280]
PBDT‐AT^d^	PC_70_BM	1:1	−5.30	1.68	0.81	12.81	57.3	5.91	[281]
PBDT‐TT‐DTNT‐HD^e^	PC_71_BM	1:1	−5.48	1.52	0.81	11.34	43.61	3.99	[282]
PBDT‐TT‐DTNT‐OD^e^	PC_71_BM	1:1	−5.54	1.52	0.79	11.46	57.52	5.21	[282]
PNTzBDT	Y6	1:1.2	−5.26	1.63	0.77	24.2	68	11.9	[283]
PNTzBDT‐F	Y6	1:1.2	−5.36	1.58	0.79	24.4	69	12.8	[283]
PNTzBDT‐Cl	Y6	1:1.2	−5.41	1.60	0.83	21.0	60	10.0	[283]
L1^f^	Y6	1:1	−5.45	1.96	0.80	23.93	74.7	13.98	[284]
L2^f^	Y6	1:1	−5.52	1.96	0.87	20.52	70.2	12.21	[284]
D16	Y6		−5.48	1.95	0.828	25.72	75.3	16	[285]
PBQx‐TF	eC9‐2Cl	1:1.2	−5.41	2.07	0.865	25.6	78.2	17.3	[286]
L18^g^, ^h^	Y6	1:1.2	−5.43	2.03	0.89	17.3	41.8	6.4	[287]
L19^g^, ^h^	Y6	1:1	−5.47	2.03	0.93	22.9	54.6	11.7	[287]
Z1	Y6	1:1.4	−5.60	2.01	0.87	23.27	59.57	11.67	[288]
Z2	Y6	1:1.2	−5.63	2.02	0.86	24.20	72.69	14.91	[288]

^A^ Weight ratio.

^B^ The HOMO energy level of polymer donors.

^C^ Estimated from the absorption edge in film ($E_{g}^{opt}=1240/\lambda_{onset}$).

^D^ Average *PCE* of OSCs.

^a^ SVA treatment for 5 min.

^b^ 0.5% DPE.

^c^ 2% DCB.

^d^ 5% DIO.

^e^ 3% DIO.

^f^ inverted device.

^g^ 0.5% CN.

^h^ 110°C annealing for 10 min.

In 2013, Wang and co‐workers reported a series of polymer donors P1 (2013), P2 (2013), P3 (2013) and P4 (2013) based on the main chain BDT‐T2R‐DBPz‐T2R.^[^ ^279^ ^]^ After blending with acceptors, the mismatched absorption and poor surface morphology in the active layer led to a low *J_sc_* and *FF* in OSCs. Furthermore, by changing the orientation of DBPz in polymer backbone, the enhanced *J_sc_* and *FF* endow P4(2015): PC_71_BM‐based (4.32%)^[^ ^223^ ^]^ and P2 (2020): PC_71_BM‐based (2.07%)^[^ ^263^ ^]^ OSCs with a slightly improved *PCE*. Moreover, Zhang et al. adjusted the π–π conjugated plane of polymer backbone, synthesizing PB (2013) by selecting acenaphtho [1, 2‐*b*] quinoxaline (APQx) as acceptor unit.^[^ ^280^ ^]^ Similarly, P3 (2013) and PBDT‐AT (2014) were also prepared by Dong et al.^[^ ^87^ ^]^ and Bin et al.^[^ ^281^ ^]^ in turn, designing by introducing TDBz or BDD as acceptor basic units, in that order. In addition, PBDT‐DTNT (2011),^[^ ^266^ ^]^ PBDT‐TT‐DTNT‐HD (2018),^[^ ^282^ ^]^ PBDT‐TT‐DTNT‐OD (2018),^[^ ^282^ ^]^ PNTzBDT (2022),^[^ ^283^ ^]^ PNTzBDT‐F (2022)^[^ ^283^ ^]^ and PNTzBDT‐Cl (2022)^[^ ^283^ ^]^ were also synthesized based on the main‐chain engineering BDT‐TR‐NQx‐TR in subsequence. Among these donors, PNTzBDT, PNTzBDT‐F and PNTzBDT‐Cl both achieve a complemental absorption with Y6, thereby effectively enhanced the *J_sc_* in corresponding OSC devices. With the incensement of polarization in substitutions, the *V_oc_* in PNTzBDT‐based, PNTzBDT‐F‐based and PNTzBDT‐Cl‐based OSCs also presented a gradual improving trend. However, the significantly decreased *J_sc_* and *FF* in PNTzBDT‐Cl: Y6‐based OSC further verified that only appropriate polarization degree can produce positive optimization for OSC devices.

Based on such novel acceptor units (e.g. 5*H*‐dithieno [3, 2‐*b*: 2′, 3′‐*d*] pyran‐5‐one (DTPO), TDBz, 5*H*‐dithieno [3, 2‐*b*: 2′, 3′‐*d*] thiopyran‐5‐one (DTTP), and dithieno [3, 2‐f: 2′, 3′‐*h*] quinoxaline (DTQx)), Liu and co‐workers designed the main chain BDT‐TR‐DTPO‐TR and then reported two novel polymer donors L1 (2019) and L2 (2019).^[^ ^284^ ^]^ After blending with the acceptor Y6, L1: Y6‐based and L2: Y6‐based OSCs obtained the *V_oc_* of 0.80 and 0.87 V, *J_sc_* of 23.93 and 20.52 mA cm^−2^, *FF* of 74.7% and 70.2% and *PCE* of 13.98% and 12.21%, respectively. Subsequently, based on the reported results of L1 and L2, Xiong et al. constructed the main‐chain BDT‐TR‐DTTP‐TR by introducing “S” atom to replace “O” atom.^[^ ^285^ ^]^ Compared with L1: Y6‐based OSC, D16 (2019): Y6‐based OSC achieved the *V_oc_* of 0.828 V, *J_sc_* of 25.72 mA cm^−2^, *FF* of 75.3% and *PCE* of 16%. Moreover, PBQx‐TF (2021),^[^ ^286^ ^]^ which was designed based on the main‐chain engineering BDT‐TR‐DTQx‐TR, also exhibiting a promising photoelectric performance in PBQx‐TF: eC9‐2Cl‐based OSC, with the *V_oc_* of 0.865 V, *J_sc_* of 25.6 mA cm^−2^, *FF* of 78.2% and *PCE* of and 17.3%. Among them, the overall optimized photoelectric performance can be attributed to the more balanced and fast hole/electron mobility induced by forming a more matched donor and acceptor units in polymer backbone. Furthermore, the famous polymer donors D18 (2020) was also reported by Liu and co‐workers based on the polymer skeleton BDT‐T‐TDBz‐T, exhibiting a promising *PCE* (18.01%) after blending with the acceptor Y6.^[^ ^9^ ^]^ Afterward, based on the great success achieved by D18, D18‐Cl (2021),^[^ ^133^ ^]^ L18 (2021),^[^ ^287^ ^]^ L19 (2022),^[^ ^287^ ^]^ Z1 (2023)^[^ ^288^ ^]^ and Z2 (2023)^[^ ^288^ ^]^ were also successfully prepared. It is worth mentioning that the introduction of “Cl” atoms with a strong electronegative endows D18‐Cl: N3‐based OSC with an excellent *PCE* (17.83%). Indeed, the high degree of π–π conjugate plane, matching donor and acceptor units, suitable polarization of side chains as well as excellent miscibility of donor materials are key factors for D18 series donors to achieve ideal photoelectric performance in OSC devices.

Notably, it is also worth mentioned that the high *Td_5%_* presented by Z1 (431°C) and Z2 (441°C) exhibited a high stability. Moreover, based on the characterization of morphology, P3: PC_61_BM‐based (1.4 nm) and P4: PC_71_BM‐based (0.81 nm) blend films; PB: PC_61_BM‐based (2.67 nm) and PBDT‐AT: PC_70_BM‐based (2.79 nm) blend films; PBDT‐TT‐DTNT‐HD: PC_71_BM‐based (3.486 nm) and PBDT‐TT‐DTNT‐OD: PC_71_BM‐based (5.707 nm) blend films; PNTzBDT: Y6‐based (0.77 nm), PNTzBET‐F: Y6‐based (0.71 nm), PNTzBDT‐Cl: Y6‐based (0.92 nm) blend films; L18: Y6‐based (0.81 nm) and L19: Y6‐based (0.584 nm) blend films as well as Z1: Y6‐based (1.20 nm) and Z2: Y6‐based (1.47 nm) blend films, the formation of blend film morphology with a suitable *RMS* endows P4: PC_71_BM‐based (49%), PB: PC_61_BM‐based (57.3%), PBDT‐TT‐DTNT‐OD: PC_71_BM‐based (57.52%), PNTzBET‐F: Y6‐based (69%), L19: Y6‐based (54.6%) and Z2: Y6‐based (72.69 nm) with a better *FF*.

**FIGURE 19:**
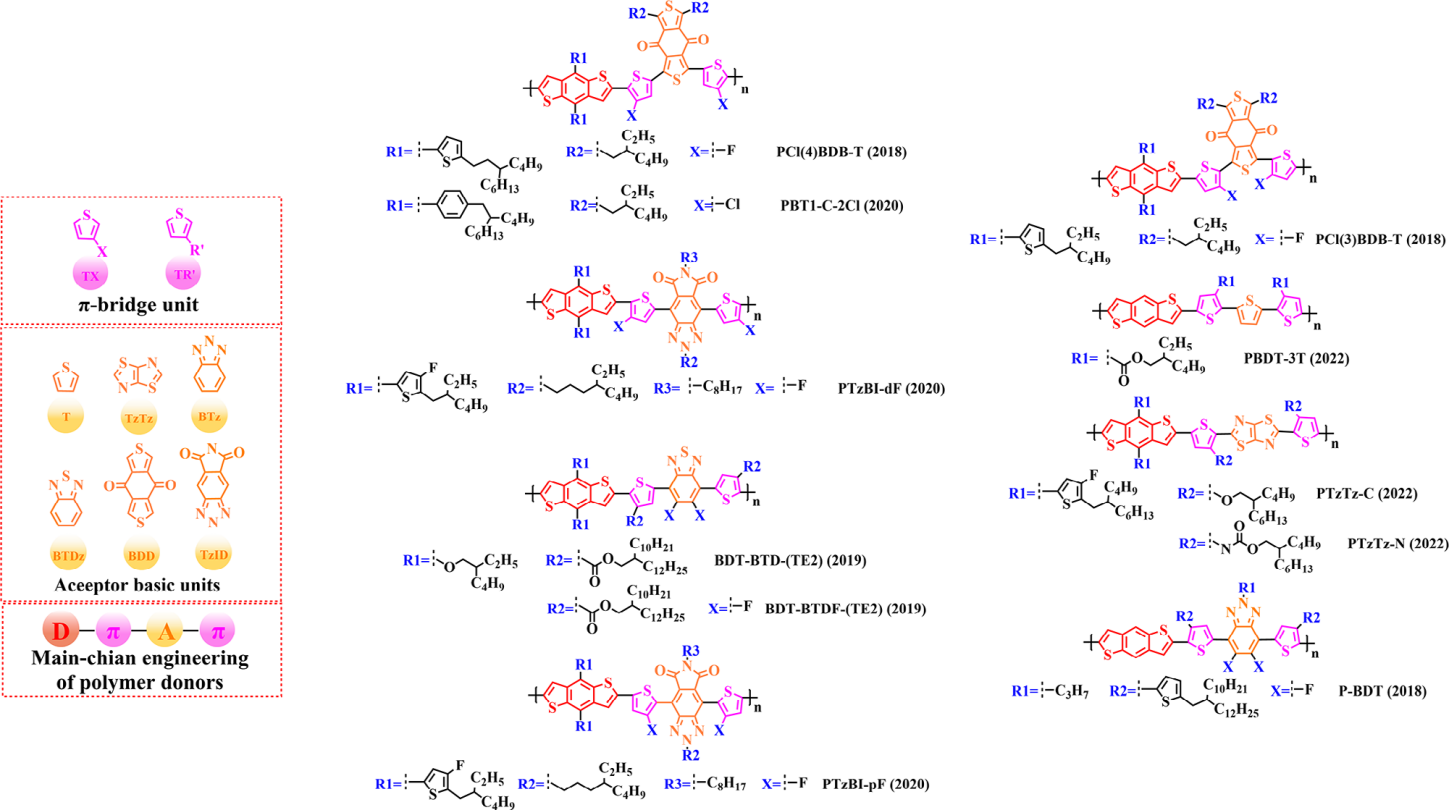
The detailed molecular structure of D‐π‐A‐π type polymer donors synthesized by introducing Halogenated and carboxylated thiophene as π‐bridge units as well as T, TzTz, BTz and other functional groups as acceptor basic units.

Thiophene or alkyl‐thiophene, as widely used π‐bridge units, exhibit superior application in main‐chain engineering. Among them, the success of PM6 and D18 is sufficient to verify the rationality of introducing π‐bridge unit into the polymer backbone. Furthermore, based on the research results of halogenated or carboxylated groups in the polymer skeleton, the molecular design strategy of constructing novel polymer backbone with halogenated or carboxylated π‐bridge units has gradually become a research hotspot. Among them, the detailed molecular structure (Figure 19) of representative polymer donors and photoelectric parameters (Table 16) in corresponding OSCs were discussed as follows.

**TABLE 16:** Photovoltaic parameters of OSCs related to Figure 19.

Donor	Acceptor	D/A^A^	HOMO [eV]^B^	$E_{g}^{opt}$ [eV]^C^	*V_OC_* [V]	*J_SC_* [mA cm^−2^]	*FF* [%]	*PCE* [%]^D^	Ref.
PCl(4)BDB‐T	IT‐4F	1.2:1	−5.48	1.78	0.84	20.60	71.09	12.33	[289]
PCl(3)BDB‐T	IT‐4F	1.2:1	−5.54	2.11	0.88	0.79	23.74	0.18	[289]
PBT1‐C‐2Cl	BTA3		−5.56	1.84	1.29	6.42	46.5	3.9	[290]
PTzBI‐df	Y6		−5.55	1.72	0.85	26.33	74.8	16.7	[291]
PTzBI‐Pf	Y6		−5.67	2.09	0.79	4.88	35.3	1.4	[291]
BDT‐BTD‐(TE2)	PCBM	1:1	−5.64	1.81	0.80	1.9	47	0.73	[292]
BDT‐BTDF‐(TE2)	PCBM	1:1	−5.76	1.82	0.90	6.5	51	3.02	[292]
PBDT‐3T^a^	ITCPTC	1:1	−5.38	1.97	0.95	13.92	56.61	7.38	[293]
PTzTz‐C	IDIC‐4F		−5.72	1.94	0.906	16.37	59.92	9.16	[294]
PTzTz‐N	BTP‐eC9		−5.53	1.81	0.870	24.14	70.99	14.91	[294]
P‐BDT^b^	ITIC‐Th	1:1.2	−5.39	1.90	0.86	14.98	67.4	8.25	[295]

^A^ Weight ratio.

^B^ The HOMO energy level of polymer donors.

^C^ Estimated from the absorption edge in film ($E_{g}^{opt}=1240/\lambda_{onset}$).

^D^ Average *PCE* of OSCs.

^a^ 0.5% DIO.

^b^ 0.2% DIO.

In 2018, Wu and co‐workers based on the reported results of PM6, introducing fluorinated thiophene group as π‐bridge unit, constructing the main chain BDT‐TX‐BDD‐TX.^[^ ^289^ ^]^ Interestingly, after adjusting the substitution site of “F” atom into the π‐bridge unit, PCl(3)BDB‐T (2018) and PCl(4)BDB‐T (2018) exhibited different photovoltaic performance in OSC devices. Among them, PCl(3)BDB‐T: IT‐4F‐based and PCl(4)BDB‐T: IT‐4F‐based OSCs obtained the *V_oc_* of 0.79 and 0.84 V, *J_sc_* of 0.79 and 20.60 mA cm^−2^, *FF* of 23.74% and 71.09%, and *PCE* of 0.18% and 12.33%, respectively. The significantly decreased *J_sc_* and *FF* in PCl(3)BDB‐T: IT‐4F‐based OSC can be attributed to the incensement of twist dihedral angle between π‐bridge and its adjacent units in the polymer backbone. Notably, excessive dihedral angel severely destroys the molecular stacking in the active layer, which not only deteriorates the blend film morphology, but also hinders the process of photon to electron transition. Likewise, an et al. also based on the main chain BDT‐TX‐BDD‐TX, prepared PBTI‐C‐2Cl (2020) by selecting chlorinated thiophene as π‐bridge unit.^[^ ^290^ ^]^ Consistent with above‐conclusion, although the *V_oc_* in PBT1‐C‐2Cl: BTA3‐based OSC has reached 1.29 V, *J_sc_* (6.42 mA cm^−2^) and *FF* (46.5%) are still low. As for PTzBI‐df (2019) and PTzBI‐pf (2019),^[^ ^291^ ^]^ which were synthesized based on the polymer skeleton BDT‐TX‐TzID‐TX, showing a consistent characterization result between PCl(3)BDB‐T and PCl(4)BDB‐T. Compared with PTzBI‐pf: Y6‐based OSC, PTzBI‐df with a smaller dihedral angel between π‐bridge and its adjacent units endows PTzBI‐df: Y6‐based OSC with a better photoelectric performance.

In addition, Jones et al. selected carboxylate substituted thiophene as π‐bridge unit, constructing the main‐chain engineering BDT‐TR’‐BTDz‐TR’ and then reporting two novel polymer donors BDT‐BTD‐(TE2) (2019) and BDT‐BTDF‐(TE2) (2019).^[^ ^292^ ^]^ Benefiting the synergistic optimization by introducing “F” atoms and carboxylate group, the fast and balanced hole/electron mobility as well as more ideal blend film morphology endow BDT‐BTDF‐(TE2): PCBM‐based OSC with a higher *V_oc_*, *J_sc_* and *FF* than BDT‐BTD‐(TE2): PCBM‐based OSC. Afterward, in order to further explore the potential application of carboxylate substituted π‐bridge unit, Gao et al. also innovatively synthesized PBDT‐3T (2022) based on the polymer backbone BDT‐TR’‐T‐TR’.^[^ ^293^ ^]^ Interestingly, without any substitutions, PBDT‐3T: ITCPTC‐based OSC achieved a promising *V_oc_* of 0.95 V, *J_sc_* of 13.92 mA cm^−2^, *FF* of 56.61% and *PCE* of 7.38%, exhibiting a great potential for development. similarly, PTzTz‐C (2022) and PTzTz‐N (2022) were also prepared by Tang et al.^[^ ^294^ ^]^ Compared with the side chain 2‐butyloctyl acetate, the introduction of “N” atoms further enhance the electronegativity of 2‐butyloctyl methyl‐*l*2‐azanecarboxylate, thus π‐bridge unit with a more suitable molecular polarity significantly optimizes the photoelectric performance of PTzTz‐N: BTP‐eC9‐based OSC, with the *V_oc_* of 0.870 V, *J_sc_* of 24.14 mA cm^−2^, *FF* of 70.99% and *PCE* of 14.91%. Among them, the higher *FF* achieved by PTzTz‐N: BTP‐eC9‐based OSC can be attributed to the formation of smoother morphology with decreased *RMS* of 2.87 nm. As for P‐BDT (2018), the introduction of alkyl‐thiophene substituted thiophene π‐bridge unit also exhibits a promising application in the main‐chain engineering BDT‐TR’‐BTz‐TR’.^[^ ^295^ ^]^

**FIGURE 20:**
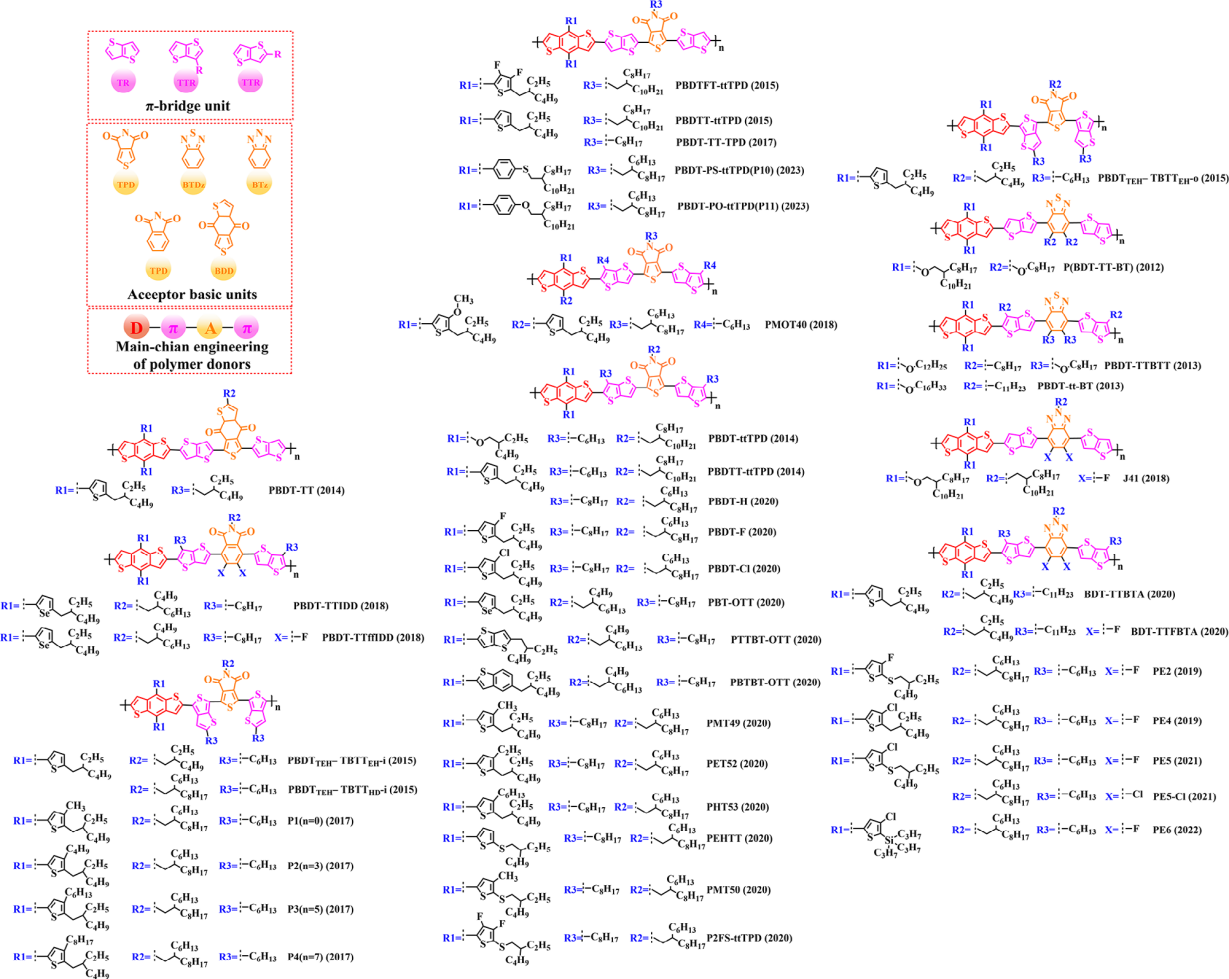
The detailed molecular structure of D‐π‐A‐π type polymer donors synthesized by introducing TT or alkyl substituted TT as π‐bridge units as well as TPD, BTDz, BDD and other functional groups as acceptor basic units.

By further extend the π–π conjugated plane of thiophene π‐bridge unit, TT as an excellent alternative π‐bridge unit, has gradually attracted the attention of researchers. Based on this molecular design strategy, a series of novel main‐chain engineering has also promoted the birth of efficient polymer donors. In this chapter, the detailed molecular structure (Figure 20) of representative polymer donors and photoelectric parameters (Table 17) in corresponding OSC devices were shown as follows.

**TABLE 17:** Photovoltaic parameters of OSCs related to Figure 20.

Donor	Acceptor	D/A^A^	HOMO [eV]^B^	$E_{g}^{opt}$ [eV]^C^	*V_OC_* [V]	*J_SC_* [mA cm^−2^]	*FF* [%]	*PCE* [%]^D^	Ref.
PBDT‐TTBTT	PC_71_BM	1:1	−5.12	1.79	0.73	9.15	53	3.54	[50]
PBDT‐ttTPD	PC_71_BM	1:1	−5.28	1.90	0.82	10.32	72	6.01	[139]
PBDTT‐ttTPD	PC_71_BM	1:1	−5.30	1.86	0.84	11.05	73	6.76	[139]
PBDT‐TT‐TPD	PC_71_BM	1:1.5	−5.21	2.03	0.74	4.88	37.2	1.36	[141]
PE5	Y6		−5.52	1.99	0.84	24.62	70.86	14.25	[183]
PE5‐Cl	Y6		−5.42	1.98	0.82	24.03	68.36	13.27	[183]
PBDT‐TT^a^	PC_70_BM	1:1.5	−5.14	1.78	0.78	5.85	31.7	1.45	[281]
P(BDT‐TT‐BT)	PC_71_BM	1:1.5	−5.21	1.78	0.69	11.34	63	4.93	[296]
PBDT‐tt‐BT	PC_70_BM	1:1	−5.21	1.62	0.73	10.58	63.6	4.91	[297]
J41^b^	ITIC	1:1			0.93	13.69	68.6	8.36	[298]
BDT‐TTBTA^c^	ITIC	1:1.5	−5.10	1.90	0.62	11.39	43	3.07	[299]
BDT‐TTFBTA^d^, ^e^	ITIC	1:1.5	−5.16	1.92	0.75	12.39	48	4.50	[299]
PE2	Y6	1:1.2	−5.32	1.93	0.83	23.24	70	13.05	[300]
PE4	Y6	1:1.2	−5.42	1.93	0.83	22.18	75.2	13.90	[301]
PE6	Y6		−5.53	1.98	0.863	25.07	69.16	15.09	[302]
PBDT‐TTIDD^f^	PC_71_BM	1:1	−5.35	2.02	0.92	7.02	58.7	3.68	[303]
PBDT‐TTffIDD^i^, ^j^	PC_71_BM	2:1	−5.54	2.04	1.02	6.95	57.8	3.98	[303]
PBDTFT‐ttTPD	PC_71_BM	1:2	−5.78	1.89	0.79	8.57	48	3.27	[304]
PBDTT‐ttTPD	PC_71_BM	1:2	−5.68	1.87	0.71	7.88	49	2.74	[304]
PBDT‐PS‐ttTPD(P10)^k^	BTP‐eC9	1:1	−5.33	1.87	0.89	22.71	63.81	12.24	[305]
PBDT‐PO‐ttTPD(P11)^l^	BTP‐eC9	1:1	−5.30	1.89	0.87	19.96	54.07	9.04	[305]
PMOT40	IDIC	1:1		1.89	0.951	17.2	70.9	11.6	[306]
PBDT‐H^m^, ^n^	Y6		−5.35	1.87	0.74	24.91	63	11.58	[307]
PBDT‐F^m^, ^n^	Y6		−5.51	1.87	0.83	25.36	68	14.37	[307]
PBDT‐Cl^m^, ^n^	Y6		−5.53	1.89	0.85	26.69	71	15.41	[307]
PBT‐OTT^a^	PC_71_BM	1:4	−5.46	1.90	0.87	14.98	62.6	8.15	[308]
PTTBT‐OTT	PC_71_BM	1:4	−5.38	1.89	0.90	9.38	50.1	4.23	[308]
PBTBT‐OTT^o^	PC_71_BM	1:4	−5.51	1.89	0.92	16.61	56.4	8.61	[308]
PMT49^p^	Y6	1:1.2	−5.35	1.88	0.81	24.99	70.3	14.0	[309]
PET52^q^	ITIC	1:1	−5.39	1.90	0.976	15.74	64.2	9.68	[309]
PHT53^q^	ITIC	1:1	−5.48	1.89	1.010	13.69	57.4	7.85	[309]
PEHTT	Y6(BO)	1:1.2			0.828	25.80	61.1	12.8	[309]
PMT50	Y6(BO)	1:1.2			0.842	26.81	67.7	15.1	[309]
P2FS‐ttTPD	ITIC‐4F	1:1	−5.70		0.97	10.29	41.6	4.15	[310]
PBDT_TEH_–TBTT_EH_‐i	PC_71_BM	1:1	−5.12	1.54	0.605	15.92	62.92	5.91	[311]
PBDT_TEH_–TBTT_HD_‐i	PC_71_BM	1:1	−5.12	1.56	0.627	15.93	64.76	6.23	[311]
PBDT_TEH_–TBTT_EH_‐o	PC_71_BM	1:1	−5.12	1.46	0.608	12.20	47.86	3.40	[311]
P1^q^	PC_71_BM		−5.18	1.61	0.72	16.36	66	7.73	[312]
P2^f^	PC_71_BM		−5.20	1.63	0.78	13.44	70	7.42	[312]
P3^q^	PC_71_BM		−5.16	1.64	0.78	15.61	75	9.10	[312]
P4^f^	PC_71_BM		−5.15	1.61	0.77	13.61	72	7.56	[312]

^A^ Weight ratio.

^B^ The HOMO energy level of polymer donors.

^C^ Estimated from the absorption edge in film ($E_{g}^{opt}=1240/\lambda_{onset}$).

^D^ Average *PCE* of OSCs.

^a^ 3% DIO.

^b^ 160°C annealing for 2 min.

^c^ 150°C annealing for 15 min.

^d^ 0.5% DIO.

^e^ 110°C annealing.

^f^ 2% DPE.

^i^ 2% CN.

^j^ 150°C annealing for 10 min.

^k^ 0.3% DIO.

^l^ 0.7% DIO.

^m^ SVA treatment for 1 min.

^n^ 100°C annealing for 10 min.

^o^ 1% DIO.

^p^ 0.5% CN.

^q^ 1% DPE.

In 2012, Wang and co‐workers based on the main‐chain engineering BDT‐TT‐BTDz‐TT, reported the polymer donor P(BDT‐TT‐BT) (2012) by further introducing the side chain 9‐(methoxymethyl) nonadecane into the BDT as well as 1‐methoxyoctane into the BTDz unit in turn.^[^ ^296^ ^]^ After blending with the acceptor PC_71_BM, P(BDT‐TT‐BT): PC_71_BM‐based OSC obtained the *V_oc_* of 0.69 V, *J_sc_* of 11.34 mA cm^−2^, *FF* of 63% and *PCE* of 4.93%. Afterward, Li et al.^[^ ^50^ ^]^ and Li et al.^[^ ^297^ ^]^ also synthesized PBDT‐TTBTT (2013) and PBDT‐ff‐BT (2013) by further introducing alkyl chain into the TT π‐bridge unit. Compared with P(BDT‐TT‐BT): PC_71_BM‐based OSC, the *V_oc_* in PBDT‐TTBTT: PC_71_BM‐based and PBDT‐ff‐BT: PC_70_BM‐based OSCs has slightly enhanced, while *J_sc_* and *FF* have not been improved. Furthermore, between the characterization results of J41 (2018),^[^ ^298^ ^]^ BDT‐TTBTA (2020)^[^ ^299^ ^]^ and BDT‐TTFBA (2020),^[^ ^299^ ^]^ the better photoelectric performance exhibited by J41: ITIC‐based OSC also verified that only introducing suitable solubilized alkyl chain into the polymer backbone can achieve positive optimization on OSC devices. Furthermore, compared with BDT‐TTFBA, a series of novel polymer donors PE2 (2019),^[^ ^300^ ^]^ PE4 (2019),^[^ ^301^ ^]^ PE5 (2021),^[^ ^183^ ^]^ PE5‐Cl (2021)^[^ ^183^ ^]^ and PE6 (2022)^[^ ^302^ ^]^ were synthesized by introducing different substitutions in polymer backbone. Among them, the introduction of tripropylsilyl effectively increases the miscibility of PE6, thereby higher *V_oc_* and *J_sc_* endows PE6: Y6‐based OSC with the best *PCE* (15.09%). In addition, PBDT‐TT (2014),^[^ ^281^ ^]^ PBDT‐TTIDD (2018)^[^ ^303^ ^]^ and PBDT‐TTffIDD (2018)^[^ ^303^ ^]^ were also synthesized by introducing TT based π‐bridge units.

Afterward, with the emergence of main chain BDT‐TT‐TPD‐TT, PBDTFT‐ttTPD (2015) and PBDT‐ttTPD (2015) were first reported by Cho et al,^[^ ^304^ ^]^ synthesizing by introducing thiophene‐based side chain into the BDT as well as alkyl group into the TPD unit, respectively. Due to the deepening of fluorination degree, PBDTFT‐ttTPD: PC_71_BM‐based OSC obtained higher *V_oc_* and *J_sc_* than PBDTT‐ttTPD: PC_71_BM‐based OSC. Subsequently, based on the reported results of PBDTT‐ttTPD, Kotturappa et al. selected alkyl chain octyl to replace the original substitution in TPD unit and then synthesizing PBDT‐TT‐TPD (2017).^[^ ^141^ ^]^ Regrettably, the shortening of the solubilized alkyl chain decreases the *J_sc_* (4.88 mA cm^−2^) and *FF* (37.2%) in PBDT‐TT‐TPD: PC_71_BM‐based OSC. Similarly, Xu et al. also prepared PBDT‐PS‐ttTPD (2023) and PBDT‐PO‐ttTPD (2023) based on the polymer skeleton BDT‐TT‐TPD‐TT.^[^ ^305^ ^]^ Benefiting the synergistic optimization by introducing benzene‐based, alkylthio, and alkyloxy side chains, PBDT‐PS‐ttTPD: BTP‐eC9‐based and PBDT‐PO‐ttTPD: BTP‐eC9‐based OSCs both obtained a high *V_oc_*. Meanwhile, the complemental absorption formed in active layer also endows corresponding OSCs with a significantly enhanced *J_sc_* and *FF*.

In addition, considering the molecular design strategy of D18, PBDT‐ttTPD (2014) and PBDTT‐ttTPD (2014) were also synthesized by Kim et al,^[^ ^139^ ^]^ designing by introducing alkyl substituted TT as π‐bridge unit. Notably, after blending with the acceptor PC_71_BM, the optimized blending surface morphology endows PBDT‐ttTPD: PC_71_BM‐based (72%) and PBDTT‐ttTPD: PC_71_BM‐based (73%) OSCs with a high *FF*. Similarly, Xie et al. innovatively constructed the polymer donor PMOT40 (2018)^[^ ^306^ ^]^; Park and co‐workers reported PBDT‐H (2020), PBDT‐F (2020) and PBDT‐Cl (2020)^[^ ^307^ ^]^; Hwang et al. synthesized PBT‐OTT (2020), PTTBT‐OTT (2020) and PBTBT‐OTT (2020)^[^ ^308^ ^]^; a series of novel polymer donors PMT49 (2020), PET52 (2020), PHT53 (2020), PEHTT (2020) and PMT50 (2020) were prepared by Zhang et al^[^ ^309^ ^]^; P2FS‐ttTPD (2020) was also designed by Ham and co‐workers.^[^ ^310^ ^]^ Among these donors, PBDT‐F: Y6‐based, PBDT‐Cl: Y6‐based and PMT49: Y6‐based OSCs exhibited relatively better photoelectric performance, with the *PCE* of 14.37%, 15.41% and 14.0%, in that order. Compared with PBDT‐H: Y6‐based OSC, by introducing side chains with a stronger polarity, PBDT‐F‐based and PBDT‐Cl‐based OSCs both obtained a better photoelectric performance, which can be attributed to the enhanced miscibility of polarized polymer donors induced by introducing octyl substituted TT as π‐bridge unit. Furthermore, by adjusting the orientation of alkyl substituted TT π‐bridge unit in polymer backbone, PBDTTEH‐TBTTEH‐i (2015), PBDTTEH‐TBTTHD‐i (2015) and PBDTTEH‐TBTTEH‐o (2015) were reported by Zhang et al.^[^ ^311^ ^]^ Subsequently, P1 (*n* = 0) (2017), P2 (*n* = 3) (2017), P3 (*n* = 5) (2017) and P4 (*n* = 7) (2017) were also successfully synthesized.^[^ ^312^ ^]^ Benefiting introducing a suitable alkyl chain, the *J_sc_* and *FF* both effectively enhanced in PBDTTEH‐TBTTHD‐i: PC_71_BM‐based OSC. As for PBDTTEH‐TBTTEH‐o: PC_71_BM‐based OSC, due to adjusting the orientation of π‐bridge unit, the twist dihedral angle between adjacent units into the polymer backbone increases, which greatly affects the molecular stacking and blend film morphology in the active layer, resulting in significantly decreased *J_sc_* and *FF* in corresponding OSC devices. Furthermore, it is also worth noting that P2: PC_71_BM‐based OSC achieved a better photoelectric performance than P1: PC_71_BM‐based, P3: PC_71_BM‐based and P4: PC_71_BM‐based OSCs, thereby further verifying the above‐conclusions that only introducing suitable alkyl chain into the polymer donors can produce a positive effect to optimize OSC devices.

Among these donors, the high *Td_5%_* presented by PBDTT‐ttTPD (422°C), BDT‐TTBTA (394°C), BDT‐TTFBT (400°C), PBDTFT‐ttTPD (414°C) exhibiting a promising stability for application. Meanwhile, based on the morphology characterization of PBDTT‐ttTPD: PC_71_BM‐based (3.7 nm) and PBDT‐TT‐TPD: PC_71_BM‐based (11.2 nm) blend films; PBDT‐tt‐BT: PC_70_BM‐based (0.45 nm) and J41: ITIC‐based (0.77 nm) blend films; PE2: Y6‐based (1.65 nm) and PE4: Y6‐based (2.09 nm) blend films; PBDT‐TTIDD: PC_71_BM‐based (1.42 nm) and PBDT‐TTffIDD: PC_71_BM‐based (2.48 nm) blend films as well as PBT‐OTT: PC_71_BM‐based (1.63 nm), PTTBT‐OTT: PC_71_BM‐based (2.26 nm) and PBTBT‐OTT: PC_71_BM‐based (0.83 nm) blend films, higher *FF* achieved by PBDTT‐ttTPD: PC_71_BM‐based (73%), J41: ITIC‐based (68.6%), PE4: Y6‐based (75.2%), PBDT‐TTIDD: PC_71_BM‐based (58.7%) and PBT‐OTT: PC_71_BM‐based (62.6%) OSCs can be attributed by forming a more ideal active layer morphology with suitable *RMS*.

**FIGURE 21:**
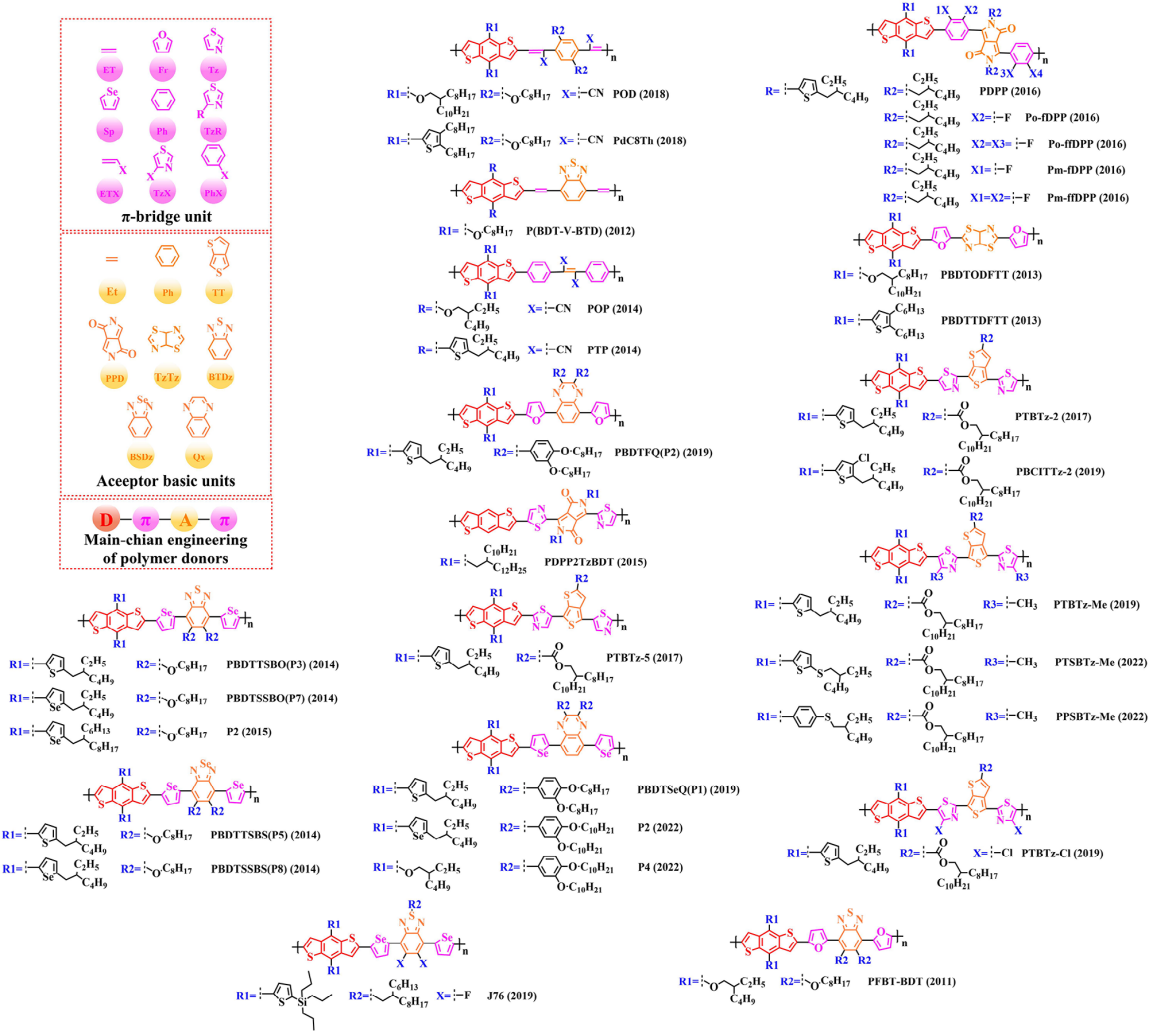
The detailed molecular structure of D‐π‐A‐π type polymer donors synthesized by introducing furan, thiazole, selenophene or phenyl as π‐bridge units as well as Et, Ph, TzTz and other functional groups as acceptor basic units.

In order to further explore the potential effect of π‐bridge unit in main‐chain engineering. The plenty of research began to select some unusual unit (e.g. furan, thiazole, selenophene and phenyl) as π‐bridge units. Among them, the detailed molecular structure (Figure 21) of representative polymer donors and photoelectric parameters (Table 18) in corresponding OSCs were discussed as follows.

**TABLE 18:** Photovoltaic parameters of OSCs related to Figure 21.

Donor	Acceptor	D/A^A^	HOMO [eV]^B^	$E_{g}^{opt}$ [eV]^C^	*V_OC_* [V]	*J_SC_* [mA cm^−2^]	*FF* [%]	*PCE* [%]^D^	Ref.
PBClTTz‐2^a^	PC_71_BM	1:1.5	−5.63	1.75	1.01	9.93	62.17	5.84	[53]
POP	PC_60_BM	1:1	−5.48	1.96	0.850	0.626	38.29	0.21	[134]
PTP	PC_60_BM	1:1	−5.47	1.95	0.975	0.473	28.37	0.13	[134]
PFBT‐BDT	PC_71_BM	1:2	−5.44	1.96	0.94	6.5	46	2.81	[155]
PBDTTSBS(P5)	PC_71_BM	1:2	−5.29	1.56	0.67	9.5	49	2.87	[156]
PBDTSSBS(P8)	PC_71_BM	1:2	−5.29	1.56	0.66	8.3	44	2.38	[156]
PBDTTSBO(P3)	PC_71_BM	1:2	−5.40	1.69	0.79	11.9	50	4.61	[156]
PBDTSSBO(P7)	PC_71_BM	1:2	−5.40	1.69	0.78	10.0	42	3.26	[156]
P2	PC_71_BM	1:2	−5.40	1.63	0.73	12.22	59.35	5.34	[157]
J76^b^, ^c^	m‐ITIC	1:1	−5.41	1.86	0.91	17.04	71.17	10.81	[182]
P2	PC_71_BM	1:2	−5.39	1.69	0.75	9.51	48.3	3.44	[201]
P4	PC_71_BM	1:2	−5.26	1.70	0.77	7.48	52.3	3.00	[201]
P(BDT‐V‐BTD)	PC_61_BM	1:1.5	−5.5	1.7	0.68	2.69	41	0.74	[313]
POD^d^	ITIC	1:1.5	−5.54	1.91	0.93	4.05	40.50	1.39	[314]
PdC8Th^d^	ITIC	1:1.5	−5.58	1.84	0.99	7.29	42.03	2.77	[314]
PDPP	PC_71_BM		−5.61		0.78	4.81	45	1.70	[315]
Po‐fdpp	PC_71_BM		−5.61		0.74	3.40	45	1.11	[315]
Po‐ffDPP	PC_71_BM		−5.65		0.80	1.67	42	0.56	[315]
Pm‐fdpp	PC_71_BM		−5.63		0.83	3.55	48	1.40	[315]
Pm‐ffDPP	PC_71_BM		−5.63		0.78	2.42	50	0.95	[315]
PBDTODFTT^e^	PC_61_BM	1:1	−5.46	2.0	0.75	5.2	48	1.87	[316]
PBDTTDFTT^f^	PC_61_BM	1:1	−5.36	1.98	0.83	7.67	48	3.06	[316]
PBDTSeQ(P1)	PC_71_BM	1:4	−5.24	1.65	0.67	13.94	43.3	4.04	[317]
PBDTFQ(P2)^g^	PC_71_BM	1:3	−5.47	1.40	0.69	8.53	48.0	2.82	[317]
PDPP2TzBDT	PCBM	1:2		1.53	0.98	6.2	53	3.2	[318]
PTBTz‐5^a^	PC_71_BM	1:1.5	−5.43	1.73	0.82	13.11	64.3	6.80	[319]
PTBTz‐2^a^	PC_71_BM	1:1.5	−5.54	1.70	0.83	16.84	69.5	9.60	[319]
PTBTz‐Cl^a^	PC_71_BM	1:1.5	−5.56	1.59	0.94	15.62	69.4	10.2	[320]
PTBTz‐Me^a^	PC_71_BM	1:1.5	−5.36	1.61	0.78	16.49	69.7	9.02	[321]
PTSBTz‐Me^a^	PC_71_BM	1:1.5	−5.57	1.69	0.89	17.34	67.39	10.03	[321]
PPSBTz‐Me^f^	PC_71_BM	1:1.5	−5.68	1.65	0.90	15.38	64.71	8.51	[321]

^A^ Weight ratio.

^B^ The HOMO energy level of polymer donors.

^C^ Estimated from the absorption edge in film ($E_{g}^{opt}=1240/\lambda_{onset}$).

^D^ Average *PCE* of OSCs.

^a^ 3% DIO.

^b^ 0.5% CN.

^c^ 130°C annealing for 2 min.

^d^ 3% DIO.

^e^ 1% DIO.

^f^ 2% DIO.

^g^ 3% DPE.

Based on the molecular design strategy of D‐π‐A‐π type polymer donors, Abbotto and co‐workers innovatively select double bonds and BTDz as π‐bridge and acceptor units in turn, constructing the main chain BDT‐ET‐BTDz‐ET.^[^ ^313^ ^]^ Afterward, by further introducing side chain 1‐methoxyoctane into the BDT unit, the novel polymer donor P(BDT‐V‐BTD) (2012) was successfully synthesized. Subsequently, Cao et al. further introduced “CN” group into the ET π‐bridge unit and synthesized two polymer donors POD (2018) and PdC8Th (2018) based on the main chain BDT‐ETX‐Ph‐ETX.^[^ ^314^ ^]^ Benefiting the synergistic optimization of introducing alkyloxy and “CN” groups, POD: ITIC‐based (0.93 V) and PdC8Th: ITIC‐based (0.99 V) OSCs both obtained a higher *V_oc_*. Furthermore, by selecting phenyl as π‐bridge unit, POP (2014), PTP (2014) and PDPP (2016) were also synthesized by Lu et al.^[^ ^134^ ^]^ and Jiang et al.^[^ ^315^ ^]^, which were designed based on the polymer skeleton BDT‐Ph‐ET‐Ph. Afterward, Po‐fDPP (2016), Po‐ffDPP (2016), Pm‐fDPP (2016) and Pm‐ffDPP (2016) were also prepared based on the reported results of PDPP, synthesizing by introducing fluorinated benzene as π‐bridge unit. Regrettably, after applying for OSC devices, low *J_sc_* and *FF* still seriously obstacle the breakthrough of *PCE* in such types of OSC devices.

In addition, furan, thiazole and selenophene were also exhibited an excellent applicability as π‐bridge unit. As for furan π‐bridge unit, PFBT‐BDT (2011) was first reported by Wang et al.^[^ ^155^ ^]^, which was designed based on the main chain BDT‐Fr‐BTDz‐Fr; PBDTODFTT (2013) and PBDTTDFTT (2013) were synthesized based on the polymer backbone BDT‐Fr‐TzTz‐Fr^[^ ^316^ ^]^; PBDTFQ(P2) (2019) was also prepared based on the polymer skeleton BDT‐Fr‐Qx‐Fr.^[^ ^317^ ^]^ Among these donors, the high *V_oc_* (0.94 V) presented in PBFT‐BDT: PC_71_BM based OSC exhibits a promising development potential. Furthermore, PDPP2TzBDT (2015),^[^ ^318^ ^]^ PTBTz‐5 (2017),^[^ ^319^ ^]^ PTBTz‐Cl (2019),^[^ ^320^ ^]^ PTBTz‐Me (2019),^[^ ^320^ ^]^ PTSBTz‐Me (2022)^[^ ^321^ ^]^ and PPSBTz‐Me (2022)^[^ ^321^ ^]^ were synthesized by introducing thiazole based π‐bridge units. Notably, the introduction of chlorinated thiazole π‐bridge unit effectively enhanced the *V_oc_* in PTBTz‐Cl: PC_71_BM‐based OSC. Furthermore, the introduction of alkyl substituted thiazole π‐bridge is also conducive to further increase the miscibility of polymer donors into the active layer, thereby PTBTz‐Me: PC_71_BM‐based, PTSBTz‐Me: PC_71_BM‐based and PPSBTz‐Me: PC_71_BM‐based OSCs were obtained a relatively higher *J_sc_* and *FF*. Moreover, based on the π‐bridge unit selenophene, PBDTTSBO(P3) (2014),^[^ ^156^ ^]^ PBDTSSBO(P7) (2014)^[^ ^156^ ^]^ and P2 (2015)^[^ ^157^ ^]^ were reported based on the main chain BDT‐Sp‐BTDz‐Sp; PBDTTSBS(P5) (2014) and PBDTSSBS(P8) (2014) were prepared based on the polymer backbone BDT‐Sp‐BSDz‐Sp^[^ ^156^ ^]^; J76 (2019) was synthesized based on the polymer skeleton BDT‐Sp‐BTDz‐Sp; BDTSeQ(P1) (2019),^[^ ^317^ ^]^ P2 (2022)^[^ ^201^ ^]^ and P4 (2022)^[^ ^201^ ^]^ were also designed based on the main‐chain engineering BDT‐Sp‐Qx‐Sp, in that order. Notably, compared with the reported results between PFBTFQ: PC_71_BM‐based and PBDTSeQ: PC_71_BM‐based OSCs, selenophene π‐bridge unit exhibited a high adaptability in main‐chain engineering, which endowed PBDTSeQ: PC_71_BM‐based OSC with an optimized photovoltaic performance. Moreover, the consistent characterization results between PFBT‐BDT: PC_71_BM‐based and PBDTTSBO: PC_71_BM‐based OSCs also further verified the above‐conclusions.

Furthermore, the high *Td_5%_* exhibited by J76 (452°C), PDPP (442°C), Po‐fdpp (431°C), Po‐ffDPP (421°C), Pm‐fdpp (444°C) and Pm‐ffDPP (440°C) also verifying the excellent stability for OSC devices. Meanwhile, among the characterization of active layer morphology, J76: m‐ITIC‐based and PTSBTz‐Me: PC_71_BM‐based OSCs both presented a high *FF*, which further prove the above‐conclusion that the fine morphology of the active layer often endows corresponding photovoltaic device with a promising *FF*.

#### 3.1.4 Multicomponent polymer donors

**FIGURE 22:**
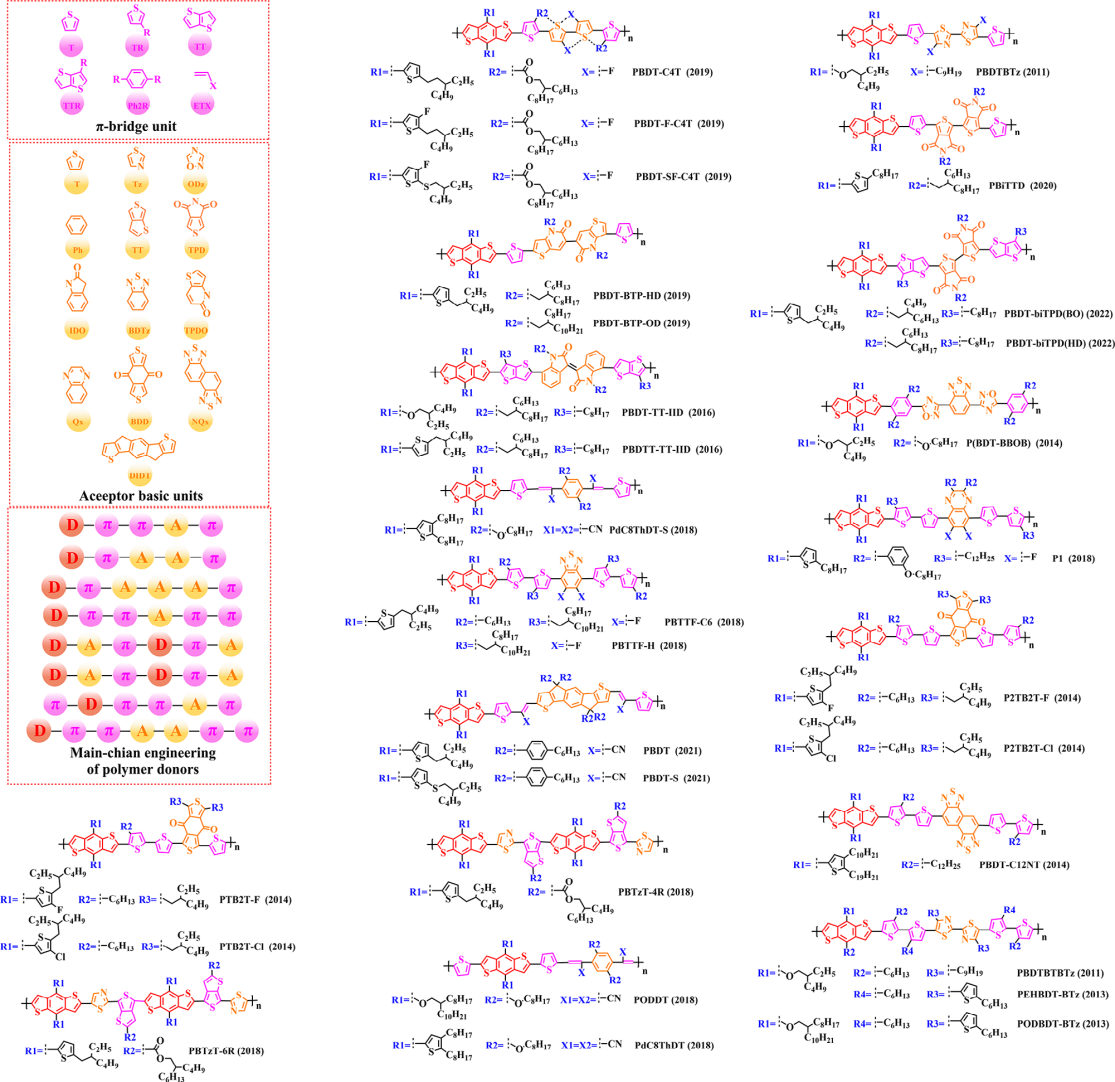
The detailed molecular structure of multicomponent polymer donors with different types of π‐bridge and acceptor units.

In recent years, with the great success achieved by D‐π‐A‐π type polymer donors, some research teams began to design a series of multicomponent polymer donors by further adjusting the proportion of donors, acceptors or π‐bridge units in the main‐chain engineering. In this chapter, the detailed molecular structure (Figure 22) of representative donor materials and photoelectric parameters (Table 19) in corresponding OSCs were shown as follows.

**TABLE 19:** Photovoltaic parameters of OSCs related to Figure 22.

Donor	Acceptor	D/A^A^	HOMO [eV] ^B^	$E_{g}^{opt}$ [eV]^C^	*V_OC_* [V]	*J_SC_* [mA cm^−2^]	*FF* [%]	*PCE* [%]^F^	Ref.
PBTzT‐6R^a^	PC_71_BM	1:1.5	−5.32	1.64	0.77	16.38	69.2	9.01	[127]
PBTzT‐4R^b^	PC_71_BM	1:1.5	−5.35	1.63	0.77	17.31	70.1	9.48	[127]
P(BDT‐BBOB)^c^	PC_61_BM	1:2	−5.31	2.32	0.70	9.59	49.43	3.31	[129]
PBiTPD^d^, ^e^	Y6	1:1.2	−5.20	1.75	0.83	25.6	66.7	14.1	[143]
PBTTF‐C6^f^	PC_71_BM	1:1.5	−5.53	1.68	0.97	2.60	50.9	1.15	[275]
PBTTF‐H^f,f^	PC_71_BM	1:1.5	−5.41	1.64	0.77	13.67	64.2	6.63	[275]
PdC8ThDT‐SD	ITIC	1:1.5	−5.58	1.88	0.89	11.14	71.92	7.02	[314]
PODDT^b^	ITIC	1:1.5	−5.51	1.91	0.87	7.62	53.95	3.35	[314]
PdC8ThDT^b^	ITIC	1:1.5	−5.57	1.86	0.89	11.76	74.18	7.43	[314]
PBDTBTz	PC_70_BM	1:1			0.86	7.84	57	3.82	[322]
PBDTBTBTz	PC_70_BM	1:1	−5.12	1.96	0.82	9.01	60.3	4.46	[322]
PEHBDT‐BTz^b^	PC_61_BM	1:1	−5.19	1.92	0.78	10.30	46.1	3.71	[323]
PODBDT‐BTz^b^	PC_61_BM	1:1	−5.26	1.91	0.82	7.18	49.7	2.92	[323]
PBDT‐TT‐IID^b^	PC_71_BM	1:1.5	−5.37	1.61	0.79	12.24	65	6.26	[324]
PBDTT‐TT‐IID^g^	PC_71_BM	1:1.5	−5.36	1.63	0.79	14.14	72	7.88	[324]
PBDT‐BTP‐HD^g^, ^h^	IT‐M	1:1	−5.22	1.90	0.89	14.82	71.78	9.18	[325]
PBDT‐BTP‐OD^h^, ^i^	IT‐M	1:1.5	−5.30	1.92	0.91	14.38	72.66	9.25	[325]
PBDT‐C4T^d^	IT‐4F	1:1	−5.58	1.96	0.80	15.4	64.3	7.6	[326]
PBDT‐F‐C4T^d^	IT‐4F	1:1	−5.63	1.95	0.94	19.0	70.3	12.0	[326]
PBDT‐SF‐C4T^d^	IT‐4F	1:1	−5.66	1.94	0.95	16.1	65.1	9.3	[326]
PBDT‐biTPD(BO)	PC_71_BM		−5.33	1.80	0.89	13.23	71.40	8.48	[327]
PBDT‐biTPD(BO)	IT‐4F		−5.33	1.80	0.91	13.44	73.54	8.99	[327]
PBDT‐biTPD(HD)	PC_71_BM		−5.38	1.80	0.82	17.08	66.31	9.32	[327]
PBDT‐biTPD(HD)	IT‐4F		−5.38	1.80	0.85	16.59	66.99	9.49	[327]
P2TB2T‐F	IT‐4F	1:1	−5.54	1.81	0.81	10.25	44.31	3.53	[328]
P2TB2T‐Cl	IT‐4F	1:1	−5.55	1.80	0.81	11.17	47.88	3.82	[328]
PTB2T‐F	IT‐4F	1:1	−5.67	1.79	0.81	20.54	65.23	10.43	[328]
PTB2T‐Cl	IT‐4F	1:1	−5.57	1.81	0.84	16.02	54.85	7.10	[328]
PBDT‐C12NT^a^	PC_61_BM	1:1	−5.19	1.51	0.68	10.74	68.53	5.00	[329]
P1(2018)	PCBM	1:1	−5.87		0.75	8.28	52.6	3.27	[330]
PBDT^j^	Y6	1:1.2	−5.48	1.87	0.88	22.16	51.31	10.04	[331]
PBDT‐S^j^	Y6	1:1.2	−5.51	1.85	0.90	16.18	46.99	6.90	[331]

^A^ Weight ratio.

^B^ The HOMO energy level of polymer donors.

^C^ Estimated from the absorption edge in film ($E_{g}^{opt}=1240/\lambda_{onset}$).

^D^ Average *PCE* of OSCs.

^a^ 2% DIO.

^b^ 3% DIO.

^c^ isopropyl alcohol (IPA).

^d^ 0.5% DIO.

^e^ 110°C annealing for 10 min.

^f^ 5% DPE.

^g^ 120°C annealing for 10 min.

^h^ 1% DIO.

^i^ 140°C annealing for 10 min.

^j^ 110°C annealing for 5 min.

In 2011, Zhang and co‐workers constructed the D‐π‐A‐A‐π type main chain BDT‐T‐Tz‐Tz‐T and then reported PBDTBTz (2011) by further introducing the side chain alkyloxy into the BDT unit as well as liner alkyl chain into the Tz units, respectively.^[^ ^322^ ^]^ After blending with the acceptor PC_70_BM, PBDTBTz: PC_70_BM‐based OSC obtained the *V_oc_* of 0.86 V, *J_sc_* of 7.84 mA cm^−2^, *FF* of 57% and *PCE* of 3.82%. Benefiting the highly π–π conjugated plane formed in polymer backbone, the *V_oc_* in PBDTBTz: PC_70_BM‐based OSC stay at a high level. Moreover, based on the molecular structure of PBDTBTz, PEHBDT‐BTz (2013) and PODBDT‐BTz (2013) were also explored by Shen et al.^[^ ^323^ ^]^ Subsequently, based on the D‐π‐A‐A‐π type polymer skeleton, PBDT‐TT‐IID (2016) and PBDTT‐TT‐IID (2016) were also synthesized by Zhu et al.^[^ ^324^ ^]^, which were designed based on the main chain BDT‐TTR‐IID‐TPDO‐IID. Compared with PBDTBTz based OSC, the introduction of more efficient acceptor unit 5*H*‐4λ^2^‐thieno [3, 2‐*b*] pyridin‐5‐one (TPDO), side chain 2‐(2‐ethylhexyl)−5‐methylthiophene, π‐bridge thieno [3, 2‐*b*] thiophene with further extended conjugate plane as well as solubilized alkyl chain 7‐ethylpentadecane, which were effectively enhanced the *J_sc_* and *FF* in PBDT‐TT‐IID: PC_71_BM‐based and PBDTT‐TT‐IID: PC_71_BM‐based OSCs. Afterward, based on the reported results of PBDT‐TT‐IID and PBDTT‐TT‐IID, Wang et al. designed the polymer backbone BDT‐T‐TPDO‐TPDO‐T and then preparing two novel polymer donors PBDT‐BTP‐HD (2019) and PBDT‐BTP‐OD (2019).^[^ ^325^ ^]^ Due to the formation of more suitable conjugated plane and more complemental absorption with acceptor in the active layer, PBDT‐BTP‐HD: IT‐M‐based and PBDT‐BTP‐OD: IT‐M‐based OSCs both exhibit a better photoelectric performance. Among them, the promising *V_oc_* (∼0.90 V) and *FF* (∼70%) also verifying the potential development of polymer backbone BDT‐T‐TPDO‐T. Furthermore, a series of novel polymer donors PBDT‐C4T (2019),^[^ ^326^ ^]^ PBDT‐F‐C4T (2019),^[^ ^326^ ^]^ PBDT‐SF‐C4T (2019),^[^ ^326^ ^]^ PBiTPD (2020),^[^ ^143^ ^]^ PBDT‐biTPD(BO) (2022)^[^ ^327^ ^]^ and PBDT‐biTPD (HD) (2022)^[^ ^327^ ^]^ were reported by different research teams in subsequence. Among them, PBDT‐F‐C4T: IT‐M‐based and PBiTPD: Y6‐based OSCs both present a relatively better photovoltaic performance, with the *PCE* of 12.0% and 14.1% in turn. Among them, the success obtained by PBiTPD: Y6‐based OSC can be attributed to the synergistic optimization effect induced by introducing “F” atoms and carboxylate‐substituted thiophene as π‐bridge unit. As for D‐π‐A‐A‐A‐π type main‐chain engineering, P(BDT‐BBOB) (2014) was also reported by Agneeswari et al.^[^ ^129^ ^]^, which was synthesized based on the novel polymer backbone BDT‐Ph2R‐ODz‐BDTz‐ODz‐Ph2R.

In addition, Lin and Co‐workers also explored the polymer backbone BDT‐TR‐T‐BDD‐T, synthesizing two novel polymer donors PTB2T‐F (2014) and PTB2T‐Cl (2014).^[^ ^328^ ^]^ Although the enhanced molecular polarity of PTB2T‐Cl endows PTB2T‐Cl: IT‐4F‐based OSC (0.84 V) with a higher *V_oc_* than PTB2T‐F: IT‐4F‐based OSC (0.81 V), *J_sc_* and *FF* decrease, which further proving that only appropriate polarization can produce a positive effect on corresponding OSC devices. Subsequently, P2TB2T‐F (2014) and P2TB2T‐Cl (2014) were also synthesized by further constructing the main chain BDT‐TR‐T‐BDD‐T‐TR with an extended conjugated plane.^[^ ^328^ ^]^ However, *J_sc_* and *FF* were significantly decreased in P2TB2T‐F: IT‐4F‐based and P2TB2T‐Cl: IT‐4F‐based OSCs, which can be attributed to the degradation of light absorbance as well as blend film morphology in the active layer. In order to further explore the molecular design strategy of D‐π‐π‐A‐π‐π type main‐chain engineering, PBDT‐C12NT (2014),^[^ ^329^ ^]^ P1 (2018),^[^ ^330^ ^]^ PdC8ThDT‐S (2018),^[^ ^314^ ^]^ PBTTF‐C6 (2018),^[^ ^275^ ^]^ PBTTF‐H (2018),^[^ ^275^ ^]^ PBDT (2021)^[^ ^331^ ^]^ and PBDT‐S (2021)^[^ ^331^ ^]^ were reported by different research teams. Among them, although the highly conjugated molecular structure endows most polymer donors with a high *V_oc_* in OSC devices, especially for PBDT: Y6‐based (0.88 V), PBDT‐S: Y6‐based (0.90 V), PdC8ThDT‐S: ITIC‐based (0.89 V) and PBTTF‐C6: PC_71_BM‐based (0.97 V) OSCs, low *J_sc_* and *FF* are still major obstacles.

As for other types of main‐chain engineering, PBDTzT‐4R (2018) and PBDTzT‐6R (2018) were synthesized by Zhu et al.,^[^ ^127^ ^]^ designing based on the main chain BDT‐Tz‐TTR‐BDT‐TTR‐Tz. At the same year, after constructing the polymer backbone T‐BDT‐T‐ETX‐Ph‐ETX, PODDT (2018) and PdC8ThDT (2018) were also prepared by Cao and co‐workers.^[^ ^314^ ^]^ After applying for the OSC devices, PBDTzT‐4R: PC_71_BM‐based, PBDTzT‐6R: PC_71_BM‐based, PODDT: ITIC‐based and PdC8ThDT: ITIC‐based OSCs obtained the *V_oc_* of 0.77, 0.77, 0.87 and 0.89 V; *J_sc_* of 17.31, 16.38, 7.62 and 11.76 mA cm^−2^; *FF* of 70.1%, 69.2%, 53.95% and 74.18%; and *PCE* of 9.48%, 9.01%, 3.35% and 7.43%, respectively. Among them, by adjusting the orientation of π‐bridge units in the polymer skeleton, the better photovoltaic performance presented by PBDTzT‐4R: PC_71_BM‐based OSC than PBDTzT‐6R: PC_71_BM‐based OSC can be attributed to the further optimized molecular stacking inside the active layer induced by the formation of more ideal conjugated structure in PBDTzT‐4R. Furthermore, the high *V_oc_* exhibited by PODDT: ITIC‐based and PdC8ThDT: ITIC‐based OSCs also verifying the potential development of multicomponent polymer donors.

Moreover, PBiTPD (440°C), P2TB2T‐F (408°C), P2TB2T‐Cl (404°C), PTB2T‐F (418°C) and PTB2T‐Cl (420°C) with an excellent *Td_5%_* can be applying for high temperature environment. Compared with PBTzT‐6R: PC_71_BM‐based (1.62 nm) blend film, the formation of more ideal active layer endows PBTzT‐4R: PC_71_BM‐based (2.89 nm) blend film with a higher *FF* (70.1%) after applying for OSC devices. Similarly, among PODDT: ITIC‐based (2.70 nm) and PdC8ThDT: ITIC‐based (1.19 nm) blend films; PBDT‐TT‐IID: PC_71_BM‐based (2.01 nm), PBDTT‐TT‐IID: PC_71_BM‐based (1.98 nm), PBDT‐BTP‐HD: IT‐M‐based (2.48 nm) and PBDT‐BTP‐OD: IT‐M‐based (2.30 nm) blend films; P2TB2T‐F: IT‐4F‐based (6.84 nm), P2TB2T‐Cl: IT‐4F‐based (4.79 nm), PTB2T‐F: IT‐4F‐based (2.55 nm) and PTB2T‐Cl: IT‐4F‐based (2.67 nm) blend films, the higher *FF* achieved by PdC8ThDT: ITIC‐based (74.18%), PBDTT‐TT‐IID: PC_71_BM‐based (72%), PBDT‐BTP‐OD: IT‐M‐based (72.66%) and PTB2T‐F: IT‐4F‐based (65.23%) OSCs further proving the above‐conclusion.

### 3.2 Side‐chain engineering

Without any doubt, main‐chain engineering, as an efficient molecular design strategy, has already been widely used to optimize the photovoltaic performance of polymer donors. Furthermore, compared with using solvent additives, side‐chain engineering is more convenient to optimize polymer donors by effectively adjusting the hole/electron mobility, molecular stacking as well as miscibility of donor materials in the active layer. Therefore, to further exploring the potential application of side‐chain engineering, we will detailedly analyze such efficient side‐chain engineering (i.e. the substitution of “F” or “Cl” atoms as well as alkoxy, alkylthio, thiophene, or benzene groups.) into the polymer donors, investigating the correlation between the photovoltaic performance in OSC devices and polymer donors, thereby providing reasonable molecular design strategy for synthesizing ideal polymer donors in future.

#### 3.2.1 Substitution of fluorine, chlorine, bromine and cyano atoms

**FIGURE 23:**
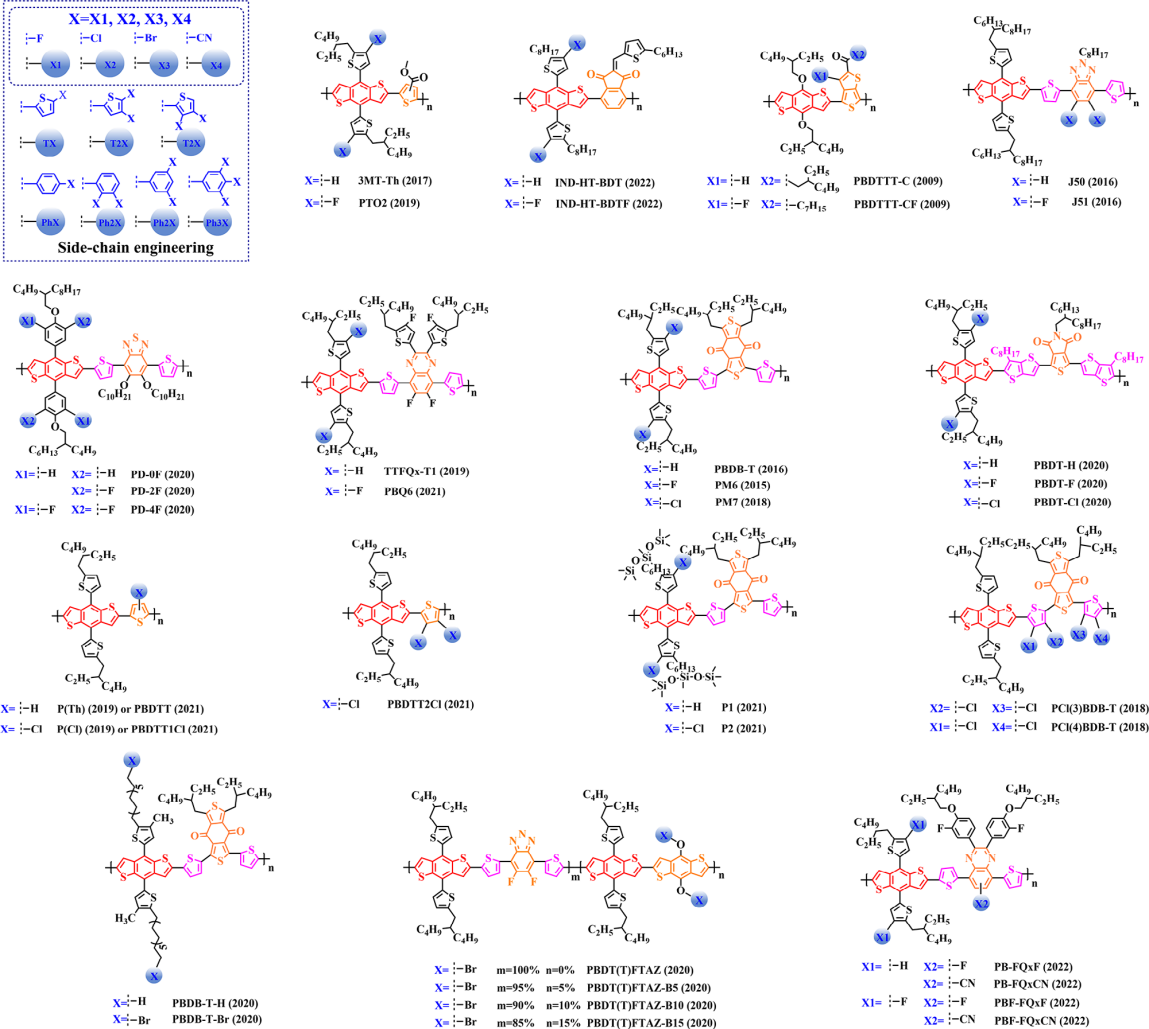
The detailed molecular structure of polymer donors synthesized by introducing fluorine, chlorine, bromine or cyano atoms.

Indeed, introducing “F”, “Cl”, “Br” and “CN” groups with electron‐withdrawing into the donor unit is a common molecular design strategy to adjust the HOMO and LUMO energy levels of polymer donors. Generally, the substitution of such functional groups with appropriate electronegativity is conducive to decrease HOMO energy level in polymer donors, thereby endows corresponding OSC devices with a high *V_oc_*, which can be confirmed in plenty of above‐characterizations. In this chapter, by selecting representative polymer donors for comparison, the detailed molecular structure (Figure 23) of donor materials and photovoltaic parameters (Table 20) in corresponding OSCs were shown as follows.

**TABLE 20:** Photovoltaic parameters of OSCs related to Figure 23.

Donor	Acceptor	D/A^A^	HOMO [eV]^B^	$E_{g}^{opt}$ [eV]^C^	*V_OC_* [V]	*J_SC_* [mA cm^−2^]	*FF* [%]	*PCE* [%]^D^	Ref.
PM6	IT‐4F	1:1	−5.54	1.81	0.87	20.39	75	13.3	[4, 5]
PM7	IT‐4F	1:1	−5.40		0.86	21.80	76	14.0	[7]
PBDTT1Cl	Y18‐1F	1:1	−5.47	2.03	0.87	26.43	71.35	16.95	[8]
PBDTT2Cl	Y18‐1F	1:1	−5.60	2.01	0.90	20.92	58.60	11.37	[8]
P(Th)^a^, ^b^	ITIC‐Th	1:1.25	−5.32	2.03	0.779	8.8	37.3	2.0	[27]
P(Cl)^a^, ^b^	ITIC‐Th	1:1.25	−5.47	1.97	0.859	18.2	68.9	10.7	[27]
3MT‐Th	ITIC	1:1	−5.42	2.00	0.95	15.96	53.60	7.97	[35]
PTO2	IT‐4F	1:1	−5.59		0.91	21.5	75	14.4	[42]
PBDTTT‐CF	PC_70_BM	1:1.5	−5.22		0.76	15.2	66.9	7.4	[46]
PBDTTT‐C	PC_70_BM	1:1.5	−5.07	1.60	0.70	15.51	59.2	6.43	[47]
IND‐HT‐BDT	Y6BO	3:5	−5.44	1.86	0.88	5.80	40.0	2.05	[86]
IND‐HT‐BDTF	Y6BO	3:4	−5.47	1.87	0.82	13.21	59.22	6.38	[86]
PD‐0F	rr‐PBN		−5.09	1.80	0.86	9.42	46.5	3.72	[160]
PD‐2F	rr‐PBN		−5.20	1.80	0.92	8.35	41.9	3.15	[160]
PD‐4F	rr‐PBN		−5.27	1.82	1.11	11.53	50.4	6.38	[160]
TTFQx‐T1^c^, ^d^	Y5	1:1.2	−5.31	1.70	0.89	21.2	69.6	12.9	[215]
PBDB‐T	ITIC	1:1	−5.33	1.82	0.888	16.23	63.5	9.03	[216]
J50	N2200	2:1	−5.13	1.88	0.60	13.93	58.74	4.80	[216]
J51	N2200	2:1	−5.26	1.91	0.83	14.18	70.24	8.10	[216]
PBQ6^e^, ^f^	Y6	1:1.3	−5.64	1.71	0.845	26.33	77.47	17.34	[217]
PB‐FQxF^g^	Y6	1:1.5	−5.04	1.79	0.54	22.97	52.39	6.46	[218]
PBF‐FQxF^g^	Y6	1:1.5	−5.08	1.81	0.63	24.00	58.06	8.73	[218]
PB‐FQxCN^g^	Y6	1:1.5	−5.17	1.68	0.68	25.15	63.94	10.90	[218]
PBF‐FQxCN^g^	Y6	1:1.5	−5.26	1.69	0.76	24.19	62.59	11.51	[218]
PBDB‐T‐H^b^, ^f^	Y6	1:1.2	−5.69	1.82	0.79	20.65	60	9.76	[232]
PBDB‐T‐Br^b^	Y6	1:1	−5.51	1.82	0.79	25.26	65	12.88	[232]
P1	IT‐4F		−5.34	1.81	0.67	17.78	48.98	5.40	[234]
P2	IT‐4F		−5.50	1.84	0.84	17.43	69.78	10.25	[234]
PCl(3)BDB‐T	IT‐4F	1.2:1	−5.54	2.11	0.88	0.79	23.74	0.18	[289]
PCl(4)BDB‐T	IT‐4F	1.2:1	−5.48	1.78	0.84	20.60	71.09	12.33	[289]
PBDT‐H^h^	Y6		−5.35	1.87	0.74	24.91	63	11.84	[307]
PBDT‐F^h^	Y6		−5.51	1.87	0.83	25.36	68	14.86	[307]
PBDT‐Cl^h^	Y6		−5.53	1.89	0.85	25.69	71	15.63	[307]
PBDT(T)FTAZ^a^, ^h^	N2200	1.5:1	−5.28	1.94	0.80	12.17	59.84	5.83	[332]
PBDT(T)FTAZ‐B5^a^, ^h^	N2200	1.5:1	−5.27	1.96	0.80	13.11	60.11	6.30	[332]
PBDT(T)FTAZ‐B5^a^, ^h^	N2200	1.5:1	−5.27	1.96	0.80	13.07	59.53	6.22	[332]
PBDT(T)FTAZ‐B10^a^, ^h^	N2200	1.5:1	−5.27	1.95	0.80	11.57	59.39	5.50	[332]
PBDT(T)FTAZ‐B15^a^, ^h^	N2200	1.5:1	−5.27	1.96	0.80	11.28	59.95	5.41	[332]

^A^ Weight ratio.

^B^ The HOMO energy level of polymer donors.

^C^ Estimated from the absorption edge in film ($E_{g}^{opt}=1240/\lambda_{onset}$).

^D^ Average *PCE* of OSCs.

^a^ 5% DPE.

^b^ 1% DIO.

^c^ 110°C annealing for 10 min.

^d^ isopropyl alcohol (IPA).

^e^ 3% DPE.

^f^ 110°C annealing for 5 min.

^g^ 120°C annealing for 10 min.

^h^ 100°C annealing for 10 min.

Benefiting “F” atoms with a small atomic size and appropriate electronegative, fluorination has been widely used as a simple and efficient molecular designing strategy in the side‐chain engineering. Among the D‐A type polymer donors, Yao et al.^[^ ^42^ ^]^ synthesized the polymer donor PTO2 (2019) by introducing single “F” atom into the alkyl‐thiophene substit0uted BDT donor unit of 3MT‐Th (2017)^[^ ^35^ ^]^, which endows PTO2 (−5.59 eV) with a lower HOMO energy level than 3MT‐Th (−5.42 eV). Therefore, PTO2: IT‐4F based OSC obtained higher *J_sc_* and *FF* than 3MT‐Th: ITIC‐based OSC and finally achieved a more efficient *PCE* (6.43%). Similarly, Nisa and co‐workers also prepared the polymer donor IND‐HT‐BDTF (2022) by further introducing single “F” atom into the alkyl‐thiophene substituted BDT unit of IND‐HT‐BDT (2022).^[^ ^86^ ^]^ Consistent with the above‐conclusion, IND‐HT‐BDTF obtained a lower HOMO energy level, the *J_sc_* and *FF* in IND‐HT‐BDTF: Y6BO‐based OSC were also significantly improved, meanwhile, the *PCE* was also increased by 4.33%. Furthermore, it is worth noting that the *V_oc_* in PTO2 based and IND‐HT‐BDTF‐based OSCs both lower than the OSC devices fabricated by original donor without fluorination, which was different from the rule that lower HOMO energy level of donor materials was conducive to enhance the *V_oc_* in OSC devices, reminding us that the change of molecule stacking and crystal degree in the active layer were also will influence the *V_oc_*. Furthermore, Hou et al.^[^ ^46^, ^331^ ^]^ also prepared PBDTTT‐CF (2009) by further introducing single “F” atom into the TT unit of PBDTTT‐C (2009).^[^ ^47^ ^]^ Compared with PBDTTT‐C, the HOMO energy level of PBDTTT‐CF was significantly reduced, meanwhile, *V_oc_*, *FF* and *PCE* in PBDTTT‐CF: PC_70_BM‐based OSC were also increased by 0.06 V, 7.7% and 0.97%, in that order. In addition, fluorination as an efficient side‐chain engineering, which also plays an important role in designing ideal D‐π‐A‐π type polymer donors. Hou et al. synthesized J51 (2016) by introducing two “F” atoms into the BTz unit of J50 (2016), the HOMO energy level of J51 was significantly reduced.^[^ ^216^ ^]^ Meanwhile, *V_oc_*, *J_sc_* and *FF* in J51: N2200‐based OSC were significantly improved. Furthermore, Wang et al. designed PD‐2F (2020) and PD‐4F (2020) by introducing single or double “F” atoms into the alkoxy‐phenyl substituted BDT unit of the PD‐0F (2020).^[^ ^160^ ^]^ Notably, compared with PD‐0F, the increased fluorinated degree also endows PD‐2F and PD‐4F with a lower HOMO energy level as well as higher *V_oc_* in corresponding OSC devices. Moreover, the best photovoltaic performance presented by PD4F: rrPBN‐based OSC indicates only utilizing appropriate fluorination for polymer donors can realize positively optimized in OSCs. Zhu et al. based on the reported results of TTFQx‐T1 (2019),^[^ ^215^ ^]^ synthesizing PBQ6 (2021) by further introducing single “F” atom into the alkyl‐thiophene substituted BDT unit.^[^ ^217^ ^]^ Interestingly, although PBQ6 (−5.64 eV) obtained a lower HOMO energy level than TTFQx‐T1 (−5.31 eV), the *V_oc_* in PBQ6: Y6‐based OSC (0.845 V) still lower than TTFQx‐T1: Y5‐based OSC (0.89 V). Therefore, it is worth mentioned that the *V_oc_* in OSC devices is not only influenced by molecular energy level, molecular stacking, miscibility between donor and acceptor and crystallinity in the active layer both are potential influencing factors. Furthermore, *J_sc_* and *FF* in PBQ6: Y6‐based OSC were significantly improved, meanwhile, the promising *PCE* (17.34%) exhibited in this OSC device verifies the potential application of selecting fluorination as side‐chain engineering.

Indeed, fluorination as an efficient side‐chain engineering has greatly promoted the development of polymer donors. However, the cumbersome and expensive synthesis process of synthesizing fluorinated polymer donors seriously obstacles further breakthrough in OSC devices. Therefore, chlorination as another potential side‐chain engineering, has gradually attracted the attention of researchers. Compared with “F” atoms, although introducing “Cl” atoms usually results in greater steric hindrance in polymer backbone, the stronger molecular polarity as well as convenient and low‐cost chlorination process give birth to a series of efficient chlorinated polymer donors. Among D‐A type polymer donors, Jeon et al. based on the polymer backbone BDT‐T, reporting a novel polymer donor P(Th) (2019).^[^ ^27^ ^]^ Subsequently, P(Cl) (2019) was synthesized by further introducing single “Cl” atom into the thiophene unit of P(Th). Benefiting from the substitution of “Cl” atoms with a strong electron‐withdrawing, the HOMO energy level of P(Cl) is significantly reduced, thereby P(Cl): ITIC‐Th‐based OSC (0.859 V) obtained a higher *V_oc_* than P(Th): ITIC‐Th‐based OSC (0.779 V). Meanwhile, the *J_sc_* and *FF* in P(Cl): ITIC‐Th‐based OSC were also achieved a significant breakthrough. Similarly, Wang et al. synthesized two polymer donors PBDTT1Cl (2021) and PBDTT2Cl (2021) by introducing single and double “Cl” atoms into the thiophene unit of P(Th).^[^ ^8^ ^]^ With the increase of chlorination degree, the HOMO energy level of such polymer donors continues to decrease, which endows the corresponding OSC with a higher *V_oc_*. It is worth noting that although the photovoltaic performance in PBDTT1Cl: Y18‐1F‐based and PBDTT2Cl: Y18‐1F‐based OSCs both better than P(Th): ITIC‐Th‐based OSC, the *PCE* in PBDTT2Cl: Y18‐1F‐based OSC weaker than PBDTT1Cl: Y18‐1F‐based OSC. Therefore, only polymer donors with appropriate chlorination degree can make a positive optimization in OSC devices. In addition, among D‐π‐A‐π type polymer donors, Thavamani et al. synthesized P1 (2021) and P2 (2021) based on the polymer backbone BDT‐T‐BDD‐T^[^ ^234^ ^]^; Wu et al. prepared PCl(3)BDB‐T (2018) and PCl(4)BDB‐T (2018) by adjusting the substitution position of “Cl” atoms in thiophene π‐bridge.^[^ ^289^ ^]^ Interestingly, the *PCE* of P2: IT‐4F‐based OSC (10.25%) was nearly double than P1: IT‐4F‐based OSC (5.40%), which further proved the feasibility of chlorination as an efficient side‐chain engineering. What's more, the huge difference of *J_sc_* between PCl(3)BDB‐T: IT‐4F‐based (0.79 mA cm^−2^) and PCl(4)BDB‐T: IT‐4F‐based (20.60 mA cm^−2^) indicates the appropriate substitution site of “Cl” atoms should be located at π‐bridge unit with minimum steric hindrance. Moreover, we also noticed that based on the same polymer donor, chlorination often can produce a more obvious optimization than fluorination. For example, Zhang et al. prepared the polymer donor PM7 (2018)^[^ ^7^ ^]^ by introducing single “Cl” atom into the alkyl‐thiophene side chain of PBDB‐T (2016),^[^ ^216^ ^]^ while Li et al. synthesized PM6 (2015) by introducing single “F” atom.^[^ ^4^, ^5^ ^]^ Consistent with the above‐conclusion, PM7: IT‐4F‐based and PM6: IT‐4F‐based OSCs both obtained better photovoltaic performance than PBDB‐T: ITIC‐based OSC, meanwhile, the higher *J_sc_* and *FF* exhibited in PM7: ITIC‐based OSC than PM6: ITIC‐based OSC can be attributed to the formation of more ideal blending morphology as well as fast and balanced exciton dissociation after introducing chlorine atoms. Similarly, Park et al. also synthesized PBDT‐F (2020) and PBDT‐Cl (2020) by introducing single “F” atom and “Cl” atom into the alkyl‐thiophene side chain of PBDT‐H (2020), respectively.^[^ ^307^ ^]^ Compared with PBDT‐H: Y6‐based OSC, the photovoltaic performance in PBDT‐F: Y6‐based and PBDT‐Cl: Y6‐based OSCs both achieved a significant improvement, meanwhile, the better photoelectric performance presented by PBDT‐Cl: Y6‐based OSC can be attributed to the accelerated hole/electron mobility induced by introducing of “Cl” atoms with a stronger electronegative. Therefore, lower HOMO energy level of PBDT‐Cl (−5.53 eV) and finer phase separation morphology were also exhibited by PBDT‐Cl: Y6‐based blend film. Indeed, chlorination as another reliable side‐chain engineering, deserving further attention and promotion.

As for the side‐chain engineering of bromination and iodination, due to the excessive atomic radius of “Br” atoms will cause a great impact on the planarity of conjugated polymer backbone, meanwhile, iodinated polymer donors always have a poor stability in practical applications, these types of side chain engineering have not been explored in depth, especially for iodinated polymer donors, there has been no detailed research report so far. As for brominated polymer donors, Liu et al. based on the polymer backbone BDT‐T‐BDD‐T, synthesizing PBDB‐T‐Br (2020) by introducing “Br” atom as end‐capping unit into the alkyl‐thiophene substituted BDT unit.^[^ ^232^ ^]^ Compared with the PBDB‐T‐H (2020) (−5.69 eV), although the HOMO energy level of PBDB‐T‐Br (−5.51 eV) was slightly increased, while PBDB‐T‐Br: Y6 based and PBDB‐T‐H: Y6‐based OSCs both obtain a same *V_oc_* (0.79 V), indicating that introducing “Br” atom as end‐capping unit of side chain will not affect the *V_oc_* in corresponding OSC devices. In addition, the *J_sc_* and *FF* in PBDB‐T‐Br: Y6‐based OSC were also enhanced, which proved that selecting “Br” atoms as terminal functional unit in side‐chain engineering is a reasonable molecular design strategy. Similarly, Lin et al. also synthesized a novel terpolymer donor PBDT(T)FTAZ (2020) based on the main chain BDT‐T‐BTz‐T. Subsequently, PBDT(T)FTAZ‐B5 (2020), PBDT(T)FTAZ‐B10 (2020) and PBDT(T)FTAZ‐B15 (2020) were also prepared by introducing 5%, 10% or 15% third component donor unit with brominated side chains.^[^ ^332^ ^]^ Although these types of donor materials with different degree of bromination, the HOMO energy level did not change significantly. Furthermore, the best photovoltaic performance presented by PBDT(T)FTAZ‐B5: N2200‐based OSC also indicated the introduction of appropriate degree of third component donor with brominated side chain is conducive to endow corresponding OSC devices with a better photovoltaic performance. Moreover, although such all‐polymer type OSCs do not achieve an ideal *PCE*, the excellent stability exhibited in practical applications also verified the feasibility by introducing brominated groups as side‐chain engineering.

In addition, “CN” atoms with a strong electron‐withdrawing and small atomic radius, has already been widely used in side‐chain engineering. In 2022, Sagita et al. based on the polymer backbone BDT‐T‐Qx‐T, synthesized PB‐FQxCN (2022) and PBF‐FQxCN (2022) by further introducing a single “CN” atom into the Qx acceptor unit of PB‐FQxF (2022) and PBF‐FQxF (2022) in turn.^[^ ^218^ ^]^ Benefiting the substitution of “F” and “CN” atoms synergistic decreases the HOMO energy level of PB‐FQxCN (−5.17 eV) and PBF‐FQxCN (−5.26 eV), PB‐FQxFCN: Y6‐based (0.80 V) and PBF‐FQxCN: Y6‐based OSC (0.76 V) both obtained a significantly enhanced *V_oc_* than PB‐FQxF: Y6‐based and PB‐FQxCN: Y6‐based OSCs. Among them, PB‐FQxFCN: Y6‐based and PBF‐FQxCN: Y6‐based OSCs also presented a wide and flat absorption curve with an obvious red‐shift (∼50 nm). Therefore, the more complemental and efficient external quantum efficiency (*EQE*) curves (maximum *EQE* more than 75/%) endows PB‐FQxFCN: Y6‐based (21.82 mA cm^−2^) and PBF‐FQxCN: Y6‐based (24.19 mA cm^−2^) OSCs with a high *J_sc_*. Therefore, due to “CN” atoms with an intense polarity effectively enhances the intermolecular interaction and crystallinity in the blend film, thereby effectively increase the *FF* in corresponding OSC devices, which is enough to prove the potential application of such side‐chain engineering.

Among these types of polymer donors, PM7: IT‐4F‐based (2.30 nm) blend film with a suitable *RMS* endows corresponding OSCs with an excellent *FF* of 76%, meanwhile, the high *FF* achieved by PM6: IT‐4F‐based (75%), PBDTT1Cl: Y18‐1F‐based (71.35%), PBQ6: Y6‐based (77.47%), PCl(4)BDB‐T: IT‐4F‐based (71.09%) and J51: N2200‐based (70.24%) OSCs also deserves further attention

#### 3.2.2 Substitution of alkyl, alkoxy, alkylthio and carboxylate groups

**FIGURE 24:**
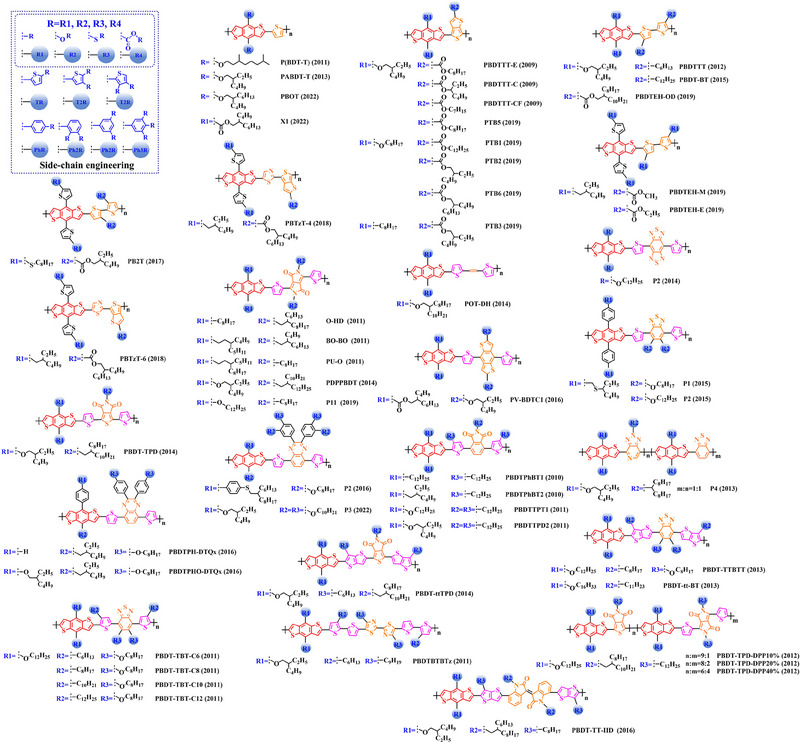
The detailed molecular structure of polymer donors with the substitution of alkyl, alkoxy, alkylthio, or carboxylate side chains.

Due to the degree of phase separation presented in the blend film surface is negative correlated with *FF* in corresponding OSC devices. Therefore, it is essential to optimize the miscibility and crystallinity of polymer donors after blending with acceptors. In order to achieve this goal, four types of functional groups alkyl, alkoxy, alkylthio and carboxylate side chains exhibited excellent application. Among them, the detailed molecular structure (Figure 24) of representative polymer donors and photovoltaic performance (Table 21) in corresponding OSCs were discussed as follows.

**TABLE 21:** Photovoltaic parameters of OSCs related to Figure 24.

Donor	Acceptor	D/A^A^	HOMO [eV]^B^	$E_{g}^{opt}$ [eV] ^C^	*V_OC_* [V]	*J_SC_* [mA cm^−2^]	*FF* [%]	*PCE* [%] ^D^	Ref.
P(BDT‐T)	PC_70_BM	1:1.5	−5.07	2.11	0.75	4.50	60.8	2.05	[25]
PABDT‐T	PC_71_BM	1:2	−5.04	2.10	0.68	7.24	39	2.03	[26]
PBOT	BTP‐eC9	1:1	−5.22	2.15	0.663	13.97	63.54	5.89	[28]
X1^a^, ^b^	BTP‐eC9	1:1	−5.44	1.85	0.845	27.62	70.69	16.5	[28]
X1^a^, ^c^	Y6	1:1	−5.44	1.85	0.833	26.19	70.50	15.4	[28]
PBDTTT‐E	PC_70_BM	1:1	−5.01	1.63	0.62	13.2	63	4.8	[46]
PBDTTT‐CF	PC_70_BM	1:1.5	−5.22		0.76	15.2	66.9	7.4	[46]
PBDTTT‐C	PC_70_BM	1:1.5	−5.07	1.60	0.70	15.51	59.2	6.43	[47]
PTB1	PC_61_BM	1:1	−4.90	1.58	0.58	12.5	65.4	4.76	[48]
PTB2	PC_61_BM	1:1	−4.94	1.59	0.60	12.8	66.3	5.10	[48]
PTB3	PC_61_BM	1:1	−5.04	1.60	0.74	13.1	56.8	5.53	[48]
PTB4	PC_61_BM	1:1	−5.12	1.63	0.76	9.20	44.5	3.10	[48]
PTB5	PC_61_BM	1:1	−5.01	1.62	0.68	10.3	43.1	3.02	[48]
PTB6	PC_61_BM	1:1	−5.01	1.61	0.62	7.74	47.0	2.26	[48]
PBDT‐TTBTT	PC_71_BM	1:1	−5.12	1.79	0.73	9.15	53	3.54	[50]
P11(2019)^d^	PC_71_BM	1:1.5	−5.23	1.34	0.83	13.88	64	7.29	[118]
PBDTTT	PCBM	1:3	−5.32	1.96	0.72	1.61	31	0.36	[119]
PBDT‐BT	PC_71_BM		−5.37	2.10	0.82	5.86	45.8	2.01	[120]
PBDTEH‐OD^e^	ITIC‐Th	1:1	−5.52	2.10	1.04	8.94	43.2	3.90	[122]
PBDTEH‐M^f^	ITIC‐Th	1:1	−5.37	1.96	0.88	14.03	50.4	6.03	[122]
PBDTEH‐E^a^, ^f^	ITIC‐Th	1:1	−5.43	1.98	0.92	14.29	58.2	7.48	[122]
PB2T^g^	IT‐M	1:1	−5.63	2.24	0.56	0.06	26	0.01	[123]
PBTzT‐4^h^	PC_71_BM	1:1.5	−5.35	1.65	0.78	16.48	69.7	9.19	[127]
PBTzT‐6^i^	PC_71_BM	1:1.5	−5.31	1.66	0.77	15.45	69.1	8.28	[127]
POT‐DH	PC_71_BM	1:1.5	−5.32	1.99	0.66	9.36	60.3	3.73	[135]
PBDT‐TPD	PC_71_BM	1:1	−5.32	1.91	0.85	6.97	68	3.89	[139]
PBDT‐ttTPD	PC_71_BM	1:1	−5.28	1.90	0.82	10.32	72	6.01	[139]
O‐HD^j^	PC_71_BM		−5.15	1.45	0.71	9.4	61	4.1	[145]
BO‐BO^j^	PC_71_BM		−5.14	1.51	0.59	3.4	46	0.93	[145]
PU‐O^j^	PC_71_BM		−5.10	1.36	0.62	5.2	43	1.4	[145]
PDPPBDT	PC_71_BM	1:2	−5.37	1.81	0.742	7.12	62	3.28	[147]
P2^(^ ^f^ ^)^	PC_61_BM	1:2	−5.34	1.73	0.81	7.72	68.79	4.28	[151]
PBDT‐TBT‐C6	PC_61_BM	1:3	−5.26	1.76	0.74	4.50	44	1.92	[154]
PBDT‐TBT‐C8	PC_61_BM	1:3	−5.29	1.77	0.70	7.19	52	2.88	[154]
PBDT‐TBT‐C10	PC_61_BM	1:3	−5.25	1.77	0.72	3.95	37	1.04	[154]
PBDT‐TBT‐C12	PC_71_BM	1:3	−5.23	1.75	0.71	8.60	51	3.15	[154]
P1	PC_71_BM	1:2.5	−5.42	1.79	0.74	11.60	63	5.4	[158]
P2	PC_71_BM	1:2.5	−5.41	1.78	0.76	11.26	72	6.1	[158]
P2^(^ ^k^ ^)^	PC_70_BM	1:1	−5.10	1.18	0.58	6.05	45	1.58	[163]
BDT‐T‐Qx‐T(P3)	PC_71_BM	1:1	−5.63	1.82	0.80	10.08	55.5	4.47	[201]
PBDTPH‐DTQx	PC_71_BM	1:2	−5.37	1.75	0.70	11.89	67.3	5.47	[221]
PBDTPHO‐DTQx	PC_71_BM	1:2	−5.34	1.74	0.75	9.60	60.4	4.25	[221]
PV‐BDTC1	PC_71_BM	1:2	−5.56	2.07	0.93	5.90	59.39	3.15	[249]
PBDTTPD2	PC_71_BM	1:1	−5.35	1.82	0.92	3.93	57	2.05	[276]
PBDTTPT1	PC_71_BM	1:1	−5.30	1.78	0.89	3.04	62	1.68	[276]
PBDT‐tt‐BT	PC_70_BM	1:1	−5.21	1.62	0.73	10.58	63.6	4.91	[297]
PBDTBTBTz	PC_70_BM	1:1	−5.12	1.96	0.82	9.01	60.3	4.46	[322]
PBDT‐TT‐IID^l^	PC_71_BM	1:1.5	−5.37	1.61	0.79	12.24	65	6.26	[324]
PBDTPhBT1	PC_71_BM	1:1	−5.35	1.98	0.90	2.40	50	1.08	[333]
PBDTPhBT2	PC_71_BM	1:1	−5.32	1.98	0.93	2.96	56	1.54	[333]
PBDT‐TPD‐DPP10%	PC_71_BM	1:2	−5.42	1.57	0.83	1.67	59	0.82	[334]
PBDT‐TPD‐DPP20%	PC_71_BM	1:2	−5.37	1.49	0.81	2.35	63	1.20	[334]
PBDT‐TPD‐DPP40%^m^	PC_71_BM	1:2	−5.25	1.34	0.67	5.02	53	1.78	[334]
P4^(n)^	PC_71_BM	1:3	−5.17		0.81	10.30	60	5.01	[335]

^A^ Weight ratio.

^B^ The HOMO energy level of polymer donors.

^C^ Estimated from the absorption edge in film ($E_{g}^{opt}=1240/\lambda_{onset}$).

^D^ Average *PCE* of OSCs.

^a^ 0.5% DIO.

^b^ 70°C annealing 15 min.

^c^ 100°C annealing 15 min.

^d^ SVA treatment.

^e^ 100°C annealing.

^f^ 150°C annealing.

^g^ 150°C annealing for 10 min.

^h^ 1% DIO.

^i^ 2% DIO.

^j^ 2.5% DIO.

^k^ DIO/THF.

^l^ 3% DIO.

^m^ 1.5% DIO (n) CB: CN = 98%: 2%.

Among D‐A type polymer donors, based on the main chain BDT‐TT, Chen et al.^[^ ^46^ ^]^ and Hou et al.^[^ ^47^ ^]^ first reported three polymer donors PBDTTT‐E (2009), PBDTTT‐C (2009) and PBDTTT‐CF (2009) by introducing side chains alkyloxy and carboxylate into the BDT and TT units in turn. After simply adjusting the side chain alkyl into the carboxylate group, the significantly enhanced *FF* (66.9%) and *J_sc_* (15.2 mA cm^−2^) exhibited by PBDTTT‐CF: PC_70_BM‐based OSC than PBDTTT‐E: PC_70_BM‐based and PBDTTT‐C: PC_70_BM‐based OSCs proves an effective way to increase the miscibility of polymer donors in the active layer. Similarly, a series of polymer donors PTB1‐PTB6 (2019) were also explored by Liang and co‐workers.^[^ ^48^ ^]^ Among them, PTB2 with the substitution of 1‐methoxyoctane obtained a high *J_sc_* (12.8 mA cm^−2^) and FF (66.3%) in PTB2: PC_61_BM‐based OSC, which further verified the above‐conclusion. As for PTB3, the introduction of alkyl chain octyl also further increases the *V_oc_* (0.74 V) in PTB3: PC_60_BM‐based OSC. In addition, based on the polymer backbone BDT‐T, P(BDT‐T) (2011), PABDT‐T (2013) and PBOT (2022) were synthesized by introducing different alkyloxy groups into the BDT unit; X1 (2022) was prepared by introducing the side chain carboxylate. Notably, compared with P(BDT‐T): PC_70_BM‐based and PABDT‐T: PC_71_BM‐based OSCs, PBOT (2022) with the substitution of 5‐(methoxymethyl) undecane into the BDT unit endows PBOT: BTP‐eC9‐based OSC with an obviously increased *J_sc_* (13.97 mA cm^−2^). Furthermore, X1 with the substitution of 2‐butyloctyl acetate also further enhances the *J_sc_* (27.62 mA cm^−2^) and *FF* in X1: BTP‐eC9‐based OSC and finally achieved an excellent *PCE* (16.5%).

Moreover, as for D‐A‐A type polymer donors, PBDTTT (2012),^[^ ^119^ ^]^ PBDT‐BT (2015),^[^ ^120^ ^]^ PB2T (2017),^[^ ^123^ ^]^ PBTzT‐4 (2018),^[^ ^127^ ^]^ PBTzT‐6 (2018),^[^ ^127^ ^]^ PBDTEH‐OD (2019),^[^ ^122^ ^]^ PBDTEH‐M (2019)^[^ ^122^ ^]^ and PBDTEH‐E (2019)^[^ ^122^ ^]^ were successfully prepared in turn. Interestingly, compared with PBDTTT: PCBM‐based OSC, due to the introduction of a long alkyl chain dodecyl, the enhanced miscibility of PBDT‐BT after blending with acceptor PC_71_BM effectively improves the *J_sc_* (5.86 mA cm^−2^) and *FF* (45.8%) in PBDT‐BT: PC_71_BM‐based OSC. Meanwhile, PBDTEH‐E with the substitution of longer alkyl chain into the carboxylate group also endows PBDTEH‐E: ITIC‐Th‐based OSC with a higher *J_sc_* (14.29 mA cm^−2^) and *FF* (58.2%). Likewise, the efficient *PCE* obtained by PBTzT‐4: PC_71_BM‐based (9.19%) and PBTzT‐6: PC_71_BM‐based (8.28%) OSCs were also proving the reasonable molecular design route by synergetic introducing alkyl and carboxylate groups into the BDT and acceptor units in turn.

Furthermore, based on the most widely applied D‐π‐A‐π type main‐chain engineering, PBDTPhBT1 (2010),^[^ ^333^ ^]^ PBDTPhBT2 (2010),^[^ ^333^ ^]^ PBDTTPT1 (2011),^[^ ^276^ ^]^ PBDTTPD2 (2011),^[^ ^276^ ^]^ PBDT‐TBT‐C6 (2011),^[^ ^154^ ^]^ PBDT‐TBT‐C8 (2011),^[^ ^154^ ^]^ PBDT‐TBT‐C10 (2011),^[^ ^154^ ^]^ PBDT‐TBT‐C12 (2011),^[^ ^154^ ^]^ O‐HD (2011),^[^ ^145^ ^]^ BO‐BO (2011),^[^ ^145^ ^]^ PU‐O (2011),^[^ ^145^ ^]^ PBDT‐TTBTT (2013),^[^ ^50^ ^]^ PBDT‐tt‐BT (2013),^[^ ^297^ ^]^ PDPPBDT (2014),^[^ ^147^ ^]^ P2(2014),^[^ ^163^ ^]^ POT‐DH (2014),^[^ ^135^ ^]^ PBDT‐TPD (2014),^[^ ^139^ ^]^ PBDT‐ttTPD (2014),^[^ ^139^ ^]^ P1 (2015),^[^ ^158^ ^]^ P2 (2015),^[^ ^158^ ^]^ PV‐BDTCl (2016),^[^ ^249^ ^]^ PBDTPH‐DTQx (2016),^[^ ^221^ ^]^ PBDTPHO‐DTQx (2016),^[^ ^221^ ^]^ P2 (2016),^[^ ^151^ ^]^ P11 (2019)^[^ ^118^ ^]^ and BDT‐T‐Qx‐T(P3) (2022)^[^ ^201^ ^]^ were subsequently prepared to further exploring the potential effect by introducing side chains alkyl, alkyloxy and alkylthio. Notably, due to the substitution of alkyloxy group further improves the electron‐donating of BDT unit, thus effectively optimizes the dissociation of exciton as well as hole/electron mobility, which endows PDPPBDT: PC_71_BM‐based and P11: PC_71_BM‐based OSCs with higher *J_sc_* and *FF* than O‐HD: PC_71_BM‐based, BO‐BO: PC_71_BM‐based and PU‐O: PC_71_BM‐based OSCs. Furthermore, benefiting introducing more suitable alkyl chain into the P11, the further enhanced *J_sc_* and *FF* endows P11: PC_71_BM‐based OSC (7.29%) with a higher *PCE* than PDPPBDT: PC_71_BM‐based OSC (3.28%). Interestingly, the above‐conclusions were also appeared in the characterization results between PBDTPhBT1: PC_71_BM‐based, PBDTPhBT2: PC_71_BM‐based, PBDTTPT1: PC_71_BM‐based and PBDTTPD2: PC_71_BM‐based OSCs. The better photovoltaic performance exhibited in PBDTTPT1: PC_71_BM‐based and PBDTTPD2: PC_71_BM‐based OSCs also verified the potential application of alkyloxy group. Moreover, compared with PBDT‐TBT‐C6: PC_61_BM‐based and PBDT‐TCT‐C10: PC_61_BM‐based OSCs, after adjusting the alkyl chain into the π‐bridge unit, the higher *J_sc_* and *FF* presented by PBDT‐TBT‐C8: PC_61_BM‐based and PBDT‐TBT‐C12: PC_71_BM‐based OSCs also remind us that adjusting the substitution of alkyl chain into π‐bridge unit is also a reasonable molecular design strategy to optimize the photoelectric performance in OSC devices. Meanwhile, the better *FF* (51%) achieved by PBDT‐TBT‐C12‐based OSC than PBDT‐TBT‐C10‐based OSC can be attributed to the formation of more ideal blend film morphology, with the *RMS* of 5.13 nm.

As for multicomponent and multi‐copolymerized polymer donors, PBDT‐TPD‐DPP10% (2012),^[^ ^334^ ^]^ PBDT‐TPD‐DPP20% (2012),^[^ ^334^ ^]^ PBDT‐TPD‐DPP40% (2012),^[^ ^334^ ^]^ P4 (2013)^[^ ^335^ ^]^ and PBDT‐TT‐IID (2016)^[^ ^324^ ^]^ were also synthesized to explore above side‐chain engineering. Benefiting the increased miscibility of polymer donors after introducing such functional side chains, corresponding OSC devices presented a high *FF*.

#### 3.2.3 Other types of side chains

**FIGURE 25:**
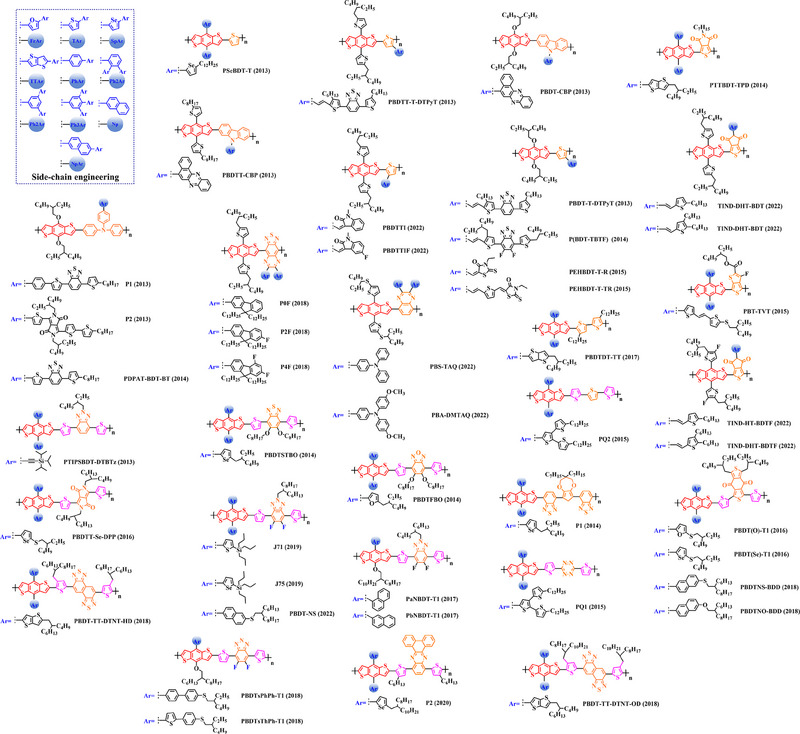
The detailed molecular structure of polymer donors with the substitution of unusual side chains.

It is undeniable that the photovoltaic performance of OSC devices can be effectively optimized by utilizing side‐chain engineering. Therefore, in order to further explore more efficient side‐chain engineering, the plenty of research has focus on constructing novel substituted groups, such as furan‐based, selenophene‐based and naphthalene‐based side chains. In this chapter, the potential relationship between detailed molecular structure of polymer donors (Figure 25) with unusual substitutions and photovoltaic parameters (Table 22) in corresponding OSC devices were shown as follows.

**TABLE 22:** Photovoltaic parameters of OSCs related to Figure 25.

Donor	Acceptor	D/A^A^	HOMO [eV] ^B^	$E_{g}^{opt}$ [eV] ^C^	*V_OC_* [V]	*J_SC_* [mA cm^−2^]	*FF* [%]	*PCE* [%] ^D^	Ref.
PSeBDT‐T	PC_71_BM	1:2	−5.14	1.96	0.72	7.73	46	2.73	[26]
PBDTT‐T‐DTPyT^a^	PCBM	1:3	−5.22	1.59	0.82	6.76	38	2.08	[30]
PBDT‐T‐DTPyT	PCBM	1:3	−5.38	1.74	0.78	4.47	38	1.32	[30]
P(BDT‐TBTF)	PC_70_BM	1:4	−5.36	1.90	0.88	11.23	57.3	5.29	[31]
PEHBDT‐T‐R	PC_71_BM	1:2	−5.48	1.86	0.99	8.61	47.50	3.89	[32]
PEHBDT‐T‐TR	PC_71_BM	1:1.5	−5.45	1.84	0.87	9.36	52.30	4.01	[32]
PBDTTI^b^	ITIC	1.5:1	−5.59	1.91	0.96	15.60	55	7.50	[39]
PBDTTIF^b^	ITIC	1.5:1	−5.60	1.89	0.98	13.38	55	7.60	[39]
PBT‐TVT	PC_71_BM	1:1.5	−5.23	1.63	0.81	15.16	62.71	8.04	[52]
TIND‐HT‐BDTF^c^, ^d^	Y6BO	3:3	−5.42	1.58	0.84	16.7	44.4	6.21	[63]
TIND‐DHT‐BDTF^c^, ^d^	Y6BO	3:4	−5.34	1.60	0.82	22.5	59.3	10.9	[63]
TIND‐HT‐BDT^c^, ^d^	Y6BO	3:3	−5.37	1.52	0.75	13.3	50.1	4.84	[63]
TIND‐DHT‐BDT^c^, ^d^	Y6BO	3:3	−5.26	1.54	0.76	24.8	56.2	10.5	[63]
PTTBDT‐TPD^e^	PC_71_BM	1:1	−5.52	1.85	0.91	10.69	62	6.03	[68]
P1^f^	PC_71_BM	1:2	−5.34	1.64	0.65	11.72	58.69	4.51	[72]
PBS‐TAQ	PC_71_BM	1:1.5	−5.35	1.77	0.85	12.64	47.93	5.24	[95]
PBS‐DMTAQ	PC_71_BM	1:1.5	−5.15	1.74	0.78	7.53	46.53	2.74	[95]
P0F^g^	PC_71_BM	1:2	−5.12	1.04	0.58	13.16	66	4.95	[98]
P2F^g^	PC_71_BM	1:2	−5.20	1.09	0.65	14.23	69	6.31	[98]
P4F^g^	PC_71_BM	1:2	−5.29	1.14	0.69	15.96	74	8.03	[98]
PBDT‐CBP	PC_71_BM	1:2	−5.50	1.56	0.81	6.97	41	2.33	[109]
PBDTT‐CBP	PC_71_BM	1:3	−5.50	1.55	0.71	4.05	30	0.90	[109]
P1(2013)	PC_71_BM	1:3	−5.57	1.93	0.86	9.40	39	3.09	[110]
P2(2013)	PC_71_BM	1:3	−5.53	1.74	0.80	10.77	35	2.95	[110]
PDPAT‐BDT‐BT^h^	PC_71_BM	1:4	−5.42	1.82	0.87	7.61	49.8	3.27	[111]
PBDTDT‐TT	ITM	1:1	−5.27	1.8	0.82	16.17	63.40	8.43	[121]
PQ1	PC_61_BM	1:3	−5.61	1.96	1.01	2.12	50.68	1.08	[136]
PQ2^i^	PC_71_BM	1:1.5	−5.28	1.97	0.80	7.68	62.05	3.80	[136]
PBDTT‐Se‐DPP^j^	PC_71_BM		−5.29	1.48	0.76	11.34	57.87	5.01	[150]
PBDTSTBO(P4)	PC_71_BM	1:2	−5.46	1.78	0.86	13.9	64	7.52	[156]
PTIPSBDT‐DTBTz^k^	PC_71_BM	1:1	−5.39	1.95	0.80	12.69	55	5.53	[173]
J71	m‐ITIC	1:1			0.94	17.53	71.20	11.45	[182]
J75^(^ ^l^, ^m^ ^)^	m‐ITIC	1:1	−5.49	1.93	0.96	17.11	69.47	11.27	[182]
PBDT‐NS^f^	LC301	1:1.2			0.848	21.30	66.4	11.99	[186]
PaNBDT‐T1	ITIC	1:1.5	−5.40	1.95	0.87	18.53	59.63	9.41	[187]
PbNBDT‐T1	ITIC	1:1.5	−5.32	1.90	0.74	18.19	50.02	6.52	[187]
PBDTsPhPh‐T1	ITIC	1:2	−5.35	1.98	0.863	15.73	65.1	8.85	[188]
PBDTsThPh‐T1	ITIC	1:1.5	−5.30	1.97	0.846	17.52	63.1	9.34	[188]
PBDTFBO^h^	PC_71_BM	1:2	−5.38	1.78	0.81	11.2	60	5.4	[190]
PBDT(O)‐T1	PC_70_BM	1:1	−5.31	1.74	0.83	9.1	49.5	4.0	[228]
PBDT(Se)‐T1	PC_70_BM	1:1	−5.53	1.80	0.91	12.6	71.7	8.4	[228]
PBDTNS‐BDD	ITIC	1:1.5	−5.35	1.81	0.87	13.49	60.05	6.89	[243]
PBDTNO‐BDD	ITIC	1:1.5	−5.27	1.83	0.94	14.86	66.47	9.18	[243]
P2(2020)^o^	PC_71_BM	1:2	−5.32	1.45	0.71	5.27	55	2.07	[263]
PBDT‐TT‐DTNT‐HD^n^	PC_71_BM	1:1	−5.48	1.52	0.81	11.34	43.61	3.99	[282]
PBDT‐TT‐DTNT‐OD^n^	PC_71_BM	1:1	−5.54	1.52	0.79	11.46	57.52	5.21	[282]

^A^ Weight ratio.

^B^ The HOMO energy level of polymer donors.

^C^ Estimated from the absorption edge in film ($E_{g}^{opt}=1240/\lambda_{onset}$).

^D^ Average *PCE* of OSCs.

^a^ inverted device.

^b^ 1% CB.

^c^ 0.5% CN.

^d^ 110°C annealing for 10 min.

^e^ 3% DIO.

^f^ 100°C annealing.

^g^ SVA treatment.

^h^ 1% DIO.

^i^ 90°C annealing for 10 min.

^j^ 2% DIO.

^k^ DIO.

^l^ 0.5% CN.

^m^ 130°C annealing for 2 min.

^n^ 0.5% DPE.

^o^ SVA treatment for 5 min.

Among D‐A type polymer donors, PSeBDT‐T (2013) was synthesized by introducing side chain 2‐dodecylselenophene into the BDT unit^[^ ^26^ ^]^; PBDT‐CBP (2013) and PBDTT‐CBP (2013) were prepared by introducing side chain benzo [*a*] phenazine into the acceptor unit^[^ ^109^ ^]^; PTTBDT‐TPD (2014) was designed to explore the potential application of side chain 2‐(2‐ethylhexyl) thieno [3, 2‐*b*] thiophene^[^ ^68^ ^]^; PBDTT‐T‐DTPyT (2013),^[^ ^30^ ^]^ PBDT‐T‐DTPyl (2013),^[^ ^30^ ^]^ P2 (2013),^[^ ^110^ ^]^ P(BDT‐TBTF) (2014),^[^ ^31^ ^]^ PDPAT‐BDT‐BT (2014),^[^ ^111^ ^]^ PBT‐TVT (2015),^[^ ^52^ ^]^ TIND‐DHT‐BDT (2022),^[^ ^63^ ^]^ TIND‐DHT‐BDT (2022),^[^ ^63^ ^]^ TIND‐HT‐BDTF (2022),^[^ ^63^ ^]^ TIND‐DHT‐BDTF (2022),^[^ ^63^ ^]^ PBDTT1 (2022)^[^ ^39^ ^]^ and PBDTT1F (2022)^[^ ^39^ ^]^ were synthesized by adjusting the conjugate plane and polarity of thiophene‐based side chains; P1 (2013),^[^ ^110^ ^]^ P0F (2018),^[^ ^98^ ^]^ P2F (2018)^[^ ^98^ ^]^ and P4F (2018)^[^ ^98^ ^]^ were constructed by introducing benzene‐based side chains; PEHBDT‐T‐R (2015),^[^ ^32^ ^]^ PEHBDT‐T‐TR (2015),^[^ ^32^ ^]^ PBS‐TAQ (2022)^[^ ^95^ ^]^ and PBS‐DMTAQ (2022)^[^ ^95^ ^]^ were prepared by innovatively introducing a series of common end‐capping groups 3‐methylene‐2‐thioxothiazolidin‐4‐one‐(R) (MTzR), triphenylamine (TPA) and triphenylamine‐(2R) (TPA2R) in SMD as side‐chain engineering, respectively. Benefiting such side chain with extended conjugated plane, thus close molecular stacking endows corresponding OSC devices with a high *V_oc_*, especially for PEHBDT‐T‐R: PC_71_BM‐based (0.99 V), PBDTTI: ITIC‐based (0.96 V) and PBDTTIF: ITIC‐based (0.98 V) OSCs. Meanwhile, with the deepen degree of fluorination, the enhanced electron‐withdrawing of acceptor unit also further accelerates the electron/hole mobility in the active layer, which explain the reason why P2F: PC_71_BM‐based and P4F: PC_71_BM‐based OSCs achieved a better photoelectric performance than P0F: PC_71_BM‐based OSC.

Furthermore, among D‐A‐A, D‐A‐A‐A and D‐π‐A‐π type polymer donors, PBDTDT‐TT (2017),^[^ ^121^ ^]^ PBDT‐TT‐DTNT‐HD (2018)^[^ ^282^ ^]^ and PBDT‐TT‐DTNT‐OD (2018)^[^ ^282^ ^]^ were synthesized by introducing TT‐based side chains; P1 (2014),^[^ ^72^ ^]^ PBDTSTBO (2014),^[^ ^156^ ^]^ PBDTT‐Se‐DPP (2016),^[^ ^150^ ^]^ PBDT(Se)‐T1 (2016),^[^ ^228^ ^]^ J75 (2019)^[^ ^182^ ^]^ and P2 (2020)^[^ ^263^ ^]^ were prepared by introducing selenophene‐based side chains; PBDTFBO (2014)^[^ ^190^ ^]^ and PBDT(O)‐T1 (2016)^[^ ^228^ ^]^ were constructed to explored the application of furan‐based side chains; PaNBDT‐T1 (2017),^[^ ^187^ ^]^ PbNBDT‐T1 (2017),^[^ ^187^ ^]^ PBDTNS‐BDD (2018),^[^ ^243^ ^]^ PBDTNO‐BDD (2018)^[^ ^243^ ^]^ and PBDT‐NS (2022)^[^ ^186^ ^]^ were innovatively introducing naphthalene‐based side chains. As for PBDTsPhPh‐T1 (2018),^[^ ^188^ ^]^ PBDTsThPh‐T1 (2018)^[^ ^188^ ^]^ and J71 (2019),^[^ ^182^ ^]^ which were designed by further introducing novel thiophene‐based or benzene‐based side chains. Among these donors, J71 and J75 innovatively introducing the side chain tripropylsilane. Similar to the function of alkyl chains, which also increases the miscibility of donor materials and finally the high *J_sc_* and *FF* endows J71: ITIC‐based (11.45%) and J75: ITIC‐based (11.27%) OSCs with a high *PCE*. Furthermore, after introducing naphthalene‐based side chain, compared with J71‐based and J75‐based OSC devices, the further enhanced *J_sc_* (21.30 mA cm^−2^) in PBDT‐NS: LC301‐based OSC verified the reasonable application of this side‐chain engineering.

Among these types of polymer donors, compared with PSeBDT‐T: PC_71_BM‐based (1.8 nm), P1: PC_71_BM‐based (1.3 nm), PBS‐TAQ: PC_71_BM‐based (2.59 nm) and PBS‐DMTAQ: PC_71_BM‐based (1.77 nm) blend films, the decreased *RMS* of J71: m‐ITIC‐based (0.773 nm) and J75: m‐ITIC‐based (0.404 nm) blend films endows corresponding OSCs with a higher *FF* of 71.20% and 69.47% in turn, which can be attributed to the formation of a more ideal active layer morphology.

All in all, excellent molecular stacking, fine blending morphology and fast dissociation and transport of excitons are the key factors to prepare efficient photovoltaic devices, which are closely related to the three photovoltaic elements *V_oc_*_,_ *J_sc_* and *FF*. However, it may be easier to improve a single photovoltaic parameter by side‐chain engineering, but the damage to other photovoltaic performance often leads to a decrease in final *PCE*. Therefore, how to achieving synergistic optimizing photovoltaic performance in OSC devices is the primary exploration goal of side‐chain engineering in future.

### 3.3 Other molecular design engineering

#### 3.3.1 Multicomponent copolymerized donors

**FIGURE 26:**
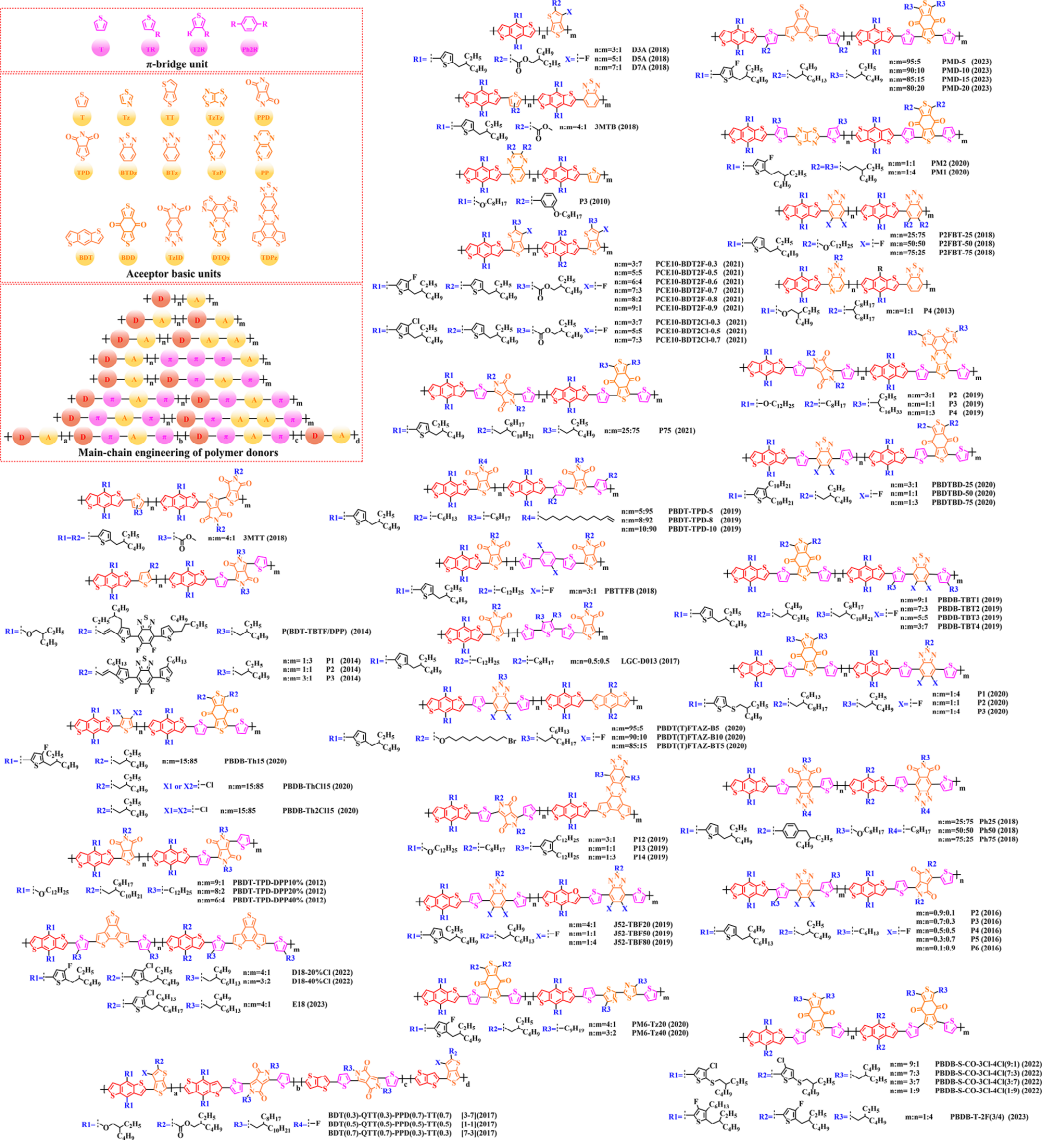
The detailed molecular structure of multicomponent copolymerized donors.

Indeed, based on the above molecular design strategy of main‐chain and side‐chain engineering, a great breakthrough has been made in the research of polymer donors so far, with the highest *PCE* has exceeded 19%. In order to further develop efficient polymer donors, by utilizing the advantages of various reported high‐efficiency polymer donors in photovoltaic devices, multi‐component copolymerization is gradually becoming another mainstream molecular design strategy. For example, PM1‐based (*PCE *= 17.3%) and PM6‐TZ40‐based (*PCE *= 17.1%) OSCs both achieved a high *PCE*. In this section, the detailed molecular structure (Figure 26) of multi‐copolymerized polymer donors and photovoltaic parameters (Table 23) in corresponding OSCs were discussed as follows.

**TABLE 23:** Photovoltaic parameters of OSCs related to Figure 26.

Donor	Acceptor	D/A^A^	HOMO [eV] ^B^	$E_{g}^{opt}$ [eV] ^C^	*V_OC_* [V]	*J_SC_* [mA cm^−2^]	*FF* [%]	*PCE* [%] ^D^	Ref.
P(BDT‐TBTF/DPP)	PC_70_BM	1:2	−5.25	1.57	0.74	13.15	36.0	3.22	[31]
PCE10‐BDT2F‐0.3	Y6		−5.31	1.58	0.678	26.10	66.56	11.55	[45]
PCE10‐BDT2F‐0.5	Y6		−5.35	1.58	0.720	26.01	69.42	12.78	[45]
PCE10‐BDT2F‐0.6	Y6		−5.37	1.58	0.739	26.01	64.08	12.30	[45]
PCE10‐BDT2F‐0.7	Y6		5.40	1.59	0.746	26.17	68.95	13.39	[45]
PCE10‐BDT2F‐0.8	Y6		−5.42	1.59	0.753	26.36	69.45	13.66	[45]
PCE10‐BDT2F‐0.9	Y6		−5.45	1.59	0.766	25.14	67.71	12.89	[45]
PCE10‐BDT2Cl‐0.3	Y6		−5.33	1.59	0.700	23.56	66.24	10.77	[45]
PCE10‐BDT2Cl‐0.5	Y6		−5.36	1.60	0.724	23.37	64.41	11.62	[45]
PCE10‐BDT2Cl‐0.7	Y6		−5.41	1.60	0.743	24.85	65.58	11.93	[45]
P3	PC_70_BM	1:4	−5.20	1.60	0.71	6.41	51.7	2.35	[97]
P12(2019)^a^	PC_71_BM	1:1.5	−5.24	1.30	0.83	14.36	66	7.81	[118]
P13(2019)^a^	PC_71_BM	1:1.5	−5.26	1.16	0.86	15.74	68	9.13	[118]
P14(2019)^a^	PC_71_BM	1:1.5	−5.28	1.15	0.88	14.78	66	8.53	[118]
P2(0.9:0.1)	PC_71_BM		−5.29	1.58	0.74	14.70	64.4	6.72	[152]
P3(0.7:0.3)	PC_71_BM		−5.28	1.52	0.73	18.45	66.9	8.80	[152]
P4(0.5:0.5)	PC_71_BM		−5.26	1.49	0.72	18.52	62.2	8.01	[152]
P5(0.3:0.7)	PC_71_BM		−5.24	1.47	0.71	15.52	61.3	6.45	[152]
P6(0.1:0.9)	PC_71_BM		−5.21	1.44	0.71	13.65	61.1	5.74	[152]
PBDTBD‐25^b^	IT‐4F	1:1.5	−5.53	1.79	0.83	11.96	42.16	3.99	[169]
PBDTBD‐50^b^	IT‐4F	1;1.5	−5.51	1.74	0.80	17.16	73.10	9.70	[169]
PBDTBD‐75^b^	IT‐4F	1:1.5	−5.49	1.72	0.75	17.89	70.70	9.14	[169]
J52‐TBF20	ITIC	1:1	−5.29	1.92	0.803	17.68	70.5	9.92	[179]
J52‐TBF50	ITIC	1:1	−5.27	1.91	0.790	17.93	72.8	10.18	[179]
J52‐TBF80	ITIC	1:1	−5.24	1.91	0.756	16.76	66.6	8.30	[179]
PBDB‐S‐CO‐3Cl‐4Cl(7:3)	Y6	1:1.2	−5.37	1.82	0.90	22.90	61	12.57	[233]
PBDB‐S‐CO‐3Cl‐4Cl(3:7)	Y6	1:1.2	−5.34	1.81	0.92	21.90	62	12.50	[233]
PBDB‐S‐CO‐3Cl‐4Cl(1:9)	Y6	1:1.2	−5.33	1.81	0.87	22.06	65	12.46	[233]
PBDB‐S‐CO‐3Cl‐4Cl(9:1)	Y6	1:1.2	−5.39	1.81	0.90	23.60	63	13.34	[233]
PBDB‐T‐2F(3/4)	Y6‐HU		−5.57	1.81	0.86	23.78	70	13.62	[237]
Ph25^c^	ITIC	1:1.5	−5.29	1.87	0.86	16.11	59.44	8.14	[252]
Ph50^c^	ITIC	1:1.5	−5.30	1.89	0.87	15.87	61.97	8.43	[252]
Ph75^c^	ITIC	1:1.5	−5.32	1.90	0.89	15.45	56.08	7.58	[252]
P2(2019)	PC_71_BM		−5.21	1.31	0.82	14.07	66	7.61	[255]
P3(2019)	PC_71_BM		−5.17	1.27	0.76	17.21	71	9.13	[255]
P4(2019)	PC_71_BM		−5.11	1.24	0.74	16.65	65	8.00	[255]
PM1(X:Y = 4:1)^d^	Y6	1:1.25	−5.52	1.86	0.87	25.9	78	17.3	[262]
PM2(X:Y = 1:1)^d^	Y6	1:1.25	−5.60	1.89	0.90	24.9	69	15.2	[262]
PBDT(T)FTAZ‐B5^e^, ^f^	N2200	1.5:1	−5.27	1.96	0.80	13.11	60.11	6.30	[332]
PBDT(T)FTAZ‐B10^e^, ^f^	N2200	1.5:1	−5.27	1.95	0.80	11.57	59.39	5.50	[332]
PBDT(T)FTAZ‐B15^e^, ^f^	N2200	1.5:1	−5.27	1.96	0.80	11.28	59.95	5.41	[332]
PBDT‐TPD‐DPP10%	PC_71_BM	1:2	−5.42	1.57	0.83	1.67	59	0.82	[334]
PBDT‐TPD‐DPP20%	PC_71_BM	1:2	−5.37	1.49	0.81	2.35	63	1.20	[334]
PBDT‐TPD‐DPP40%^g^	PC_71_BM	1:2	−5.25	1.34	0.67	5.02	53	1.78	[334]
P4^h^	PC_71_BM	1:3	−5.17		0.81	10.30	60	5.01	[335]
P2FBT‐25^a^	PC_71_BM	1:1.5	−5.41	1.75	0.89	10.52	54	4.95	[336]
P2FBT‐50^a^	PC_71_BM	1:1.5	−5.23	1.73	0.89	9.53	52	4.36	[336]
P2FBT‐75^a^	PC_71_BM	1:1.5	−5.31	1.73	0.89	11.39	56	5.33	[336]
D3A	ITIC	1:1.5			0.87	16.58	65	9.12	[337]
D5A	ITIC	1:1.5	−5.37		0.89	17.24	67	10.12	[337]
D7A	ITIC	1:1.5			0.88	16.71	62	8.88	[337]
3MTB	ITIC	1:1.5	−5.45	1.78	0.92	16.34	54.9	8.27	[338]
3MTT	ITIC	1.2:1	−5.50	1.87	1.00	15.67	51.47	8.04	[338]
P1	PC_61_BM	1:2	−5.29	1.43	0.72	6.16	49	2.16	[339]
P2	PC_61_BM	1:2	−5.36	1.47	0.74	12.21	46	4.17	[339]
P3	PC_61_BM	1:2	−5.47	1.52	0.78	14.13	48	5.29	[339]
PBDT‐TPD‐5^i^	PNDI‐T‐5	1.5:1	−5.32	1.80	0.79	2.7	46.1	0.98	[340]
PBDT‐TPD‐8^i^	PNDI‐T‐5	1.5:1	−5.31	1.80	0.81	7.6	54.3	3.34	[340]
PBDT‐TPD‐10^i^	PNDO‐T‐5	1.5:1	−5.31	1.79	0.80	5.5	51.3	2.26	[340]
PBDB‐Th15	Y6	1:1.2	−5.36		0.83	24.80	72.33	14.54	[341]
PBDB‐ThCl15	Y6	1:1.2	−5.41		0.84	25.21	74.03	15.42	[341]
PBDB‐Th2Cl15	Y6	1:1.2	−5.45		0.87	24.93	58.10	12.22	[341]
LGC‐D013^j^	PC_71_BM	1:2	−5.56	1.84	0.87	11.29	73.13	6.99	[342]
PBTTFB^k^	PC_71_BM	1:1.5	−5.58	1.89	0.88	14.51	65	7.78	[343]
PBDB‐TBT1	ITIC	1:1	−5.30	1.79	0.86	16.84	62.85	9.09	[344]
PBDB‐TBT2	ITIC	1:1	−5.33	1.78	0.88	15.50	58.26	7.95	[344]
PBDB‐TBT3	ITIC	1:1	−5.35	1.77	0.91	11.45	56.91	5.93	[344]
PBDB‐TBT4	ITIC	1:1	−5.36	1.75	0.90	4.12	56.80	2.12	[344]
P1(2020)	ITIC		−5.25	1.87	0.946	15.94	57.2	8.62	[345]
P2(2020)	ITIC		−5.26	1.87	0.925	15.90	57.2	8.41	[345]
P3(2020)	ITIC		−5.19	1.93	0.913	16.87	59.2	9.12	[345]
P75	Y6	1:1.5	−5.43		0.81	22.6	58	10.28	[346]
D18‐20%Cl	Y6	1:1.6	−5.52	1.97	0.861	27.20	78.06	18.12	[347]
D18‐40%Cl	Y6	1:1.6	−5.53	1.98	0.866	26.34	75.46	17.05	[347]
E18	BTA3‐4F	1:1	−5.54		1.30	10.95	68.66	9.89	[347]
PMD‐5	L8‐BO	1:1.2	−5.49	1.81	0.892	25.16	76.61	17.19	[348]
PMD‐10	L8‐BO	1:1.2	−5.50	1.82	0.895	25.44	76.72	17.47	[348]
PMD‐15	L8‐BO	1:1.2	−5.51	1.82	0.899	25.64	77.01	17.75	[348]
PMD‐20	L8‐BO	1:1.2	−5.52	1.83	0.907	25.53	76.14	17.26	[348]
PM6‐TZ20(n:m = 80:20)^l^	Y6	1:1	−5.46	1.84	0.86	26.2	75	17.3	[349]
PM6‐TZ40(n:m = 60:40)^m^	Y6	1:1	−5.46	1.84	0.85	24.8	72	15.3	[349]
[3, 4, 5, 6, 7]^j^	PC_71_BM	1:2	−5.38	1.37	0.681	19.1	65.2	9.18	[350]
[1–1]^j^	PC_71_BM	1:2	−5.41	1.43	0.664	18.4	65.0	8.10	[350]
[7–3]^j^	PC_71_BM	1:1.5	−5.42	1.48	0.669	16.8	60.7	7.05	[350]

^A^ Weight ratio.

^B^ The HOMO energy level of polymer donors;

^C^ Estimated from the absorption edge in film ($E_{g}^{opt}=1240/\lambda_{onset}$).

^D^ Average *PCE* of OSCs.

^a^ SVA treatment.

^b^ 2% DPE.

^c^ 0.5% DBE.

^d^ 0.75% CN.

^e^ 100°C annealing for 10 min.

^f^ 0.5% DIO.

^g^ 1.5% DIO.

^h^ CB: CN = 98%: 2%.

^i^ 150°C annealing for 1.5 h.

^j^ 3% DIO.

^k^ DPE.

^l^ Ag cathode.

^m^ Al cathode.

In 2010, Yuan and co‐workers selected thiophene and pyrido [3, 4‐*b*] pyrazine (PP) as acceptor units, constructing the novel main chain engineering (BDT‐T)‐(BDT‐PP) by combining polymer backbone BDT‐T and BDT‐PP.^[^ ^97^ ^]^ Meanwhile, through controlling the ratio of BDT‐T and BDT‐PP to be 1: 1, P3 (2010) was successfully synthesized. Furthermore, after blending with the acceptor PC_70_BM, P3: PC_70_BM‐based OSC obtained the *V_oc_* of 0.71 V, *J_sc_* of 6.41 mA cm^−2^, *FF* of 51.7% and *PCE* of 2.35%, respectively. Subsequently, P4 (2013) was also explored by Kotowski et al.^[^ ^335^ ^]^, which was also designed based on the polymer skeleton (BDT‐TzP)‐(BDT‐T) with the ratio of BDT‐TzP and BDT‐T was 1: 1. Due to the introduction of acceptor units with a stronger electronegative, the photoelectric performance in P4: PC_71_BM‐based OSC has significantly optimized, with the *V_oc_* of 0.81 V, *J_sc_* of 10.30 mA cm^−2^, *FF* of 60% and *PCE* of 5.01%. Similarly, based on the polymer backbone (BDT‐BTz)‐(BDT‐BTz), P2FBT‐25 (2018), P2FBT‐50 (2018) and P2FBT‐75 (2018) were prepared by Li et al.^[^ ^336^ ^]^; PCE10‐BDT2F‐0.3 (2021), PCE10‐BDT2F‐0.5 (2021), PCE10‐BDT2F‐0.6 (2021), PCE10‐BDT2F‐0.7 (2021), PCE10‐BDT2F‐0.8 (2021), PCE10‐BDT2F‐0.9 (2021), PCE10‐BDT2Cl‐0.3 (2021), PCE10‐BDT2Cl‐0.3 (2021) and PCE10‐BDT2Cl‐0.7 (2021) with different copolymerized ratio were also explored by Huang et al.^[^ ^45^ ^]^ Among these donors, PCE10‐BDT2F‐0.8: Y6‐based OSC achieved the best photoelectric performance, with the *V_oc_* of 0.753 V, *J_sc_* of 26.36 mA cm^−2^, *FF* of 69.45% and *PCE* of 13.66%, which reminds us that adjusting copolymerized ratio is also a reasonable molecular design route. Furthermore, the high *V_oc_* exhibited in P2FBT‐25: PC_71_BM‐based (0.89 V), P2FBT‐50: PC_71_BM‐based (0.89 V) and P2FBT‐75: PC_71_BM‐based (0.89 V) OSCs were further proving the above‐conclusion. As for other types of multicomponent copolymerized donors, Kim and co‐workers innovatively adjusted the copolymerization ratio of BDT and TT units, synthesizing a series of novel D‐A type copolymerized donors D3A (2018), D5A (2018) and D7A (2018)^[^ ^337^ ^]^; Hoang et al. also prepared 3MTT (2018) based on the main‐chain engineering (BDT‐T)‐(BDT‐TPD‐TPD).^[^ ^338^ ^]^

In addition, the plenty of research has begun to focus on copolymerizing D‐A and D‐π‐A‐π type polymer donors. In 2012, PBDT‐TPD‐DPP10% (2012), PBDT‐TPD‐DPP20% (2012) and PBDT‐TPD‐DPP40% (2012) were first reported by Zhang et al.^[^ ^334^ ^]^ Regrettably, the mismatched absorption after blending with acceptor PC_71_BM leads to the extremely low *J_sc_* in PBDT‐TPD‐DPP10%: PC_71_BM‐based (1.67 mA cm^−2^), PBDT‐TPD‐DPP20%: PC_71_BM‐based (2.35 mA cm^−2^) and PBDT‐TPD‐DPP40%: PC_71_BM‐based (5.02 mA cm^−2^) OSCs. Subsequently, P(BDT‐TBTF/DPP) (2014),^[^ ^31^ ^]^ P1 (2014),^[^ ^339^ ^]^ P2 (2014),^[^ ^339^ ^]^ P3 (2014),^[^ ^339^ ^]^ P12 (2019),^[^ ^118^ ^]^ P13 (2019),^[^ ^118^ ^]^ P14 (2019),^[^ ^118^ ^]^ PBDT‐TPD‐5 (2019),^[^ ^340^ ^]^ PBDT‐TPD‐8 (2019), ^[^ ^340^ ^]^ PBDT‐TPD‐10 (2019),^[^ ^340^ ^]^ PBDT(T)FTAZ‐B5 (2020),^[^ ^332^ ^]^ PBDT(T)FTAZ‐B10 (2020),^[^ ^332^ ^]^ PBDT(T)FTAZ‐BT5 (2020),^[^ ^332^ ^]^ PBDB‐Th15 (2020),^[^ ^341^ ^]^ PBDB‐ThC115 (2020)^[^ ^341^ ^]^ and PBDB‐Th2Cl15 (2020)^[^ ^341^ ^]^ were also successfully prepared in turn. Notably, with the adjustment of π‐bridge and acceptor units, the optimized light absorption ability as well as finer blend film morphology endows such OSCs with an increased *J_sc_* and *FF*. Among them, the photovoltaic performance in PBDB‐Th15: Y6‐based and PBPB‐ThC115: Y6‐based OSCs both achieved an obvious breakthrough, the *PCE* were respectively reached at 14.54% and 15.42%. Moreover, two novel copolymerized donors LGC‐D013 (2017)^[^ ^342^ ^]^ and PBDTTFB (2018)^[^ ^343^ ^]^ constructed based on the polymer skeleton (BDT‐TPD)‐(BDT‐T‐PPD‐T) were also obtained a higher *J_sc_* and *FF* in corresponding OSC devices.

Besides the molecular design strategy of (D‐A)‐(D‐π‐A‐π) type copolymerized main‐chain engineering, constructing (D‐π‐A‐π)‐(D‐π‐A‐π) type copolymerized polymer backbone was also attracted the attention of researchers. In 2016, P2 (2016), P3 (2016), P4 (2016), P5 (2016) and P6 (2016) were first reported by Xu et al.^[^ ^152^ ^]^ Among them, P3 with the ratio of polymer backbone BDT‐TR‐BTDz‐TR at 70% and BDT‐T‐PPD‐T at 30% exhibited the best *PCE* (8.80%). Similarly, among Ph‐25 (2018),^[^ ^336^ ^]^ Ph50 (2018)^[^ ^336^ ^]^ and Ph75 (2018)^[^ ^336^ ^]^, Ph50 with the copolymerized ratio of 1: 1 also presented the best photovoltaic performance in Ph50: ITIC‐based OSC. Afterward, J52‐TBF20 (2019), J52‐TBF50 (2019) and J52‐TBF80 (2019) were reported by Wang et al.^[^ ^179^ ^]^; P2 (2019), P3 (2019) and P4 (2019) were synthesized by Keshtov et al.^[^ ^255^ ^]^; PBDB‐TBT1 (2019), PBDB‐TBT2 (2019), PBDB‐TBT3 (2019), PBDB‐TBT3 (2019) and PBDB‐TBT4 (2019) were prepared by Wu et al.^[^ ^344^ ^]^; PM1 (2020) and PM2 (2020) were designed by Wu et al.^[^ ^262^ ^]^; P1 (2020), P2 (2020), P3 (2020) were explored by Jing et al.^[^ ^345^ ^]^; PBDTBD25 (2020), PBDTBD50 (2020) and PBDTBD75 (2020) were synthesized by Jung et al.^[^ ^169^ ^]^; P75 (2021) was also designed by Ji et al.^[^ ^346^ ^]^ Recently, a series of novel copolymerized donors PBDB‐S‐CO‐3Cl‐4Cl (9:1) (2022),^[^ ^233^ ^]^ PBDB‐S‐CO‐3Cl‐4Cl (7:3),^[^ ^233^ ^]^ PBDB‐S‐CO‐3Cl‐4Cl (3:7) (2022),^[^ ^233^ ^]^ PBDB‐S‐CO‐3Cl‐4Cl (1:9) (2022),^[^ ^233^ ^]^ D18‐20%Cl (2022),^[^ ^347^ ^]^ D18‐40%Cl (2022),^[^ ^347^ ^]^ E18 (2023),^[^ ^347^ ^]^ PMD‐5 (2023),^[^ ^348^ ^]^ PMD‐10 (2023),^[^ ^348^ ^]^ PMD‐15 (2023),^[^ ^348^ ^]^ PMD‐20 (2023)^[^ ^348^ ^]^ and PBDB‐T‐2F (3/4) (2023)^[^ ^237^ ^]^ were also successfully synthesized in turn. Among them, we can easily find that appropriate copolymerization ratio is an essential factor to design an ideal copolymerized donor. Furthermore, a series of novel multicomponent copolymerized donors with acceptor units BDD and DTTz presented a more efficient *PCE* in OSC devices than others, especially for PM1: Y6‐based (17.3%), PMD‐15: L8‐BO‐based (17.75%) OSC and D18‐20%Cl: Y6‐based (18.12%) OSCs.

In addition, considering the success of multicomponent copolymerized donors with acceptor unit BDD, Guo and co‐workers synthesized PM6‐Tz20 (2020) and PM6‐Tz40 (2020) based on the main chain (BDT‐T‐BDD‐T)‐(BDT‐T‐Tz‐Tz‐T).^[^ ^349^ ^]^ Surprisingly, PM6‐Tz20 with the similar molecular structure of PM1, which was synthesized by adjusting the blending ratio to be 4: 1 and finally the *V_oc_*, *J_sc_*, *FF* and *PCE* in PM6‐Tz20: Y6‐based OSC were reached at 0.86 V, 26.2 mA cm^−2^, *FF* of 75% and 17.3%, respectively. As for three novel component copolymerized donors [3–7] (2017), [1–1] (2017) and [7–3] (2017), which were designed by innovatively adjusting the BDT, QTT, PPD or TT units in polymer backbone.^[^ ^350^ ^]^ Although the OSC devices based on such donor materials have not achieved ideal photovoltaic performance, this innovative molecular design strategy also deserves further attention.

Among these multicomponent polymer donors, the excellent *Td_5%_* obtained by PBDTBD‐25 (391°C), PBDTBD‐50 (395°C), PBDTBD‐75 (404°C) and LGC‐D013 (422°C) exhibiting a high stability. Furthermore, based on the characterization result of morphology, PBDTBD‐50: IT‐4F‐based (0.77 nm), PBDTBD‐75: IT‐4F‐based (0.91 nm), J52‐TBF20: ITIC‐based (1.11 nm), J52‐TBF50: ITIC‐based (1.40 nm), PBDB‐Th15: Y6‐based (2.99 nm), PBDB‐ThC115: Y6‐based (2.68 nm), D18‐20%Cl: Y6‐based (0.84 nm), D18‐40%: Y6‐based (0.86 nm), PMD‐5: L8‐BO‐based (0.925 nm), PMD‐10: L8‐BO‐based (0.919 nm), PMD‐15: L8‐BO‐based (0.863 nm) and PMD‐20: L8‐BO‐based (1.06 nm), PM6‐TZ20: Y6‐based (1.81 nm) and PM6‐TZ40: Y6‐ased (1.04 nm) blend films obtained a suitable *RMS*, with the excellent *FF* of 73.10%, 70.70%, 70.5%, 72.8%, 72.33%, 74.03%, 73.13%, 78.06%, 75.46%, 76.61%, 76.72%, 77.01%, 76.14%, 75% and 72%, which can be attributed the formation of an ideal blend film morphology.

#### 3.3.2 BDT‐derived donor units

**FIGURE 27:**
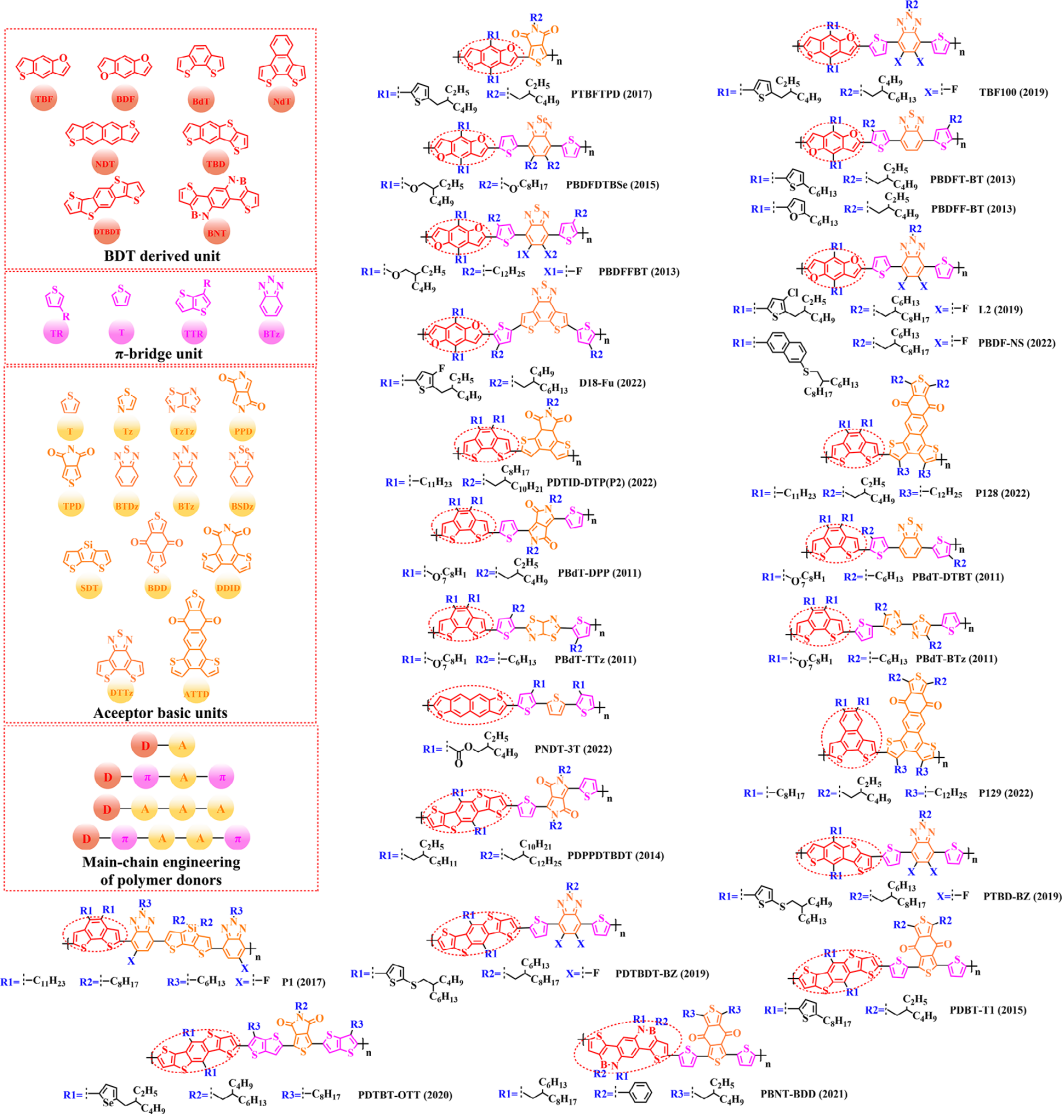
The detailed molecular structure of polymer donors synthesized by introducing BDT derivatives as donor units.

Due to the excellent ability of lone pair donor exhibited by “S” atoms in BDT unit effectively optimizes the π‐π conjugated structure and electron‐donating, thus BDT as an ideal donor core unit, has made a great breakthrough in the field of polymer donors. Therefore, based on the success obtained by BDT‐based donor materials, a series of novel polymer donors with BDT‐derived donor units were also successfully synthesized, which have also achieved excellent photovoltaic performance in corresponding OSC devices, such as D18‐Fu based OSC (16.75%) and PBDF‐NS based OSC (15.70%). In this section, the detailed molecular structure (Figure 27) of representative polymer donors with BDT‐derived donor unit and photovoltaic parameters (Table 24) in corresponding OSC devices were shown as follows.

**TABLE 24:** Photovoltaic parameters of OSCs related to Figure 27.

Donor	Acceptor	D/A^A^	HOMO [eV] ^B^	$E_{g}^{opt}$ [eV] ^C^	*V_OC_* [V]	*J_SC_* [mA cm^−2^]	*FF* [%]	*PCE* [%] ^D^	Ref.
PTBFTPD^(^ ^a^, ^b^ ^)^	PC_71_BM	1:1.5	−5.64	1.83	1.09	7.51	52.9	4.33	[70]
PDTID‐DTB(P2)^c^	PThIND‐Cl	1:1.2	−5.67	2.07	1.02	13.36	53	7.04	[115]
P1(2017)^d^	PC_71_BM	1:2	−5.28	1.83	0.88	12.21	61	6.83	[130]
PDPPDTBDT	PC_71_BM	1:2	−5.35	1.78	0.743	9.71	65	4.69	[147]
PDBT‐T1^e^	IDIC	1:1	−5.36		0.834	16.68	73.2	10.16	[159]
PDTBDT‐BZ	BDTB‐Ph	1:1.25	−5.32	1.91	0.883	13.20	53.9	6.19	[178]
PTBD‐BZ	TBDB‐Na	1:1	−5.40	1.90	0.905	19.50	69.2	12.25	[178]
TBF100	ITIC	1:1	−5.22	1.91	0.745	15.57	66.2	7.52	[179]
PBDF‐NS^f^	LC301	1:1.2	−5.44	1.87	0.840	23.70	74.2	14.82	[186]
PBDF‐NS^g^	PY‐IT	1:1.2	−5.44	1.87	0.852	25.00	74.2	15.70	[186]
PBDFT‐BT^h^	PC_71_BM	1:1	−5.08	1.68	0.73	9.94	60.9	4.42	[265]
PBDFF‐BT	PC_71_BM	1:1	−5.11	1.61	0.80	5.84	55.6	2.60	[265]
PBDFFBT	PC_71_BM	1:3	−5.33	1.60	0.62	9.17	58.4	3.3	[269]
PNDT‐3T	ITCPTC	1:1	−5.39	1.99	0.96	15.90	63.70	9.65	[293]
PDTBT‐OTT	PC_71_BM	1:4	−5.36	1.99	0.86	9.79	71.1	6.00	[308]
PBDFDTBSe	PC_71_BM	1:1	−5.27	1.60	0.71	10.37	44.6	3.28	[351]
L2	TTPT‐T‐4F		−5.50		0.86	22.17	73.1	13.68	[352]
D18‐Fu	PC_71_BM		−5.48	1.97	0.96	9.32	61.2	5.49	[353]
D18‐Fu	Y6‐1O		−5.48	1.97	0.89	23.72	77.3	16.20	[353]
P128^i^	Y6	1:1.2	−5.66	2.21	0.92	11.52	59	6.06	[354]
P129^i^	Y6	1:1.2	−5.56	2.13	0.86	23.58	71	14.33	[354]
PBdT‐DPP	PC_70_BM	1:3	−5.01	1.41	0.71	3.28	56.1	1.31	[355]
PBdT‐DTBT	PC_70_BM	1:3	−5.09	1.70	0.80	4.58	30.7	1.13	[355]
PBdT‐TTz	PC_70_BM	1:3	−5.19	1.91	0.90	5.50	68.7	3.40	[355]
PBdT‐BTz	PC_70_BM	1:3	−5.16	2.01	0.88	5.20	61.3	2.81	[355]
PBNT‐BDD^j^	Y6‐BO	1:1.2	−5.52		0.88	25.4	72	15.9	[356]

^A^ Weight ratio.

^B^ The HOMO energy level of polymer donors.

^C^ Estimated from the absorption edge in film ($E_{g}^{opt}=1240/\lambda_{oset}$).

^D^ Average *PCE* of OSCs.

^a^ 3% DIO.

^b^ 100°C annealing.

^c^ THF SVA treatment for 40s.

^d^ DIO.

^e^ 0.25% DIO.

^f^ 100°C annealing.

^g^ 80°C annealing.

^h^ 3% DIO.

^i^ SVA treatment.

^j^ 3% DIO and 2% DPE.

In 2013, Zhang and co‐workers introduced O atom to replace “S” atom of BDT unit, designing a novel donor unit BDF.^[^ ^265^ ^]^ Afterward, based the main chain BDF‐T‐BTz‐T, PBDFT‐BT (2013) and PBDFF‐BT (2013) were successfully synthesized in subsequence. After blending with the acceptor PC_71_BM, PBDFT‐BT: PC_71_BM‐based and PBDFF‐BT: PC_71_BM‐based OSCs obtained the *V_oc_* of 0.73 and 0.80 V, *J_sc_* of 9.94 and 5.84 mA cm^−2^, *FF* of 60.9% and 55.6% as well as *PCE* of 4.42% and 2.60%, respectively. Similarly, PBDFFBT (2013) was explored by Xiao et al.,^[^ ^269^ ^]^ which was prepared by further introducing two “F” atoms into the BTDz unit; PBDFDTBSe (2015) was synthesized by introducing BSDz as acceptor unit^[^ ^351^ ^]^; L2 (2019)^[^ ^352^ ^]^ and PBDF‐NS (2020)^[^ ^186^ ^]^ were designed by introducing BTz as acceptor unit. As for D18‐Fu (2020),^[^ ^353^ ^]^ which was synthesized based on the main‐chain engineering BDF‐TR‐DTTz‐TR. Among these donors, with the enhancement of electronegativity of acceptor units, wider and more efficient complementary light absorption was exhibited by D18‐Fu: Y6‐1O‐based, L2: TTPT‐T‐4F‐based, and PBDF‐NS: PY‐1T‐based OSCs, which can be attributed to the significantly optimized exciton dissociation and hole/electron mobility. Among them, the efficient *J_sc_* in D18‐Fu: Y6‐1O‐based (23.72 mA cm^−2^), L2: TTPT‐T‐4F‐based (22.17 mA cm^−2^) and PBDF‐NS: PY‐IT‐based (25.00 mA cm^−2^) OSCs further verified the above‐conclusion. Moreover, PTBFTPD (2017)^[^ ^70^ ^]^ and TBF100 (2019)^[^ ^179^ ^]^ were designed by introducing TBF as donor unit. After applying for OSC devices, PTFTPD: PC_71_BM‐based OSC exhibited a promising *V_oc_* (1.09 V), as well as TBF100: ITIC‐based OSC with a significantly enhanced *J_sc_* (15.57 mA cm^−2^) and *FF* (66.2%).

In addition, after adjusting the binding site of thiophene and benzene units, a series of novel polymer donors designed by introducing the donor unit benzo [2, 1‐*b*: 3, 4‐*b*’] dithiophene (BdT) were also successfully synthesized. In 2011, P128 (2011) was first reported by Keshtov and co‐workers^[^ ^354^ ^]^; a series of novel polymer donors PBdT‐DTBT (2011), PBdT‐DPP (2011), PBdT‐TTz (2011) and PBdT‐BTz (2011) were also prepared by Zhang et al.^[^ ^355^ ^]^ After applying for OSC devices, the formation of highly π‐π conjugated structure endows such OSCs with a high *V_oc_*, especially for P128: Y6‐based (0.92 V) and PBdT‐TTz: PC_70_BM‐based (0.90 V) OSCs. Furthermore, P1 (2017) and PDTID‐DTB(P2) (2022) were also successfully synthesized by Keshtov and co‐workers.^[^ ^116^, ^131^ ^]^ Notably, the D‐A type polymer donor PDTID‐DTB (P2) was also exhibited an excellent *V_oc_* (1.02 V) in PDTID‐DTB: PThIND‐Cl‐based OSC, meanwhile, compared with P128: Y6‐based OSC, the enhanced *J_sc_* and *FF* were also verifying the potential development of polymer donors with BdT donor unit. Afterward, based on the reported results of P128, Keshtov and co‐workers constructed the donor unit NdT by introducing benzene group to further extend the conjugated plane of BdT, followed by the synthesis of P129 (2022).^[^ ^354^ ^]^ Notably, after blending with the acceptor Y6, P129: Y6‐based OSC exhibited the *V_oc_* of 0.86 V, *J_sc_* of 23.58 mA cm^−2^, *FF* of 71% and *PCE* of 14.33%. Compared with P128: Y6‐based OSC, the significantly optimized photovoltaic performance presented in P129: Y6‐based OSC can be attributed to the formation of more complemental absorption and finer fibrous network morphology of P129: Y6‐based blend film.

As for other BDT derived donor unit, naphtho[2,1‐*b*:3,4‐*b*′]dithiophene (NDT), thienobenzodithiophene (TBD), dithienobenzodithiophene (DTBDT) and (B‐N)‐benzodithiophene (BNT) also exhibited promising application in the field of polymer donors. Based on the DTBDT, PDPPDTBDT (2014), PDBT‐T1 (2015), PDTBDT‐BZ (2019) and PDTBT‐OTT (2020) were designed by Tae et al.,^[^ ^147^ ^]^ Lin et al.,^[^ ^159^ ^]^ Wang et al.^[^ ^178^ ^]^ and Hwang et al.^[^ ^308^ ^]^ in that order. Furthermore, PTBD‐BZ (2019),^[^ ^178^ ^]^ PBNT‐BDD (2021)^[^ ^356^ ^]^ and PNDT‐3T (2022)^[^ ^293^ ^]^ were also prepared based on the donor units TBD, BNT and NDT in turn. Among these donors, PTBD‐BZ: TBDB‐Na‐based (12.25%), PDBT‐T1: IDIC‐based (10.16%) and PBNT‐BDD: Y6‐BO‐based (15.9%) OSCs both achieve a high *PCE*. Therefore, it is undeniable that constructing polymer donors by introducing BDT derivatives as donor units is a reasonable molecular design strategy.

Notably, among these polymer donors, the high *Td_5%_* achieved by P1(2017) (392°C), P128 (415°C) and P129 (423°C) verifying an excellent stability for application. Meanwhile, due to the *PCE* maintained 97% in 3MT‐Th: ITIC‐based OSC and 78% in PTB7Th: ITIC‐based OSC after 1100 h under ambient condition as well as 30% *PCE* decreasement exhibited by PDPPBDT: PC_71_BM‐based OSC after 180 h, which further prove an excellent device stability. In addition, compared with PBDFF‐BT: PC_71_BM‐based (0.96 nm) blend film, the slightly decreased *RMS* endows PBDFT‐BT: PC_71_BM‐based (0.43 nm) blend films with a smoother morphology, the accelerated hole/electron mobility and exciton dissociation endows PBDFT‐BT: PC_71_BM‐based OSC with a higher *FF* of 60.9%. Similarly, the formation of smooth active layer morphology with suitable *RMS* exhibited by PDTBT‐OTT: PC_71_BM‐based (0.64 nm), L2: TTPT‐T‐4F‐based (1.77 nm) and PBNT‐BDD: Y6‐BO‐based (1.88 nm) blend films also endows corresponding OSCs with a high *FF* of 71.1%, 73.1% and 72%, respectively.

## 4 BDT‐BASED SMALL MOLECULAR DONORS

Indeed, as a widely used donor type, BDT‐based polymer donors have achieved a significantly breakthroughs after applying main‐chain and side‐chain engineering. Up to now, the highest *PCE* presented by polymer donors based OSC devices has already exceeded 19%. In addition, BDT‐based SMDs as an integral part of efficient donor materials. Although SMDs still have a poor film‐forming ability due to their low molecular weight as well as relatively low *PCE* in corresponding OSCs compared with polymer donor based OSCs, the more controllable molecular structure of SMDs has simplified the preparation and purification routes. More important, SMD‐based OSCs usually exhibited a more ideal *V_oc_* and hole/electron mobility. In order to further exploring the potential application of SMDs, this chapter will select representative SMDs and elaborate on molecular design strategies such as main‐chain engineering and side‐chain engineering, thus providing reasonable ideas for designing ideal BDT‐based donor material in future.

### 4.1 Main‐chain engineering

Interestingly, SMDs are similar to polymer donors, the main‐chain engineering usually consists of electron‐donating units, π‐bridge units and acceptor units. Among them, the end‐capping unit, as a special group in molecular backbone, can also be subdivided into units with electron‐donating or electron‐withdrawing. Notably, when the unit with electronegative is introduced as end‐capping group, which often acts as electron‐acceptor unit to match with electron‐donor unit to accelerate the mobility of hole and electron. On the contrary, if electron‐donating unit is selected as end‐capping group, the purpose is usually to match with acceptor units, thereby further promoting the conversion of photons to electrons.

#### 4.1.1 OCAR and its derivatives as end‐capping units

**FIGURE 28:**
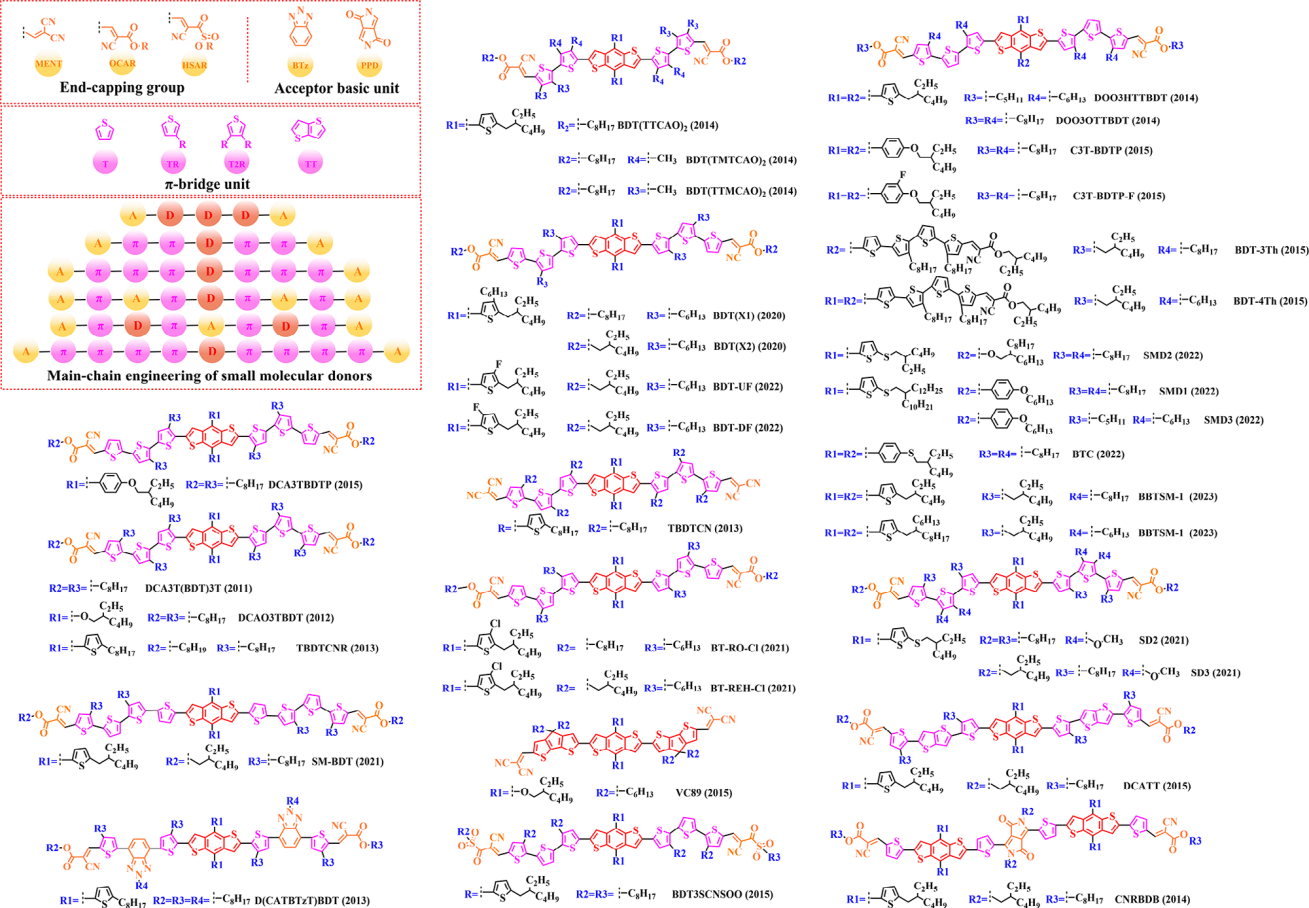
The detailed molecular structure of SMDs synthesized by introducing OCAR and its derivatives as end‐capping basic units.

In recent years, a series of efficient end‐capping units with electronegative, 2‐methylenemalononitrile (MENT), 2‐cyanoarylic acid‐(R) (OCAR) and 2‐(hydrosulfonylcarbonyl)acrylonitrile‐(R) (HSAR) have presented a promising application. Among them, the detailed molecular structure (Figure 28) of SMDs and photoelectric parameters (Table 25) in corresponding OSCs were shown as follows.

**TABLE 25:** Photovoltaic parameters of OSCs related to Figure 28.

Donor	Acceptor	D/A^A^	HOMO [eV] ^B^	$E_{g}^{opt}$ [eV] ^C^	*V_OC_* [V]	*J_SC_* [mA cm^−2^]	*FF* [%]	*PCE* [%] ^D^	Ref.
BDT(TTCAO)_2_	PCBM	1:0.7	−5.30		1.08	2.57	47.3	1.31	[357]
BDT(TMTCAO)_2_	PCBM	1:0.5	−5.25		1.08	6.26	54.0	3.66	[357]
BDT(TTMCAO)_2_	PCBM		−5.23						[357]
DOP3HTTBDT^a^	PC_71_BM	1:1.2	−5.11	1.77	0.87	9.94	65	5.64	[358]
DOO3OTTBDT^b^	PC_71_BM	1:1.2	−5.19	1.76	0.94	8.0	70	5.26	[358]
C3T‐BDTP^c^, ^d^	PC_71_BM	1:1	−5.13	1.79	0.908	9.65	60.1	5.16	[359]
C3T‐BDTP‐F^c^, ^d^	PC_71_BM	1:1	−5.18	1.75	0.968	8.80	63.6	5.30	[359]
BDT‐3Th	Y6		−5.04	1.97	0.85	11.7	35.0	3.50	[360]
BDT‐4Th	Y6		−5.14	2.01	0.84	16.4	39.6	5.60	[360]
BT‐RO‐Cl	Y6		−5.41	1.84	0.865	22.50	68.59	13.20	[361]
BT‐REH‐Cl	Y6		−5.41	1.83	0.868	22.93	69.86	13.52	[361]
SMD1	PC_71_BM		−5.23		0.96	6.95	53.76	3.25	[362]
SMD2	PC_71_BM		−5.19		0.95	5.11	64.39	2.86	[362]
SMD3	PC_71_BM		−5.40		0.94	8.00	62.18	4.48	[362]
BTC	Y6	2:1	−5.37		0.795	18.25	49.74	7.20	[363]
DCAO3T(BDT)3T	PC_61_BM	1:0.5	−5.11	1.83	0.93	9.77	59.9	5.44	[364]
DCAO3TBDT	PC_61_BM	1:0.5	−5.04	1.84	0.95	8.00	60.0	4.56	[365]
TBDTCNR	PC_61_BM	1:0.4	−5.40	1.75	0.90	9.08	66	5.42	[366]
TBDTCN	PC_61_BM	1:0.7	−5.45	1.72	0.88	5.14	43	1.93	[366]
DCA3TBDTP^e^	PC_61_BM	2:1	−5.25	1.82	0.90	7.88	63.66	4.43	[367]
SD2	Y6‐T		−5.11		0.89	17.79	52.3	8.00	[368]
SD3	Y6‐T		−5.10		0.89	14.47	45.0	5.43	[368]
BDT(X1)	PC_71_BM		−5.29		0.96	10.71	69	6.77	[369]
BDT(X1)	IDIC		−5.29		0.92	13.26	71	8.27	[369]
BDT(X2)	PC_71_BM		−5.32		1.00	12.61	69	8.41	[369]
BDT(X2)	IDIC		−5.32		0.94	12.90	58	6.49	[369]
BDT‐UF	N3		−5.39		0.855	24.8	69.9	14.8	[370]
BDT‐DF	N3		−5.37		0.854	24.3	68.8	14.3	[370]
DCATT^f^, ^g^	PC_61_BM	1:1	−5.10	1.85	0.89	8.83	66.23	5.20	[371]
SM‐BDT	Y8		−5.10	1.84	0.84	20.98	57.95	10.36	[372]
D(CATBTzT)BDT	PC_61_BM	1.5:1	−5.13		0.94	5.86	61.2	3.39	[373]
CNRBDB^h^	PC_71_BM	1:1	−5.17	1.53	0.78	3.44	57	1.50	[374]
VC89^i^	PC_71_BM	1:1	−5.38	1.68	0.92	11.56	62	6.56	[375]
BDT3SCNSOO	PC_71_BM	1.5:1	−5.11	1.85	0.93	6.1	53	2.85	[376]

^A^ Weight ratio.

^B^ The HOMO energy level of polymer donors.

^C^ Estimated from the absorption edge in film ($E_{g}^{opt}=1240/\lambda_{onset}$).

^D^ Average *PCE* of OSCs.

^a^ 70°C annealing for 10 min.

^b^ 0.25% DIO.

^c^ 0.4% DIO.

^d^ PDIN layer.

^e^ 70°C annealing for 10 min.

^f^ 0.25% DIO.

^g^ 80°C annealing for 10 min.

^h^ 0.2% DIO.

^i^ SVA‐SA treatment.

In 2014, park and co‐workers reported BDT(TTCAO)_2_, BDT(TMTCAO)_2_ and BDT(TTMCAO)_2_ based on the main chain engineering OCAR‐T2R‐T2R‐BDT‐T2R‐T2R‐OCAR.^[^ ^357^ ^]^ Notably, after blend with the acceptor PCBM, BDT(TTCAO)_2_: PCBM‐based and BDT(TMTCAO)_2_: PCBM‐based OSCs both obtained a high *V_oc_*, while the mismatched absorption and poor blend morphology of active layer lead to low *J_sc_* and *FF*. Afterward, by further introducing thiophene as π‐bridge unit to extend the conjugated plane and optimize hole/electron mobility, Deng et al. constructed the polymer backbone OCAR‐TR‐T‐TR‐BDT‐TR‐T‐TR‐OCAR and then synthesized two novel SMDs DOO3HTTBDT (2014) and DOO3OTTBDT (2014).^[^ ^358^ ^]^ Compared with BDT(TTCAO)_2_: PCBM‐based and BDT(TMTCAO)_2_: PCBM‐based OSCs, the *J_sc_* and *FF* in DOO3HTTBDT: PC_71_BM‐based and DOO3OTTBDT: PC_71_BM‐based OSCs were significantly enhanced. Subsequently, Qiu and co‐workers also prepared C3T‐BDTP (2015) and C3T‐BDTP‐F (2015) based on the main‐chain OCAR‐TR‐T‐TR‐BDT‐TR‐T‐TR‐OCAR,^[^ ^359^ ^]^ which were designed by further introducing side chains alkylbenzene and fluorinated alkyl‐benzene in turn. Benefiting from the highly π‐π conjugated molecular structure, such SMDs both exhibited a promising *V_oc_* (∼0.90 V) in corresponding OSC devices. Furthermore, BDT‐3Th (2015) and BDT‐4Th (2015) were explored by Xu et al.^[^ ^360^ ^]^; RT‐RO‐C1 (2021) and RT‐REH‐C1 (2021) were designed by Chen et al.^[^ ^361^ ^]^; SMD1 (2022), SMD2 (2022) and SMD3 (2022) were synthesized by Huang and co‐workers,^[^ ^362^ ^]^ respectively. In recent years, BTC (2022) was also successfully prepared.^[^ ^363^ ^]^ Due to SMDs usually have a better crystallinity than polymer donors, thus SMD‐based OSC devices can usually present a more ideal *V_oc_*, especially for SMD1: PC_71_BM‐based (0.96 V), SMD2: PC_71_BM‐based (0.95 V) and SMD3: PC_71_BM‐based (0.94 V) OSCs. Furthermore, compared with BDT(X1): IDIC‐based OSC, the substitution of chlorinated alkyl‐thiophene further enhanced the electronegative of OCAR, thereby endows BT‐RO‐Cl: Y6‐based (22.50 mA cm^−2^) and BT‐REH‐Cl: Y6‐based (22.93 mA cm^−2^) OSCs with a higher *J_sc_*. Moreover, it is also worth noting that the branched alkyl substituted thiophene π‐bridge unit effectively increases the miscibility of BT‐REH‐Cl, which can be verified by the higher *FF* (69.86%) exhibited in BT‐REH‐Cl: Y6‐based OSC. Subsequently, after adjusting the substitution of alkyl chain in molecular backbone, DCAO3T(BDT)_3_T (2011),^[^ ^364^ ^]^ DCAO3TBDT (2012)^[^ ^365^ ^]^ and TBDTCNR (2015)^[^ ^366^ ^]^ were reported based on the main chain OCAR‐TR‐TR‐TR‐BDT‐TR‐TR‐TR‐OCAR. Similarly, DCA3TBDTP (2015) was prepared based on the molecular skeleton OCAR‐T‐TR‐TR‐BDT‐TR‐TR‐T‐OCAR;^[^ ^367^ ^]^ SD2 (2021) and SD3 (2021) were explored based on the SMD main‐chain engineering OCAR‐TR‐T2R‐TR‐BDT‐TR‐T2R‐TR‐OCAR^[^ ^368^ ^]^; BDT(X1) (2020),^[^ ^369^ ^]^ BDT(X2) (2020),^[^ ^369^ ^]^ BDT‐UF (2022)^[^ ^370^ ^]^ and BDT‐DF (2022)^[^ ^370^ ^]^ were also successfully constructed based on the SMD backbone OCAR‐T‐TR‐TR‐BDT‐TR‐TR‐T‐OCAR. After applying these SMDs for OSC devices, BDT‐UF: N3‐based (14.8%) and BDT‐DF: N3‐based (14.3%) OSCs both exhibited an efficient *PCE* is OSC devices. Among them, the higher *PCE* presented in BDT‐UF based OSC can be attributed to the more ideal conjugated structure formed by introducing alkyl‐thiophene side chain with ortho‐substituted “F” atom in end‐capping group. In addition, based on the main‐chain engineering OCAR‐TR‐T‐TR‐BDT‐TR‐T‐TR‐OCAR, DCATT (2015) was designed by innovatively introducing TR‐TT‐TR as π‐bridge unit^[^ ^371^ ^]^; SM‐BDT (2021) was constructed by further extending the π–π conjugated plane.^[^ ^372^ ^]^ Moreover, D(CATBTzT)BDT (2013) and CNRBDB (2014) were also synthesized by Chen et al.^[^ ^373^ ^]^ and Zhang et al.^[^ ^374^ ^]^ in turn, which were designed by adjusting the ratio of donor and acceptor units in the main‐chain engineering. Among these SMDs, the highly π‐π conjugated molecular structure with efficient acceptor units endows such SMDs: acceptor‐based OSCs with a promising *V_oc_*. However, how to collaboratively optimize the miscibility of SMDs and blend film morphology in the active layer while maintaining the highly conjugated molecular structure is a critical factor to achieving further breakthrough in OSC devices.

In addition, based on such OCAR derived end‐capping units (e.g. MENT and HSAR), TBTDCN (2013) and VC89 (2015) were synthesized based on the end‐capping group MENT^[^ ^366^, ^375^ ^]^; BDT3SCNSOO (2015) was prepared based on the end‐capping group HSAR.^[^ ^376^ ^]^ Although such SMDs have not achieved excellent photovoltaic performance in photovoltaic devices, constructing derivatives of efficient end‐capping units still has practical guiding significance for designing novel SMDs in future.

Notably, based on the characterization of blend film morphology, compared with BDT(TTCAO)_2_: PCBM‐based blend film (5.68 nm), the smooth active layer with suitable *RMS* of 1.22 nm endows BDT(TMTCAO)_2_: PCBM‐based blend film with a higher *FF* (54.0%). Similarly, among RT‐RO‐Cl: Y6‐based (2.4 nm) and RT‐REH‐Cl: Y6‐based (2.7 nm) blend films; SMD1: PC_71_NM‐based (6.45 nm), SMD2: PC_71_BM‐based (6.53 nm) and SMD3: PC_71_BM‐based (1.43 nm) blend films; DCAO3T(BDT)3T: PC_61_BM‐based (2.16 nm) and DCAO3TBDT: PC_61_BM‐based (4.38 nm) blend films; TBDTCNR: PC_61_BM‐based (1.54 nm) and TBDTCN: PC_61_BM‐based (0.85 nm) blend films; BDT(X1): PC_71_BM‐based (1.86 nm) and BDT(X2): PC_71_BM‐based (1.63 nm) blend films; BDT‐UF: N3‐based (1.90 nm) and BDT‐DF: N3‐based (1.56 nm) blend films; DCATT: PC_61_BM‐based (3.9 nm) and BM‐BDT: Y8‐based (1.85 nm) blend films, the higher *FF* achieved by RT‐REH‐Cl: Y6‐based (69.86%), SMD2: PC_71_BM‐based (64.39%), DCAO3TBDT: PC_61_BM‐based (60.0%), TBDTCNR: PC_61_BM‐based (66%), BDT(X1): PC_71_BM‐based (69%), BDT(X2): PC_71_BM‐based (69%), BDT‐UF: N3‐based (69.9%) and DCATT: PC_61_BM‐based (66.23%) OSCs also can be attributed to the formation of more ideal blend film morphology.

#### 4.1.2 MTOR and its derivatives as end‐capping units

**FIGURE 29:**
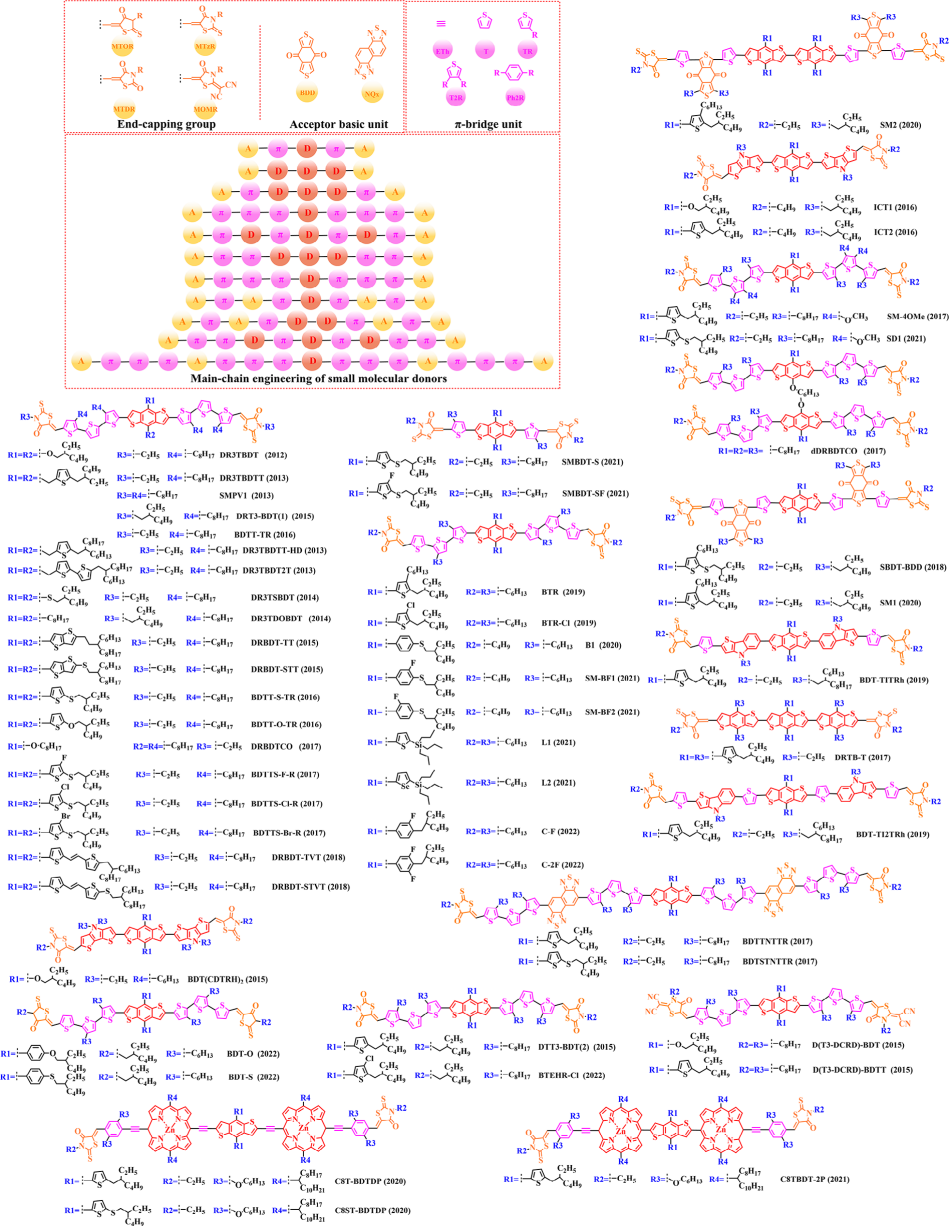
The detailed molecular structure of SMDs synthesized by introducing MTOR and its derivatives as end‐capping basic units.

In recent years, due to such SMDs with MTOR and its derivatives as end‐capping groups exhibited efficient *PCE* in OSC devices (e.g. BTR‐Cl based, B1‐based, SM‐BF1 based and L2 based OSCs), thus utilizing these promising end‐capping groups has attracted the attention of researchers. In order to further explore the potential application of this molecular design strategy, detailed molecular structure (Figure 29) of SMDs and photovoltaic performance (Table 26) in corresponding OSC devices were discussed as follows.

**TABLE 26:** Photovoltaic parameters of OSCs related to Figure 29.

Donor	Acceptor	D/A^A^	HOMO [eV] ^B^	$E_{g}^{opt}$ [eV] ^C^	*V_OC_* [V]	*J_SC_* [mA cm^−2^]	*FF* [%]	*PCE* [%] ^D^	Ref.
DR_3_TBDT^a^	PC_71_BM	1:0.8	−5.02	1.75	0.93	12.21	65.0	7.38	[365]
SD1	Y6‐T		−5.08		0.88	18.23	63.1	9.87	[368]
DR3TBDTT^a^	PC_71_BM	1:0.8	−5.02	1.72	0.93	13.17	66.3	8.12	[377]
DR3TBDTT‐HD^a^	PC_71_BM	1:0.8	−5.06	1.77	0.96	11.92	59.4	6.79	[377]
DR3TBDT2T^a^	PC_71_BM	1:0.8	−5.07	1.78	0.92	12.09	72.1	8.02	[377]
SMPV1^b^	PC_71_BM	1:0.8	−5.51		0.94	12.5	69	8.1	[377]
DR3TSBDT^c^	PC_71_BM	1:0.8	−5.07	1.74	0.96	14.45	73.0	9.60	[378]
DR3TDOBDT^d^	PC_71_BM	1:0.8			0.94	12.56	70	8.26	[379]
DRT3‐BDT^e^	PC_71_BM	1:1			0.90	11.92	63	6.76	[380]
DTT3‐BDT^f^	PC_71_BM	1:1			0.86	10.52	58	5.25	[380]
DRBDT‐TT^g^	PC_71_BM	1:0.8	−5.13	1.78	0.91	12.93	71.0	8.50	[381]
DRBDT‐STT^g^	PC_71_BM	1:0.8	−5.15	1.80	0.90	12.20	70.0	7.85	[381]
BDTT‐TR	PC_70_BM	1:0.8	−5.17	1.73	0.93	11.75	68.1	7.21	[382]
BDTT‐S‐TR	PC_70_BM	1:0.8	−5.18	1.73	0.97	13.45	70.5	9.01	[382]
BDTT‐O‐TR	PC_70_BM	1:0.8	−5.14	1.73	0.90	11.03	65.5	6.26	[383]
DRBDTCO	PC_71_BM	1:1	−4.94	1.73	0.86	12.31	74	7.83	[384]
d‐DRBDTCO^h^	PC_71_BM	1:1	−5.02	1.80	0.88	10.62	72	6.73	[384]
BDTTS‐F‐R	PC_71_BM		−5.28	1.76	0.95	14.31	68.9	9.15	[385]
BETTS‐Cl‐R	PC_71_BM		−5.35	1.77	0.96	14.92	75.3	10.51	[385]
BDTTS‐Br‐R	PC_71_BM		−5.40	1.78	0.98	13.85	63.1	8.24	[385]
DRBDT‐TVT^e^	PC_71_BM		−5.11	1.70	0.879	10.73	72.76	6.67	[386]
DRBDT‐STVT^e^	PC_71_BM		−5.14	1.71	0.907	10.25	73.61	6.61	[386]
BTR^i^	PC_71_BM				0.99	13.40	74	8.9	[387]
BTR	Y6		−5.34	1.78	0.85	22.25	56.4	10.67	[388]
BTR‐Cl	Y6		−5.34	1.78	0.86	24.17	65.5	13.29	[388]
B1^j^	BO‐4Cl		−5.37		0.83	25.27	73	15.3	[389]
SM‐BF1	Y6	2:1	−5.49		0.846	26.64	69.7	15.48	[390]
SM‐BF2	Y6	2:1	−5.45		0.802	20.21	63.1	10.05	[390]
C‐F	N3		−5.29	1.88	0.79	20.51	47.52	7.76	[391]
C‐2F	N3		−5.36	1.90	0.85	24.87	69.33	14.64	[391]
L1	Y6	1.5:1	−5.32	1.76	0.83	25.18	68.3	14.2	[392]
L2	Y6	1.5:1	−5.33	1.77	0.82	26.24	70.4	15.4	[392]
SM‐4OMe	PC_71_BM	1:1.5	−5.25		0.80	2.20	45.21	0.81	[393]
SMBDT‐S^k^	PC_71_BM	1:0.5	−5.56	1.85	1.09	4.86	49	2.62	[394]
SMBDT‐SF^k^	PC_71_BM	1:0.5	−5.72	1.86	1.16	1.44	44	0.74	[394]
BDT(CDTRH)2^e^	PC_71_BM	1:1			0.94	10.42	62	6.07	[395]
ICT1^d^	PC_71_BM		−5.39	1.68	0.87	10.15	58	5.12	[396]
ICT2^d^	PC_71_BM		−5.48	1.63	0.92	10.68	60	5.90	[396]
BDT‐TITRh	PC_71_BM	1:2	−5.24	1.78	0.80	13.05	33.8	3.52	[397]
BDT‐TI2TRh	PC_71_BM	1:2	−5.17	1.75	0.85	13.23	37.3	4.19	[397]
DRTB‐T	IC‐C6IDT‐IC		−5.51	2.0	0.98	14.25	65	9.08	[398]
C8T‐BDTDP	6TIC	1:0.6	−5.24	1.59	0.79	19.36	65.6	10.03	[399]
C8ST‐BDTDP	6TIC	1:0.6	−5.28	1.58	0.75	17.27	64.0	8.29	[399]
C8TBDT‐2P^l^	IDIC	1:1.2	−5.19		0.80	6.83	45.8	2.58	[400]
BDTTNTTR	PC_71_BM		−5.29		0.89	15.70	71.7	9.84	[401]
BDTSTNTTR	PC_71_BM		−5.35		0.93	16.21	76.5	11.22	[401]
SBDT‐BDD^m^	IDIC	1:1	−5.25		0.97	15.13	61.1	8.9	[402]
SM1^n^	ITIC‐4F	1:1	−5.37	1.79	0.83	2.28	21	0.41	[403]
SM2^o^	ITIC‐4F	1:1	−5.50	1.64	0.71	4.77	24	0.82	[403]
BTEHR‐CT^p^	F‐2Cl	1:1	−5.56		0.939	16.66	69.1	10.81	[404]
D(T3‐DCRD)‐BDT	PC_61_BM	1:1	−5.39	1.62	0.93	2.44	49	1.10	[405]
D(T3‐DCRD)‐BDTT	PC_61_BM	1:1	−5.46	1.61	0.96	3.69	55	1.94	[405]
BDT‐O^q^	Y6‐BO		−5.22	1.77	0.817	23.07	64.02	11.84	[406]
BDT‐S^r^	Y6‐BO		−5.31	1.78	0.832	25.14	68.30	14.03	[406]

^A^ Weight ratio.

^B^ The HOMO energy level of polymer donors.

^C^ Estimated from the absorption edge in film ($E_{g}^{opt}=1240/\lambda_{onset}$).

^D^ Average *PCE* of OSCs.

^a^ 0.2 mg ml^−1^ PDMS.

^b^ 0.5 mg ml^−1^ PDMS.

^c^ thermal annealing and SVA treatments.

^d^ SVA treatment.

^e^ CS_2_ SVA treatment for 30s.

^f^ 150°C annealing for 10 min.

^g^ SVA treatment.

^h^ CS_2_ SVA treatment for 90s.

^i^ THF SVA treatment.

^j^ CB SVA treatment.

^k^ SVA treatment for 60s.

^l^ 0.5% DIO.

^m^ CS_2_ SVA treatment for 45s.

^n^ 150°C annealing for 10 min.

^o^ CF/CN cast solvent.

^p^ Thermal annealing treatment.

^q^ 0.25% DIO.

^r^ 100°C annealing for 10 min and 0.25% DIO.

In 2012, a series of novel SMDs DR3TBDT (2012), DR3TBDTT (2012), DR3TBDTT‐HD (2013) and DR3TBDT2T (2013) were first reported by Zhou and co‐workers,^[^ ^366^, ^377^ ^]^ which were designed based on the main chain MTzR‐TR‐T‐TR‐BDT‐TR‐T‐TR. After blending with the acceptor PC_71_BM, the wide conjugated plane in such SMDs endows corresponding OSC devices with a high *V_oc_*. It is also worth noting that the substitution of 2‐hexyloctyl further increases the miscibility of DR3TBDTT, thus the *J_sc_* (13.17 mA cm^−2^) and *FF* (66.3%) both optimized in DR3TBDTT: PC_71_BM‐based OSC. Subsequently, based on the SMD backbone MTzR‐TR‐T‐TR‐BDT‐TR‐T‐TR, SMPV1 (2013),^[^ ^377^ ^]^ DR3TSBDT (2014),^[^ ^378^ ^]^ DR3TDOBDT (2014)^[^ ^379^ ^]^ and DR3‐BDT(1) (2015)^[^ ^380^ ^]^ were also explored by different research teams in subsequence. Benefiting the substitution of alkylthio enhances the electron‐donating of BDT unit as well as solubilized alkyl chain adjusts the miscibility of DR3TSBDT, thereby DR3TSBDT: PC_71_BM‐based OSC obtained a higher *J_sc_* (14.45 mA cm^−2^) and *FF* (73%) than other types of OSC devices. Afterward, considering the molecular design strategy of above SMDs, Kan and co‐workers reported DRBDT‐TT (2015) and DRBDT‐STT (2015) by innovatively introducing thio‐thiophene based side chain into the BDT unit as well as linear alkyl chains into the π‐bridge and end‐capping units in turn.^[^ ^381^ ^]^ Furthermore, BDTT‐TR (2016) and BDTT‐S‐TR (2016) were synthesized by Cui et al.^[^ ^382^ ^]^; BDTT‐O‐TR (2016) and DRBDTCO (2017) were prepared by Min et al.^[^ ^383^ ^]^ and Guo et al.^[^ ^384^ ^]^ in subsequence; BDTTS‐F‐R (2017), BDTTS‐Cl‐R (2017) and BDTTS‐Br‐R (2017) were explored by Ji et al.^[^ ^385^ ^]^; DRBDT‐TVT (2018) and DRBDT‐STVT (2018) were also designed by Huo et al.^[^ ^386^ ^]^ Notably, the introduction of alkylthio effectively enhances the electron‐donating of BDT unit, which effectively promotes the hole/electron mobility in the active layer. Therefore, BDTT‐STR: PC_70_BM‐based OSC exhibited an optimized *J_sc_* and *FF*. Meanwhile, the more ideal crystallinity and closer molecular stacking also ensure the *V_oc_* (0.97 V) to stay at a high level. As for BDTTS‐F‐R (2017), BDTTS‐Cl‐R (2017) and PDTTS‐Br‐R (2017), with the incensement of electronegativity of substitutions, the HOMO energy level gradually decreases.^[^ ^385^ ^]^ Therefore, the stable improvement of *V_oc_* presented in corresponding OSCs can be attributed to the certain positive correlation with *V_oc_* in OSC devices maintained by the difference between the HOMO energy level of SMDs and LUMO energy level of acceptors. However, the excessive atomic radius of “Br” atoms greatly affects the conjugated plane structure of SMDs, which explains the reason why *J_sc_* and *FF* in BDTTS‐Br‐R: PC_71_BM‐based OSC both lower than BDTTS‐F‐R: PC_71_BM‐based and PDTTS‐Cl‐R: PC_71_BM‐based OSCs. Subsequently, by adjusting the substitution of alkyl chain in π‐bridge unit, BTR (2019) and BTR‐Cl (2019) were first reported by Sun et al.^[^ ^387^ ^]^ and Chen et al.^[^ ^388^ ^]^, designing based on the SMD main chain MTzR‐T‐TR‐TR‐BDT‐TR‐TR‐T‐MTzR. Compared with BTR: Y6‐based OSC, due to the more complemental absorption exhibited by BTR‐Cl: Y6‐based blend film, which endows BTR‐Cl: Y6‐based OSC with higher *J_sc_* (24.17 mA cm^−2^) and *FF* (65.5%). Furthermore, by introducing benzene‐based side chain into the BDT unit, B1 (2020),^[^ ^389^ ^]^ SM‐BF1 (2021),^[^ ^390^ ^]^ SM‐BF2 (2021),^[^ ^390^ ^]^ C‐F (2022)^[^ ^391^ ^]^ and C‐2F (2022)^[^ ^391^ ^]^ were also successfully designed by different research teams. After applying for OSC devices, B1: BO‐4Cl‐based and SM‐BF1: Y6‐based OSCs obtained the *V_oc_* of 0.83 and 0.846 V, *J_sc_* of 25.27 and 26.64 mA cm^−2^, *FF* of 73% and 69.7%, and *PCE* of 15.3% and 15.48%, respectively. Moreover, compared with SM‐BF‐based OSC, the *PCE* in SM‐B2F: Y6‐based OSC was significantly decreased. Interestingly, these reported results were also presented between BDT‐UF‐based and BDT‐DF‐based OSCs, which reminds us that steric hindrance is also a critical factor when designing efficient SMDs. In addition, with the incensement of fluorination degree, C‐2F: N3‐based OSC exhibited a better photoelectric performance than C‐F: N3‐based OSC, which confirms that utilizing appropriate molecular polarization is conducive to optimize OSC devices. As for L1 (2021) and L2 (2021),^[^ ^392^ ^]^ which were designed by innovatively introducing side chains (5‐methylthiophen‐2‐yl) tripropylsilane and (5‐methylselenophen‐2‐yl) tripropylsilane into the BDT unit. Similar to the solubilized alkyl chain, tripropylsilane group also effectively improves the miscibility of SMDs, thereby L1: Y6‐based (73%) and L2: Y6‐based (69.7%) OSCs both achieved a high *FF*. Moreover, the introduction of selenophene unit also further enhances the intermolecular interaction of L2, which endows L2: Y6‐based OSC with a better photoelectric performance. In addition, the dimer SMD dDRBDTCO (2017) with the connector octamethylene was first explored by Guo and co‐workers^[^ ^384^ ^]^; SM‐4OMe (2017)^[^ ^393^ ^]^ and SD1 (2021)^[^ ^368^ ^]^ were prepared by further introducing the side chain alkyloxy into the π‐bridge unit; SMBDT‐S (2021) and SMBDT‐SF (2021) were also designed based on the SMD backbone MTzR‐TR‐BDT‐TR‐MTzR.^[^ ^394^ ^]^ Among these SMDs, the highly conjugated molecular structure makes such OSCs to maintain a promising *V_oc_*, especially for SMBDT‐S: PC_71_BM‐based (1.09 V) and SMBDT‐SF: PC_71_BM‐based (1.16 V) OSCs.

Afterward, through adjusting the ratio of donor, acceptor and π‐bridge units, BDT(CDTRH)2 (2015) was first reported based on the A‐D‐D‐D‐A type SMD skeleton MTzR‐DTP‐BDT‐DTP‐MTzR^[^ ^395^ ^]^; Busireddy et al. also synthesized ICT1 (2016) and ICT2 (2016).^[^ ^396^ ^]^ Although the *V_oc_* in SMDs: PC_71_BM‐based OSCs both stay at a high level, while *J_sc_* and *FF* are low. Focusing on this question, BDT‐TITRh (2019) and BDT‐TI2TRh (2019) were explored by further introducing thiophene as π‐bridge unit.^[^ ^397^ ^]^ However, although the extended π‐bridge unit effectively optimized the *J_sc_* in BDT‐TITRh: PC_71_BM‐based and BDT‐TI2TRh: PC_71_BM‐based OSCs, the *V_oc_* and *FF* were significantly decreased. Furthermore, based on the molecular design strategy of above SMDs, DRTB‐T (2017),^[^ ^398^ ^]^ C8T‐BDTDP (2020),^[^ ^399^ ^]^ C8ST‐BDTDP (2020)^[^ ^399^ ^]^ and C8TBDT‐2P (2021)^[^ ^400^ ^]^ were also successfully prepared. Compared with C8TBDT‐2P, the further extended π‐bridge unit endows C8T‐BDTDP: 6TIC‐based and C8ST‐BDTDP: 6TIC‐based OSCs with enhanced *J_sc_* and *FF*, which is similar to the above‐conclusions. As for BDTTNTTR (2017),^[^ ^401^ ^]^ BDTSTNTTR (2017),^[^ ^401^ ^]^ SBDT‐BDD (2018),^[^ ^402^ ^]^ SM1 (2020)^[^ ^403^ ^]^ and SM2 (2020),^[^ ^403^ ^]^ which were designed by further introducing acceptor units NQx and BDD into the SMD backbone. Notably, with the introduction of alkylthio into the BDT unit, the photovoltaic performance of SBDT‐BDD: IDIC‐based OSC was effectively optimized. As for BDTTNTTR and BDTSTNTTR, which were designed based on the long SMD main‐chain engineering MTzR‐TR‐T‐TR‐NPTz‐TR‐T‐TR‐BDT‐TR‐T‐TR‐NPTz‐TR‐T‐TR‐MTzR, both presented a high *PCE* after applying for OSC devices. Similarly, the substitution of alkylthio also endows a better photoelectric performance for BDTSTNTTR: PC_71_BM‐based OSC than BDTTNTTR: PC_71_BM‐based OSC.

In addition, considering the success obtained by such SMDs with end‐capping unit MTzR, a series of novel derived units (e.g. 2‐methylene‐5‐thioxodihydrothiophen‐3(2*H*)‐one‐(R) (MTOR), 5‐methylene‐3λ^2^‐thiazolidine‐2,4‐dione‐(R) (MTDR) and 2‐(5‐methylene‐4‐oxo‐3λ^2^‐thiazolidin‐2‐ylidiene)malononitrile (MOMR)) were also successfully constructed. In 2015, DTT3‐BDT (2) (2015) and BTEHR‐Cl (2022) were reported by Kumar et al.^[^ ^380^ ^]^ and Liu et al.^[^ ^404^ ^]^ in turn, synthesizing based on the end‐capping group MTDR. Furthermore, D(T3‐DCRD)‐BDT (2015) and D(T3‐DCRD)‐BDTT (2015) were explored by introducing MOMR as end‐capping unit^[^ ^405^ ^]^; BDT‐O (2022) and BDT‐S (2022) were also designed by introducing the end‐capping unit MTOR.^[^ ^406^ ^]^ After blending with acceptors, SMD: acceptor‐based OSCs have exhibited a high *V_oc_*, meanwhile, the significantly enhanced *J_sc_* achieved by BDT‐O: Y6‐BO‐based (23.07 mA cm^−2^) and BDT‐S: Y6‐BO‐based (25.14 mA cm^−2^) OSCs can be attributed the more complemental absorption presented in corresponding blend films. Moreover, compared with BDT‐O, BDT‐S with the substitution of alkylthio endows BDT‐S: Y6‐BO‐based OSC with a higher *J_sc_* and *FF*, which further verifying the potential application of utilizing MTzR‐derived units as end‐capping units in the SMD main‐chain engineering.

Notably, based on the characterization of device stability and blend film morphology, the high *Td_5%_* obtained by DRBDT‐TT (393°C) and D(T3‐DCRD)‐BDTT (396°C) as well as only *PCE* decreasement of 25%, 30% and 38% in BDT‐S‐TR: PC_70_BM‐based, BDTT‐TR: PC_70_BM‐based and BDTT‐TR: PC_70_BM‐based OSCs for 580 h in turn exhibiting an excellent device stability, meanwhile, the better *FF* achieved by DRBDT‐TT: PC_71_BM‐based (1.21 nm), DRBDT‐STT: PC_71_BM‐based (1.48 nm), BDTT‐S‐TR: PC_70_BM‐based (1.60 nm), DRBDTCO: PC_71_BM‐based (0.89 nm), d‐DRBDTCO: PC_71_BM‐based (0.46 nm), B1: BO‐4Cl‐based (1.04 nm), SM‐BF1: Y6‐based (1.14 nm), C‐2F: N3‐based (1.26 nm), BTEHR‐CT: F‐2Cl‐based (1.35 nm), D(T3‐DCRD)‐BDTT: PC_61_BM‐based (0.967 nm), BDT‐S: Y6‐BO‐based (1.06 nm) blend films also can be attributed to the formation of suitable blend film morphology.

#### 4.1.3 MTCR, MID and their derivatives as end‐capping units

**FIGURE 30:**
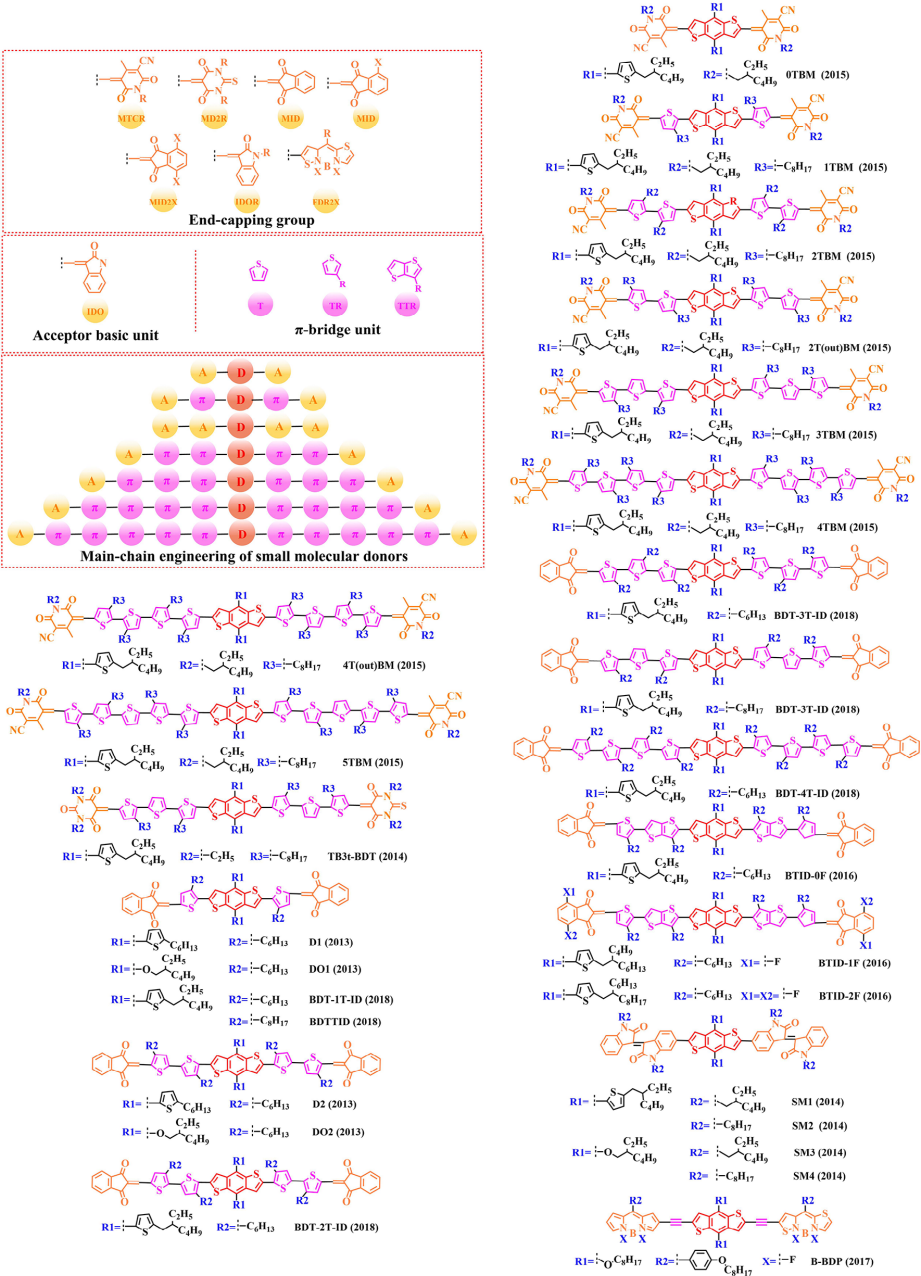
The detailed molecular structure of SMDs synthesized by introducing MTCR, MID and their derivatives as end‐capping basic units.

Indeed, end‐capping groups with electron‐withdrawing have exhibited a remarkable potential application after introducing into the SMD main‐chain engineering. With the deepening of the research, a series of novel end‐capping groups (e.g. 4‐methyl‐5‐methylene‐2,6‐dioxo‐1,2,5,6‐tetrahydropyridine‐3‐carbonitrile‐(R) (MTCR), 2‐methylene‐1H‐indene‐1,3(2*H*)‐dione (MID) and 5*H*,9a*H*‐isothiazolo[2,3‐*c*]thiazolo[2,3‐*f*][1,3,2]diazaborinine‐(2X) (FDR2X) units) have also been continuously designed. In this chapter, the detailed molecular structure (Figure 30) of representative SMDs and photovoltaic parameters (Table 27) in corresponding OSC devices were shown as follows.

**TABLE 27:** Photovoltaic parameters of OSCs related to Figure 30.

Donor	Acceptor	D/A^A^	HOMO [eV] ^B^	$E_{g}^{opt}$ [eV] ^C^	*V_OC_* [V]	*J_SC_* [mA cm^−2^]	*FF* [%]	*PCE* [%] ^D^	Ref.
0TBM^a^	PC_71_BM	1:1	−5.13	1.42	0.64	0.10	31.5	0.02	[407]
1TBM^a^	PC_71_BM	1:1	−5.11	1.46	0.82	3.49	33.2	0.95	[407]
2TBM^a^	PC_71_BM	1:1	−5.10	1.46	0.79	6.66	35.1	1.85	[407]
2T(out)BM	PC_71_BM	1;1	−5.09	1.48	0.81	6.33	30.4	1.56	[407]
3TBM^b^	PC_71_BM	1:1	−5.10	1.42	0.79	14.38	55.4	6.29	[407]
4TBM^a^	PC_71_BM	1:1	−5.06	1.42	0.78	5.01	40.2	1.56	[407]
4T(out)BM	PC_71_BM	1:1	−5.12	1.56	0.76	2.33	25.2	0.45	[407]
5TBM^b^	PC_71_BM	1:1	−5.10	1.42	0.81	9.62	68.7	5.35	[407]
TB3t‐BDT	PC_61_BM	3:2	−5.20	1.65	0.95	9.1	48	4.1	[408]
D1	PC_70_BM	1.5:1	−5.19	1.61	1.03	10.07	54.7	5.67	[409]
DO1	PC_70_BM	1.5:1	−5.18	1.59	0.91	9.47	48.2	4.15	[409]
D2	PC_70_BM	1.5:1	−5.16	1.60	0.92	11.05	66.4	6.75	[409]
DO2	PC_70_BM	1.5:1	−5.16	1.60	0.92	8.58	64.8	5.11	[409]
BDT‐1T‐ID	PC_71_BM	3:1	−5.23		1.06	11.0	49	5.7	[410]
BDT‐2T‐ID	PC_71_BM	3:1	−5.13		0.96	14.0	49	6.7	[410]
BDT‐3T‐ID	PC_71_BM	3:1	−5.03		0.93	13.5	49	6.1	[410]
BDT‐4T‐ID	PC_71_BM	3:1	−4.99		0.88	11.3	51	5.0	[410]
BDTTID^c^	PC_70_BM	7:3	−5.36	1.74	1.03	10.20	53	5.54	[411]
BDT3TID^c^	PC_70_BM	1:1	−5.16	1.67	0.89	9.04	59	4.74	[411]
BTID‐0F	PC_71_BM		−4.91		0.93	14.0	64.0	8.21	[412]
BTID‐1F	PC_71_BM		−4.98		0.94	15.3	72.0	10.37	[412]
BTID‐2F	PC_71_BM		−5.05		0.95	15.7	76.0	11.08	[412]
SM1	PCBM	1:1	−5.50	1.86	0.99	6.82	36.57	2.46	[413]
SM2	PCBM	1:1	−5.48	1.87	0.95	3.67	46.13	1.61	[413]
SM3	PCBM	1:1	−5.38	1.86	0.93	2.62	34.59	0.85	[413]
SM4	PCBM	1:1	−5.37	1.86	0.93	2.23	30.76	0.64	[413]
B‐BDP	PC_71_BM		−5.11	1.46	0.73	11.84	53.8	4.61	[414]

^A^ Weight ratio.

^B^ The HOMO energy level of polymer donors.

^C^ Estimated from the absorption edge in film ($E_{g}^{opt}=1240/\lambda_{onset}$).

^D^ Average *PCE* of OSCs.

^a^ Ca electron extraction layer.

^b^ PDINO layer.

^c^ 1.5% DIO.

In 2015, based on the end‐capping unit MTCR, Tang and co‐workers first reported a series of SMDs 0TBM (2015), 1TBM (2015), 2TBM (2015), 2T(out)BM (2015), 3TBM (2015), 4TBM (2015), 4T(out)BM (2015) and 5TBM (2015), which were synthesized by introducing π‐bridge units with different extended conjugate plane.^[^ ^407^ ^]^ Interestingly, 3TBM presents the most suitable molecular stacking, thus exhibiting the best photoelectric performance among these SMDs after applying for OSC devices. Furthermore, based on the reported results of 3TBM, TB3t‐BDT (2014) was also prepared by introducing the MTCR derivative group MT2R as end‐capping unit.^[^ ^408^ ^]^ Regrettably, the decreased *J_sc_* and FF lead to a low *PCE* in TB3t‐BDT: PC_61_BM‐based OSC. In addition, based on the end‐capping unit MID, D1 (2013) and DO1 (2013) were first reported by Shen and co‐workers,^[^ ^409^ ^]^ which were designed based on the main chain MID‐TR‐BDT‐TR‐MID. Compared with DO1, D1 with the substitution of alkyl‐thiophene effectively broaden the π‐π conjugated plane of SMD backbone, thereby the optimized molecular stacking significantly increases the *V_oc_* (1.03 V) in D1: PC_70_BM‐based OSC. Meanwhile, the solubilized alkyl chain hexyl also enhances the miscibility of D1. Therefore, the finer blending surface morphology endows D1: PC_70_BM‐based OSC with a higher *J_sc_* (10.07 mA cm^−2^) and *FF* (54.7%). Similarly, considering the reported results between D1 and DO1 based OSCs, BDT‐1T‐ID (2018) and BDTTID (2018) were synthesized by Komiyama et al.^[^ ^410^ ^]^ and Lee et al.^[^ ^411^ ^]^ in turn. After introducing side chain 2‐ethylhexyl substituted Thiophene into the BDT unit, the *J_sc_* of BDT‐1T‐ID: PC_71_BM‐based (11.0 mA cm^−2^) and BDTTID: PC_70_BM‐based (10.20 mA cm^−2^) OSCs both achieved a slightly breakthrough. Likewise, by further extending the conjugated plane of π‐bridge unit in this SMD main chain, D2 (2013),^[^ ^409^ ^]^ DO2 (2013)^[^ ^409^ ^]^ and BDT‐2T‐ID (2018)^[^ ^410^ ^]^ were reported based on the SMD skeleton MID‐TR‐TR‐BDT‐TR‐TR‐MID; BDT‐3T‐ID (2018)^[^ ^410^ ^]^ and BDT3TID (2018)^[^ ^411^ ^]^ were prepared based on the SMD backbone MID‐TR‐TR‐TR‐BDT‐TR‐TR‐TR‐MID and MID‐TR‐T‐TR‐BDT‐TR‐T‐TR‐MID, respectively. As for BDT‐4T‐ID (2018),^[^ ^410^ ^]^ which was also synthesized based on the SMD main chain MID‐TR‐TR‐TR‐TR‐BDT‐TR‐TR‐TR‐TR‐MID. By appropriately extending the conjugate plane of π‐bridge unit, although the *V_oc_* is slightly reduced in corresponding OSCs, enhanced *J_sc_* and *FF* endow D2: PC_70_BM‐based (6.75%), BDT‐2T‐ID: PC_71_BM‐based (6.7%) and BDT‐3T‐ID: PC_71_BM‐based (6.1%) OSCs with a higher *PCE*. Moreover, BTID‐0F (2016) was innovatively synthesized based on the main chain MID‐TR‐TTR‐BDT‐TTR‐TR‐MID.^[^ ^412^ ^]^ Compared with D2: PC_70_BM‐based OSC, the higher *J_sc_* (14.0 mA cm^−2^) further increases the *PCE* (8.21%) in BTID: PC_71_BM‐based OSC, which further verifying the reasonability of introducing TT as π‐bridge unit. Afterward, based on the reported results of BTID‐0F, BTID‐1F (2016) and BTID‐2F (2016) were also synthesized based on the end‐capping groups 2‐methylene‐1*H*‐indene‐1,3(2*H*)‐dione‐(X) (MIDX) and 2‐methylene‐1H‐indene‐1,3(2H‐dione‐(2X)) (MID2X) in turn.^[^ ^412^ ^]^ Consistent with the characterizations in polymer donors, with the deepening of fluorination degree, enhanced electron‐withdrawing of end‐capping units effectively optimize the photoelectric performance in BTID‐1F: PC_71_BM‐based and BTID‐2F: PC_71_BM‐based OSCs, with the *V_oc_* of 0.94 and 0.95 V, *J_sc_* of 15.3 and 15.7 mA cm^−2^, *FF* of 72.0% and 76.0%, and *PCE* of 10.37% and 11.08%, respectively. Moreover, compared with DO1: PC_70_BM‐based (1.397 nm) and DO2: PC_70_BM‐based (1.73 nm) blend films, the formation of smoother active layer morphology of D1: PC_70_BM‐based (0.783 nm) and D2: PC_70_BM‐based (0.890 nm) blend films endow corresponding OSCs with a higher *FF*, which deserves further attention.

In addition, SM1 (2014), SM2 (2014), SM3 (2014) and SM4 (2014) were designed by Park and co‐workers,^[^ ^413^ ^]^ synthesizing based on the end‐capping unit 3‐methylene‐1λ^2^‐indolin‐2‐one (IDOR). As for B‐BDP (2017),^[^ ^414^ ^]^ which was constructed based on the end‐capping unit FDR2X. Among them, benefiting the highly conjugated plane of these SMDs, the *V_oc_* in corresponding OSCs both stay at a high level, while *J_sc_* and *FF* were not ideal. Therefore, how to optimize the photovoltaic parameters in SMD‐based OSC devices together still needs to be further explored.

#### 4.1.4 Thiophene and its derivatives as end‐capping units

**FIGURE 31:**
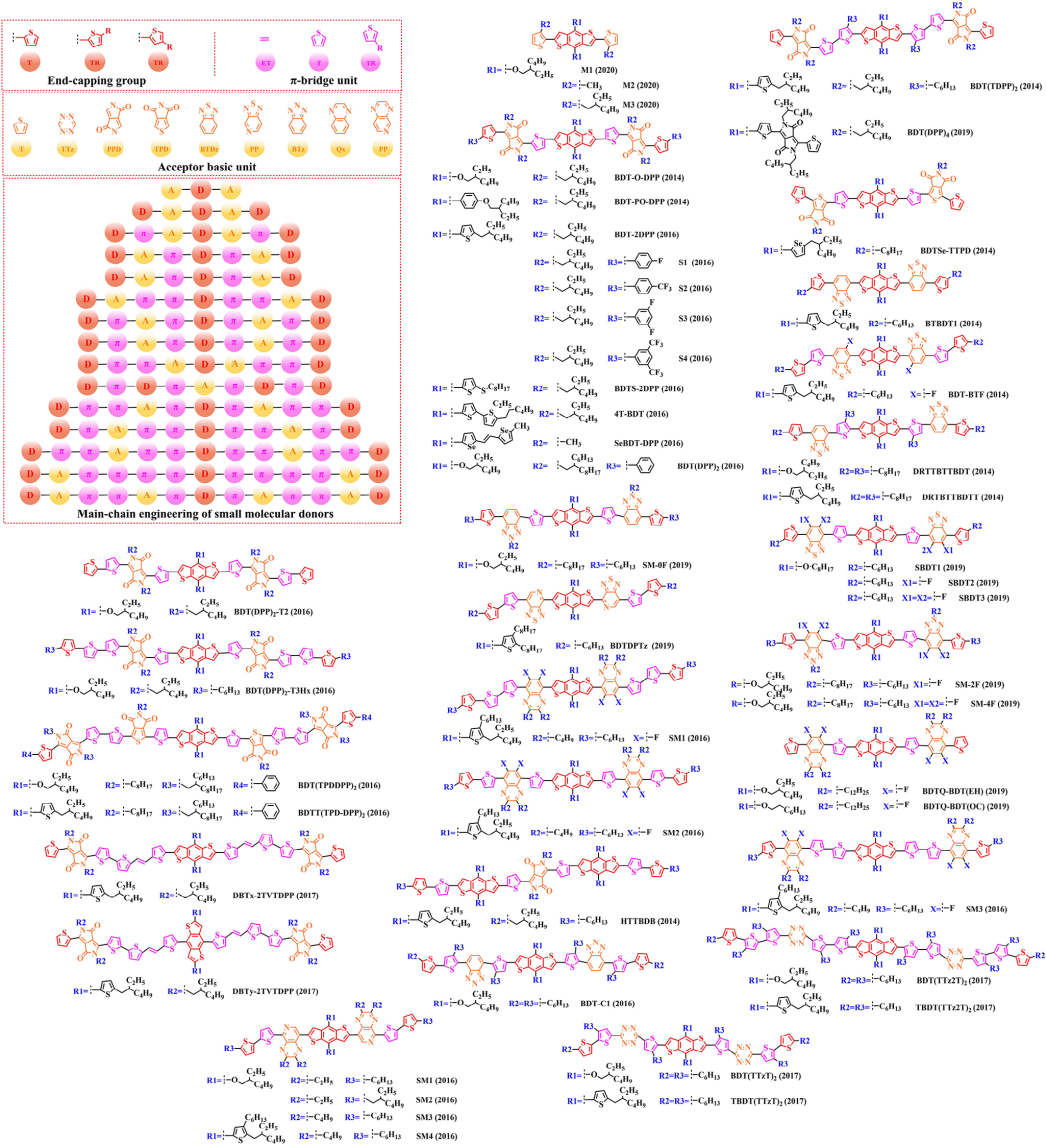
The detailed molecular structure of SMDs synthesized by introducing thiophene and its derivatives as end‐capping basic units.

In order to further explore the reasonability of introducing end‐capping groups with electron‐donating into the SMD main‐chain engineering, the plenty of research have begun to design SMDs with such end‐capping units (e.g. thiophene and alkyl‐thiophene). Among them, the detailed molecular structure (Figure 31) of representative SMDs and photovoltaic parameters (Table 28) in corresponding OSC devices were shown as follows.

**TABLE 28:** Photovoltaic parameters of OSCs related to Figure 31.

Donor	Acceptor	D/A^A^	HOMO [eV] ^B^	$E_{g}^{opt}$ [eV] ^C^	*V_OC_* [V]	*J_SC_* [mA cm^−2^]	*FF* [%]	*PCE* [%] ^D^	Ref.
HTTBDB^a^	PC_71_BM	1:1	−5.13	1.55	0.64	9.66	46	2.81	[374]
BDT‐O‐DPP^b^	PC_61_BM	1:1	−5.16	1.69	0.88	9.54	51.15	4.07	[415]
BDT‐PO‐DPP^c^	PC_61_BM	1:1	−5.25	1.70	0.83	11.23	60.37	5.47	[415]
BDT‐2DPP	IEIC	1:1	−5.23		0.90	8.24	54	3.81	[416]
BDTS‐2DPP^d^	IEIC	1:1	−5.28		0.94	10.87	59	5.69	[416]
4T‐BDT	PC_61_BM		−5.21		0.799	12.17	62.1	5.93	[417]
SeBDT‐DPP^e^	PC_71_BM	1:1			0.79	10.98	58	4.96	[418]
BDT(DPP)_2_	PC_61_BM	1:1	−5.62	1.55	0.81	3.20	49.2	1.26	[419]
BDT(TPDDPP)_2_	PC_61_BM	1:1	−5.68	1.52	0.78	2.83	34.9	0.77	[419]
BDTT(TPD‐DPP)_2_	PC_61_BM	1.5:1	−5.68	1.55	0.77	10.83	50.9	4.25	[419]
S1^f^	PC_71_BM	1:1.5	−5.23		0.76	10.1	52	3.8	[420]
S2^f^	PC_71_BM	1:1.5	−5.33		0.83	6.1	54	2.6	[420]
S3^f^	PC_71_BM	1:1.5	−5.35		0.85	8.3	60	4.0	[420]
S4^f^	PC_71_BM	1:1.5	−5.47		0.94	2.5	45	1.0	[420]
BDT(TDPP)_2_ ^a^	PC_71_BM	1:1	−5.20		0.71	9.85	57	3.90	[421]
BDT(DPP)_4_	C8‐ITIC	1:1	−5.36		0.86	10.1	45	3.6	[422]
BDT(DPP)_2_‐T2	PCBM		−5.19	1.77	0.78	9.72	44.2	3.36	[423]
BDT(DPP)_2_‐T3Hx	PCBM		−5.26	1.70	0.68	4.46	65.6	1.99	[423]
BDTx‐2TVTDPP	PC_61_BM	1:2	−5.1	1.61	0.67	3.61	65.82	1.58	[424]
BDTy‐2TVDPP	PC_71_BM	1:3	−5.3	1.71	0.86	8.60	38.78	2.85	[424]
M1	C60	1:1.5	−5.3	2.73	0.80	2.67	28	0.76	[425]
M2	C60	1:1.5	−5.3	2.72	0.75	4.34	30	1.20	[425]
M3	C60	1:1.5	−5.3	2.76	0.69	2.73	34	0.79	[425]
BDTSe‐TTPD^g^	PC_71_BM	1:2	−5.34	1.86	0.90	10.5	46.3	4.37	[426]
BTBDT1	PC_71_BM	1:2	−5.22	1.67	0.90	6.87	36.2	2.24	[427]
BDT‐BTF	PC_71_BM	3:1			0.85	10.48	66	5.76	[428]
DRTBTTBDT	PC_61_BM	3:1	−5.09	1.81	0.70	4.15	35	1.01	[429]
DRTBTTBDTT	PC_61_BM	1:2	−5.13	1.80	0.89	2.07	29	0.54	[429]
SBDT1	PC_71_BM	1:2	−5.08		0.79	9.97	51.4	3.99	[430]
SBDT2	PC_71_BM	1:2	−5.19		0.85	10.56	56.4	5.06	[430]
SBDT3	PC_71_BM	1:2	−5.33		0.89	9.96	50.3	4.45	[430]
SM1^h^	PC_71_BM	1:1			0.75	6.7	42	2.1	[431]
SM2^i^	PC_71_BM	1:1			0.71	3.6	37	0.9	[431]
SM3^i^	PC_71_BM	1:1			0.81	5.7	49	2.3	[431]
SM4^a^	PC_71_BM	1:1			0.90	10.5	67	6.3	[431]
SM1^a^	PC_71_BM	1:1		1.85	0.88	10.3	70	6.3	[432]
SM2	PC_71_BM	2:3		1.98	0.92	8.8	69	5.6	[432]
SM3	PC_71_BM	3:2		2.01	0.84	4.4	56	2.0	[432]
BDT(PTTPh)_2_			−5.31	1.53					[433]
BDTQ‐BDT(EH)^k^	PC_71_BM	1:2	−5.36		0.83	4.50	32	1.20	[434]
BDTQ‐BDT(OC)^k^	PC_71_BM	1:2	−5.30		0.79	3.52	30	0.83	[434]
BDTDPTz^j^	PC_71_BM	1.5:1	−5.42	1.65	0.868	12.31	56.4	6.28	[434]
BDT‐C1	PC_70_BM	1:1.5	−5.26			10.9	63.4	5.9	[435]
SM‐0F	PC_71_BM	1:1.5	−5.09		0.73	7.3	43.4	2.56	[436]
SM‐2F	PC_71_BM	1:1.5	−5.12		0.75	11	44.9	3.94	[436]
SM‐4F	PC_71_BM	1:1.5	−5.13		0.77	9.1	46.7	3.48	[436]
BDT(TTzT)_2_ ^l^	PC_71_BM		−5.51	1.91	0.98	9.48	53.0	4.91	[437]
TBDT(TTzT)_2_ ^l^	PC_71_BM	1.5:1	−5.59	1.99	1.03	9.50	61.6	6.04	[437]
BDT(TTz2T)_2_ ^l^	PC_71_BM		−5.30	1.77	0.87	10.54	56.8	5.22	[437]
TBDT(TTz2T)_2_ ^l^	PC_71_BM	1:1	−5.37	1.82	0.94	10.65	65.1	6.56	[437]

^A^ Weight ratio.

^B^ The HOMO energy level of polymer donors.

^C^ Estimated from the absorption edge in film ($E_{g}^{opt}=1240/\lambda_{onset}$).

^D^ Average *PCE* of OSCs.

^a^ 0.2% DIO.

^b^ 110°C annealing for 10 min.

^c^ 110°C annealing for 10 min and 3% 1,2‐Dichlorobenzene.

^d^ invert device.

^e^ 2% 1‐CN.

^f^ 0.5% 1‐chloronaphthalene.

^g^ active layer thickness is 95 nm.

^h^ 1% DIO.

^i^ 0.5% DIO.

^j^ 0.25% PN.

^k^ 3% DIO.

^l^ DIO.

In 2014, two novel SMDs BDT‐O‐DPP (2014) and BDT‐PO‐DPP (2014) were first reported by Du and co‐workers,^[^ ^415^ ^]^ synthesizing based on the main chain TR‐PPD‐T‐BDT‐T‐PPD‐TR. After blending with the acceptor PC_61_BM, BDT‐O‐DPP: PC_61_BM‐based and BDT‐PO‐DPP: PC_61_BM‐based OSCs obtained the *V_oc_* of 0.88 and 0.83 V, *J_sc_* of 9.54 and 11.23 mA cm^−2^, *FF* of 51.15% and 60.37%, and *PCE* of 4.07% and 5.47%, respectively. Subsequently, BDT‐2DPP (2016),^[^ ^416^ ^]^ BDTS‐2DPP (2016),^[^ ^416^ ^]^ 4T‐BDT (2016)^[^ ^417^ ^]^ and SeBDT‐DPP (2016)^[^ ^418^ ^]^ were also successfully prepared in turn. Notably, compared with BDT‐PO‐DPP, the substitution of alkylthio substituted thiophene side chain effectively enhanced the *V_oc_* in BDT‐2DPP: IEIC‐based (0.90 V) and BDTS‐2DPP: IEIC‐based (0.94 V) OSCs. Meanwhile, the introduction of alkyl‐thiophene substituted thiophene side chain also endows 4T‐BDT with an optimized *J_sc_* (12.17 mA cm^−2^) and *FF* (62.1%) in 4T‐BDT: PC_61_BM‐based OSC. In addition, BDT(DPP)_2_ (2016),^[^ ^419^ ^]^ S1 (2016),^[^ ^420^ ^]^ S2 (2016),^[^ ^420^ ^]^ S3 (2016)^[^ ^420^ ^]^ and S4 (2016)^[^ ^420^ ^]^ were also explored by innovatively introducing fluorinated benzene substituted thiophene side chain into the BDT unit. Interestingly, with the deepening of fluorination degree, the *V_oc_* in BDT(DPP)2: PC_61_BM‐based, S1: PC_71_BM‐based, S2: PC_71_BM‐based, S3: PC_71_BM‐based and S4: PC_71_BM‐based OSCs exhibited a gradual upward trend, which is consistent with the characterization in polymer donors. Furthermore, the best photoelectric performance presented in S3 based OSC also verifying that only appropriate fluorination degree can produce a positive optimization for OSC devices. Subsequently, a series of novel SMDs were also synthesized by adjusting the π‐bridge unit, BDT‐(TDPP)_2_ (2014)^[^ ^421^ ^]^ and BDT (DPP)_4_ (2014)^[^ ^422^ ^]^ were synthesized based on the SMD main‐chain engineering T‐TPD‐T‐TR‐BDT‐TR‐T‐TPD‐T; BDT(DPP)_2_‐T2^[^ ^423^ ^]^ and BDT(DPP)_2_‐T3Hx (2016)^[^ ^423^ ^]^ were prepared based on the SMD backbone T‐T‐TPD‐T‐BDT‐T‐TPD‐T‐T and TR‐T‐T‐TPD‐T‐BDT‐T‐TPD‐T‐T‐TR in turn; BDT(TPDDPP)_2_ (2016),^[^ ^419^ ^]^ BDTT(TPD‐DPP)_2_ (2016),^[^ ^419^ ^]^ DBTx‐2TVTDPP (2017)^[^ ^424^ ^]^ and DBTy‐2TVTDPP (2017)^[^ ^424^ ^]^ were also successfully designed. Furthermore, Zhang and co‐workers reported HTTBDB (2014) by introducing two BDT groups as donor units into the SMD backbone^[^ ^374^ ^]^; Yagui et al. innovatively constructed a series of novel SMDs M1 (2020), M2 (2020) and M3 (2020) after constructing the simple SMD backbone TR‐BDT‐TR.^[^ ^425^ ^]^ Notably, with the extending of π‐bridge unit in SMD skeleton, the *V_oc_* in corresponding OSCs presented a gradually decreased trend. Moreover, between the characterization results of BDT(TPDDPP)_2_: PC_61_BM‐based and BDTT(TPD‐DPP)_2_: PC_61_BM‐based OSCs, the substitution of 2‐(2‐ethylhexyl)−5‐methylthiophene effectively increased the *J_sc_* (10.83 mA cm^−2^) and *FF* (50.9%) in BDTT(TPD‐DPP)2: PC_61_BM‐based OSC. Meanwhile, the better photoelectric performance exhibited by M2: C60‐based OSC than M1: C60‐based and M3: C60N‐based OSCs further proved that the positive optimization for OSC devices can be achieved by introducing appropriate alkyl chain into the end‐capping group of SMD backbone.

In addition, by introducing other acceptor units (e.g. TPD, BTDz, BTz and Qx) with stronger electron‐withdrawing into the SMD main‐chain engineering. BDTSe‐TTPD (2014) was first reported by Kim et al.^[^ ^426^ ^]^, synthesizing by introducing TPD as acceptor unit. after blending with the acceptor PC_71_BM, BDTSe‐TTPD: PC_71_BM‐based OSC obtained the *V_oc_* of 0.90 V, *J_sc_* of 10.5 mA cm^−2^, *FF* of 46.3% and *PCE* of 4.37%. Similarly, after introducing the efficient acceptor unit BTDz, BTBDT1 (2014),^[^ ^427^ ^]^ BDT‐BTF (2014),^[^ ^428^ ^]^ DRTTBTTBDT (2014),^[^ ^429^ ^]^ DRTBTTBDTT (2014),^[^ ^429^ ^]^ SBDT1 (2019),^[^ ^430^ ^]^ SBDT2 (2019)^[^ ^430^ ^]^ and SBDT3 (2019)^[^ ^430^ ^]^ were also synthesized by different research teams. Among the characterization results of BDT‐BTF: PC_71_BM‐based, DRTBTTBDTT: PC_61_BM‐based and SBDT2: PC_71_BM‐based OSCs, our group noticed that adjusting the order of π‐bridge unit into the SMD skeleton will produce a great impact on OSC devices, the best photoelectric performance presented by BDT‐BTF‐based OSC deserves further attention. Moreover, compared with SBDT1: PC_71_BM‐based and SBDT3: PC_71_BM‐based OSCs, the significantly enhanced *J_sc_* and *FF* presented by SBDT2: PC_71_BM‐based OSC once again verified only under the appropriate fluorination degree can produce a positive optimization for SMD‐based OSC devices. Afterward, based on the reported results of BDT‐BTF, a series of novel SMDs SM1 (2016), SM2 (2016), SM3 (2016) and SM4 (2016) were synthesized by wolf et al.^[^ ^431^ ^]^; SM1 (2016) and BDTDPTz (2019) were prepared by Wang et al.^[^ ^432^ ^]^; BDT(PTTPh)_2_ (2023) was also designed by Liu et al.^[^ ^433^ ^]^ Among these donors, due to the introduction of acceptor units with strong electron‐withdrawing, higher *PCE*s were also achieve by BDTDPTz: PC_71_BM‐based (6.28%), SM4: PC_71_BM‐based (6.3%) and SM1: PC_71_BM‐based (6.3%) OSCs. Furthermore, SM2 (2016),^[^ ^432^ ^]^ SM3 (2016),^[^ ^432^ ^]^ BDTQ‐BDT(EH) (2019)^[^ ^434^ ^]^ and BDTQ‐BDT(OC) (2019)^[^ ^434^ ^]^ were also explored by introducing the acceptor unit Qx; BDT‐Cl (2016),^[^ ^435^ ^]^ SM‐0F (2019),^[^ ^436^ ^]^ SM‐2F (2019)^[^ ^436^ ^]^ and SM‐4F (2019)^[^ ^436^ ^]^ were synthesized based on the acceptor unit BTz; BDT(TTzT)_2_ (2017),^[^ ^437^ ^]^ TBDT(TTzT)_2_ (2017),^[^ ^437^ ^]^ BDT(TTz2T)_2_ (2017)^[^ ^437^ ^]^ and TBDT(TTz2T)_2_ (2017)^[^ ^437^ ^]^ were innovatively introducing TTz as acceptor unit. Benefiting the highly π–π conjugated structure formed in SMD polymer backbone, the *V_oc_* in corresponding OSC devices stay at a high level, especially for BDT(TTzT)_2_: PC_71_BM‐based (0.98 V) and TBDT(TTzT)_2_: PC_71_BM‐based (1.03 V) OSCs.

Among these types of SMDs, the high *Td_5%_* achieved by BDTx‐2TVTDPP (406°C), BDTy‐2TVDPP (429°C), BDTSe‐TTPD (419°C), BDT‐BTF (425°C) and BDTDPTz (433°C) exhibiting an excellent stability. Meanwhile, the better blend film morphology formed by BDTx‐2TVTDPP: PC_61_BM‐based (2.61 nm), BDT‐Cl: PC_70_BM‐based (6.5 nm), TBDT(TTzT)_2_: PC_71_BM‐based (4.7 nm) and TBDT(TTz2T)_2_: PC71BM‐based (3.1 nm) blend films endow corresponding OSCs with a better photoelectric performance, with the *FF* of 65.82%, 63.4%, 61.6% and 65.1%, respectively.

#### 4.1.5 BDT2R, BTIR, TPA and their derivatives as end‐capping units

**FIGURE 32:**
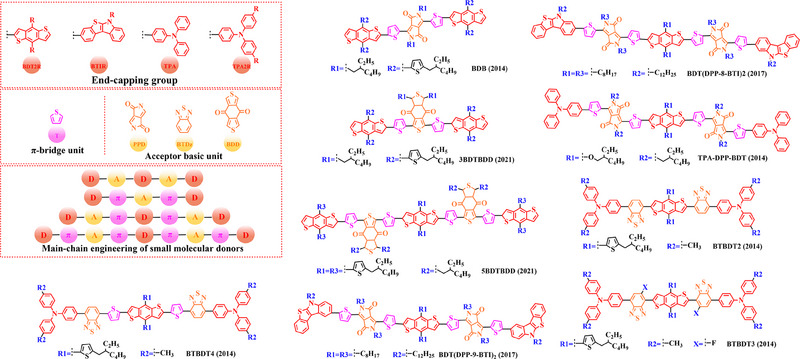
The detailed molecular structure of SMDs synthesized by introducing BDT2R, BTIR, TPA and their derivatives as end‐capping basic units.

Indeed, selecting thiophene and its derivatives as end‐capping groups in SMD main‐chain engineering has exhibited a promising application. However, compared with SMDs synthesized based on the end‐capping groups with electronegative, the *PCE* in such OSCs prepared based on SMDs with electron‐donating end‐capping group is still low. In order to break this dilemma, some research teams begun to introduce end‐capping groups with stronger electron‐donating into the SMD backbone. Among them, the detailed molecular structure (Figure 32) of representative SMDs and photovoltaic parameters (Table 29) in corresponding OSC devices were discussed as follows.

**TABLE 29:** Photovoltaic parameters of OSCs related to Figure 32.

Donor	Acceptor	D/A^A^	HOMO [eV] ^B^	$E_{g}^{opt}$ [eV] ^C^	*V_OC_* [V]	*J_SC_* [mA cm^−2^]	*FF* [%]	*PCE* [%] ^D^	Ref.
BDB	PC_71_BM	1:1	−5.16	1.58	0.78	4.22	27	0.86	[374]
BTBDT2	PC_71_BM	1:2	−5.25	1.72	0.93	8.19	46.1	3.51	[427]
BTBDT3	PC_71_BM	1:2	−5.23	1.69	0.90	10.08	43.1	3.91	[427]
BTBDT4	PC_71_BM	1:2	−5.11	1.64	0.82	8.89	42.6	3.10	[427]
3BDTBDD^a^	ITIC	1:0.8	−5.50		0.90	9.51	50.6	4.02	[438]
5BDTBDD^a^	ITIC	1:0.8	−5.46		0.91	13.23	65.6	7.66	[438]
TPA‐DPP‐BDT	PC_71_BM	1:1	−5.12	1.52	0.76	9.71	40.3	2.97	[439]
BDT(DPP‐9‐BTI)2	PC_71_BM	1:4	−5.07	1.46	0.71	7.14	33.78	1.72	[440]
BDT(DPP‐8‐BTI)2	PC_71_BM	1:1	−5.20	1.43	0.62	7.23	49.3	2.00	[440]

^A^ Weight ratio.

^B^ The HOMO energy level of polymer donors.

^C^ Estimated from the absorption edge in film ($E_{g}^{opt}=1240/\lambda_{onset}$).

^D^ Average *PCE* of OSCs.

^a^ 0.2% DIO.

Considering BDT group as an efficient electron donor, exhibiting an excellent applicability in the field of polymer donors and SMDs. Therefore, the novel SMD BDB (2014) was first reported by Zhang and co‐workers.^[^ ^374^ ^]^ After blending with the acceptor PC_71_BM, BDB: PC_71_BM based OSC obtained the *V_oc_* of 0.78 V, *J_sc_* of 4.22 mA cm^−2^, *FF* of 27% and *PCE* of 0.86%, respectively. Subsequently, by introducing efficient acceptor unit BDD, 3BDTBDD (2021) and 5BDTBDD (2021) were also prepared by Xia et al.^[^ ^438^ ^]^ Due to forming a more complemental absorption in 3BDTBDD: ITIC‐based and 5BDTBDD: ITIC‐based blend films, the *J_sc_* of 3BDTBDD: ITIC‐based (9.51 mA cm^−2^) and 5BDTBDD: ITIC‐based (13.23 mA cm^−2^) OSCs both significantly enhanced. Meanwhile, compared with 3BDTBDD: ITIC‐based OSC (4.02%), the higher *PCE* presented by 5BDTBDD: ITIC‐based OSC (7.66%) also proves the superiority of D‐π‐A‐π‐D‐π‐A‐π‐D type SMD skeleton.

In addition, based on the SMD backbone of 5BDTBDD, TPA‐DPP‐BDT (2014) was synthesized by introducing TPA as end‐capping group^[^ ^439^ ^]^; BDT‐(DPP‐8‐BDT) (2017) and BDT(DPP‐9‐BDT) (2017) were prepared based on the end‐capping group BTIR.^[^ ^440^ ^]^ Interestingly, compared with BDT‐(DPP‐9‐BDT): PC_71_BM‐based OSC, BDT‐(DPP‐8‐BDT) with meta‐substituted end‐capping unit presented a higher *J_sc_* (7.23 mA cm^−2^) and *FF* (49.3%) in BDT‐(DPP‐8‐BDT): PC_71_BM‐based OSC. Therefore, it reminds us that adjusting the substitution sites of end‐capping groups in SMD backbone is also a rational molecular design strategy. As for BTBDT2 (2014), BTBDT3 (2014) and BTBDT4 (2014), which were synthesized by innovatively introducing triphenylamine‐(2R) (TPA2R) as end‐capping unit.^[^ ^427^ ^]^ After applying for OSC devices, the more suitable fluorination degree endows higher *PCE* for BTBDT2: PC_71_BM‐based OSC (3.91%) than BTBDT1: PC_71_BM‐based (3.51%) and BTBDT3: PC_71_BM‐based (3.10%) OSCs.

Moreover, based on the characterization of blend films morphology, among BDB: PC_71_BM‐based (3.69 nm), 3BDTBDD: ITIC‐based (0.87 nm), 5BDTBDD: ITIC‐based (1.23 nm) and TPA‐DPP‐BDT: PC_71_BM‐based (0.492 nm) blend films, the formation of active layer with suitable *RMS* endows 5BDTBDD: ITIC‐based OSC with a higher *FF* of 65.5%.

#### 4.1.6 Other molecular design strategies

**FIGURE 33:**
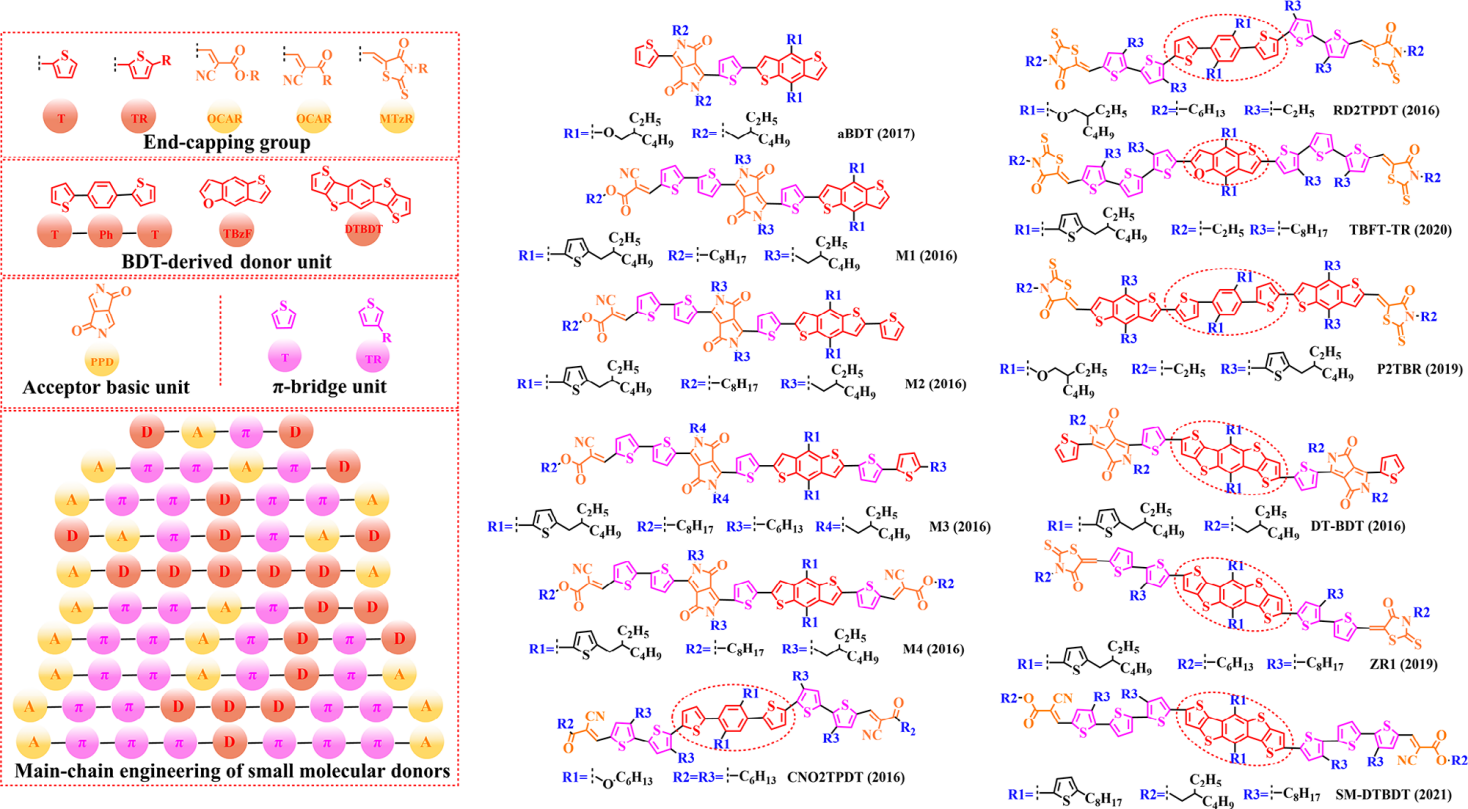
The detailed molecular structure of SMDs synthesized by introducing the derivatives of BDT as donor units.

In recent years, the breakthrough of *PCE* in binary OSC devices prepared based on the SMDs with symmetrical electronegativity or electron‐donating end‐capping units has stagnated. In order to break up this dilemma, by designing asymmetric SMD main‐chain engineering as well as introducing BDT derivatives as donor units both exhibiting a promising application. Among them, the detailed molecular structure (Figure 33) of representative SMDs and photovoltaic performance (Table 30) in corresponding OSC devices were shown as follows.

**TABLE 30:** Photovoltaic parameters of OSCs related to Figure 33.

Donor	Acceptor	D/A^A^	HOMO [eV] ^B^	$E_{g}^{opt}$ [eV] ^C^	*V_OC_* [V]	*J_SC_* [mA cm^−2^]	*FF* [%]	*PCE* [%] ^D^	Ref.
SM‐DTBDT	Y8		−5.05	1.83	0.80	24.55	63.04	12.45	[372]
DT‐BDT	PC_61_BM		−5.13		0.848	9.76	48.0	3.95	[417]
M1^a^	PC_71_BM	1:1	−5.14	1.53	0.82	7.06	61.7	3.48	[441]
M2^b^	PC_71_BM	1:1	−5.19	1.48	0.74	6.65	62.3	3.01	[441]
M3^b^	PC_71_BM	1:1	−5.20	1.46	0.67	6.45	58.4	2.45	[441]
M4^b^	PC_71_BM	1:1	−5.27	1.44	0.78	2.76	42.0	0.83	[441]
aBDT	PCBM	1.5:1	−5.23	1.82	0.81	1.74	27	0.4	[442]
CNO2TPDT	PC_71_BM	1.5:1	−5.03	1.72	0.87	7.09	67.6	4.07	[443]
RD2TPDT^c^	PC_71_BM	1:0.8	−4.98	1.66	0.77	12.74	67.6	6.59	[443]
P2TBR^d^	IDIC	1:1	−5.33	1.93	0.94	16.8	70.1	11.2	[444]
TBFT‐TR	Y6				0.784	24.09	72.78	14.03	[445]
ZR1	Y6		−5.32		0.861	24.34	68.44	14.27	[446]
BBTSM‐1	Y6	1.8:1	−5.56	1.89	0.87	20.7	62.3	11.3	[447]
BBTSM‐2	Y6	1.2:1	−5.50	1.89	0.78	16.0	43.0	5.3	[447]

^A^ Weight ratio.

^B^ The HOMO energy level of polymer donors.

^C^ Estimated from the absorption edge in film ($E_{g}^{opt}=1240/\lambda_{onset}$).

^D^ Average *PCE* of OSCs.

^a^ 0.2% DIO.

^b^ 1% DIO.

^c^ 0.8% DIO.

^d^ CS_2_ SVA treatment for 30 s.

In 2016, M1 (2016) with a single end‐capping unit OCAR was first designed based on the asymmetric SMD main‐chain engineering OCAR‐T‐T‐PPD‐T‐BDT.^[^ ^441^ ^]^ After blending with the acceptor PC_71_BM, M1: PC_71_BM‐based OSC obtained the *V_oc_* of 0.82 V, *J_sc_* of 7.06 mA cm^−2^, *FF* of 61.7% and *PCE* of 3.48%. Subsequently, based on the molecular structure of M1, M2 (2016) and M3 (2016) were prepared by further introducing thiophene as end‐capping unit.^[^ ^441^ ^]^ Moreover, M4 (2016) synthesized based on the asymmetric SMD backbone OCAR‐T‐T‐PPD‐T‐BDT‐T‐OCAR^[^ ^441^ ^]^; aBDT (2017) was also designed based on the asymmetric SMD skeleton T‐TPD‐T‐BDT.^[^ ^442^ ^]^ Although these SMDs do not exhibit ideal photovoltaic performance after applying for OSC devices, constructing asymmetric SMD backbone also provides us with a reasonable molecular design strategy worth exploring.

In addition, by introducing BDT‐derived donor unit to construct novel SMDs. CNO2TPDT (2016),^[^ ^443^ ^]^ RD2TPDT (2016)^[^ ^443^ ^]^ and P2TBR (2019)^[^ ^444^ ^]^ were reported based on the donor unit T‐Ph‐T. After blending with acceptors, CNO2TPDT: PC_71_BM‐based, RD2TPDT: PC_71_BM‐based and P2TBR: IDIC‐based OSCs obtained the *V_oc_* of 0.87, 0.77 and 0.94 V; *J_sc_* of 7.09, 12.74, 16.8 mA cm^−2^; *FF* of 67.6%, 67.6% and 70.1%; and *PCE* of 4.07%, 6.59% and 11.2%, respectively. Among them, the better photoelectric performance exhibited by P2TBR: IDIC‐based OSC can be attributed to forming a more complemental absorption and finer blend film morphology in the active layer. As for other BDT derivatives, TBFT‐TR (2020) was reported based on the donor unit TBzF^[^ ^445^ ^]^; DT‐BDT (2016),^[^ ^417^ ^]^ ZR1 (2019)^[^ ^446^ ^]^ and SM‐DTBDT (2021)^[^ ^372^ ^]^ were prepared by introducing DTBDT as donor unit; BBTSM‐1 (2023) and BBTSM‐2 (2023) were also synthesized based on the BDT derivative unit BzBT.^[^ ^447^ ^]^ Comparing the detailed photovoltaic parameters between BBTSM‐1: Y6‐based and BBTSM‐2: Y6‐based OSCs, π‐bridge unit in BBTSM‐1 with a longer alkyl chain octyl significantly optimized the light absorption, molecular stacking and miscibility in the active layer, which explains the reason why BBTSM‐1: Y6‐based OSC achieved a higher *V_oc_* (0.87 V), *J_sc_* (20.7 mA cm^−2^) and *FF* (62.3%). Furthermore, although the advantage of high *V_oc_* in such SMD‐based OSC devices has been weakened, the significantly enhanced *J_sc_* and *FF* endows TBFT‐TR: Y6‐based (14.03%), ZR1: Y6‐based (14.27%) and SM‐DTBDT: Y8‐based (12.45%) OSCs with a higher *PCE*. Therefore, introducing BDT derivative groups as donor units into the SMD backbone also exhibiting a promising potential application.

Moreover, among these types of SMDs, SM‐DTBDT: Y8‐based (1.44 nm), CNO2TPDT: PC_71_BM‐based (1.51 nm), RD2TPDT: PC_71_BM‐based (0.61 nm) and BBTSM‐1: Y6‐based (2.22 nm) blend films with suitable *RMS* endow corresponding OSCs with a better *FF* of 63.04%, 67.6%, 67.6% and 62.3% in turn, which deserves further attention.

### 4.2 Side‐chain engineering

Without any doubt, main‐chain engineering as an efficient molecular design strategy, has been widely used to endow SMD with an ideal crystallinity and tight molecular accumulation in the active layer, thereby effectively optimizing the *V_oc_* in corresponding OSC devices. However, the low *J_sc_* and *FF* still seriously hinder the further breakthrough of SMD‐based OSC devices. Therefore, it is particularly important to further regulate the molecular structure of SMDs by utilizing side‐chain engineering.

#### 4.2.1 Substitution of fluorine, chlorine and bromine atoms

**FIGURE 34:**
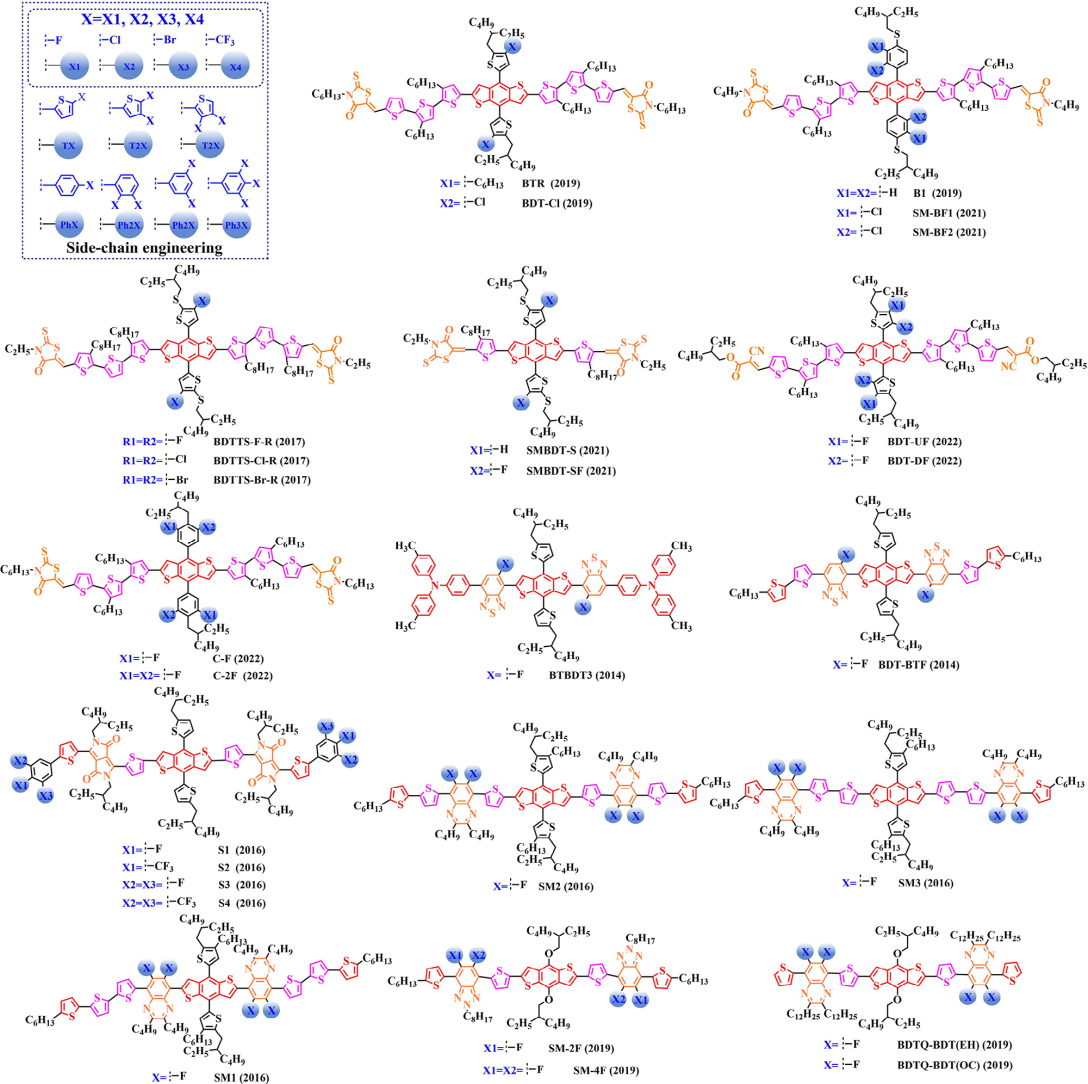
The detailed molecular structure of SMDs synthesized by introducing fluorine, chlorine and bromine atoms.

Similar to the polymer donors, the introduction of fluorine, chlorine and bromine atoms with strong electronegative into the SMDs also effectively decreases the HOMO energy level and accelerates hole/electron mobility in corresponding SMD‐based OSC devices. To explore the potential relationship between SMDs and corresponding OSCs, the detailed molecular structure (Figure 34) of SMDs and photoelectric performance (Table 31) in OSC devices were discussed as follows.

**TABLE 31:** Photovoltaic parameters of OSCs related to Figure 34.

Donor	Acceptor	D/A^A^	HOMO [eV] ^B^	$E_{g}^{opt}$ [eV] ^C^	*V_OC_* [V]	*J_SC_* [mA cm^−2^]	*FF* [%]	*PCE* [%] ^D^	Ref.
BDT‐UF	N3		−5.39		0.855	24.8	69.9	14.8	[370]
BDT‐DF	N3		−5.37		0.854	24.3	68.8	14.3	[370]
BDTTS‐F‐R	PC_71_BM		−5.28	1.76	0.95	14.31	68.9	9.15	[385]
BETTS‐Cl‐R	PC_71_BM		−5.35	1.77	0.96	14.92	75.3	10.51	[385]
BDTTS‐Br‐R	PC_71_BM		−5.40	1.78	0.98	13.85	63.1	8.24	[385]
BTR	Y6		−5.34	1.78	0.85	22.25	56.4	10.67	[388]
BTR‐Cl	Y6		−5.34	1.78	0.86	24.17	65.5	13.29	[388]
B1^a^	BO‐4Cl		−5.37		0.83	25.27	73	15.3	[389]
SM‐BF1	Y6	2:1	−5.49		0.846	26.64	69.7	15.48	[390]
SM‐BF2	Y6	2:1	−5.45		0.802	20.21	63.1	10.05	[390]
C‐F	N3		−5.29	1.88	0.79	20.51	47.52	7.76	[391]
C‐2F	N3		−5.36	1.90	0.85	24.87	69.33	14.64	[391]
SMBDT‐S^b^	PC_71_BM	1:0.5	−5.56	1.85	1.09	4.86	49	2.62	[394]
SMBDT‐SF^b^	PC_71_BM	1:0.5	−5.72	1.86	1.16	1.44	44	0.74	[394]
S1^c^	PC_71_BM	1:1.5	−5.23		0.76	10.1	52	3.8	[420]
S2^c^	PC_71_BM	1:1.5	−5.33		0.83	6.1	54	2.6	[420]
S3^c^	PC_71_BM	1:1.5	−5.35		0.85	8.3	60	4.0	[420]
S4^c^	PC_71_BM	1:1.5	−5.47		0.94	2.5	45	1.0	[420]
BTBDT3	PC_71_BM	1:2	−5.23	1.69	0.90	10.08	43.1	3.91	[427]
BDT‐BTF	PC_71_BM	3:1			0.85	10.48	66	5.76	[428]
SM1^d^	PC_71_BM	1:1		1.85	0.88	10.3	70	6.3	[432]
SM2	PC_71_BM	2:3		1.98	0.92	8.8	69	5.6	[432]
SM3	PC_71_BM	3:2		2.01	0.84	4.4	56	2.0	[432]
BDTQ‐BDT(EH)^e^	PC_71_BM	1:2	−5.36		0.83	4.50	32	1.20	[434]
BDTQ‐BDT(OC)^e^	PC_71_BM	1:2	−5.30		0.79	3.52	30	0.83	[434]
SM‐2F	PC_71_BM	1:1.5	−5.12		0.75	11	44.9	3.94	[436]
SM‐4F	PC_71_BM	1:1.5	−5.13		0.77	9.1	46.7	3.48	[436]

^A^ Weight ratio.

^B^ The HOMO energy level of polymer donors.

^C^ Estimated from the absorption edge in film ($E_{g}^{opt}=1240/\lambda_{onset}$).

^D^ Average *PCE* of OSCs.

^a^ CB SVA treatment.

^b^ SVA treatment for 60 s.

^c^ 0.5% 1‐chloronaphthalene.

^d^ 0.2% DIO.

^e^ 3% DIO.

Based on main‐chain engineering with electronegative end‐capping group, three novel SMDs BDTT‐F‐R (2017), BDTTS‐Cl‐R (2017) and BDTTS‐Br‐R (2017) were synthesized by Ji and co‐workers, synthesizing by introducing “F”, “Cl”, or “Br” atoms substituted thiophene side chains into the BDT unit, respectively.^[^ ^385^ ^]^ After applying for OSC devices, with the deepening of molecular polarity, the HOMO energy level of SMDs gradually decreases, thus the *V_oc_* in OSC devices increases continuously. Moreover, compared with BDTTS‐Br‐R: PC_71_BM‐based OSC, the higher *PCE* achieved by BDTTS‐Cl‐R: PC_71_BM‐based OSC verifying that the steric hindrance of side‐chain engineering is also one of the important factors affecting the photovoltaic performance of OSC devices. Notably, these characterization results were consistent with the above‐conclusion in polymer donors. Afterward, BTR (2019) and BDT‐Cl (2019) were reported by Chen and co‐workers.^[^ ^388^ ^]^ compared with BTR, BTR‐Cl with the substitution of chlorinated thiophen side chain effectively enhances the *J_sc_* (24.17 mA cm^−2^) and *FF* (65.5%) in the BTR: Y6‐based OSC, which can be attributed to the accelerated hole/electron mobility and optimized blend film morphology. Likewise, between the characterization results of B1 (2019)^[^ ^389^ ^]^ based and SM‐BF1 (2021)^[^ ^390^ ^]^ based OSCs, which has also exhibited a consistent conclusion. Therefore, chlorination is an efficient side‐chain engineering. Furthermore, after adjusting the substitution sites of “Cl” atoms in side‐chain engineering, SM‐BF2 (2021) based OSC presents a significantly decreased photoelectric performance compared with SM‐BF1: Y6‐based OSC,^[^ ^390^ ^]^ reminding us that the possible damage to the photovoltaic performance of OSC will be caused by the expansion of steric hindrance in side‐chain engineering. Moreover, based on the reported results of SMBDT‐S (2021),^[^ ^394^ ^]^ SMBDT‐SF (2021),^[^ ^394^ ^]^ BDT‐UF (2022),^[^ ^370^ ^]^ BDT‐DF (2022),^[^ ^370^ ^]^ C‐F (2022)^[^ ^391^ ^]^ and C‐2F (2022),^[^ ^391^ ^]^ these SMDs were synthesized to further explore the potential application of fluorinated side chains. After adjusting the substitution sites of “F” atoms as well as fluorination degree in side‐chain engineering, the higher *PCE* obtained by BDT‐UF: N3‐based (14.8%) and C‐2F: N3‐based (14.64%) OSCs were also consistent with the characterization results in fluorinated polymer donors, verifying the reasonable application of introducing fluorinated side chains.

In addition, among such SMDs synthesized based on the main‐chain engineering with electron‐donating end‐capping groups, BTBDT3 (2014),^[^ ^427^ ^]^ BDT‐BTF (2014),^[^ ^428^ ^]^ SM1 (2016),^[^ ^432^ ^]^ SM2 (2016),^[^ ^432^ ^]^ SM3 (2016),^[^ ^432^ ^]^ SM‐2F (2019),^[^ ^436^ ^]^ SM‐4F (2019),^[^ ^436^ ^]^ BDTQ‐BDT(EH) (2019)^[^ ^434^ ^]^ and BDTQ‐BDT(OC) (2019)^[^ ^434^ ^]^ were synthesized by adjusting the degree of fluorination in acceptor unit; S1 (2016),^[^ ^420^ ^]^ S2 (2016),^[^ ^420^ ^]^ S3 (2016),^[^ ^420^ ^]^ S4 (2016)^[^ ^420^ ^]^ were designed to explore end‐capping groups with different degree of fluorination. Although compared to SMDs synthesized with electronegative end‐capping groups, the photovoltaic performance in OSC devices prepared based on the SMDs with electronegative end‐capping groups is still not ideal, both types of OSCs can be positively optimized by utilizing fluorination in the acceptor or end‐capped groups.

#### 4.2.2 Substitution of alkyl, alkoxy and alkylthio groups

**FIGURE 35:**
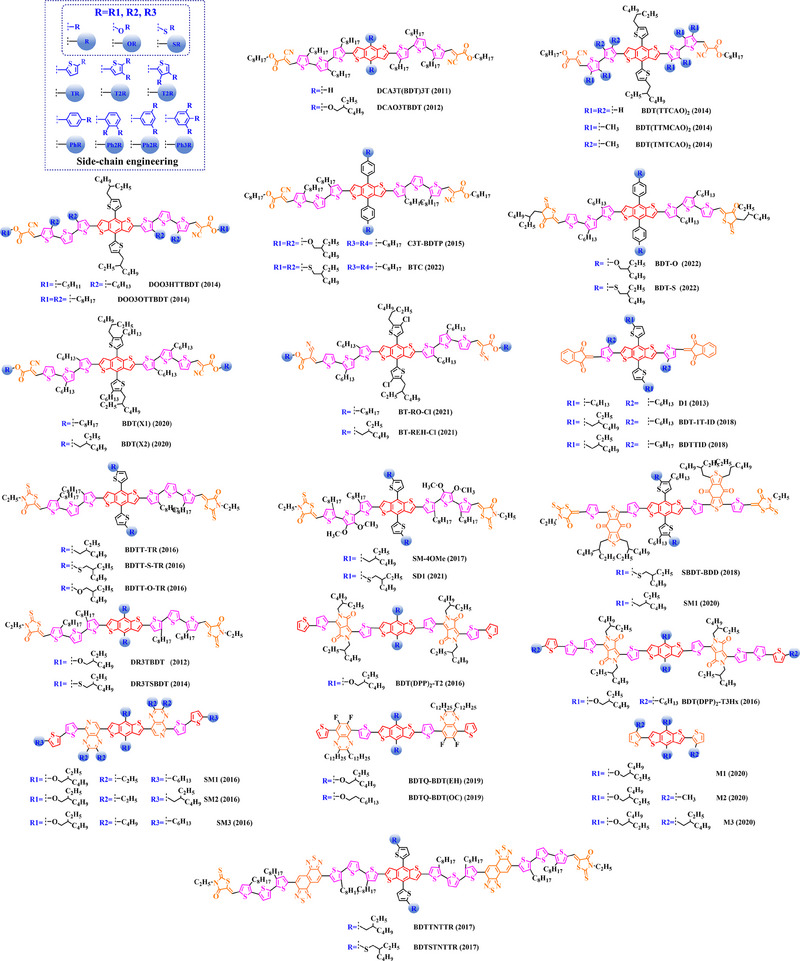
The detailed molecular structure of SMDs synthesized by introducing alkyl, alkoxy and alkylthio groups.

In order to further optimize the photoelectric performance of OSC devices, the molecular design strategy of introducing a series of functional groups (e.g. alkyl, alkoxy and alkylthio groups) also exhibits excellent applicability. In this chapter, based on the detailed photoelectric parameters (Table 32) in such representative SMDs (Figure 35) based OSC devices, we explore in depth the potential correlation between them.

**TABLE 32:** Photovoltaic parameters of OSCs related to Figure 35.

Donor	Acceptor	D/A^A^	HOMO [eV] ^B^	$E_{g}^{opt}$ [eV] ^C^	*V_OC_* [V]	*J_SC_* [mA cm^−2^]	*FF* [%]	*PCE* [%] ^D^	Ref.
BDT(TTCAO)_2_	PCBM	1:0.7	−5.30	1.89	1.08	2.57	47.3	1.31	[357]
BDT(TMTCAO)_2_	PCBM	1:0.5	−5.25	1.87	1.08	6.26	54.0	3.66	[357]
BDT(TTMCAO)_2_	PCBM		−5.23	1.84					[357]
C3T‐BDTP^(a,b)^	PC_71_BM	1:1	−5.13	1.79	0.908	9.65	60.1	5.16	[359]
BT‐RO‐Cl	Y6		−5.41	1.84	0.865	22.50	68.59	13.20	[361]
BT‐REH‐Cl	Y6		−5.41	1.83	0.868	22.93	69.86	13.52	[361]
BTC	Y6	2:1	−5.37		0.795	18.25	49.74	7.20	[363]
DCAO3T(BDT)3T	PC_61_BM	1:0.5	−5.11	1.83	0.93	9.77	59.9	5.44	[364]
DOO3HTTBDT^c^	PC_71_BM	1:1.2	−5.11	1.77	0.87	9.94	65	5.64	[364]
DCAO3TBDT	PC_61_BM	1:0.5	−5.04	1.84	0.95	8.00	60.0	4.56	[365]
DR_3_TBDT^d^	PC_71_BM	1:0.8	−5.02	1.75	0.93	12.21	65.0	7.38	[365]
DOO3OTTBDT^e^	PC_71_BM	1:1.2	−5.19	1.76	0.94	8.0	70	5.26	[365]
SD1	Y6‐T		−5.08		0.88	18.23	63.1	9.87	[368]
BDT(X1)	PC_71_BM		−5.29		0.96	10.71	69	6.77	[369]
BDT(X1)	IDIC		−5.29		0.92	13.26	71	8.27	[369]
BDT(X2)	PC_71_BM		−5.32		1.00	12.61	69	8.41	[369]
BDT(X2)	IDIC		−5.32		0.94	12.90	58	6.49	[369]
DR3TSBDT^f^	PC_71_BM	1:0.8	−5.07	1.74	0.96	14.45	73.0	9.60	[378]
BDTT‐TR	PC_70_BM	1:0.8	−5.17	1.73	0.93	11.75	68.1	7.21	[382]
BDTT‐S‐TR	PC_70_BM	1:0.8	−5.18	1.73	0.97	13.45	70.5	9.01	[382]
BDTT‐O‐TR	PC_70_BM	1:0.8	−5.14	1.73	0.90	11.03	65.5	6.26	[382]
SM‐4OMe	PC_71_BM	1:1.5	−5.25		0.80	2.20	45.21	0.81	[393]
BDTTNTTR	PC_71_BM		−5.29		0.89	15.70	71.7	9.84	[401]
BDTSTNTTR	PC_71_BM		−5.35		0.93	16.21	76.5	11.22	[401]
SBDT‐BDD^g^	IDIC	1:1	−5.25		0.97	15.13	61.1	8.9	[402]
SM1^h^	ITIC‐4F	1:1	−5.37	1.79	0.83	2.28	21	0.41	[403]
BDT‐O^i^	Y6‐BO		−5.22	1.77	0.817	23.07	64.02	11.84	[406]
BDT‐S^j^	Y6‐BO		−5.31	1.78	0.832	25.14	68.30	14.03	[406]
D1	PC_70_BM	1.5:1	−5.19	1.61	1.03	10.07	54.7	5.67	[409]
BDT‐1T‐ID	PC_71_BM	3:1	−5.23		1.06	11.0	49	5.7	[410]
BDTTID^k^	PC_70_BM	7:3	−5.36	1.74	1.03	10.20	53	5.54	[411]
BDT(DPP)_2_‐T2	PCBM		−5.19	1.77	0.78	9.72	44.2	3.36	[423]
BDT(DPP)_2_‐T3Hx	PCBM		−5.26	1.70	0.68	4.46	65.6	1.99	[423]
M1	C60	1:1.5	−5.3	2.73	0.80	2.67	28	0.76	[425]
M2	C60	1:1.5	−5.3	2.72	0.75	4.34	30	1.20	[425]
M3	C60	1:1.5	−5.3	2.76	0.69	2.73	34	0.79	[425]
SM1^l^	PC_71_BM	1:1			0.75	6.7	42	2.1	[431]
SM2^m^	PC_71_BM	1:1			0.71	3.6	37	0.9	[431]
SM3^m^	PC_71_BM	1:1			0.81	5.7	49	2.3	[431]
BDTQ‐BDT(EH)^n^	PC_71_BM	1:2	−5.36		0.83	4.50	32	1.20	[434]
BDTQ‐BDT(OC)^n^	PC_71_BM	1:2	−5.30		0.79	3.52	30	0.83	[434]

^A^ Weight ratio.

^B^ The HOMO energy level of polymer donors.

^C^ Estimated from the absorption edge in film ($E_{g}^{opt}=1240/\lambda_{onset}$).

^D^ Average *PCE* of OSCs.

^a^ 0.4% DIO.

^b^ PDIN layer.

^c^ 70°C annealing for 10 min.

^d^ 0.2 mg mL^−1^ PDMS.

^e^ 0.25% DIO.

^f^ thermal annealing and SVA treatments.

^g^ CS_2_ SVA treatment for 45 s.

^h^ 150°C annealing for 10 min.

^i^ 0.25% DIO.

^j^ 100°C annealing for 10 min and 0.25% DIO.

^k^ 1.5% DIO.

^l^ 1% DIO.

^m^ 0.5% DIO.

^n^ 3% DIO.

Among such SMDs with electronegative end‐capping units, BDT(TTCAO)_2_ (2014) and BDT(TTMCAO)_2_ (2014) were prepared to explore the application effect of alkyl chain.^[^ ^357^ ^]^ Interestingly, with the introduction solubilized alkyl chain into the π‐bridge unit, the enhanced *J_sc_* (6.26 mA cm^−2^) and *FF* (54.0%) endows BDT(TMTCAO)_2_: PCBM‐based OSC with a higher *PCE* (3.66%) than PBDT(TTCAO)_2_: PCBM‐based OSC, which is consistent with the characterization results in polymer donors. Subsequently, considering the reasonability of introducing solubilized alkyl chains as side‐chain engineering, D1 (2013),^[^ ^409^ ^]^ DOO3HTTBDT (2014),^[^ ^364^ ^]^ DOO3OTTBDT (2014),^[^ ^365^ ^]^ BDT‐1T‐ID (2018),^[^ ^410^ ^]^ BDTTID (2018),^[^ ^411^ ^]^ BDT(X1) (2020),^[^ ^369^ ^]^ BDT(X2) (2020),^[^ ^369^ ^]^ BT‐RO‐Cl (2021)^[^ ^361^ ^]^ and BT‐REH‐Cl (2021)^[^ ^361^ ^]^ were also successfully synthesized in subsequence. Among them, compared with D1: PC_70_BM‐based, BDT(X1): PC_71_BM‐based and BT‐RO‐Cl: Y6‐based OSCs, BDT‐1T‐ID: PC_71_BM‐based, BDT(X2): PC_71_BM‐based and RT‐REH‐Cl: Y6‐based OSCs achieved a higher *J_sc_* and *FF* in subsequence, reminding us that introducing branched alkyl side chain is more conducive to optimize the miscibility of SMDs than linear alkyl side chain in the active layer. Furthermore, DOO3HTTBDT with the substitution of more suitable solubilized alkyl chain in π‐bridge unit also achieves a higher *PCE* in DOO3HTTBDT: PC_61_BM‐based OSC (5.64%) than DOO3OTTBDT: PC_61_BM‐based OSC (5.26%). Moreover, based on the functional groups such as alkyloxy and alkylthio side chains, DCAO3T(BDT)3T (2011) and DCAO3TBDT (2011) were first reported by Liu et al.^[^ ^364^ ^]^ and Zhou et al.^[^ ^365^ ^]^ in turn. Afterward, DR3TBDT (2012)^[^ ^365^ ^]^ and DR3TSBDT (2014)^[^ ^378^ ^]^ were prepared by introducing side chains alkyloxy and alkylthio in turn. Similarly, C3T‐BDTP (2015)^[^ ^359^ ^]^ and BTC (2022),^[^ ^363^ ^]^ BDT‐O (2022)^[^ ^406^ ^]^ and BDT‐S (2022)^[^ ^406^ ^]^ were also successfully constructed. Notably, due to the introduction of alkylthio effectively improves the electron‐donating of BDT unit, the accelerated hole/electron mobility endows BDT‐S: Y6‐BO‐based and BDTT‐S‐TR: PC_70_BM‐based OSCs with a higher *J_sc_* and *FF* than BDT‐O: Y6‐BO‐based and BDTT‐O‐TR: PC_70_BM‐based OSCs. Between the characterization results of BDTT‐TR (2016) based,^[^ ^382^ ^]^ BDTT‐S‐TR (2016) based^[^ ^382^ ^]^ and BDTT‐O‐TR (2016) based^[^ ^382^ ^]^ OSCs, the highest *PCE* exhibited by PDTT‐S‐TR: PC_70_BM‐based OSC further verifying the above‐conclusion. Furthermore, compared with SM‐4OMe (2017): PC_71_BM‐based and SM1 (2020): ITIC‐4F‐based OSCs,^[^ ^394^, ^403^ ^]^ the significantly enhanced *J_sc_* and *FF* obtained by SD1 (2021): Y6‐T‐based and SBDT‐BDD (2018): IDIC‐based OSCs can be attributed to introducing alkylthio side chain effectively enhancing the electron‐donating of BDT unit.^[^ ^368^, ^402^ ^]^

As for such SMDs synthesized based on the main‐chain engineering with electron‐donating end‐capping group, BDT(DPP)_2_‐T2 (2016)^[^ ^423^ ^]^ and BDT(DPP)_2_‐T3Hx (2016)^[^ ^423^ ^]^ were constructed to explore the potential effect of introducing alkyloxy side chains. Afterward, SM1 (2016), SM2 (2016) and SM3 (2016) were also synthesized by introducing different types of alkyloxy and alkyl side chains.^[^ ^431^ ^]^ With the introduction of the most suitable solubilized alkyl chain into the acceptor and end‐capping groups, the best photoelectric performance achieved in SM3: PC_71_BM based OSC deserves further attention. Furthermore, BDTQ‐BDT(EH) (2019),^[^ ^434^ ^]^ BDTQ‐BDT(OC) (2019),^[^ ^434^ ^]^ M1 (2020),^[^ ^425^ ^]^ M2 (2020)^[^ ^425^ ^]^ and M3 (2020)^[^ ^425^ ^]^ were also successfully prepared by different research teams. After adjusting the substitution of alkyl chain, the better photoelectric performance presented by SM3: PC_70_BM‐based and M2: C60‐based OSCs further proving the above‐conclusion. In addition, between the characterization results of BDTTNTTR (2017): PC_71_BM‐based and BDTSTNTTR (2017): PC_71_BM‐based OSCs,^[^ ^401^ ^]^ the higher *J_sc_* and *FF* obtained by the latter is also can be attributed to the enhanced electron‐donating of BDT donor unit achieved by introducing alkylthio side chain.

#### 4.2.3 Unusual side‐chain engineering

**FIGURE 36:**
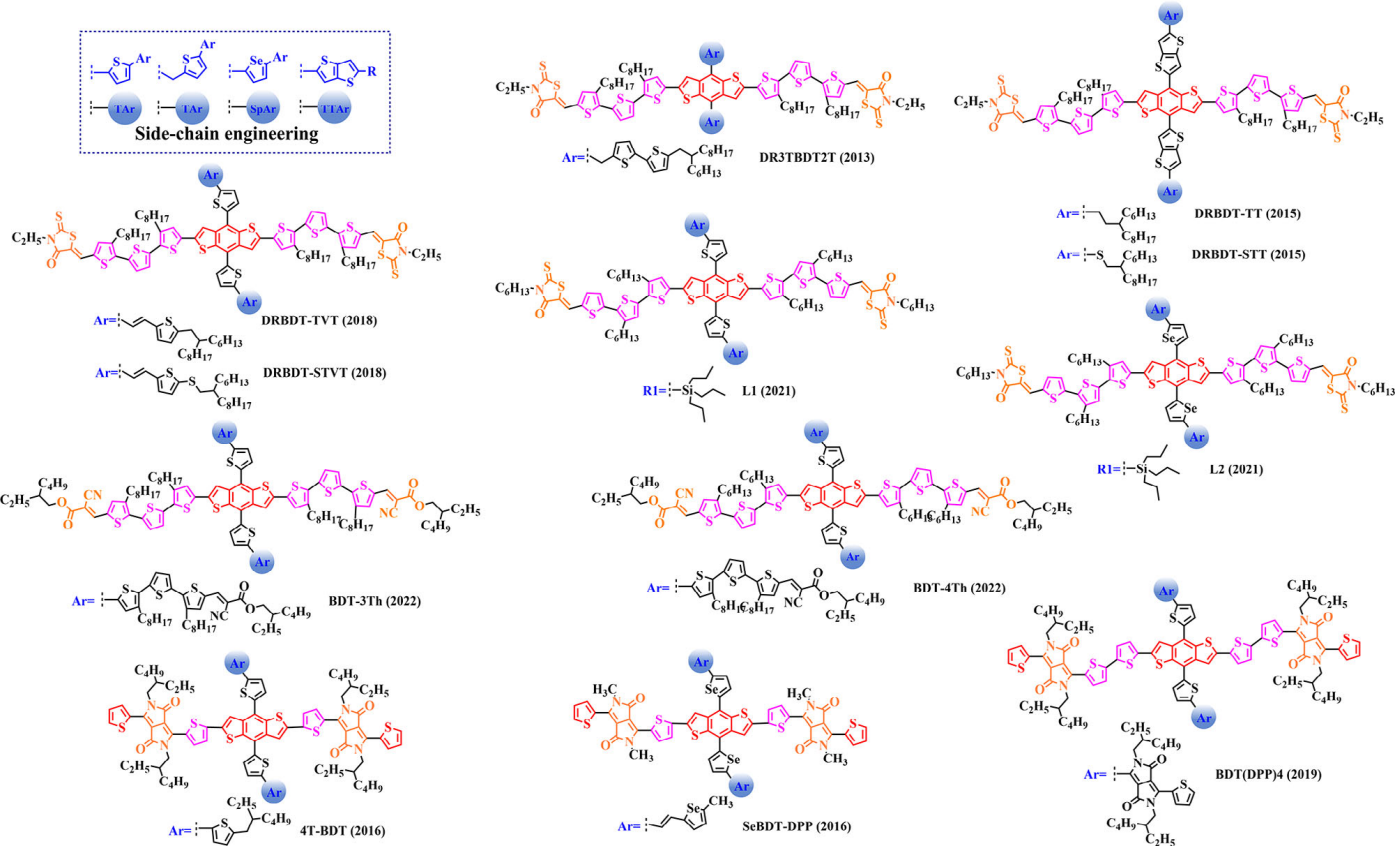
The detailed molecular structure of SMDs synthesized by introducing unusual side‐chain engineering.

Consistent with polymer donors, the introduction of a series of novel side‐chain engineering (e.g. TAr, SpAr or TTAr groups) has also greatly promoted the improvement of photovoltaic performance in SMD‐based OSC devices. Among these SMDs, the potential relationship between detailed molecular structure of SMDs (Figure 36) and photoelectric performance (Table 33) in corresponding OSCs were discussed as follows.

**TABLE 33:** Photovoltaic parameters of OSCs related to Figure 36.

Donor	Acceptor	D/A^A^	HOMO [eV] ^B^	$E_{g}^{opt}$ [eV] ^C^	*V_OC_* [V]	*J_SC_* [mA cm^−2^]	*FF* [%]	*PCE* [%] ^D^	Ref.
BDT‐3Th	Y6		−5.04	1.97	0.85	11.7	35.0	3.50	[360]
BDT‐4Th	Y6		−5.14	2.01	0.84	16.4	39.6	5.60	[360]
DR3TBDT2T^a^	PC_71_BM	1:0.8	−5.07	1.78	0.92	12.09	72.1	8.02	[377]
DRBDT‐TT^b^	PC_71_BM	1:0.8	−5.13	1.78	0.91	12.93	71.0	8.50	[381]
DRBDT‐STT^b^	PC_71_BM	1:0.8	−5.15	1.80	0.90	12.20	70.0	7.85	[381]
DRBDT‐TVT^c^	PC_71_BM		−5.11	1.70	0.879	10.73	72.76	6.67	[386]
DRBDT‐STVT^c^	PC_71_BM		−5.14	1.71	0.907	10.25	73.61	6.61	[386]
L1	Y6	1.5:1	−5.32	1.76	0.83	25.18	68.3	14.2	[392]
L2	Y6	1.5:1	−5.33	1.77	0.82	26.24	70.4	15.4	[392]
4T‐BDT	PC_61_BM		−5.21		0.799	12.17	62.1	5.93	[417]
SeBDT‐DPP^d^	PC_71_BM	1:1			0.79	10.98	58	4.96	[418]
BDT(DPP)_4_	C8‐ITIC	1:1	−5.36		0.86	10.1	45	3.6	[422]

^A^ Weight ratio.

^B^ The HOMO energy level of SMDs.

^C^ Estimated from the absorption edge in film ($E_{g}^{opt}=1240/\lambda_{onset}$).

^D^ Average *PCE* of OSCs.

^a^ 0.2 mg mL^−1^ PDMS.

^b^ SVA treatment.

^c^ CS_2_ SVA treatment for 30 s.

^d^ 2% 1‐CN.

Based on the SMDs with electronegative end‐capping group, DR3TBDT2T (2013),^[^ ^377^ ^]^ DRBDT‐TVT (2018),^[^ ^386^ ^]^ DRBDT‐STVT (2018),^[^ ^386^ ^]^ BDT‐3Th (2022)^[^ ^360^ ^]^ and BDT‐4Th (2022)^[^ ^360^ ^]^ were synthesized by introducing thiophene‐based side chains. Due to forming a highly conjugated SMD main chain, both SMDs: acceptor‐based OSCs achieve a promising *V_oc_*, especially for DR3TBDT2T: PC_71_BM‐based (0.92 V) and DRBDT‐STVT: PC_71_BM‐based (0.907 V) OSCs. Furthermore, DRBDT‐TT (2015) and DRBDT‐STT (2015) were synthesized by introducing TT‐based side chains^[^ ^381^ ^]^; L1 (2021) and L2 (2021) were explored by introducing the side chain tripropylsilane.^[^ ^392^ ^]^ Benefiting the flexible silicon‐based side chains effectively increases the miscibility of SMDs, the significantly enhanced *J_sc_* and *FF* presented by L1: Y6‐based and L2: Y6‐based OSCs verifying the reasonability of introducing the functional group tripropylsilane as side‐chain engineering.

In addition, based on the SMDs with electron‐donating end‐capping groups, 4T‐BDT (2016),^[^ ^417^ ^]^ SeBDT‐DPP (2016)^[^ ^418^ ^]^ and BDT(DPP)4 (2016)^[^ ^422^ ^]^ were also synthesized by introducing a series of novel side chains. Although an ideal photoelectric performance is not achieved in such types of SMD‐based OSC devices, the molecular design strategy of introducing unusual functional groups as side‐chain engineering is still worth exploring further.

## 5 SUMMARY AND OUTLOOK

**FIGURE 37:**
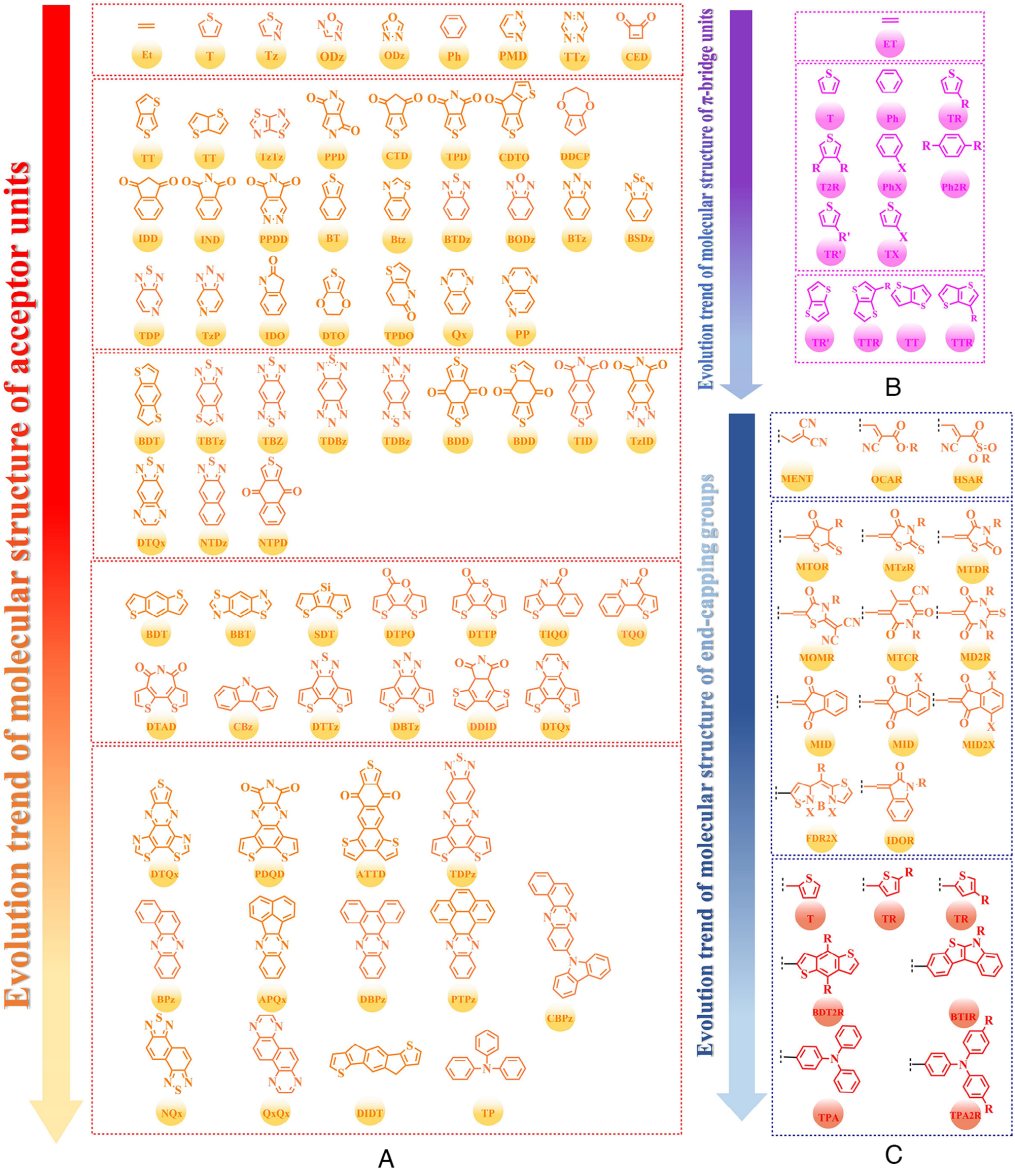
The evolution trend of (A) acceptor units; (B) π‐bridge units; and (C) end‐capping units.

Since the birth of BDT‐based polymer donor in 2008, As one of the most promising donor materials, polymer donors have significantly accelerated the development of OSC. The well‐planar and electron‐rich properties of the BDT unit promote its surprising molecular construction with other electron‐deficient units (Figure 37A) in high performance OSCs. Meanwhile, the photoelectric prosperity of donor materials was also significantly improved by the continuous optimization of the π‐bridge units (Figure 37B) and end‐capping groups (Figure 37C). However, because of the inevitable structural defects and wide molecular distribution in the polymer synthesis process, this also leads to the issue of poor repeatability of efficient polymer donor materials. Nonetheless, achieving breakthroughs in the photovoltaic performance of OSC devices through a series of molecular design strategies remains an esoteric and worthy of further exploration.

As a result, based on the characterization results of the various types of polymer donors mentioned above, this paper summarizes the potential relationship between donor materials and photovoltaic performance in corresponding OSC devices.

Among main‐chain engineering, D‐A type polymer donors based on highly conjugated polymer skeletons are frequently able to accomplish tight molecular packing and better crystallinity in the active layer, which are essential factors for achieving ideal *V_oc_* in OSC devices. Additionally, through introducing more effective donor or acceptor units, the accelerated exciton dissociation procedure and hole/electron mobility are more advantageous to form a more complementary spectral absorption between polymer donor and acceptor, endowing corresponding OSC devices with an ideal *J_sc_*, which can be associated to synergistic effect of strong electron‐donating and electronegativity in the polymer backbone.

In terms of side‐chain engineering, the introduction of functional groups in the polymer donor is frequently conducive to further optimizing the HOMO energy level and miscibility of donor materials as well as blend film morphology, thus effectively increasing *J_sc_* and *FF* in photovoltaic devices on the assumption of ensuring the highly conjugated structure of the polymer backbone. However, as previously mentioned, side‐chain engineering frequently results in irreversible damage to other photovoltaic performance even though it may be very simple to improve a single photovoltaic parameter. Therefore, there are still plenty of challenges to overcome in order enhance OSC devices’ photovoltaic performance in a balanced and stable way.

Moreover, it is also important to note that the primary trend for polymer donors in the future is the molecular design strategy of generating unique polymer donors by using multi‐component copolymerization and incorporating BDT‐derived donor units.

**FIGURE 38:**
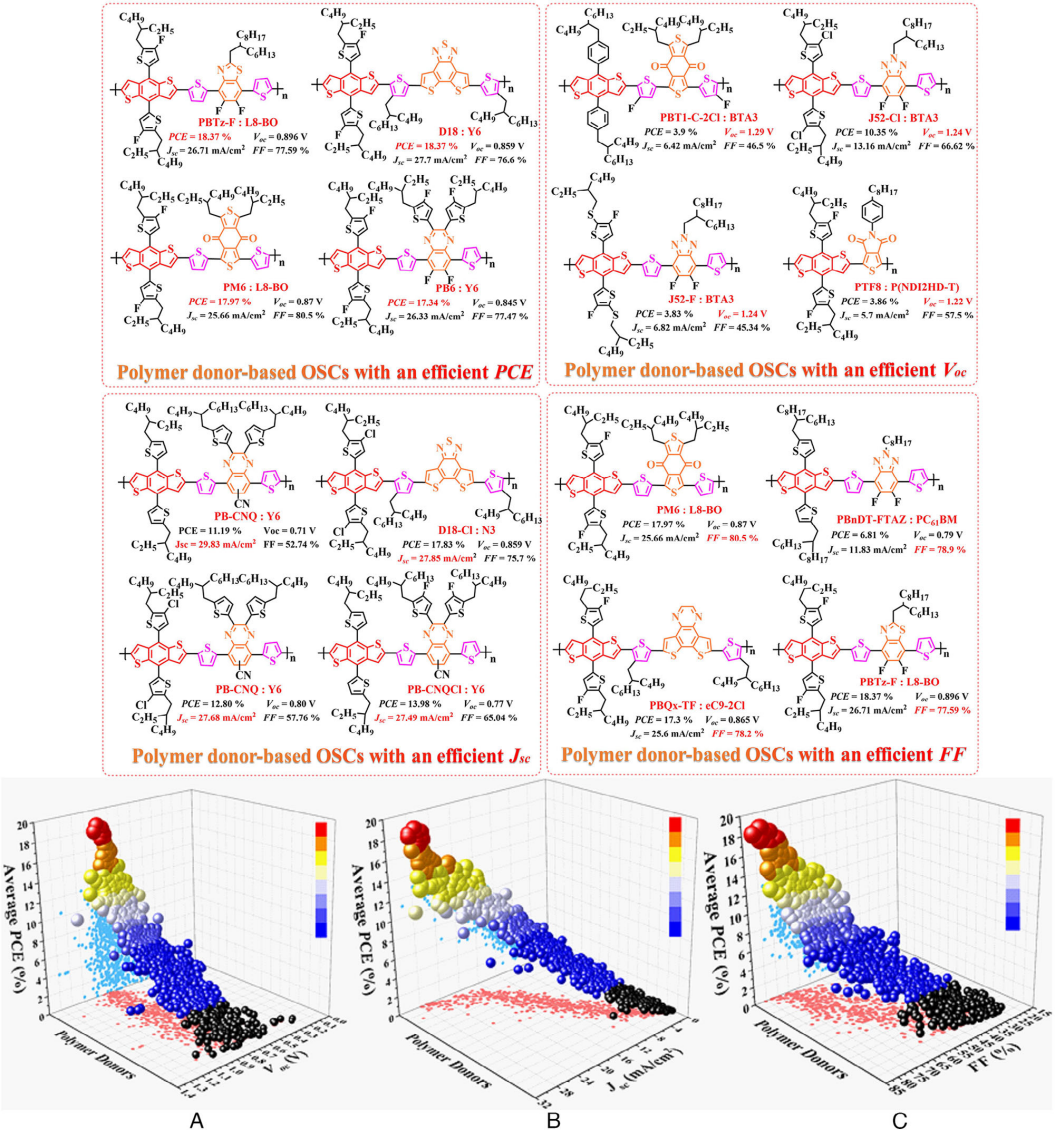
The potential relationship of *V_oc_*, *J_sc_* and *FF* in polymer donor based OSCs: (A) *V_oc_* and *PCE*; (B) *J_sc_* and *PCE*; (C) *FF* and *PCE*.

The relationship between different types of representative polymer donors and photovoltaic performance in related OSC devices is also summarized in this study. Remarkably, *J_sc_* and *FF* are not prominent in a variety of OSC devices with high *V_oc_* (Figure 38A). Indeed, High *FF* and *J_sc_* (Figure 38B,C) ensured the achievement of high *PCE* despite the OSC based on such high‐performance polymer donors failed to achieve an ideal *V_oc_*.

**FIGURE 39:**
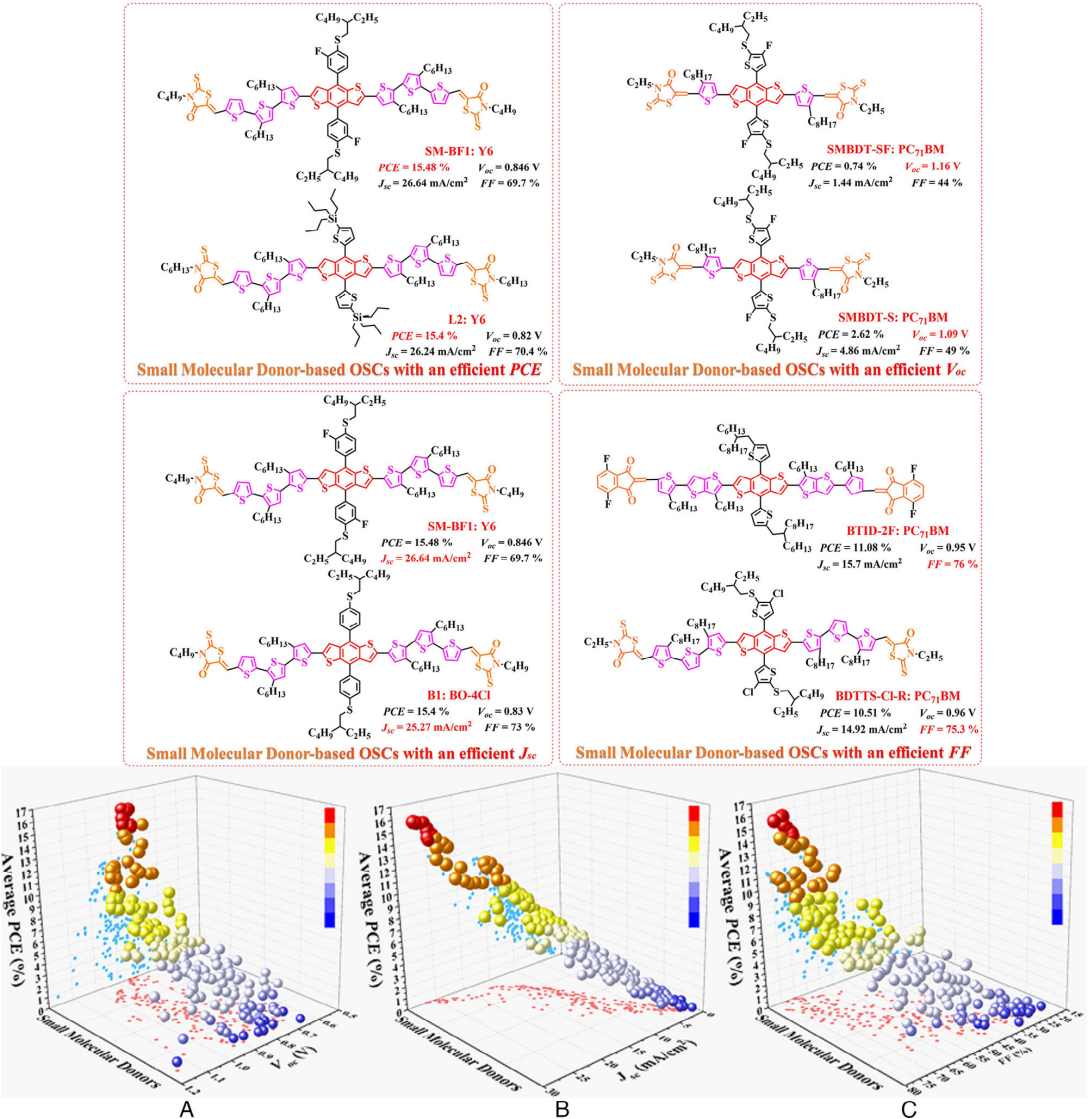
The potential relationship of *V_oc_*, *J_sc_* and *FF* in SMD based OSCs: (A) *V_oc_* and *PCE*; (B) *J_sc_* and *PCE*; (C) *FF* and *PCE*.

Moreover, compared with polymer donors, SMDs, because of their precise molecular structure and stable molecular weight, address the problem of poor reproducibility that results from a large number of defects in the molecular structure and wide molecular weight distribution of polymer donors. However, although SMDs effectively ensure that the *V_oc_* in corresponding OSCs typically lies at a relatively high level (>0.6 V) owing to their high conjugated main chain structure (Figure 39A), weaker exciton dissociation and transport compared to polymer donor based OSC normally lead to a lower *J_sc_* (Figure 39B) and *FF* (Figure 39C), which seriously hinder the further breakthrough of *PCE* in SMD‐based OSC devices. However, the photovoltaic performance of SMD‐based OSC devices has also been enhanced to some extent after applying a variety of effective molecular design techniques, such as main‐chain engineering, side‐chain engineering, building asymmetric molecular structures of SMDs and introducing BDT‐derived groups as donor units.

In addition, we should also note that these donor molecules exhibit excellent application in photovoltaic devices through appropriate molecular design strategies. Among them, a series of new D‐π‐A‐π type polymer donors and SMDs obtained a decomposition temperature of more than 400°C, which is sufficient to prove their potential application in high temperature environment. At the same time, the high efficiency and stability of OSCs based on PDPPBDT, PBDTQEH, PBDTBPA(H)‐DPP, BDTT‐TR and BDTT‐S‐TR in a certain period of time are sufficient to further verify the rationality of developing ideal donor molecules through molecular design strategies.

It is also worth mentioning that there is also a strong correlation between the surface morphology of the active layer and the performance of the photovoltaic device, especially for the potential correlation between *RMS* and *FF*. In general, the formation of a relatively smooth and fine bi‐continuous interpenetrating network morphology of the blend film is conducive to the dissociation of excitons in photovoltaic devices and the transport of electron/hole. However, we notice that in most cases, the introduction of additives will enhance the crystallinity of the blend film, but in contrast, the increase of the phase domain tends to increase the *RMS* slightly. By properly optimizing the phase separation morphology of the blend film surface, it can further promote the dissociation and transport of excitons, which deserves our further attention.

Overall, although organic solar cells fabricated using polymer donors or small molecule donors have encountered various challenges, it is optimistic that we will soon be able to develop more effective and stable donor materials via rational molecular design strategies for improving the performance and stability of OSCs, and it holds great promise for future photovoltaic applications.

## CONFLICT OF INTEREST STATEMENT

The authors declare no conflicts of interest.

## DATA AVALIBILITY STATEMENT

All data of this work are present in the article and the Sup‐porting Information. The other data that support the findings of this work are available from the corresponding author upon reasonable request.
